# Calcium-regulatory proteins as modulators of chemotherapy in human neuroblastoma

**DOI:** 10.18632/oncotarget.15283

**Published:** 2017-02-11

**Authors:** Ana-Maria Florea, Elizabeth Varghese, Jennifer E. McCallum, Safa Mahgoub, Irfan Helmy, Sharon Varghese, Neha Gopinath, Steffen Sass, Fabian J. Theis, Guido Reifenberger, Dietrich Büsselberg

**Affiliations:** ^1^ Institute of Neuropathology, Heinrich Heine University Düsseldorf, Düsseldorf, Germany; ^2^ Weill Cornell Medicine in Qatar, Qatar Foundation-Education City, Doha, Qatar; ^3^ Institute of Computational Biology, Helmholtz Zentrum München, Neuherberg, Germany

**Keywords:** neuroblastoma, cisplatin, topotecan, calcium signaling, chemotherapy

## Abstract

Neuroblastoma (NB) is a pediatric cancer treated with poly-chemotherapy including platinum complexes (e.g. cisplatin (CDDP), carboplatin), DNA alkylating agents, and topoisomerase I inhibitors (e.g. topotecan (TOPO)). Despite aggressive treatment, NB may become resistant to chemotherapy. We investigated whether CDDP and TOPO treatment of NB cells interacts with the expression and function of proteins involved in regulating calcium signaling. Human neuroblastoma cell lines SH-SY5Y, IMR-32 and NLF were used to investigate the effects of CDDP and TOPO on cell viability, apoptosis, calcium homeostasis, and expression of selected proteins regulating intracellular calcium concentration ([Ca^2+^]_i_). In addition, the impact of pharmacological inhibition of [Ca^2+^]_i_-regulating proteins on neuroblastoma cell survival was studied. Treatment of neuroblastoma cells with increasing concentrations of CDDP (0.1−10 μM) or TOPO (0.1 nM−1 μM) induced cytotoxicity and increased apoptosis in a concentration- and time-dependent manner. Both drugs increased [Ca^2+^]_i_ over time. Treatment with CDDP or TOPO also modified mRNA expression of selected genes encoding [Ca^2+^]_i_-regulating proteins. Differentially regulated genes included *S100A6, ITPR1, ITPR3, RYR1* and *RYR3*. With FACS and confocal laser scanning microscopy experiments we validated their differential expression at the protein level. Importantly, treatment of neuroblastoma cells with pharmacological modulators of [Ca^2+^]_i_-regulating proteins in combination with CDDP or TOPO increased cytotoxicity. Thus, our results confirm an important role of calcium signaling in the response of neuroblastoma cells to chemotherapy and suggest [Ca^2+^]_i_ modulation as a promising strategy for adjunctive treatment.

## INTRODUCTION

Neuroblastoma (NB) accounts for approximately 7% of pediatric malignancies and is responsible for more than 10% of cancer-related mortality in children [1]. Prognosis and treatment are determined by clinical and biological risk factors [2]. Estimated 5-year survival of patients with high-risk NB is less than 50%, despite receiving multimodal and highly aggressive treatment schemes that include surgery, high-dose polychemotherapy and radiation, as well as myeloablative treatment and immune-therapy [1].

Despite chemotherapy dose intensification, approximately 20% of neuroblastoma patients show an inadequate response to induction therapy and/or experience disease progression following initial therapy. Standard North American Children Oncology Group (COG) induction regimens include combinations of anthracyclines, DNA alkylating agents, platinum compounds and topoisomerase II inhibitors delivered every 21 days for 5 to 7 cycles. The International Society of Pediatric Oncology Europe Neuroblastoma uses a more rapid regimen in which cycles are delivered every 10 days and that demonstrated superior 5-year event-free survival of 30%, compared with 18% for standard interval chemotherapy [1, 3]. Furthermore, the topoisomerase I inhibitor topotecan has demonstrated efficacy in recurrent NB and has recently been incorporated into the COG induction regimens [4, 1]. Both, cisplatin and topotecan are currently used in the treatment of patients with recurrent neuroblastoma [1, 3, 4].

Calcium signaling controls physiologic and pathologic cellular processes. Intracellular Ca^2+^ is a universal second messenger whose concentration ([Ca^2+^]_i_) is precisely regulated. A considerable body of evidence demonstrates that changes in the expression or function of [Ca^2+^]_i_-regulating proteins (channels or active transport proteins) deregulate [Ca^2+^]_i_, which in turn may cause perturbation of important signaling pathways that govern cell homeostasis. Several anti-cancer drugs, including cisplatin (CDDP), have been shown to modulate [Ca^2+^]_i_ in cancer cells, triggering calcium-dependent cell death via apoptosis [5, 6, 7]. Nevertheless, the mechanisms by which [Ca^2+^]_i_ is elevated depend on the drug used. For example, CDDP and arsenic trioxide (As_2_O_3_) strongly elevate [Ca^2+^]_i_ and enhance apoptosis of tumor cells. However, the two drugs elevate [Ca^2+^]_i_ via different mechanism. Whilst CDDP induces extracellular Ca^2+^ uptake into the cytosol, with Ca^2+^ then being pumped into the calcium stores of the cells [5], As_2_O_3_ triggers a Ca^2+^ release from intracellular stores [8, 9]; for review see [10]. Therefore, a combination of different anticancer drugs that rise [Ca^2+^]_i_ by different mechanisms may improve efficiency of induced tumor cell death, For e.g. this was shown in neuroblastoma cells by treating cell cultures first with CDDP, triggering Ca^2+^ uptake and intracellular storage of Ca^2+^, followed by application of As_2_O_3_ that led to an abrupt release of Ca^2+^ from the intracellular stores [11].

Microarray and quantitative real-time PCR (qRT-PCR) analyses in combination with bioinformatics approaches can determine gene expression profiles that may elucidate the cellular response to anticancer drugs and identify gene signatures that may predict the development of drug resistance. For instance, gene expression profiling studies have revealed distinct gene expression signatures in neuroblastoma tissue samples linking the expression of several calcium signaling-associated genes to patient prognosis [12]. In the microarray-based Neuroblastoma Database reported by Chen and colleagues (2008) [12], the authors identified genetic signatures of 160 top-regulated genes linked to patient prognosis [12]. In addition, genes associated with mitochondria, cell metabolism and cell cycle were highlighted as potential therapeutic targets [12]. In another study, the importance of alternative splicing following high-level amplification of the *MYCN* gene in neuroblastoma has been explored [13].

In this study we investigated changes in expression of selected genes whose gene products are directly linked to the regulation of calcium dynamics in established neuroblastoma cell line models following treatment with the clinically important drugs CDDP and topotecan. We used database interrogation of the microarray-based Neuroblastoma Database [12] to identify and select a limited number of potential [Ca^2+^]_i_ signaling-related molecules that might be of relevance in neuroblastoma, including inositol triphosphate receptors I and III (*ITPR1*, *ITPR3*), ryanodine receptors 1 and 3 (*RYR1*, *RYR3*), and the S100 calcium-binding protein A6 (*S100A6*). To determine the relationship between gene expression and function of these selected [Ca^2+^]_i_ signaling regulators in the drug sensitivity of neuroblastoma cells, we incorporated cytotoxicity and apoptosis assays, live-cell calcium imaging, gene and protein expression analyses and functional assays.

## RESULTS

### CDDP and TOPO decrease viability of neuroblastoma cells in a time- and concentration-dependent manner

**Figure 1:**
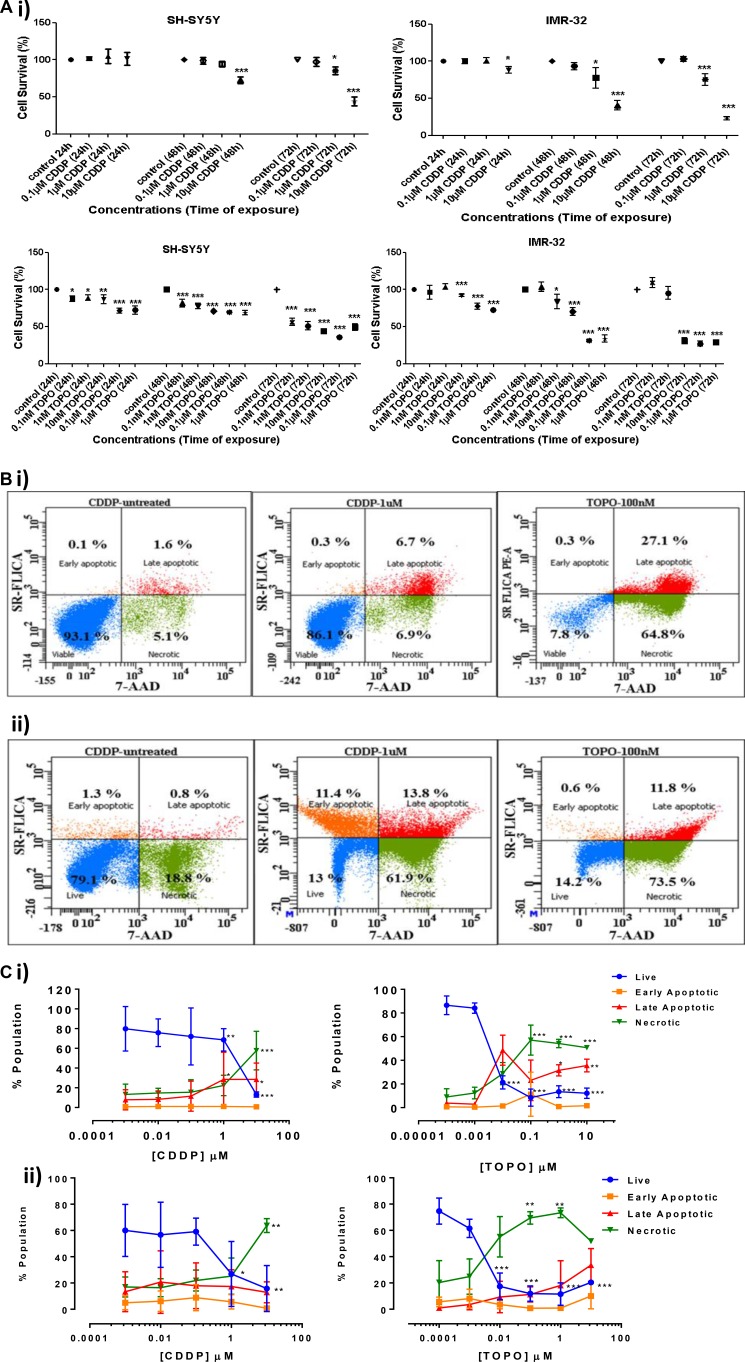
Cell survival and apoptosis in neuroblastoma cells following CDDP or TOPO treatment (**A**) Cell survival detected by the trypan blue exclusion test following exposure to 0.1 μM-10 μM CDDP and 0.1 nM-1 μM TOPO for 24, 48 and 72 h in SH-SY5Y (i) and IMR-32 cells (ii). Shown are three independent experiments each (*n* = 3). (**B**) Examples of representative scatter plots outlining the population distributions (live, early apoptotic, late apoptotic and necrotic) of untreated, CDDP-treated (1 μM) and TOPO-treated (100 nM) SH-SY5Y (i) and IMR-32 (ii) cells as detected by FACS analysis following 72 h of drug exposure using a total cytotoxicity kit with fluorescent markers 7-amino actinomycin D (7-AAD) and sulforhodamine flurochrome labeled inhibitors of apoptosis (SR-FLICA) (ImmunoChemistry Technologies). (**C**) Quantification of cell apoptosis and necrosis via FACS analysis in SH-SY5Y (i) and IMR-32 (ii) cells incubated with different concentrations of CDDP (0.001 μM-10 μM) or TOPO (100 pM–10 μM) at 72 h. Shown are three independent experiments each (*n* = 3). Statistical significance is relative to untreated v's treated conditions and is considered if *p* < 0.05 (*), *p* < 0.01 (**), *p* < 0.001 (***) when assessed via a One-Way ANOVA (C) and Two-Way ANOVA (A) tests with Dunnett's Test for multiple comparisons.

In human neuroblastoma cell lines we tested the sensitivity to cisplatin (CDDP) and topotecan (TOPO) (Figure 1A and 1B). CDDP and TOPO triggered cell toxicity in a concentration- and time-dependent manner. A significant decrease of cell viability was observed after 48 h of exposure of SH-SY5Y cells to 10 μM CDDP and after 72 h exposure of SH-SY5Y cells to 1 and 10 μM CDDP (*p* < 0.01; *p* < 0.001) (Figure 1Ai). IMR-32 neuroblastoma cells were more sensitive to CDDP, showing a significant decrease in cell viability after treatment with 10 μM CDDP for 24 h (*p* < 0.05); 1 and 10 μM CDDP for 48 h (*p* < 0.05 and *p* < 0.001) and 72 h (*p* < 0.001 and *p* < 0.001) (Figure 1Bi). A third neuroblastoma cell line, NLF, was less sensitive to CDDP, i.e., demonstrated a significant decrease in cell viability only after 48h treatment with 10 μM CDDP (*p* < 0.001; Supplementary Figure 1).

TOPO (0.1 nM to 1 μM) demonstrated a stronger cytotoxic effect compared to CDDP in all neuroblastoma cell lines tested and cell viability was significantly reduced in SH-SY5Y cell after 24 h, 48 h and 72 h of exposure (Figure 1Ai). The cytotoxic effects of TOPO were stronger in IMR-32 cells as compared with SH-SY5Y and NLF cells (Figure 1Ai and 1Bi) (Supplementary Figure 1).

### CDDP and TOPO trigger cell death, mainly by apoptosis, in a time- and concentration-dependent manner

Neuroblastoma cells treated with CDDP and TOPO showed significantly increased apoptotic and necrotic cell populations, clearly visible in the fluorescently gated representative scatter plots for SH-SY5Y (Figure 1Aii) and IMR-32 (Figure 1Bii).

The cell populations measured by FACS following 72 h of drug exposure demonstrated that the predominant mechanism of cell death was apoptosis. Measurements showed that apoptotic and necrotic cell population's increased significantly with ≥1 μM CDDP or ≥ 0.01 μM TOPO for both SH-SY5Y and IMR-32 cells (Figure 1Ci and 1Cii).

Both cell lines exhibited similar increases in apoptotic cell fractions following exposure to either drug, with a concomitant decrease in vital cell populations (*p* < 0.001). TOPO was more efficient than CDDP in inducing apoptosis in both cell lines, compared to CDDP: concentrations as low as 0.001 μM of TOPO were sufficient to significantly increase cell death by apoptosis in both SH-SY5Y and IMR-32 cells (Figure 1Ci and 1Cii).

### [Ca^2+^]_i_ increased time- and concentration-dependently with the application of CDDP or TOPO

**Figure 2:**
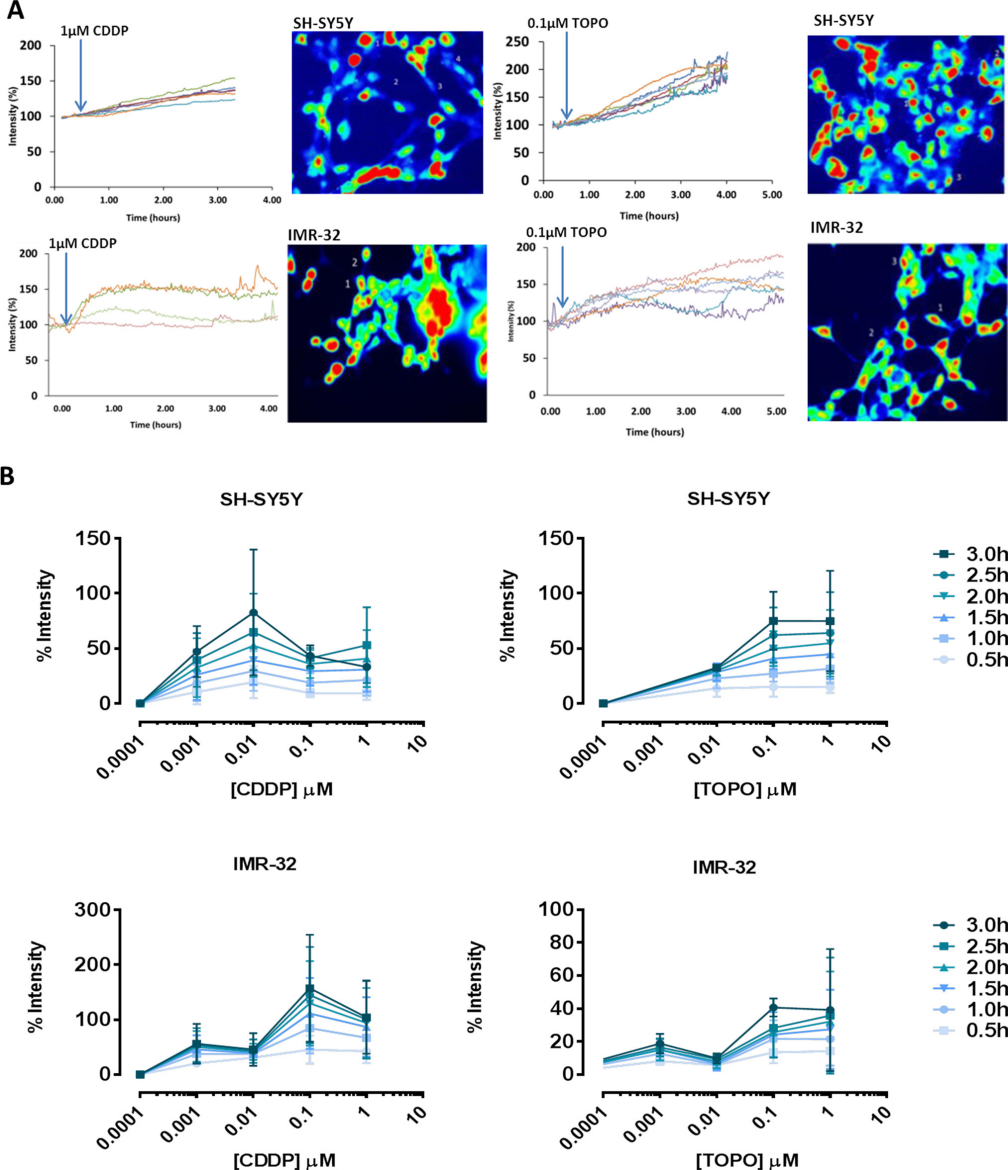
CDDP or TOPO treatment of neuroblastoma cells induces changes in [Ca^2+^]_i_ in a time- and concentration-dependent manner (**A**) Representative examples of increasing fluorescence intensity in individually selected cells following drug addition alongside corresponding images displaying subtracted cell fluorescence, highlighting accumulated calcium over 1–4 hours in SH-SY5Y and IMR-32 cells. (**B**) Increasing fluorescence intensity over a range of 0.5 h time points (0.5 h–3.0 h) following exposure to increasing concentrations of CDDP (0.001–1 μM) or TOPO (0.0001–1 μM) in SH-SY5Y and IMR-32 cells, expressed as percentage intensity relative to untreated fluorescence levels (% intensity) (shown are results of three independent experiments at each time point and drug concentration). Two-way ANOVA statistical testing indicates significant variance that is both time- and concentration-dependent, *p* < 0.001 (***) as shown in the Supplementary Table: “Statistics Figure 2B”.

Individual (but not all) neuroblastoma cells increased [Ca^2+^]_i_ time- and concentration-dependently following application of either CDDP or TOPO (0.01 μM-1 μM). Table 1 outlines the percentage of responding cells following exposure to increasing drug concentrations. Figure 2A shows representative examples of individually selected cells/ROIs increasing in fluorescence intensity over time. Only responding cells were used to analyze the increase in [Ca^2+^]_i_ (Figure 2B, statistics shown in Supplementary Files). In both SH-SY5Y and IMR-32 cells, [Ca^2+^]_i_ increased following drug exposure, reaching a steady state after 1–3 h.

**Table 1:** The percentage of neuroblastoma cells responsive to chemotherapeutic drugs via an increase in [Ca^2+^]_i_ is concentration dependent

Cell Type	Drug	Concentration (μM)	Total Cells (*n*)	Selected Cells (*n*)	Cells analyzed (%)
**SH-SY5Y**	**CDDP**	**0.001**	44	17	38.64
		**0.01**	52	40	76.92
		**0.1**	52	48	92.31
		**1**	62	50	80.65
**SH-SY5Y**	**TOPO**	**0.01**	27	22	81.48
		**0.1**	53	37	69.81
		**1**	42	31	73.81
**IMR-32**	**CDDP**	**0.001**	40	19	47.50
		**0.01**	22	13	59.09
		**0.1**	38	30	78.95
		**1**	36	25	69.44
**IMR-32**	**TOPO**	**0.001**	29	6	20.69
		**0.01**	46	26	56.52
		**0.1**	23	11	47.83
		**1**	45	29	64.44

The total and responding numbers of cells were recorded within a selected microscopic field for each experiment and the percentage of responsive cells (%) was calculated following exposure to increasing concentrations of either CDDP or TOPO.

Averaged data from at least three independent applications per concentration are illustrated in Figure 2B. There was a clear time-dependent effect of CDDP on SH-SY5Y cells concerning [Ca^2+^]_i_. However, a concentration of 0.01 μM was more effective than higher concentrations tested in this experimental setup. The rise of [Ca^2+^]_i_ was higher in IMR-32 cells than in SH-SY5Y cells following CDDP but not TOPO treatment (Figure 2B). For CDDP, this finding was mirrored by a higher sensitivity in apoptosis assays (Figure 1A and 1B). Treatment of IMR-32 cells with TOPO induced a smaller increase of [Ca^2+^]_i_ (Figure 2B).

### CDDP and TOPO deregulate mRNA expression of genes encoding [Ca^2+^]_i_-regulators

**Figure 3:**
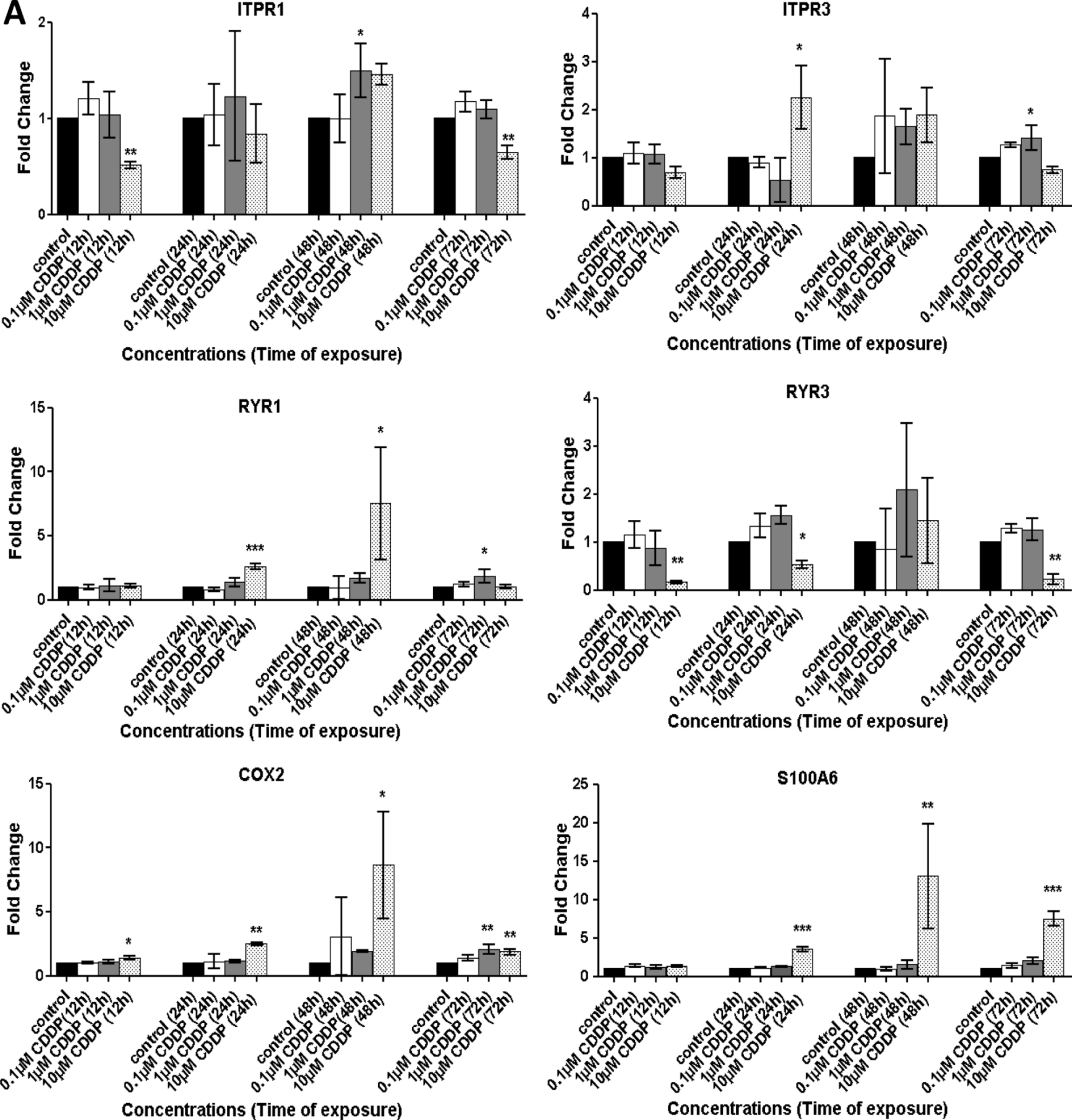

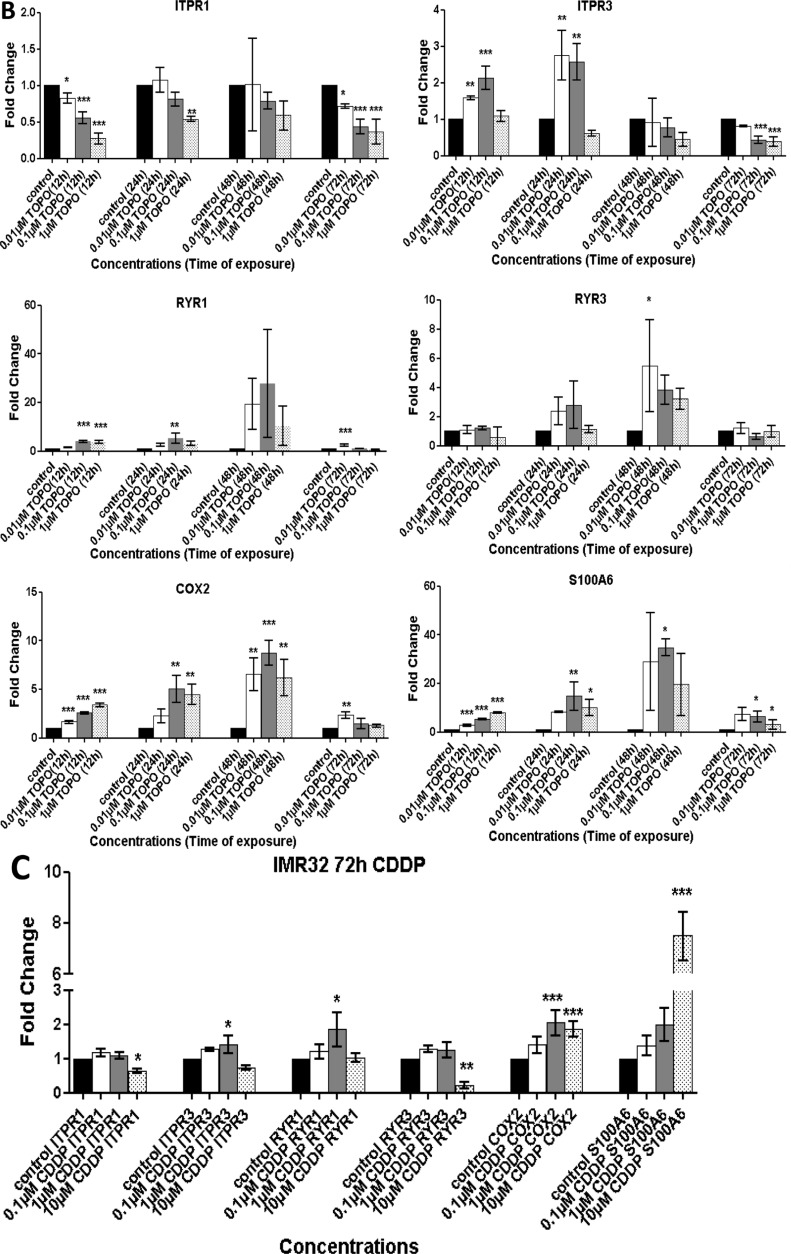
Treatment with CDDP or TOPO induces alterations in the mRNA expression of key [Ca^2+^]_i_ modulators in neuroblastoma cells Changes in mRNA expression of *ITPR1, ITPR3, RYR1, RYR3*, *S100A6* and *COX2* were assessed via qRT-PCR in SH-SY5Y cells following 12 h, 24 h, 48 h or 72 h exposure to (**A**) 0.1 μM–10 μM CDDP or (**B**) 0.001 μM–1 μM TOPO. The expression pattern in IMR-32 cells was also assessed following exposure to 0.1 μM–10 μM CDDP (**C**). Data are expressed as fold-change (RQ), relative to the untreated control and are normalized to expression of *ARF-1* mRNA as reference. Data are derived from three independent biological experiments each. Statistical significance is relative to the untreated control and considered if *p < 0.05* (*), *p < 0.01* (**), *p < 0.001* (***) as assessed by one-way ANOVA with Dunnett's test for multiple comparisons.

Next, we investigated whether the mRNA expression of selected genes encoding regulators of [Ca^2+^]_i_ (Figure 3 and Supplementary Figures 2–3) changed following exposure to CDDP (Figure 3A) or TOPO (Figure 3B) in SH-SY5Y and in IMR-32 cells (Figure 3C). qRT-PCR analysis indicated that mRNA expression of *S100A6* in SH-SY5Y cells increased with treatment of either drug (Figure 3A). This increase was paralleled by up-regulation of *COX2* mRNA expression in SH-SY5Y cells treated with CDDP and TOPO (Figure 3). This was also true for IMR-32 cells exposed to CDDP (*p* < 0.001) (Figure 3C). The increase of *COX2* mRNA expression showed similar dynamics for CDDP and TOPO treatment, i.e., up-regulation of *COX2* mRNA peaked at 48 h exposure, followed by a marked decrease in expression after 72 h (Figure 3A and 3B).

Investigation of IMR-32 cells revealed that CDDP (Figure 3C) or TOPO (Supplementary Figure 3) treatment also triggered an up-regulation of *S100A6* and *COX2* mRNA expression, thus confirming the findings with SH-SY5Y cells (Figure 3C and Supplementary Figure 3). Similarly, CDDP treatment of NLF cells caused increased expression of both transcripts (Supplementary Figure 3). For 72 h CDDP treatment, the increase in mRNA expression of *S100A6* was 8-10-fold higher compared to control conditions in all three cell lines tested (*p* < 0.001) (Figure 3A and 3C, Supplementary Figure 3). Following TOPO treatment, this increase was the strongest in SH-SY5Y (Figure 3B) followed by IMR-32 (Supplementary Figure 3) and NLF cells (data not shown).

Changes in expression of *ITPR1*, *ITPR3*, *RYR1* and *RYR3* transcripts upon treatment with CDDP or TOPO were also assessed and results indicated that *RYR1* and *RYR3* were both deregulated at the mRNA level in SH-SY5Y cells (Figure 3A and 3B). Expression of *ITPR1* and *ITPR3* transcripts was as well deregulated in SH-SY5Y neuroblastoma cells upon treatment with CDDP or TOPO (Figure 3A and 3B) this effect was also observed for both treatments in IMR-32 cells (Figure 3C and Supplementary Figure 3) and for CDDP treatment in NLF cells (Supplementary Figure 3).

### Expression of essential [Ca^2+^]_i_-regulating proteins following CDDP or TOPO treatment

**Figure 4:**
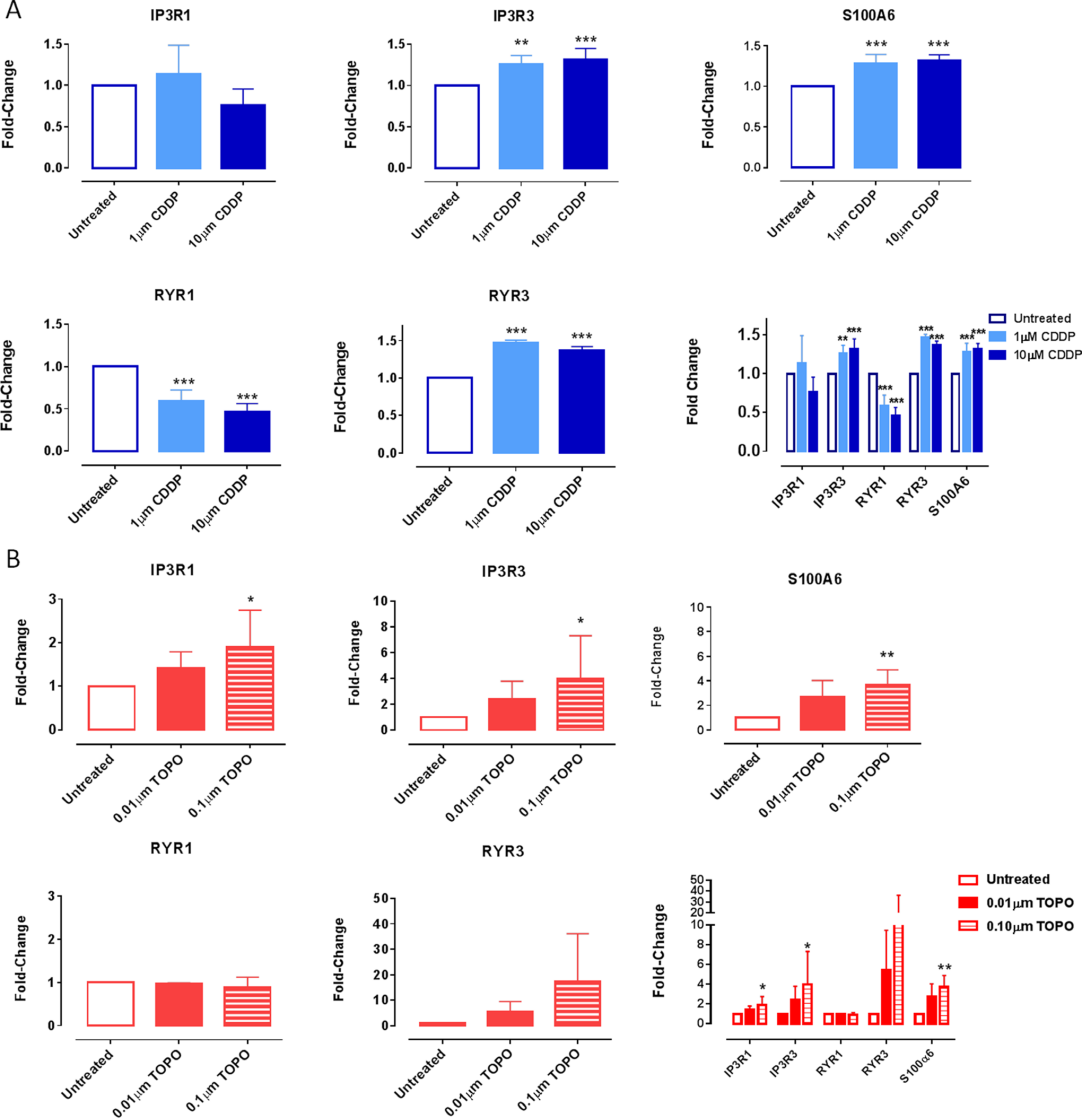
Changes in the expression of IP3R3, RYR3 and S100A6 at the protein level in SH-SY5Y cells following exposure to CDDP or TOPO SH-SY5Y cells were treated with either (**A**) CDDP (1 μM/10 μM) or (**B**) TOPO (0.01 μM/0.1 μM) for 72 hours and then harvested, fixed, permeabilized and incubated with primary antibodies specific for IP3R1, IP3R3, RYR1, RYR3 or S100A6 followed by incubation with a fluorescently conjugated (Alexa-488 nm) secondary antibody. The percentage of positive cells is individually presented for each protein or together for comparative analysis (expressed as a fold-change, relative to the untreated control). Data are derived from three independent biological experiments each. Statistical significance was calculated relative to untreated cells and considered if *p* < 0.01 (**) or *p* < 0.001 (***) as assessed by a one-way ANOVA with Dunnett's test for multiple comparisons.

**Figure 5:**
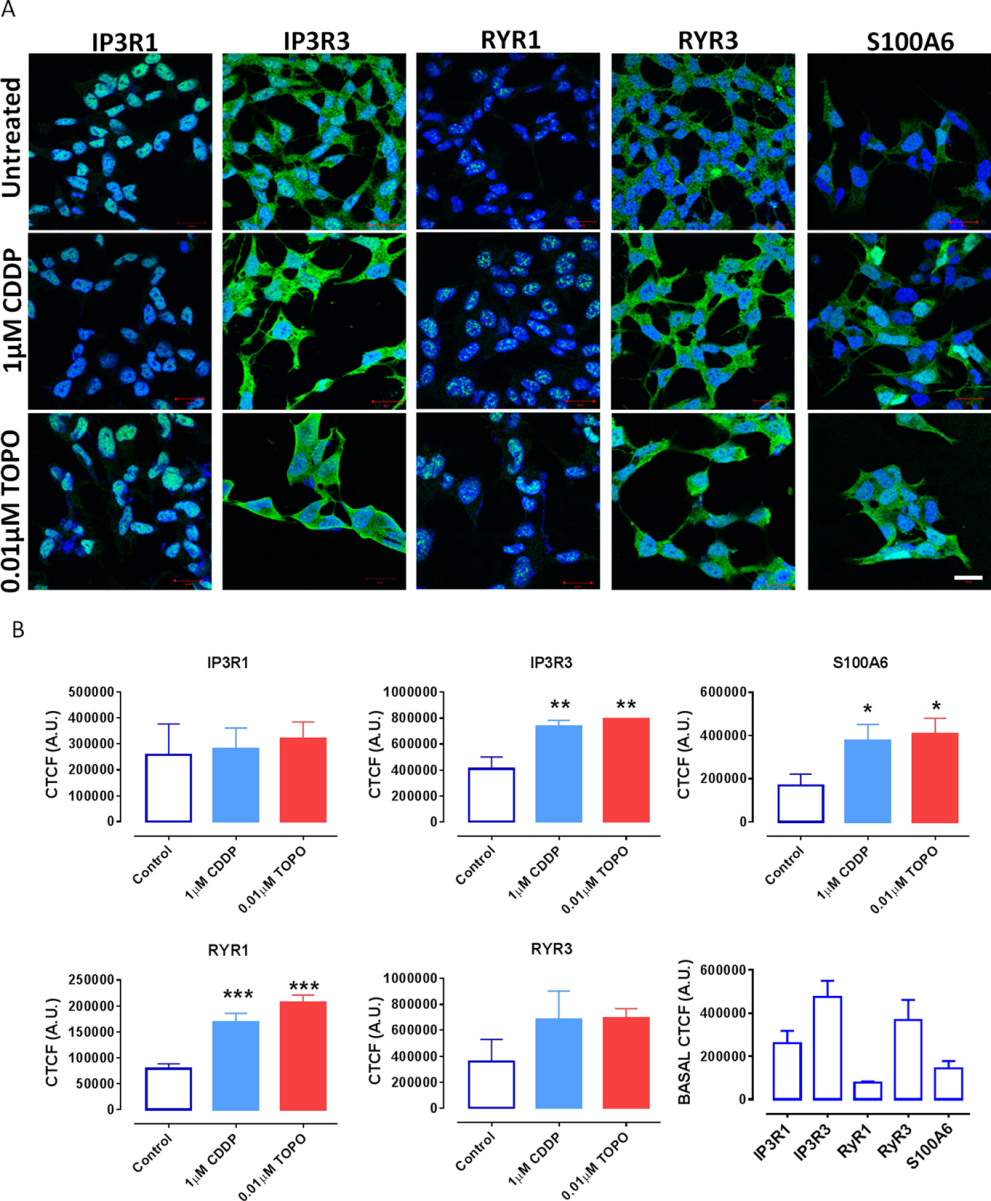
Confocal laser scanning microscopy demonstrates increased expression levels of IP3R3, RYR1 and S100A6 in SH-SY5Y cells following CDDP or TOPO treatment (**A**) Representative fluorescence confocal images of SH-SY5Y cells that endogenously express IP3R1, IP3R3, RYR1, RYR3 and S100A6 as detected by Fluo-488 nm conjugated antibodies (green fluorescence) using images taken in similar experimental set ups following exposure to either 1 μM CDDP or 0.01 μM TOPO for 72 h. Cell nuclei are counterstained with DAPI (blue). scale bar = 20 μm. (**B**) Quantification of fluorescence intensity (protein expression) expressed as mean corrected total cell fluorescence (CTCF) ± standard deviation (SD). Data are derived from three independent biological experiments each. Statistical significance is relative to untreated cells and considered if *p* < 0.05 (*), *p* < 0.01 (**) or *p* < 0.001 (***) as assessed by a one-way ANOVA with Dunnett's test for multiple comparisons.

The expression of the selected proteins was performed using FACS and confocal microscopy (Figures 4, 5, Table 2). FACS analysis in SH-SY5Y cells revealed significant increases in the expression of selective isoforms of IP3 and ryanodine receptors, specifically IP3R3 and RYR3, following treatment with CDDP at 1 μM or 10 μM (*p* < 0.001) (Figure 4A).

**Table 2:** Fold-change in protein expression levels of Ca^2+^ regulatory proteins IP3R, RYR and S100A6 in SH-SY5Y cells

	1 μM CDDP Fold-change		0.01 μM TOPO Fold-change	
**IP3R1**	**1.09**		**1.24**	
**IP3R3**	**1.79**	**	**1.93**	**
**RYR1**	**2.13**	***	**2.6**	***
**RYR3**	**1.90**		**1.93**	
**S100A6**	**2.22**	*	**2.41**	*

Quantification of protein expression (fluorescence intensity/confocal microscopy) of IP3R1, IP3R3, RYR1, RYR3 or S100A6 following exposure to either 1 μM CDDP or 0.01 μM TOPO for 72 h, expressed as a fold-change, relative to untreated cells. Statistical significance is relative to untreated cells and considered if *p* < 0.05 (*), *p* < 0.01 (**) or *p* < 0.001 (***), assessed via Student's *T*-test.

A significant increase in expression in the calcium-binding protein S100A6 was also evident at these concentrations (*p* < 0.01) (Figure 4A). We detected no significant changes in the expression levels of either RYR1 or RYR3 receptors following treatment with TOPO (Figure 4B). Expression of IP3R3 and S100A6 proteins was increased to higher levels following exposure to an increased concentration of 0.1 μM when compared to 0.01μM TOPO (Figure 4B). In contrast, higher concentrations of CDDP did not demonstrate a stronger effect on protein expression levels (Figure 4A). This was also investigated in IMR-32 cells (Supplementary Figure 4) that confirmed the up-regulation of S100A6; IP3R3 and RYR3 but showed no down-regulation of RYR1 in CDDP treated neuroblastoma cells (Supplementary Figure 4).

To confirm the results obtained by FACS analysis, we investigated the changes in the expression level of inositol trisphosphate receptors 1 and 3 (IP3R1, IP3R3), ryanodine receptors 1 and 3 (RYR1, RYR3) and the calcium-binding protein S100A6 in SH-SY5Y following treatment with CDDP or TOPO via confocal microscopy (Figure 5). In order to perform quantitative confocal analysis experiments lower drug concentrations ranging 0.01–1 μM were used for image acquirement and quantification of the protein expression since higher concentrations of chemotherapeutic agents induced cytotoxicity (Figure 5A).

The variable patterns of expression of the investigated proteins are demonstrated in Figure 5A and further highlighted in the quantification profiles of basal expression in Figure 5B. RYR1 and S100A6 proteins were expressed at very low levels, a finding in line with the FACS results showing basal levels of < 20% and < 30% positive cells, respectively (Figure 4).

IP3R3 and RYR3 were found to be most robustly expressed amongst the investigated proteins and this was also in agreement with the FACS results, demonstrating basal levels in excess of 60% positive fluorescence intensity for both proteins (Figures 4 and 5). Importantly, the quantification of expression confirmed increased protein expression levels of IP3R3 and S100A6 following treatment with low concentrations of either CDDP or TOPO. However we could not determine a significant increase in the protein expression of RYR3, that was shown with FACS experiments, while, in line with the respective mRNA data (Figure 3A), an increase of protein expression of RYR1 was determined in the confocal microscopy experiments following treatment with low concentrations of either CDDP or TOPO (Figure 5B and Table 2), as opposed to the respective FACS results (Figure 4B).

### Microarray analysis reflects the calcium-dependent activation of signaling pathways involved in p53 signaling, cell cycle control and RNA transport

We next checked whether calcium-dependent signaling in SH-SY5Y is changed upon treatment with 10 μM CDDP for 72 h using microarray-based expression profiling (See Supplementary Figure 9 and Supplementary File 1 for complete deregulated gene list). Principal component analysis revealed a clear separation of untreated (control) cells and CDDP-treated cells (See Supplementary Figure 9). Bioinformatic evaluation of the microarray data including Gene Ontology term and KEGG pathway analysis using the RAMONA software [14] confirmed that several genes involved in calcium signaling were deregulated upon CDDP treatment (Supplementary Files 2 and 3). Furthermore, exposure to CDDP deregulated several other signaling pathways including: (i) KEGG pathways - purine metabolism, p53 signaling, RNA transport, cell cycle regulation as well as caspase 8 / caspase 3-dependent apoptosis as well as (ii) GO terms - mitotic cell cycle, ncRNA metabolic process, organonitrogen compound biosynthetic process, response to toxic substances (Supplementary Files 2 and 3; hsa04110_cellcycle; hsa04115_p53; hsa05034_alcoholism). Furthermore, qRT-PCR analysis validated selected genes showing differential gene expression upon microarray analysis including *GDF15*, *PPEF1, PLCH1, PLCD3, NNAT, MYC, ABCB1, CAMTA1, ABCC1, ABCG2, COX2, S100A6, ITPR1, ITPR3*, and *RYR3* (Supplementary Files 4 and 5).

### Combinations of pharmacological modulators of calcium signaling with CDDP or TOPO enhance cytotoxicity

**Figure 6:**
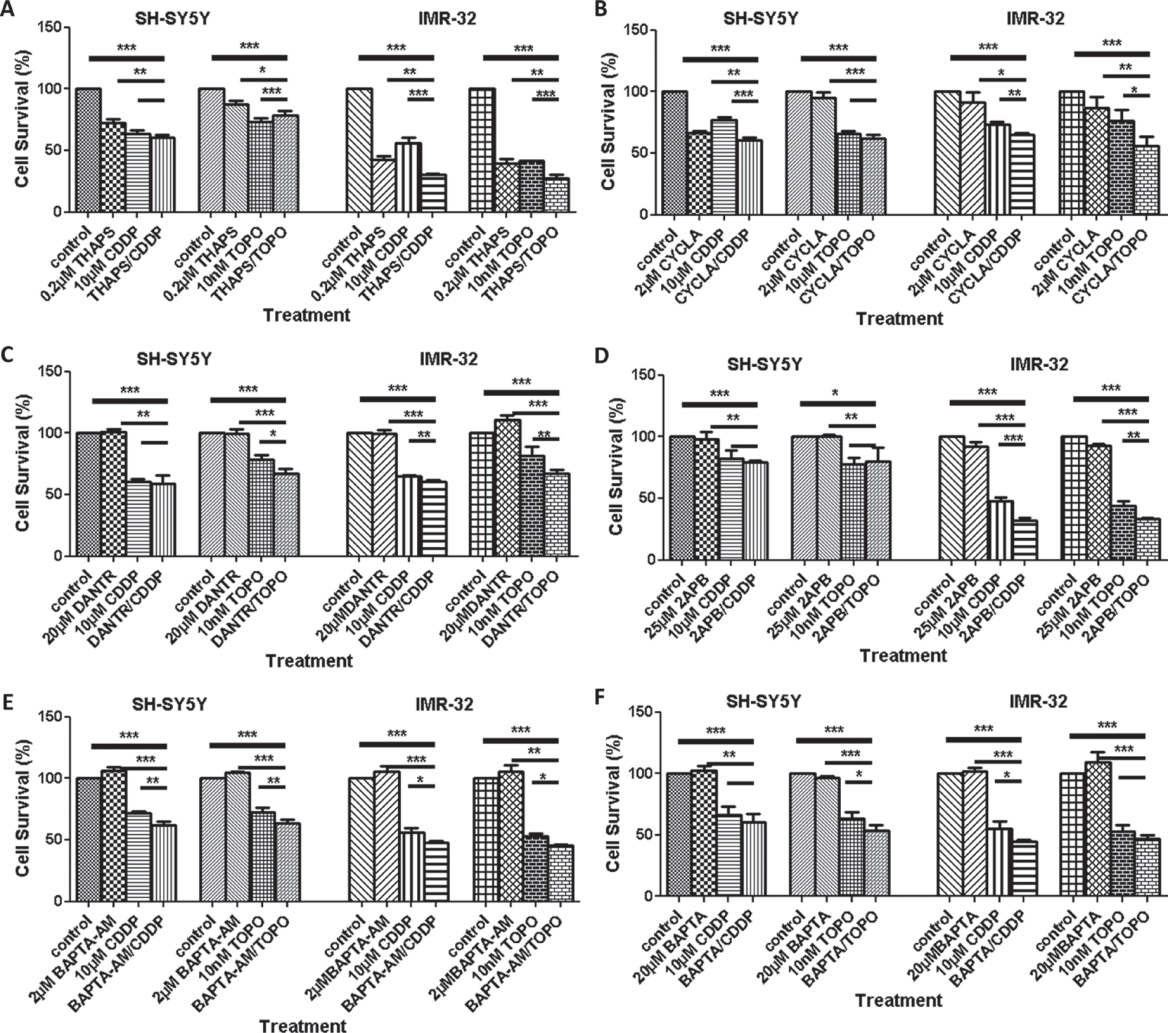
Viability of neuroblastoma cells treated with different types of calcium modulators alone or in combination with CDDP or TOPO Sensitivity to calcium modulators alone or in combination with CDDP or TOPO was assessed in SH-SY5Y and IMR-32 human neuroblastoma cells using the trypan blue exclusion test after 72 h of drug exposure. The following pharmacological calcium modulators were applied: (**A**) thapsigargin (THAPS), (**B**) cyclosporine (CYCLA), (**C**) datrolene (DANTR), (**D**) 2-APB, (**E**) BAPTA-AM and (**F**) BAPTA. Data are based on three independent biological experiments and represent mean cell viability (%) relative to control cells ± standard deviation. Statistical significance is relative to the untreated control and considered if *p < 0.05* (*), *p < 0.01* (**), *p < 0.001* (***) and were assessed via a One-Way ANOVA with Dunnett's Test for multiple comparisons.

To assess a synergistic potential of pharmacologically targeting selected proteins involved in [Ca^2+^]_i_ regulation alongside conventional chemotherapy, we analyzed the viability of neuroblastoma cells following application of calcium signaling modulators alone or in combination with either CDDP or TOPO. Thapsigargin (THAPS), a sarco/endoplasmic reticulum Ca^2+^ ATPase (SERCA) pump inhibitor, evoked the largest cytotoxic effect of all investigated calcium signaling modulators when applied as single agent at a concentration of 0.2μM. This effect was more pronounced in IMR-32 compared to SH-SY5Y cells (Figure 6A). Co-administration with either CDDP or TOPO significantly enhanced cytotoxicity in IMR-32 cells (but not SH-SY5Y cells) compared to single application of either drug (Figure 6A).

Treatment of SH-SY5Y with cyclosporine A (CYCLA) also had a significant cytotoxic effect. Furthermore, when CYCLA was combined with CDDP and applied to SH-SY5Y an enhanced cytotoxic effect was observed (*p* < 0.001) that was reproducible in IMR-32 cells for combinatory treatment with CYCLA and CDDP but also for CYCLA and TOPO (Figure 6B).

A similar result was found for dantrolene (DANTR), a ryanodine receptor antagonist (Figure 6C). 2-APB, an IP3 receptor antagonist, also was effective in reducing cell survival in combination with either CDDP or TOPO in IMR-32 cells but not in SH-SY5Y cells (Figure 6D). In addition, the membrane-permeable calcium chelator BAPTA-AM (2 μM) was able to enhance cytotoxicity when given in combination with CDDP or TOPO in SH-SY5Y and IMR-32 cells (Figure 6E). Results of further experiments using additional calcium signaling modulators and an additional neuroblastoma cell line (NLF cells) are provided as supplementary information (Supplementary Figures 5–8).

## DISCUSSION

Neuroblastoma (NB), the most common extracranial solid tumor in children, is stratified into five stages (1, 2, 3, 4 and 4S), two of which (3 and 4) identify a highly aggressive disease [15]. High-risk NB have been previously associated with distinct genomic alterations including mutations or rearrangements of *ALK*, *ATRX*, and *TERT*, as well as amplification of *MYCN* [14]. These and other molecular aberrations may reduce susceptibility to chemotherapy-induced programmed cell death (drug resistance) and thus are key factors in the treatment failure of advanced NB patients [15]. Primary and acquired drug resistance may lead to survival of tumor cells following chemotherapy and thereby trigger tumor recurrence and metastasis formation in neuroblastoma [2]. Therefore, finding alternative strategies to promote apoptosis or other types of programmed cell death pathways in response to treatment is relevant for the clinical management of aggressive forms of NB [15]. Since calcium signaling is a key factor in triggering cellular processes (e.g. differentiation, apoptosis, proliferation) [7, 16], we focused on its role in modulating response to CDDP and TOPO treatment of NB cells *in vitro*.

CDDP and TOPO are part of the pharmacological regimen for treatment of advanced neuroblastoma. CDDP is a chemotherapeutic drug used for several human malignancies and cytotoxicity is primarily mediated by its ability to cause DNA damage and subsequent apoptotic cell death [17–18, 6]. Other reports suggested that CDDP may directly interact with mitochondria to induce apoptosis, and this may account for a significant part of its clinical activity [18]. In contrast, the cytotoxicity of TOPO, a topoisomerase I inhibitor, is reportedly due to a ternary complex of topoisomerase I, DNA and TOPO that interferes with the moving replication fork and eventually leads to a replication arrest and the formation of cytotoxic double-stranded breaks in the DNA [19]. Despite evidence of a role for calcium signaling in the therapeutic success of these drugs, the underlying mechanisms have yet to be fully elucidated.

Data presented here demonstrate that relatively low concentrations of CDDP and TOPO can significantly reduce survival of NB cells in a time- and concentration-dependent manner. This effect is triggered mainly by an apoptotic pathway that activates effector caspases, though necrosis-mediated cell death is prominent following exposure to higher concentrations of CDDP or TOPO. Compared to CDDP, TOPO treatment resulted in more pronounced cell necrosis in our experiments. A role of [Ca^2+^]_i_ in both apoptotic and necrotic cell death has been reported [15]. In apoptosis, fluctuations in store-dependent Ca^2+^ release mediated by IP3 receptors can induce prolonged endoplasmic reticulum (ER) stress and activation of pro-caspase-12, a precursor of effector caspases [20]. In necrosis, intracellular Ca^2+^ overload may trigger the activation of Ca^2+^-dependent proteases and a subsequent loss of cell membrane integrity [21]. Importantly, each neuroblastoma cell line exposed to CDDP or TOPO showed differences in intrinsic drug sensitivity, a finding in line with the observation of heterogeneity in neuroblastoma treatment response in patient studies [22].

Induction of apoptosis by CDDP has been shown to involve both caspase-dependent and caspase-independent apoptotic pathways [18] and has been linked to increased [Ca^2+^]_i_ [16]; [5]. Our microarray-based gene expression profiling data demonstrated that CASP8 and CASP3 mRNA expression increased upon exposure of SH-SY5Y neuroblastoma cells to CDDP. We also investigated changes in [Ca^2+^]_i_ upon treatment of neuroblastoma cells with CDDP or TOPO. In line with previous reports, we found a time- and concentration-dependent increase of [Ca^2+^]_i_ with either drug. Splettstoesser et al. [5] reported that CDDP increases [Ca^2+^]_i_ time- and concentration-dependently in HeLa-S3 but not in U2-OS cells. The increase of [Ca^2+^]_i_was related to the activation of calpain, rather than caspase-8, triggering apoptosis (See Supplementary Files) [5]. Data from our cytotoxicity and [Ca^2+^]_i_ assay studies suggest that CDDP- or TOPO-induced cell death of SH-SY5Y and IMR-32 cells may also be caspase-independent at higher drug concentrations.

Although we were unable to directly assess the contribution of intracellular Ca^2+^ release following CDDP induction without promoting a stress-response via the omission of an external Ca^2+^ source (Supplementary Figure 10), our previous investigations showed that IP3 receptor-regulated Ca^2+^-release governs CDDP-mediated apoptosis in cancer cells [5]. Whilst increases in [Ca^2+^]_i_ following exposure to CDDP or topotecan are both time- and concentration-sensitive in either neuroblastoma cell line, it was evident that the relationship is more linear in SH-SY5Y than in IMR-32 cells. We can speculate that the non-linear response exhibited in IMR-32 cells might be indicative of differential sources of calcium entry or release. We demonstrate that IMR-32 cells display higher sensitivity to the calcium antagonists thapsigargin, 2-APB and dantrolene when compared with SH-SY5Ys in cell survival assays. This might be suggestive of preferential intracellular calcium release in IMR-32 cells.

Here, we focused on changes in the expression and function of selected proteins involved in calcium homeostasis following chemotherapy and explored whether combinatorial treatment of neuroblastoma cells with calcium modulators and CDDP or TOPO results in synergistic effects as indicated by increased cytotoxicity. In addition, we investigated whether chemotherapy alters mRNA and protein expression of key regulators involved in [Ca^2+^]_i_ homeostasis including the calcium-binding protein S100A6 (calcyclin), the inositol trisphosphate receptors 1 and 3 (*ITPR1* and *ITPR3*), as well as the ryanodine receptors 1 and 3 (*RYR1* and *RYR3*). *COX2* expression was investigated to check for mitochondrial stress [23] known to occur upon exposure to anticancer drugs. The selected genes were found to be differentially expressed in the neuroblastoma cell lines following treatment. Most prominently, *ITPR3* and *RYR1* were consistently up-regulated at the mRNA and protein level following CDDP exposure in SH-SY5Y cells, suggesting a key role for the governance of intracellular calcium dynamics preceding chemotherapy-induced cell death, and this was paralleled by an up-regulation of *COX2* mRNA levels. However, it is important to note that basal expression of RYR1 was initially very low in SH-SY5Y cells and FACS analysis may be less sensitive in detecting changes in its expression. In this instance, it resulted in either no change in expression following exposure to TOPO, or negative expression for CDDP. In all neuroblastoma cell lines tested, expression of S100A6 was significantly up-regulated following exposure to either drug. The S100 gene family shares a common structure containing a Ca^2+^-binding EF-hand motif mediating the formation of protein-protein interactions with effector molecules. S100 proteins play key roles in various processes including differentiation, cytoskeleton dynamics, enzyme activity, Ca^2+^ homeostasis, cell growth as well as cell survival and apoptosis [24]. S100A6 expression has been associated with protection from TNF-α-induced apoptosis in cardiomyocytes [25], decreased metastasis rate in cancer of the bone [26], as well as the regulation of cell cycle progression [27]. Furthermore, S100A6 induces epithelial-mesenchymal transition and promotes cell migration and invasion in a β-catenin-dependent manner [28]. We speculate that the increased expression of S100A6 following exposure to anticancer drugs demonstrated in our study may indicate a protective role for this protein in neuroblastoma cells and thus suggest this protein as a potential therapeutic target for combined chemotherapy strategies. Indeed, previous studies proposed S100A6 as potential therapeutic target in pancreatic cancer [28] and gastric cancer [29].

Treatment of SH-SY5Y cells for 72h with CDDP deregulated the mRNA expression of several genes whose gene products are involved in calcium signaling as well as p53 signaling, cell cycle, apoptosis, histone-related epigenetic changes, metabolism and response to a toxic substance. These signaling pathways include many components that might be activated upon calcium increase [42] (e.g. cell cycle, p53), might be triggered by a calcium overload (e.g. apoptosis), or might take a role in buffering of the [Ca^2+^]_i_ overloads. Thus, [Ca^2+^]_i_ changes observed upon exposure of neuroblastoma cells to CDDP might be able to disturb important signaling pathways that in turn might change global mRNA expression. In addition, previous work showed that CDDP also influences the expression of miRNAs, such as hsa-miR-204 [30], hsa-miR-21 [31] and hsa-miR-16 [32].

As we observed treatment-related changes in [Ca^2+^]_i_ and in expression of several genes encoding modulators of calcium signaling, we also investigated whether treatment of neuroblastoma cells with pharmacological modulators of calcium-regulatory proteins may increase cytotoxic effects of CDDP and TOPO in neuroblastoma cell lines. Among the different pharmacological calcium modulators investigated, thapsigargin showed the highest cytotoxic effects. This compound is currently being assessed as a potential anticancer drug [30, 33]. It is a strong inhibitor of sarco-endoplasmic reticulum Ca^2+^-ATP-ases (SERCA) and triggers store-operated Ca^2+^-entry [30, 31]. Upon depletion of the ER Ca^2+^ stores, thapsigargin triggers the opening of plasma membrane Ca^2+^ channels and an ER stress response [33–35]. This is followed by activation of apoptotic pathways within the ER and the mitochondria. A sustained accumulation of Ca^2+^ in the mitochondrial matrix, induced by ER stress, might trigger the proapoptotic mitochondrial changes such as the permeability transition, dissipation of the electrochemical potential, matrix swelling, re-localization of Bax to mitochondria and the release of cytochrome c and other apoptosis-inducing factors [36]. Thereby, thapsigargin induces apoptosis, which could further enhance the apoptotic effects caused by conventional chemotherapeutics like CDDP or TOPO. Recently, analogues of thapsigargin have been developed for the treatment of prostate cancer and hepatocellular carcinoma [33]. Structure-activity relationships enabled design of equipotent analogues containing a linker coupled with peptides that are substrates for either prostate specific antigen (PSA) or prostate specific membrane antigen (PSMA), which enables specific targeting of the prodrug to prostate cancer cells and hepatocellular carcinoma cells. Results from a first phase I trial using a thapsigargin-based PSMA-activated prodrug in advanced solid cancers have been recently published [33, 37].

The second most toxic inhibitor of calcium-regulatory proteins in neuroblastoma cells was cyclosporine A, a drug that is currently clinically used as an immunosuppressive agent [38, 39]. It targets calcineurin, resulting in a complete block of the translocation of the cytosolic component of the nuclear factor of activated T cells (NF-AT), consequently, resulting in a failure to activate genes regulated by the NF-AT transcription factor [40, 38]. In addition, cyclosporine A has been shown to induce mitochondrial permeability transition pore opening followed by reduction of delta psi m and caspase activation, thereby leading to apoptosis [41]. Therefore, cyclosporine A may lead to increased cytotoxicity when given in combination with CDDP or TOPO [40, 41].

A third calcium-regulatory compound with cytotoxicity in SH-SY5Y cells was 2-APB that blocks the function of IP3R [42]. Thus, long-time exposure to this compound might induce increased ER stress which in turn can induce cytotoxicity in neuroblastoma cells and increase the cytotoxic efficacy of CDDP or TOPO. Importantly, 2-APB has antagonistic effects on Ca^2+^-entry rather than Ca^2+^-release and modulates the store-operated Ca^2+^-entry [42]. Thus, our *in vitro* findings suggest that the three compounds discussed above or clinically tolerable derivatives of them may bear potential as anticancer drugs by fostering anti-cancer efficacy CDDP or TOPO. In addition, application of the RYR antagonist DANTR enhanced CDDP and TOPO cytotoxicity in IMR-32 cells, and CDDP toxicity in SH-SH5Y cells is likely due to increased ER stress as a result of long-time inhibition of RYRs that are normally required for the maintenance of cell function.

Finally, we investigated whether interfering with intra- or extra-cellular calcium can modulate the effectiveness of CDDP or TOPO treatment in neuroblastoma cells. Chelation of Ca^2+^ using membrane permeant BAPTA-AM enhanced cytotoxicity of both CDDP and TOPO indicating an induced stress response following a reduction in free intracellular calcium ions (Figure 6). Importantly, this is in agreement with reports of an upregulation in ER stress marker XBP1 in neuronal cell cultures following exposure to BAPTA-AM [43]. Furthermore, a partial chelation of extracellular calcium enhanced the effect in CDDP treatment in IMR-32 and NLF cells which cannot be explained in detail at this point, since more experiments will be required to elucidate the mechanisms of enhanced cytotoxicity due to regulation of [Ca^2+^]_i_.

In conclusion, the treatment of cultured neuroblastoma cells with various concentrations of either CDDP or TOPO significantly decreased cell viability, increased apoptosis and increased [Ca^2+^]_i_ in a time- and concentration-dependent manner. Thus, deregulation of [Ca^2+^]_i_ may contribute to the toxic effects of these chemotherapeutic agents. Moreover, expression of crucial regulators of calcium signaling, such as IP3R3, RYR3 and S100A6, are deregulated upon CDDP or TOPO treatment of neuroblastoma cells. Importantly, pharmacological modulation of the [Ca^2+^]_i_ response increased the cytotoxic effects of CDDP and TOPO *in vitro*, suggesting a potential role for combinatorial therapy in advanced neuroblastoma.

## MATERIALS AND METHODS

### Chemicals

The following chemicals of highest available purity were purchased from Tocris Biosciences, Bristol, Great Britain or R&D, Germany: cisplatin (Cat. No. 2251), topotecan hydrochloride (Cat. No. 4562), thapsigargin (Cat. No. 1138), 2-aminoethoxydiphenylborane (2-APB, Cat. No. 1224), cylopiazonic acid (Cat. No. 0507), dantrolene (Cat. No. 0507), ryanodine (No. 1329), verapamil (Cat. No. 0654), nifedipine (Cat. No. 1075), 1,2-Bis(2-aminophenoxy) ethane-N,N,N’,N’-tetraacetic acid (BAPTA, Cat. No. 2786), BAPTA-AM (Cat. No. 2787), and YM58483 (Cat. No. 3949).

### Cell culture and treatment schemes

The SH-SY5Y (ATCC^®^ CRL2266^™^), IMR-32 (ATCC^®^ CCL127^™^) neuroblastoma cell lines were obtained from American Type Culture Collection (ATCC, Manassas, Virginia). The NLF cell line was obtained from the CHOP cell line bank (Philadelphia, PA, USA). Neuroblastoma cells were maintained in culture as recommended by the provider. SH-SY5Y cells were grown in DMEM/F12 medium supplemented with 10% (v/v) heat inactivated fetal bovine serum (FBS), penicillin (0.1lU) and streptomycin (100 μg/mL); IMR-32 cells were grown in DMEM medium supplemented with 10% FBS, penicillin (0.1lU) and streptomycin (100 μg/mL); NLF cells were grown in RPMI medium supplemented with 10% FBS, penicillin (0.1lU) and streptomycin (100 μg/mL). All neuroblastoma cells were cultured under sterile conditions and were grown in a humidified incubator at 37°C with 95% O_2_ and 5% CO_2_.

### Trypan blue cytotoxicity test (TBCT)

After exposure (12 h–72 h) to low concentrations of anticancer drugs (CDDP or TOPO at 0.1 nM–10 μM) or calcium signaling modulators, the cells were trypsinized and collected in fresh culture medium, centrifuged at 2000 rpm for 5 minutes, resuspended in 500 μl complete culture medium and then analyzed using the TBCT test with a VICell XR cell analyzer (Beckmann Coulter, Germany) as recommended by the manufacturer.

### Apoptosis and cytotoxicity tests via fluorescence activated cell sorting (FACS)

Total cytotoxicity and apoptosis were assessed using a kit with two fluorescent dyes, namely SR-FLICA (Fluorescent Labelled Inhibitors of Caspases) that covalently binds active caspase -3 and -7 enzymes, and 7-AAD that stains permeable necrotic cells (ImmunoChemistry Technologies, Bloomington, MN). Four types of cell population were quantified: 1) early apoptotic, 2) late apoptotic, 3) vital and 4) necrotic cell population. Cells were incubated with various concentrations of CDDP or TOPO for 72h. After drug incubation the cells were harvested and stained with SR-FLICA, followed by 7-AAD. After washing with PBS, fluorescence was acquired using the BD LSR Fortessa (BD Medical Technology) with 488nM laser for excitation and emission with two filters 585/15 and 610/20 for SR-FLICA and 7-AAD, respectively. Data were processed using FACSDiva 6.3. 50,000 events were recorded for each sample. Cells were sorted in four different types of cell population, indicated by four quadrants in the representative scatter plots.

### Live calcium imaging

Cells were plated in 35mm `easy grip’ BD Falcon tissue culture plates (Becton-Dickinson, USA). The plates were washed with Tyrode`s buffer and loaded with Fluo-4AM (2 μM final concentration) (Molecular Probes, USA), a calcium sensitive dye, for 45 min at 37°C. Serial fluorescent images were taken with an Olympus Microscope BX51 Wi with Xenon Arc Burner and “Xcellence rt” software. An excitation wavelength of 494 nm and an emission wavelength of 516 nm were used. Images were taken every minute for up to 3.5 hrs. Cells were pre-defined via selected regions of interest (ROI) and the analysis was carried out offline. Results are expressed as percentage change in fluorescence intensity (%) before and after drug application.

### Protein quantification via FACS

For the quantification of protein expression, we utilized a flow cytometry-based technique that incorporates the use of monoclonal primary antibodies and fluorescently conjugated secondary antibodies for protein detection. This technique has been validated for intracellular and cell surface proteins and is relatively high-throughput [42, 44]. Moreover, it has comparative sensitivity and specificity to other methods of protein quantification [45]. Monoclonal primary antibodies; IP3R1 (Ms, Santa Cruz), IP3R3 (Rb, Santa Cruz), RYR1 (Rb, Millipore), RYR3 (Rb, Millipore) and S100A6 (Rb, Ab Cam) detected protein expression in SH-SY5Y and IMR-32 cells fluorescently stained with alexa-488 conjugated secondary Ab (Life Technologies). This was analyzed by using the BD LSR Fortessa (BD Medical Technology). Fluorescence signals were acquired using a 488 Laser (530/30) FITC filter and 640 laser with APC 660/40. To exclude duplets, aggregates and debris, FSC/SSC gating was applied to an unstained control sample and single stained control samples were used to adjust the voltage of the photomultiplier tubes (PMTs) to generate a signal free from background fluorescence. Finally, fluorescence intensity was measured with respect to the unstained and isotype control and the data were analyzed using BD FACS Diva 6.3.

### Immunofluorescence imaging via confocal laser scanning microscopy

SH-SY5Y cells were plated in 12-well plates before treatment with CDDP (1 μM) or TOPO (0.01 μM) for 72 h. Then, cells were fixed in 4% paraformaldehyde (PFA) at 4°C and permeabilized using ice-cold 100% methanol. Non-specific antibody-binding was reduced by blocking with 5% horse serum in PBS for 1 hour at room temperature. IP3R1, IP3R3, RYR1, RYR3 or S100A6 primary antibodies (as described for flow cytometry) in 5% horse serum at dilutions of 1 in 250 were used and a secondary alexa-488 conjugated antibody was applied. Coverslips were mounted using Prolong Gold anti-fade reagent with, and without, DAPI (Thermo Fisher Scientific). Images were acquired using a Carl Zeiss LSM-710 inverted confocal microscope (X63) with Zeiss Zen software. Fluorescence intensity was analyzed using ImageJ software and is expressed as ‘corrected total cell fluorescence’ (CTCF).

### Preparation of nucleic acids, cDNA synthesis and RT-PCR

For extraction of RNA and DNA we used the AllPrep DNA/RNA Mini Kit (Qiagen, Hilden, Germany) following the provider's protocol. Extracted RNA was kept at −80°C and was used for cDNA synthesis using the Superscript II Kit (Invitrogen, Carlsbad, California, USA). For mRNA analysis, specific primers were designed using the Primer3 software and oligonucleotides were purchased from Eurofins MWG (München, Germany). Prior to use, the primers were tested using Human Universal Reference RNA (Stratagene, San Diego, California, USA), which was used as interplate calibrator for the RT-PCR analyses. As reference, expression levels of 18s and ARF1 were initially tested and ARF1 transcript levels were used for the experiments presented in this manuscript using the 2(−DeltaDeltaCt) method. The following primers were used: ITPR1 F: TTCCATCCTAACGGAACGAG; ITPR1 R: CACTCTGTTGCCAAAGCAAG; ITPR3 F: ACTGC CTCTTCAAGGTGTGC; ITPR3 R: CCCATGCACCTTC TTGTTCT; RYR1 F: GTCATCCTGTTGGCCATCATC; RYR1 R: GGTCTCGGAGCTCACCAAAAG; RYR3 F: GGCCACAGGACCCTGTTAT; RYR3 R: CTGTGGCATG TTCCCGTAG; S100A6 F: GAAGGAGCTGAAGGAGCT GA S100A6 R: CCCTTGAGGGCTTCATTGTA 18S F: CG GCTACCACATCCAAGGAA; 18S R: GCTGGAATTAC CGCGGCT; ARF1 F: GACCACGATCCTCTACAAGC; ARF1 R: TCCCACACAGTGAAGCTGATG.

### Microarray analysis

Three samples of parental SH-SY5Y cells and four samples of SH-SY5Y cells treated for 72 h with 10 μM CDDP were subjected to microarray-based gene expression analyses. RNA quality was determined using the Agilent Bioanalyzer and the Eukaryote Total RNA Nano Chip (Agilent Technologies, Hayward, CA, USA). The RNA integrity number (RIN) was measured as 9.9–10. The Affymetrix HuGene 2.0 ST and WT plus reagent kit (Affymetrix, Santa Clara, California, United States) was used following the provider's recommendation with a start material of 100 ng of total RNA. Affymetrix chip hybridizations were performed at the Center for Biological and Medical Research (BMFZ) at Heinrich Heine University Düsseldorf using a GeneChip Hybridization Oven 645 and a GeneChip^®^ Scanner 3000 7G. Bioinformatic evaluation of the microarray data was done with the R framework for statistical computing. The dataset was normalized and log2-transformed using the robust multi-array average (RMA) method implemented in the affy package. Differential expression of mRNA was assessed by computing the moderated t-statistics using the LIMMA package. The resulting *p*-values were corrected for multiple testing by controlling the false discovery rate. A mRNA was considered as being differentially expressed when the corrected *p*-value was below 0.05. For mapping of gene ontology terms and KEGG pathway terms the web interface RAMONA was used for the genes that showed a significant fold change (FC) in expression of < 0.5 or > 0.5 (Sass et al., 2015). The microarray data were submitted to the Gene Expression Omnibus repository (GEO); datasets are found under the GEO submission number GSE86842.

### Statistics

Results are shown as means ± standard deviation (SD). One-way ANOVA tests with Dunnett's test for multiple comparisons and Student’ *t*-test were used to compare statistical differences between controls and each of the treatment groups and a two-way ANOVA test was used to interpret the statistical relationship between controls and treatment groups. *P* values of < 0.05 (*), < 0.01 (**) and < 0.001 (***) were considered as statistically significant. Experiments were repeated on at least three independent occasions (*n* = 3–5) and analyzed using Microsoft Excel and GraphPad Prism 6 software.

## SUPPLEMENTARY FIGURES












