# Supplementary material for: Calcium-regulatory proteins as modulators of chemotherapy in human neuroblastoma
Source: Oncotarget. 2017 Feb 11;8(14):22876–93. doi: 10.18632/oncotarget.15283 (PMC5410270; doi:10.18632/oncotarget.15283)
Supplement: Supplementary file 1 [file oncotarget-08-22876-s001.pdf]

Calcium-regulatory proteins as modulators of chemotherapy in human neuroblastoma

Supplementary Materials

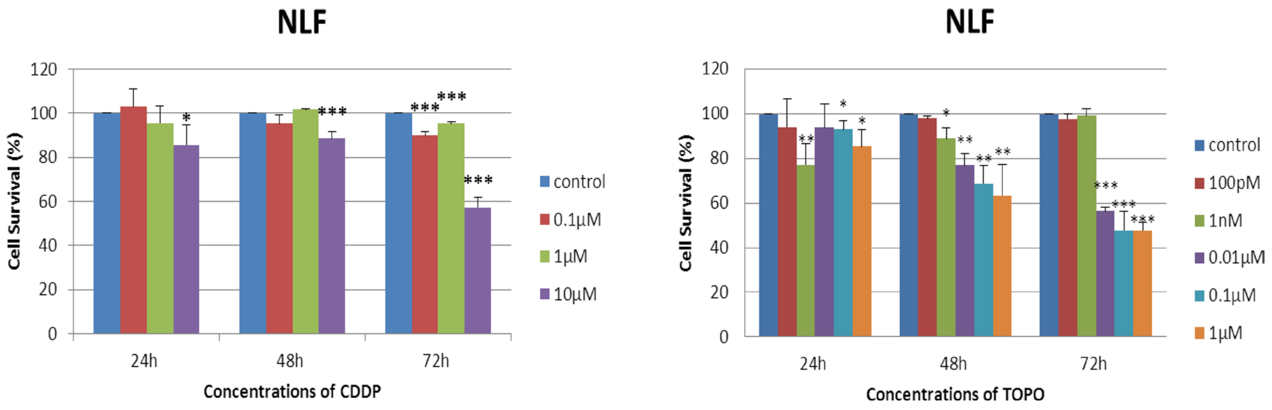

Supplementary Figure 1: Cell survival and apoptosis in NLF cells following chemotherapeutic treatment. (A) Sensitivity to chemotherapy via trypan blue exclusion test following exposure to 0.1 µM-10 µM CDDP and 0.1 nM-1 µM TOPO for 24, 48 and 72 h. Statistical significance is relative to untreated v's treated conditions and is considered if $p < 0.05$ (*), $p < 0.01$ (**), $p < 0.001$ (***), assessed via Student's *T*-test. NLF cells were grown RPMI medium supplemented with 10% heat inactivated fetal bovine serum (FBS), and antibiotics (penicillin and streptomycin). NLF neuroblastoma cells were the most insensitive to CDDP showing significant decrease of cell viability after CDDP exposure of 48 h with 10 µM CDDP, and after 72 h exposure of NLF cells to 1 and 10 µM CDDP.

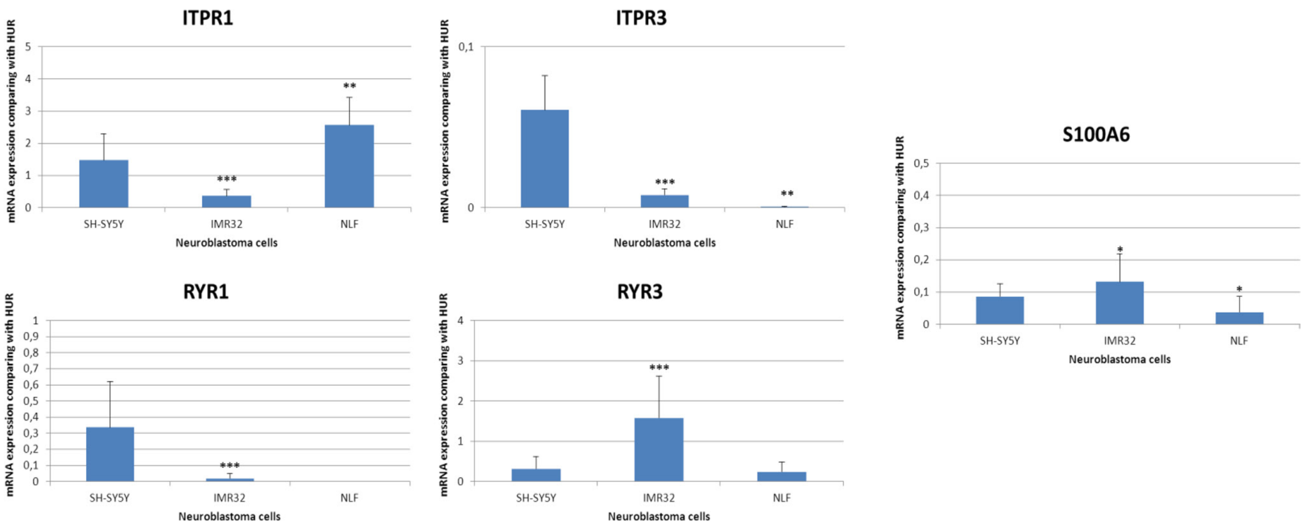

Supplementary Figure 2: mRNA expression of $[Ca^{2+}]_i$ signaling related genes: S100A6, IP3R1, IP3R3, RYR1 and RYR3 differs between neuroblastoma cell lines. We investigated whether the treatment of neuroblastoma cells with CDDP and TOPO induces changes in the gene expression profile of seven calcium signaling related genes that have been associated with the pathology of neuroblastoma. This includes: (i) the S100A6 gene that encodes the S100A6 protein; Inositol trisphosphate receptor (IP3R) 1 and 3 encoding genes (iv) ITPR1 and (v) ITPR3; but also Ryanodine receptor (RYR) 1 and 3 encoding genes (vi) RYR1 and (vii) RYR3. COX2 gene expression was added to check mitochondrial stress that occurs upon exposure of neuroblastoma cells to anticancer drugs.

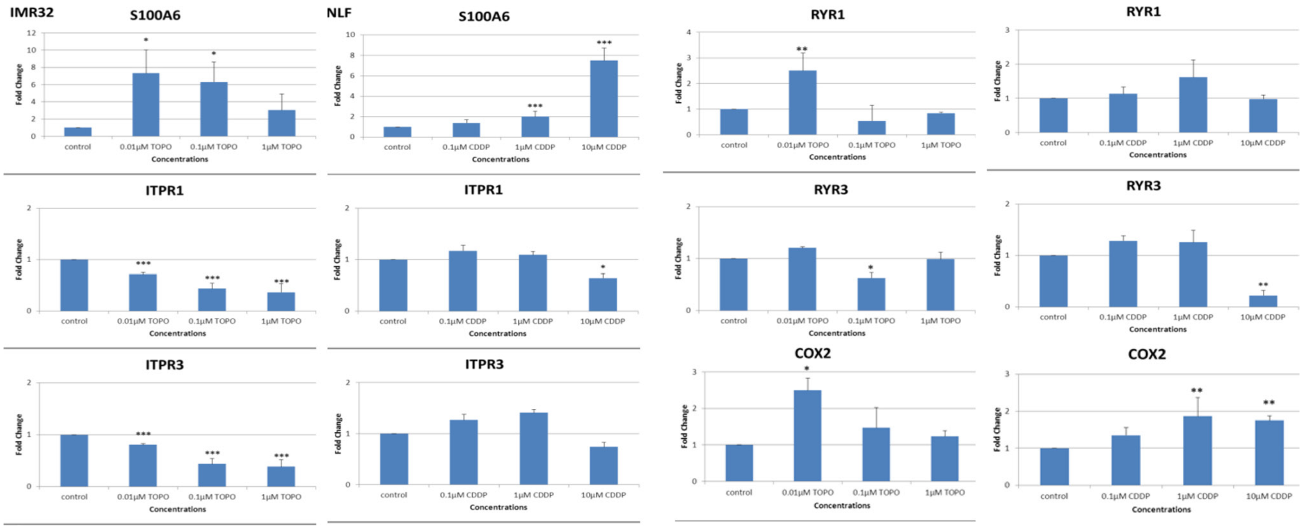

Supplementary Figure 3: Testing IMR32 and NLF cells revealed that CDDP and TOPO treatment triggers a deregulation of mRNA expression thus confirming the findings with SH-SY5Y cells. For 72 h CDDP treatment, the mRNA increase of S100A6 was 10-fold higher, compared to control conditions in all cell lines tested. Following TOPO treatment, this increase was the strongest in SH-SY5Y (10-fold) followed by IMR-32 and NLF cells.

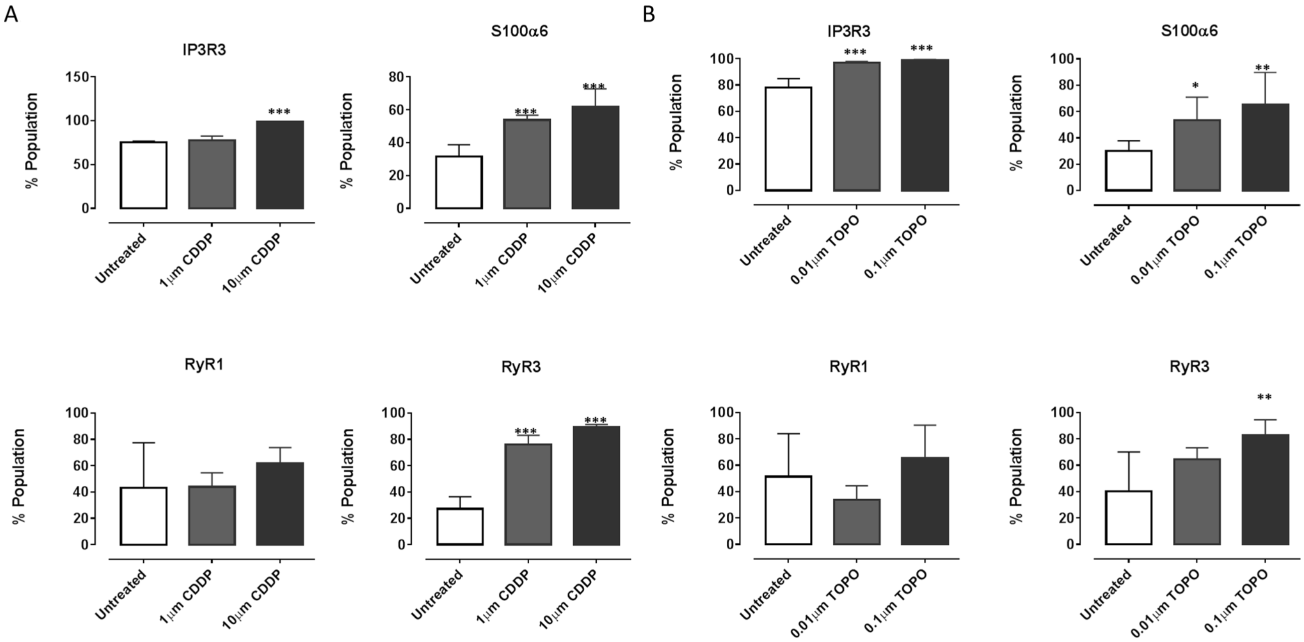

Supplementary Figure 4: Increased expression of calcium handling proteins IP3R3, RYR3 and is also evident in a second neuroblastoma cell line IMR-32, following exposure to CDDP and TOPO. IMR-32 cells treated with either (**A**) CDDP (1 µM or 10 µM) or (**B**) TOPO (0.01 µM or 0.1 µM) for 72 hours were harvested, fixed, permeabilized and incubated with primary antibodies specific for IP3R3, RyR1, RyR3 or S100α6 followed by incubation with a fluorescently conjugated (Alexa-488 nm) secondary antibody. Experiments were carried out on at least three independent occasions and statistical significance is relative to untreated cells and considered if $p < 0.05$ (*), $p < 0.01$ (**) or $p < 0.001$ (***), assessed via One-Way ANOVA with Dunnett's Test.

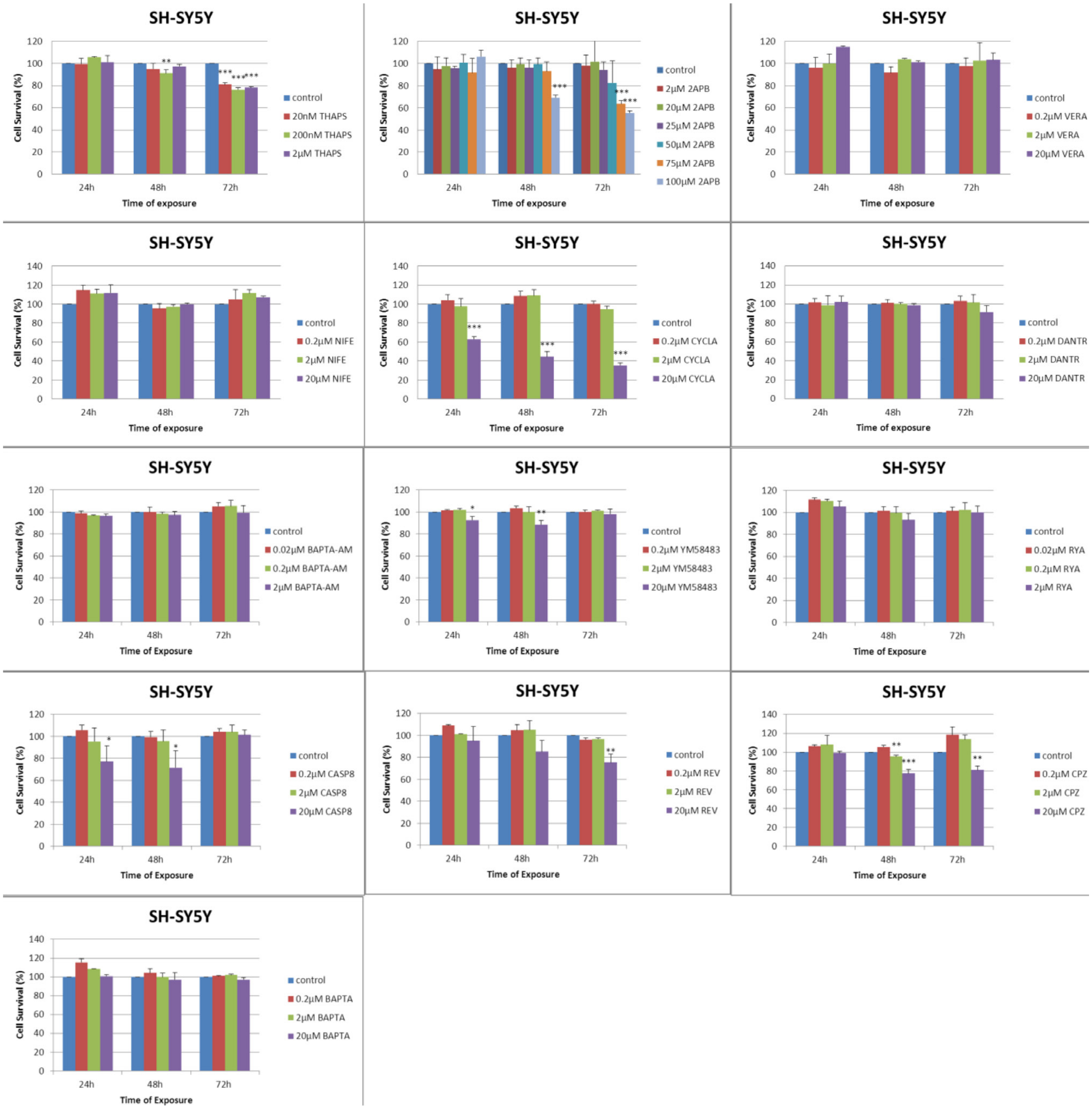

Supplementary Figure 5: Sensitivity to calcium modulator of SH-SY5Y human neuroblastoma cells, evaluated using the Trypan Blue Cytotoxicity test. Data show means with standard deviations while significance was tested with Student's *t*-test, where *$p < 0.05$, **$p < 0.01$, ***$p < 0.001$.

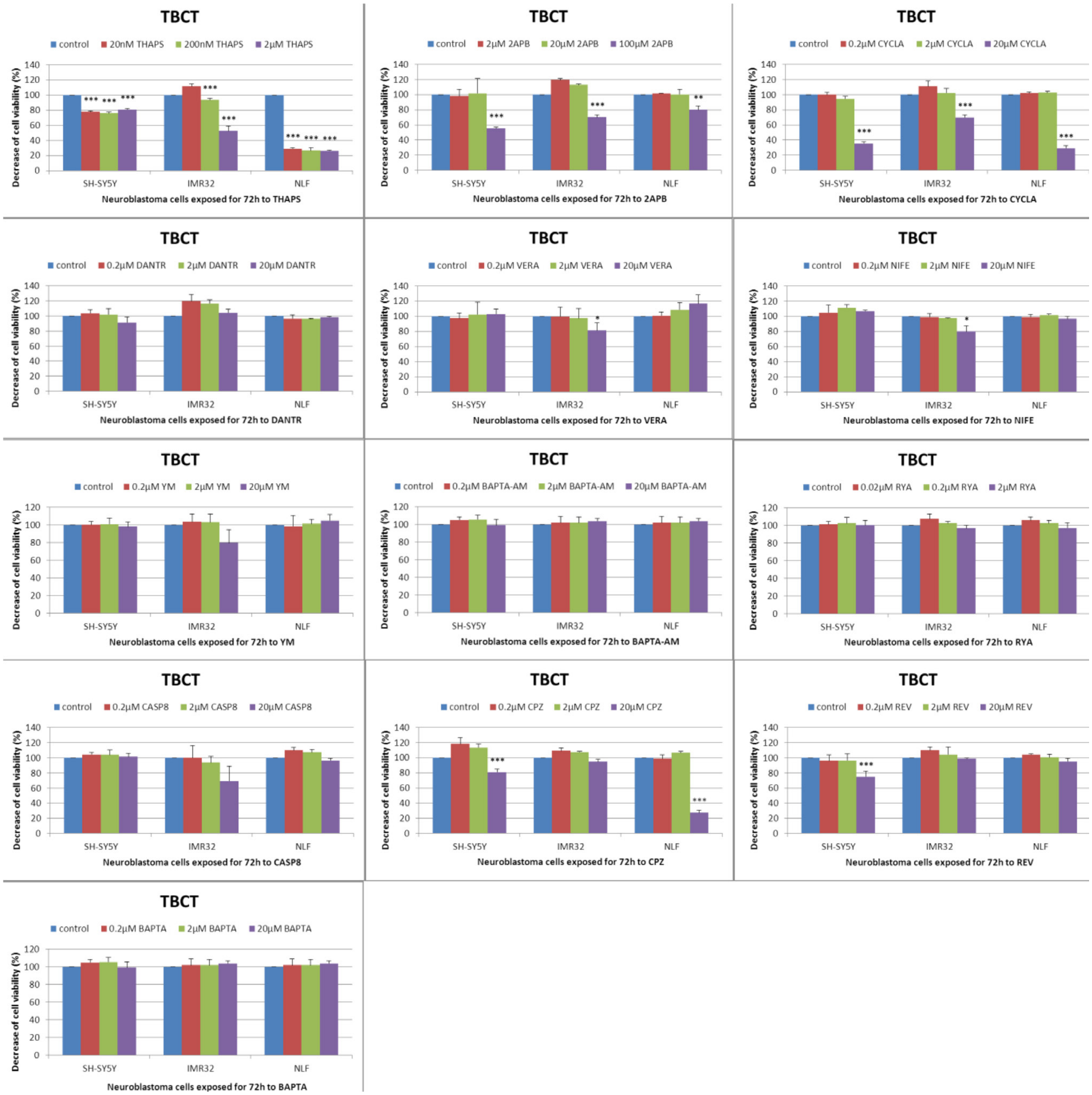

Supplementary Figure 6: Sensitivity to calcium modulators in human neuroblastoma cells, evaluated using the Trypan Blue Cytotoxicity test after 72 h of exposure. Data show means with standard deviations while significance was tested with Student's *t*-test, where $*p < 0.05$, $**p < 0.01$, $***p < 0.001$.

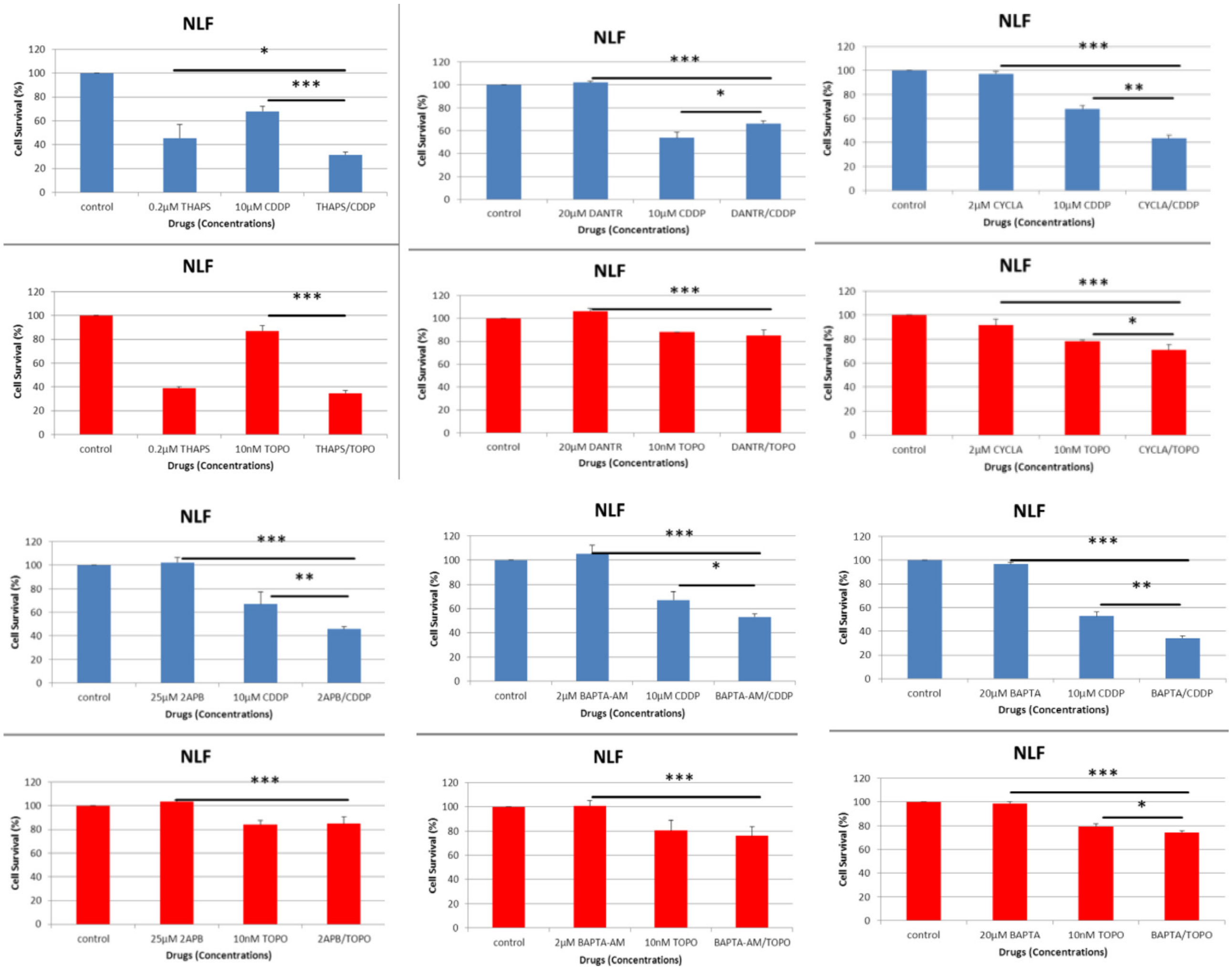

Supplementary Figure 7: Pharmacological modulation of key $[Ca^{2+}]_i$ regulators in combination with chemo-therapeutics can enhance cyto-toxicity in NLF cells. Sensitivity to calcium modulators alone, or in combination with CDDP or TOPO, evaluated using the trypan blue exclusion test after 72 h exposure. Calcium modulators; thapsigargin (THAPS), cyclosporine (CYCLA), C. datrolene (DANTR), 2-APB, BAPTA-AM and BAPTA. Data are expressed as cell viability (%), mean ± standard deviation (n = 3). Statistical significance is relative to untreated cells and considered if $p < 0.05$ (*), $p < 0.01$ (**) or $p < 0.001$ (***), assessed via Student's t-test.

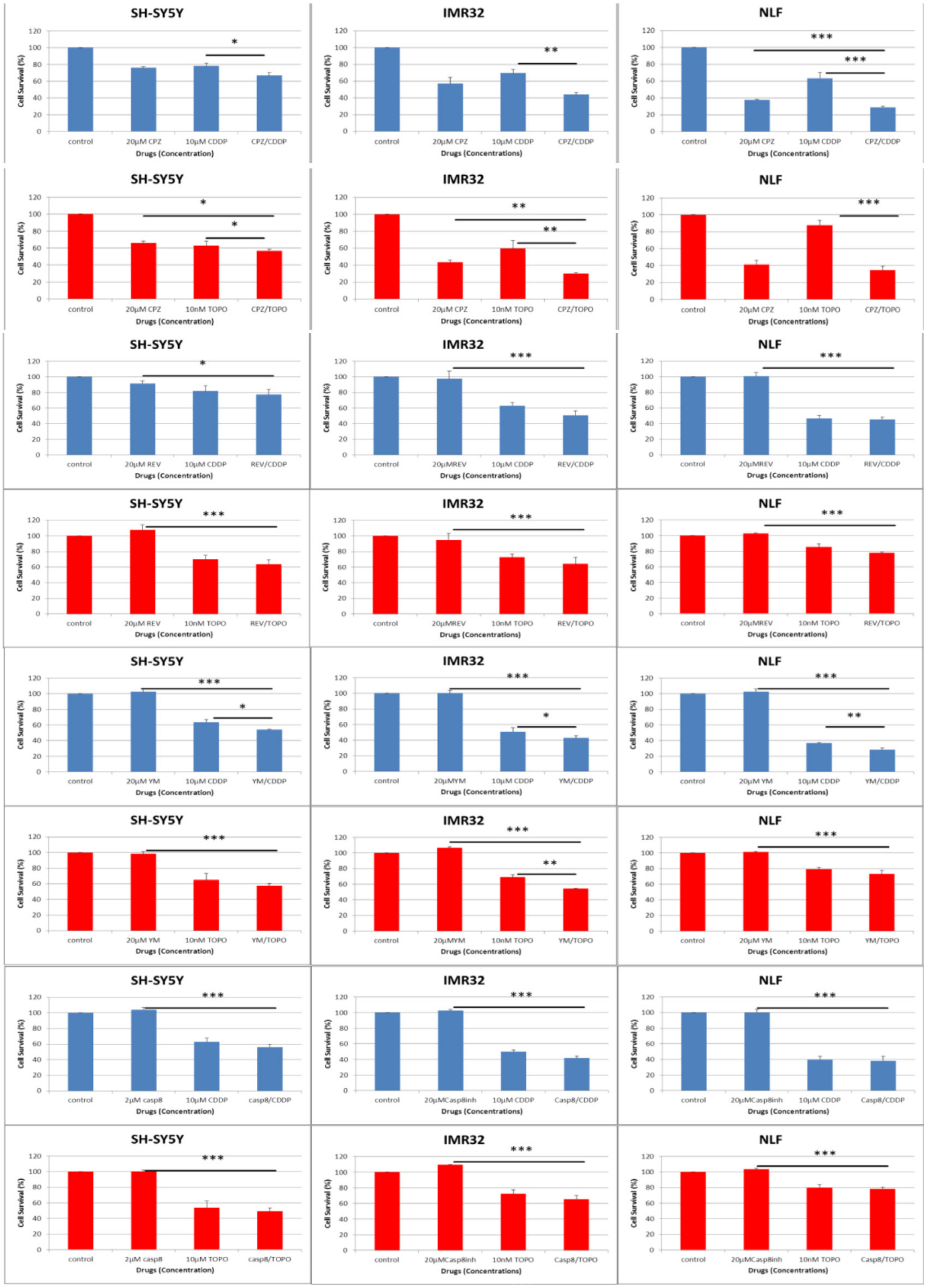

Supplementary Figure 8: The inhibitory effects MDR1 using reversan (REV), and SOCE using YM58483 were tested in all neuroblastoma cells in combination with CDDP or TOPO. The use of REV in combination with CDDP or TOPO did not show any effects. SOCE inhibition increased cytotoxicity in combination with CDDP. Finally we tested the role of casp8 inhibition in the survival of neuroblastoma cells. The application of casp8 inhibitor did not change the survival of neuroblastoma cells for any of the drug combination used. Data are expressed as cell viability (%), mean ± standard deviation ($n = 3$). Statistical significance is relative to untreated cells and considered if $p < 0.05$ (*), $p < 0.01$ (**) or $p < 0.001$ (***), assessed via Student's *t*-test.

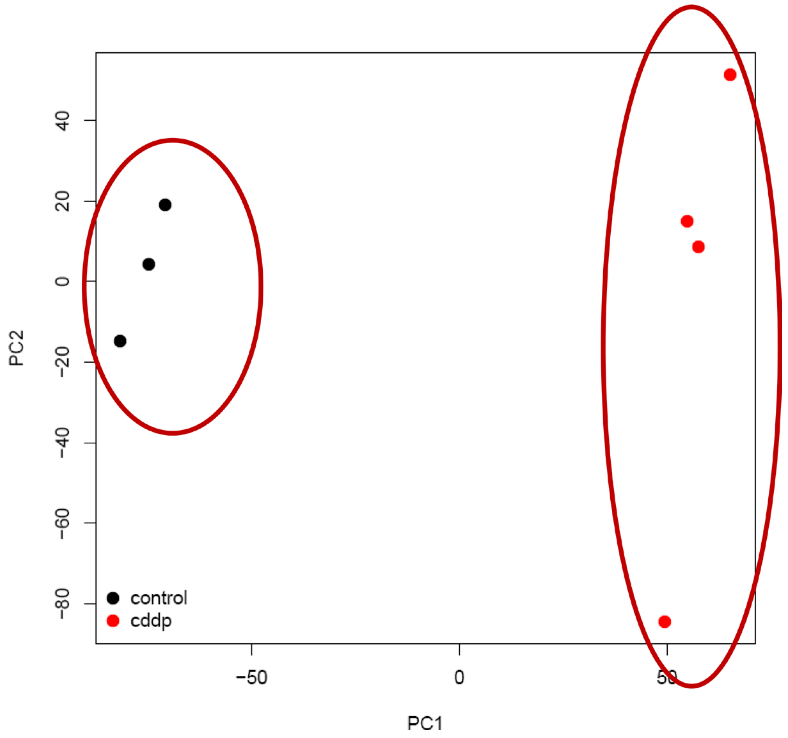

Supplementary Figure 9: Principal component analysis of the microarrays. Notice clear separation of the conditions used in the experiment.

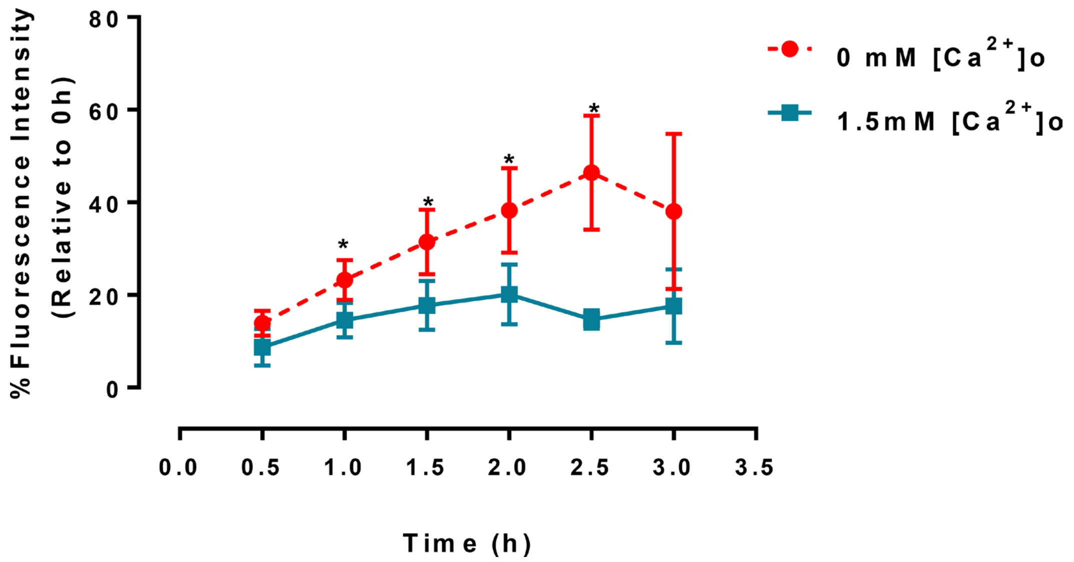

Supplementary Figure 10: Clamping extracellular calcium in SH-SY5Y cells creates a cellular stress response in prolonged measurement. Fluorescence intensity over a range of 0.5 h time points (0.5 h–3.0 h) in Fluo-4-AM loaded SH-SY5Y cells following exposure to 0 mM or 1.5 mM external calcium ([Ca2+]o) in Tyrode's Solution. Results are expressed as percentage intensity relative to untreated fluorescence levels at time point 0h (n = 3). Statistical significance is relative to fluorescence prior to drug infusion and is considered if $p < 0.05$ (*), $p < 0.01$ (**), $p < 0.001$ (***), assessed via multiple Student's T test.

Table Analyzed	Ca2+ CDDP SH-SY5Y				
Two-way ANOVA	Ordinary				
Alpha	0,05				
Source of Variation	% of total variation	P value	P value sumr	Significant?	
Interaction	11,86	0,5295	ns	No	
Row Factor	27,08	< 0.0001	****	Yes	
Column Factor	27,66	< 0.0001	****	Yes	
ANOVA table	SS	DF	MS	F (DFn, DFd)	P value
Interaction	8131	24	338,8	F (24, 61) = 0.9584	P = 0.5295
Row Factor	18569	4	4642	F (4, 61) = 13.13	P < 0.0001
Column Factor	18967	6	3161	F (6, 61) = 8.942	P < 0.0001
Residual	21564	61	353,5		

CDDP treatment of neuroblastoma cells induces changes in $[Ca^{2+}]_i$ in a time- and concentration-dependent manner. Two-way ANOVA statistical analysis for the increasing fluorescence intensity following exposure to increasing concentrations of CDDP (0.001-1 µM) SH-SY5Y cells, expressed as percentage intensity relative to untreated fluorescence levels (% intensity) indicates significant variance that is both time- and concentration-dependent, $p < 0.001$ (***).

Table Analyzed	Ca2+ TOPO SH-SY5Y				
Two-way ANOVA	Ordinary				
Alpha	0,05				
Source of Variatio	% of total variation	P value	P value sumr	Significant?	
Interaction	14,97	0,0444	*	Yes	
Row Factor	36,81	< 0.0001	****	Yes	
Column Factor	27,77	< 0.0001	****	Yes	
ANOVA table	SS	DF	MS	F (DFn, DFd)	P value
Interaction	8685	18	482,5	F (18, 48) = 1.861	P = 0.0444
Row Factor	21353	3	7118	F (3, 48) = 27.46	P < 0.0001
Column Factor	16111	6	2685	F (6, 48) = 10.36	P < 0.0001
Residual	12442	48	259,2		

TOPO treatment of neuroblastoma cells induces changes in $[Ca^{2+}]_i$ in a time- and concentration-dependent manner. Two-way ANOVA statistical analysis for the increasing fluorescence intensity following exposure to increasing concentrations of TOPO (0.0001-1 µM) SH-SY5Y cells, expressed as percentage intensity relative to untreated fluorescence levels (% intensity) indicates significant variance that is both time- and concentration-dependent, $p < 0.001$ (***).

Table Analyzed	Ca2+ CDDP IMR-32				
Two-way ANOVA	Ordinary				
Alpha	0,05				
Source of Variation	% of total variation	P value	P value sumr	Significant?	
Interaction	10,95	0,6186	ns	No	
Row Factor	35,45	< 0.0001	****	Yes	
Column Factor	18,77	< 0.0001	****	Yes	
ANOVA table	SS	DF	MS	F (DFn, DFd)	P value
Interaction	34950	24	1456	F (24, 65) = 0.8857	P = 0.6186
Row Factor	113137	4	28284	F (4, 65) = 17.20	P < 0.0001
Column Factor	59916	6	9986	F (6, 65) = 6.073	P < 0.0001
Residual	106876	65	1644		

CDDP treatment of neuroblastoma cells induces changes in $[Ca^{2+}]_i$ in a time- and concentration-dependent manner. Two-way ANOVA statistical analysis for the increasing fluorescence intensity following exposure to increasing concentrations of CDDP (0.001-1 µM) IMR-32 cells, expressed as percentage intensity relative to untreated fluorescence levels (% intensity) indicates significant variance that is both time- and concentration-dependent, $p < 0.001$ (***).

Table Analyzed	Ca2+ TOPO IMR-32				
Two-way ANOVA	Ordinary				
Alpha	0,05				
Source of Variation	% of total variation	P value	P value sumr	Significant?	
Interaction	9,459	0,9007	ns	No	
Row Factor	32,93	< 0.0001	****	Yes	
Column Factor	16,56	0,0009	***	Yes	
ANOVA table	SS	DF	MS	F (DFn, DFd)	P value
Interaction	2489	24	103,7	F (24, 67) = 0.6241	P = 0.9007
Row Factor	8666	4	2166	F (4, 67) = 13.04	P < 0.0001
Column Factor	4358	6	726,3	F (6, 67) = 4.371	P = 0.0009
Residual	11133	67	166,2		

TOPO treatment of neuroblastoma cells induces changes in [Ca2+]i in a time- and concentration-dependent manner. Two-way ANOVA statistical analysis for the increasing fluorescence intensity following exposure to increasing concentrations of TOPO (0.0001-1 µM) IMR-32 cells, expressed as percentage intensity relative to untreated fluorescence levels (% intensity) indicates significant variance that is both time- and concentration-dependent, $p < 0.001$ (***).

Supplementary File 1: Full listing of differentially expressed mRNA in the comparison CDDP vs. control. See_Supplementary_File 1

Supplementary File 2: RAMONA outcome of the enrichment of gene ontology terms for differentially expressed mRNA in the comparison CDDP vs. control. See_Supplementary_File 2

Supplementary File 3: RAMONA outcome of the enrichment of KEGG pathway for differentially expressed mRNA in the comparison CDDP vs. control. See_Supplementary_File 3

Supplementary File 4: Validation of the microarray analysis results using RT-PCR. See_Supplementary_File 4

Supplementary File 5: Validation of the microarray analysis results using RT-PCR. See_Supplementary_File 5

Additional information not included in the text, primers:

CALM1_ fwd: CCGTGCCGTTACTCGTAGTC;
CALM1_REV: CAATCTGTTCTTCGGTCAGC;
CAMTA1_fwd: ATTCTGCGGAACTAGCACCT;
CAMTA1_REV: ATTTCGGCAGACATTCAAGC;
GDF15_fwd: CTCCAGATTCCGAGAGTTGC;
GDF15_REV: AGAGATACGCAGGTGCAGGT;
PLCD3_fwd: CGCAAGAACCTGGACCTG;
PLCD3_REV: CAGCAGGCTCTTGATCTCCT;
MYC_fwd: TTTCGGGTAGTGGAAAACCA;
MYC_REV: CAGCAGCTCGAATTTCTTCC;
PLCH1_fwd: TGATGAGGAAGACACACAGCA;
PLCH1_REV: TCATCCACAATGTCCTGAGC;
NNAT_fwd: TTTCTCGACCACCCACCTAC;
NNAT_REV: CTGTGTCCCTGGAGGATTTC;
PPEF1_fwd: GACTGGGAAACCCTCTTCAA;
PPEF1_REV: CAAATGCTCTCTGGCTTTCC;
ABCG2_fwd: CGGGTGACTCATCCCAACAT;
ABCG2_REV: CAGGATCTCAGGATGCGTGC;
ABCB1_fwd: CCCATCATTGCAATAGCAGG;
ABCB1_REV: GTTCAAACTTCTGCTCCTGA;
ABCC1_fwd: TGGGACTGGAATGTCACG;
ABCC1_REV: AGGAATATGCCCCGACTTC.
