# Supplementary material for: Calcium-regulatory proteins as modulators of chemotherapy in human neuroblastoma
Source: Oncotarget. 2017 Feb 11;8(14):22876–93. doi: 10.18632/oncotarget.15283 (PMC5410270; doi:10.18632/oncotarget.15283)
Supplement: Supplementary file 5 [file oncotarget-08-22876-s005.pdf]

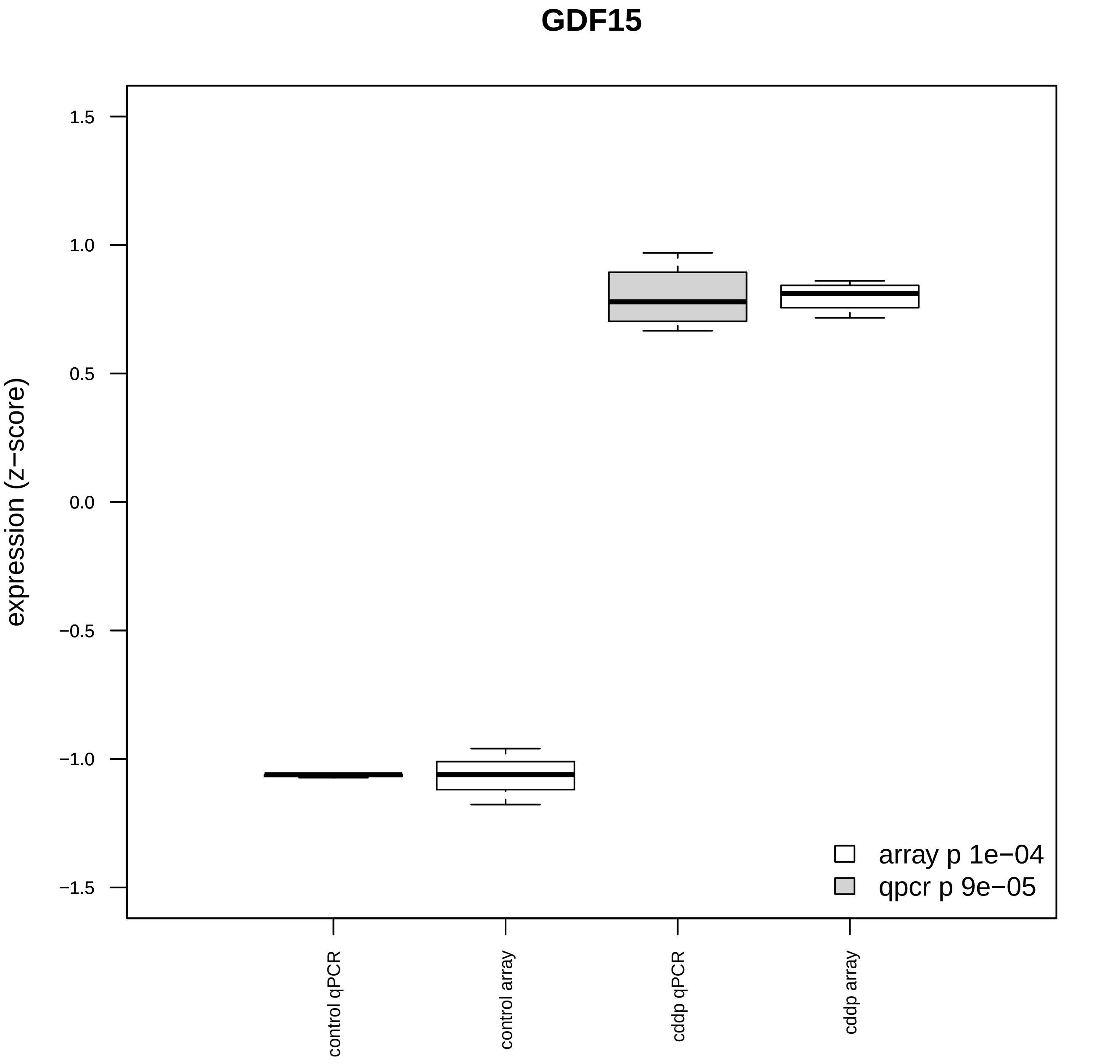
GDF15
expression (z−score)
1.5
1.0
0.5
0.0
−0.5
−1.0
−1.5
control qPCR
control array
cddp qPCR
cddp array
array p 1e−04
qpcr p 9e−05

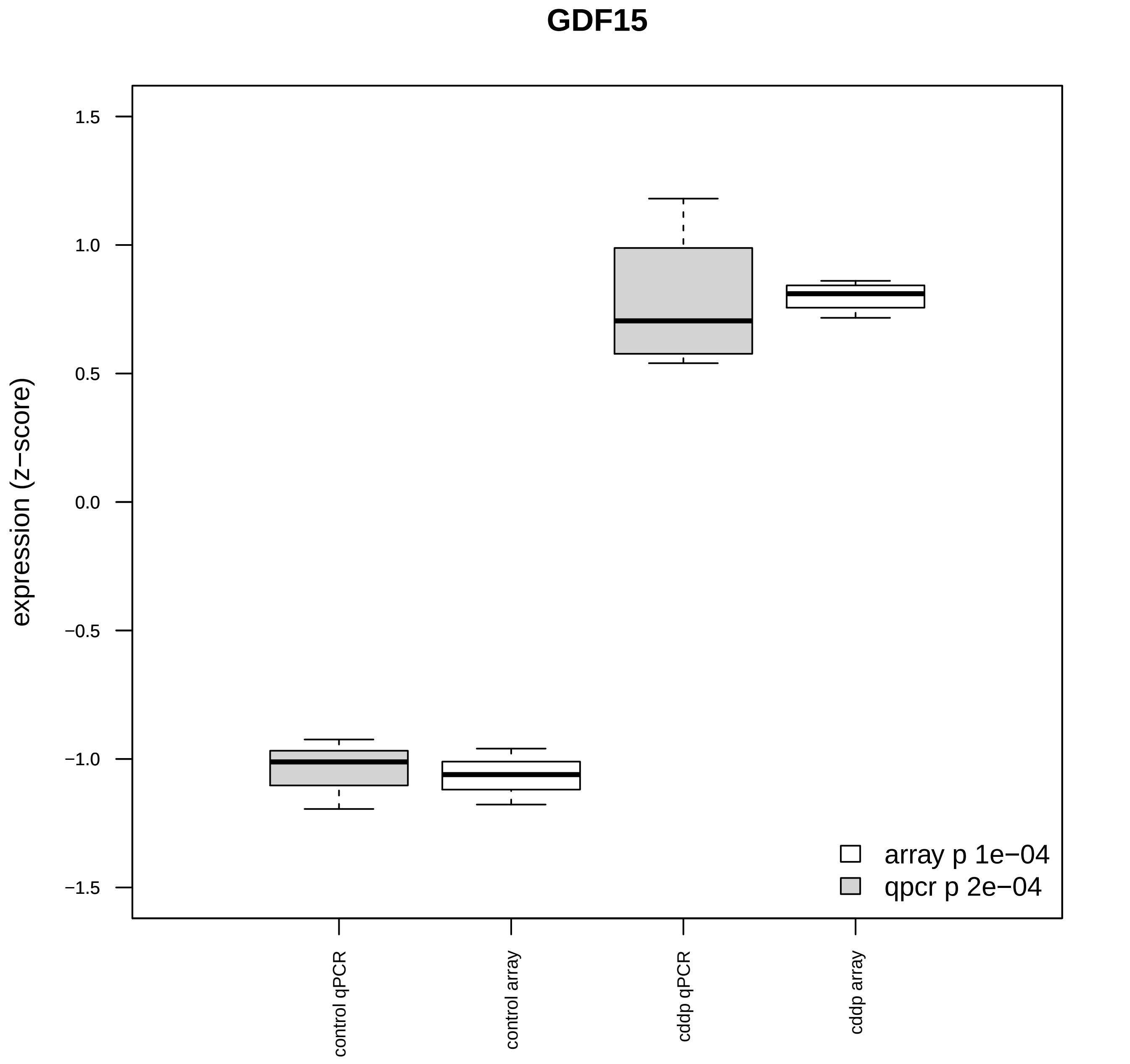

GDF15
expression (z−score)
1.5
1.0
0.5
0.0
−0.5
−1.0
−1.5
array p 1e−04
qpcr p 2e−04
control qPCR
control array
cddp qPCR
cddp array

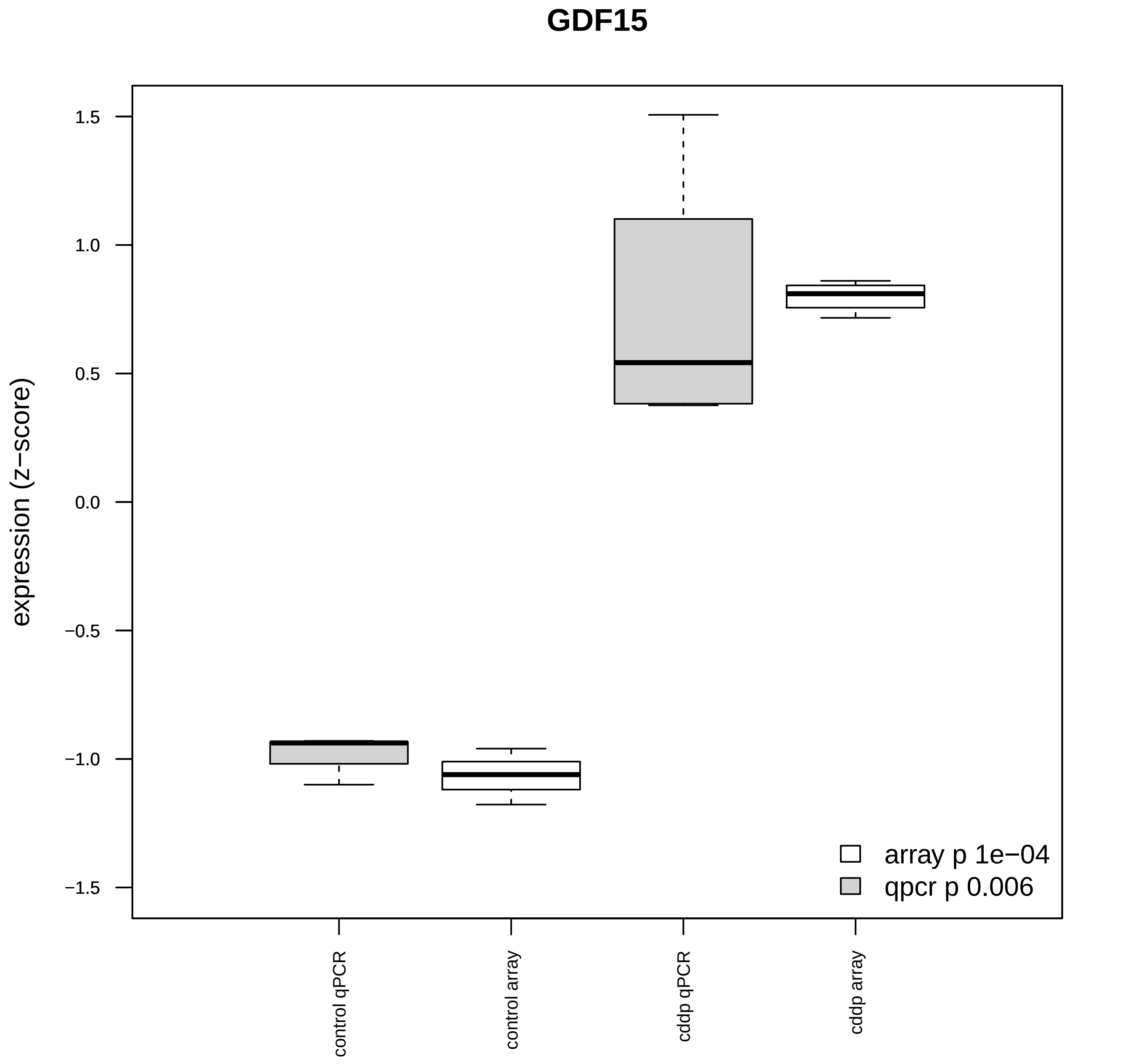
GDF15
expression (z−score)
1.5
1.0
0.5
0.0
−0.5
−1.0
−1.5
array p 1e−04
qpcr p 0.006
control qPCR
control array
cddp qPCR
cddp array

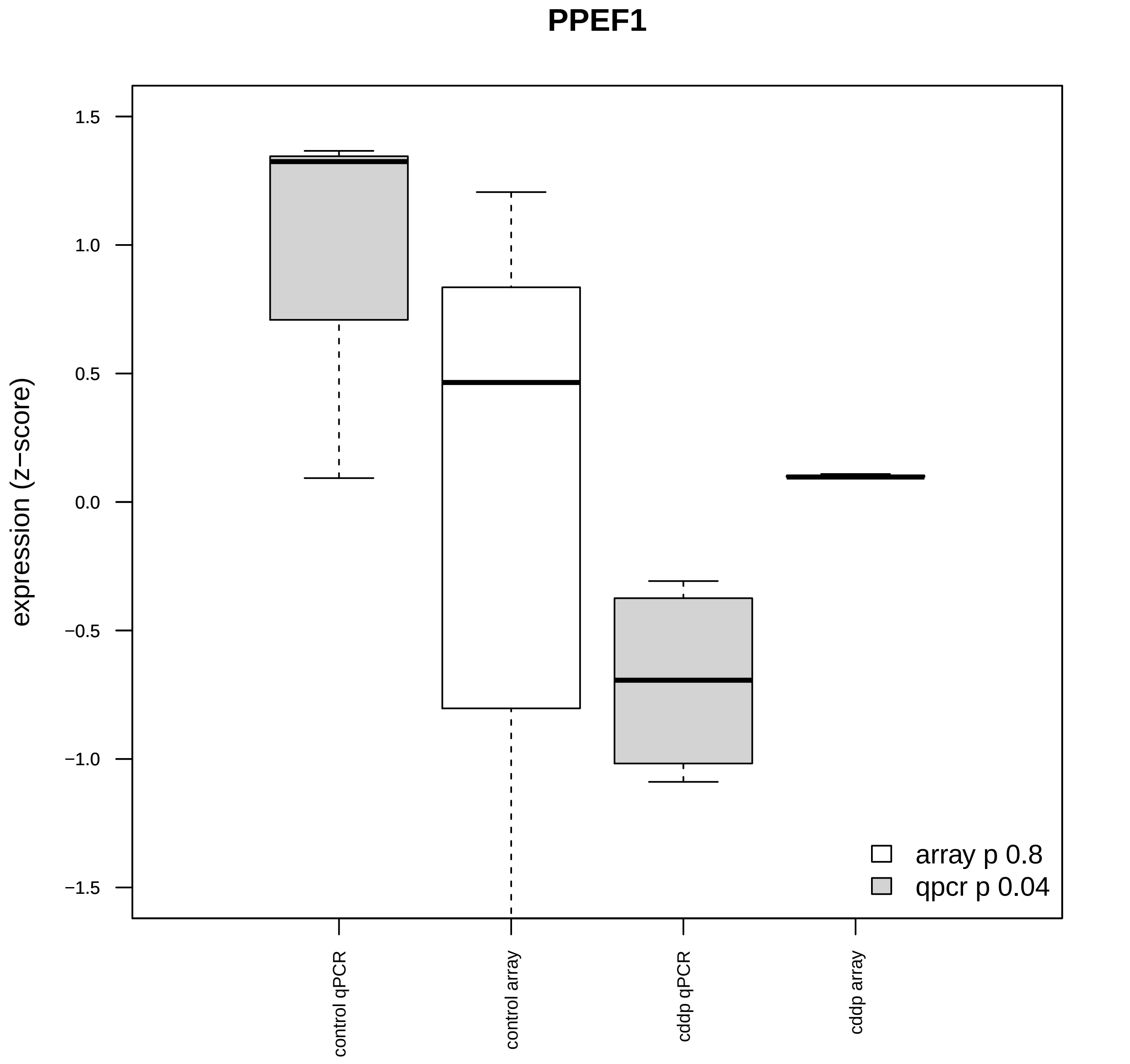
PPEF1
expression (z−score)
1.5
1.0
0.5
0.0
−0.5
−1.0
−1.5
array p 0.8
qpcr p 0.04
control qPCR
control array
cddp qPCR
cddp array

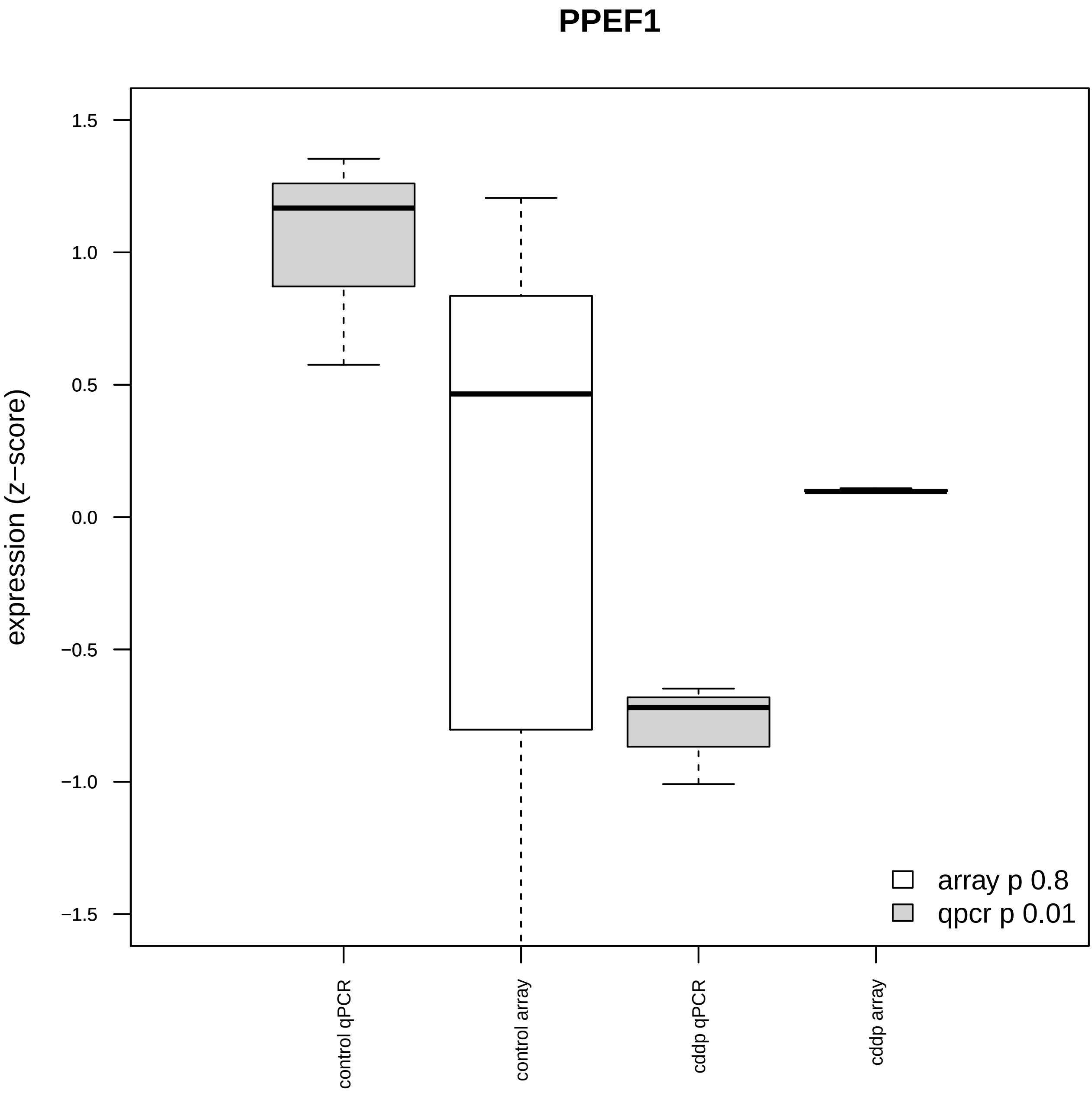
PPEF1
expression (z−score)
1.5
1.0
0.5
0.0
−0.5
−1.0
−1.5
control qPCR
control array
cddp qPCR
cddp array
array p 0.8
qpcr p 0.01

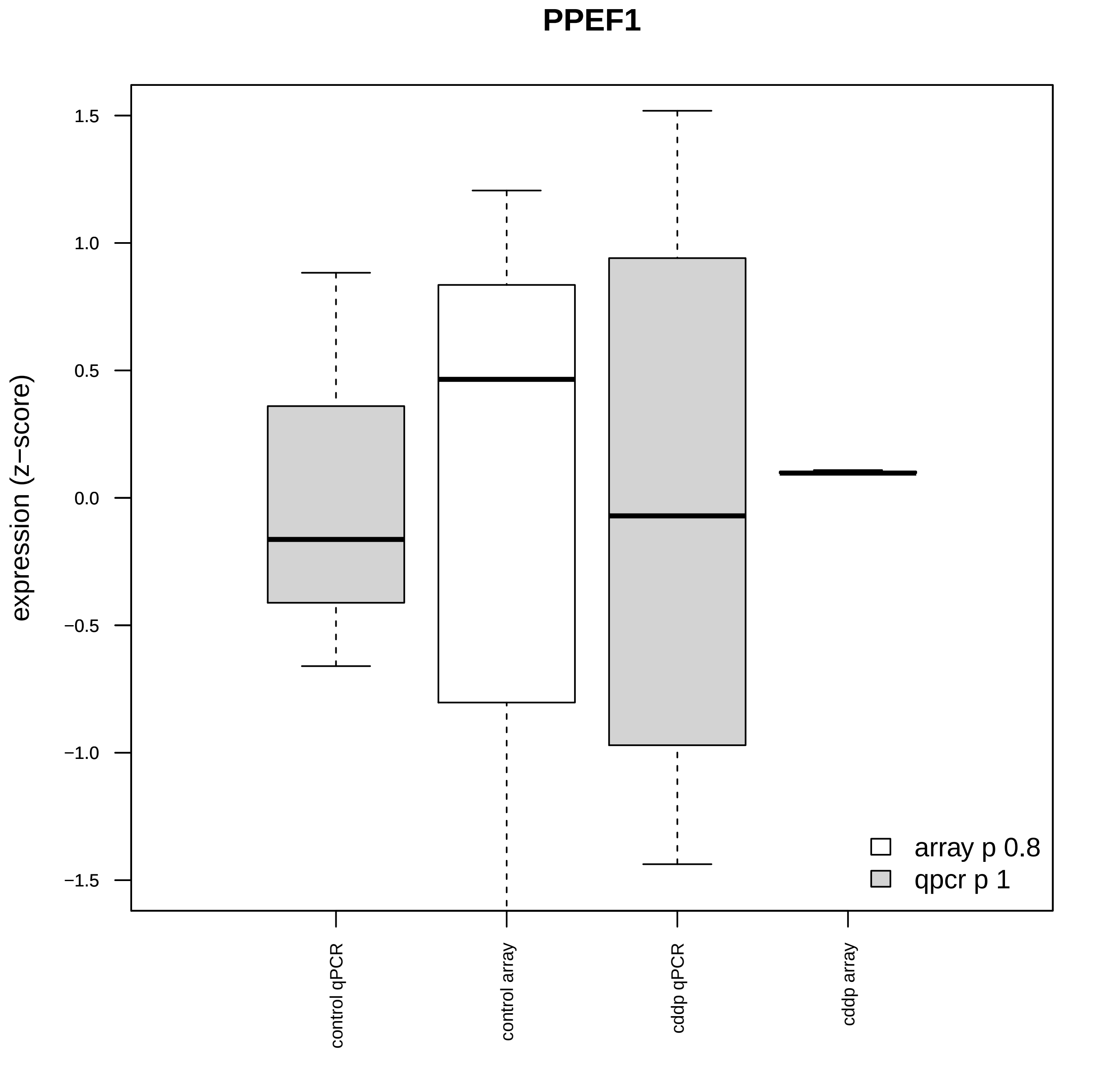
PPEF1
expression (z−score)
1.5
1.0
0.5
0.0
−0.5
−1.0
−1.5
control qPCR
control array
cddp qPCR
cddp array
array p 0.8
qpcr p 1

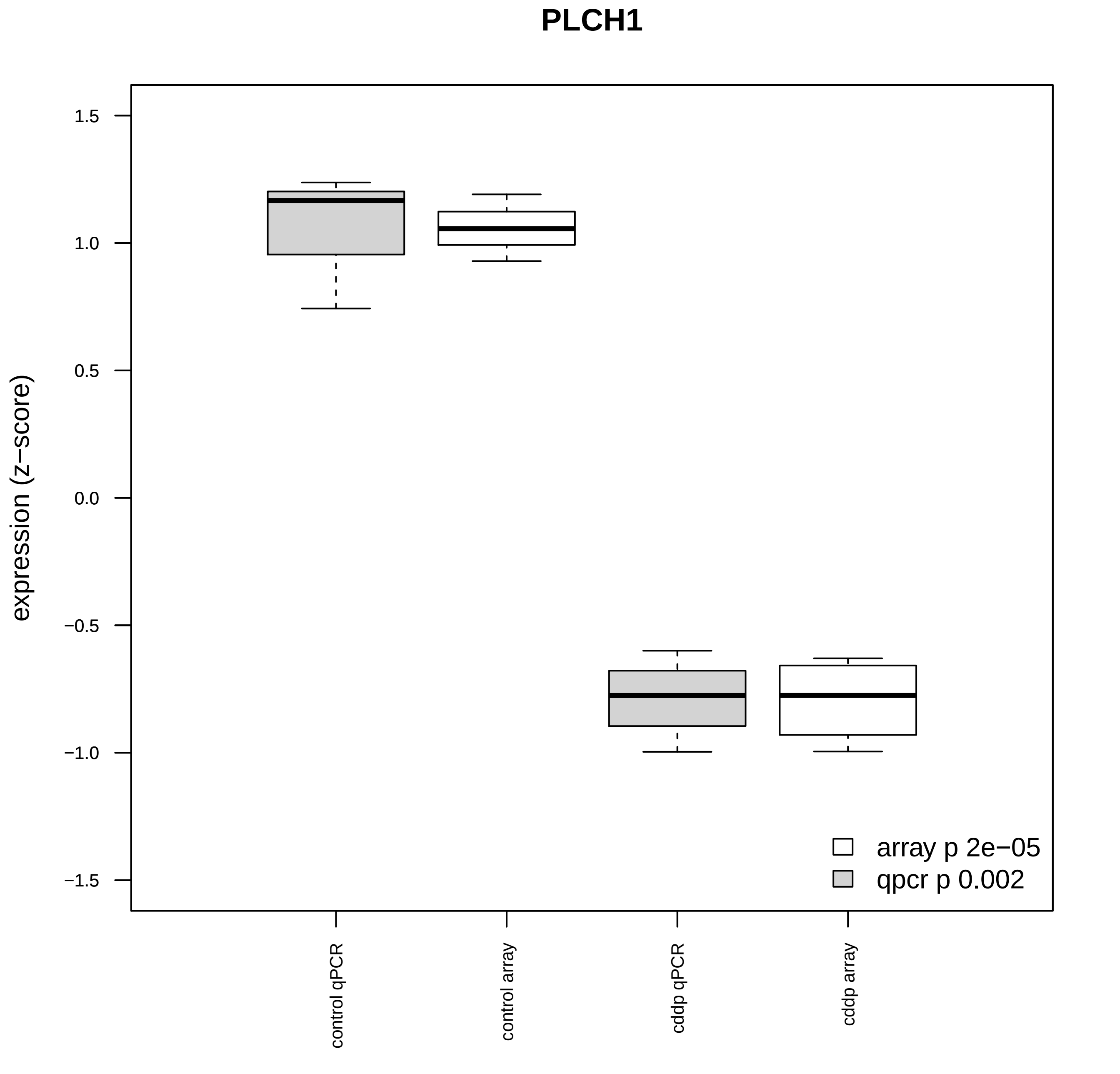

PLCH1
expression (z−score)
1.5
1.0
0.5
0.0
−0.5
−1.0
−1.5
control qPCR
control array
cddp qPCR
cddp array
array p 2e−05
qpcr p 0.002

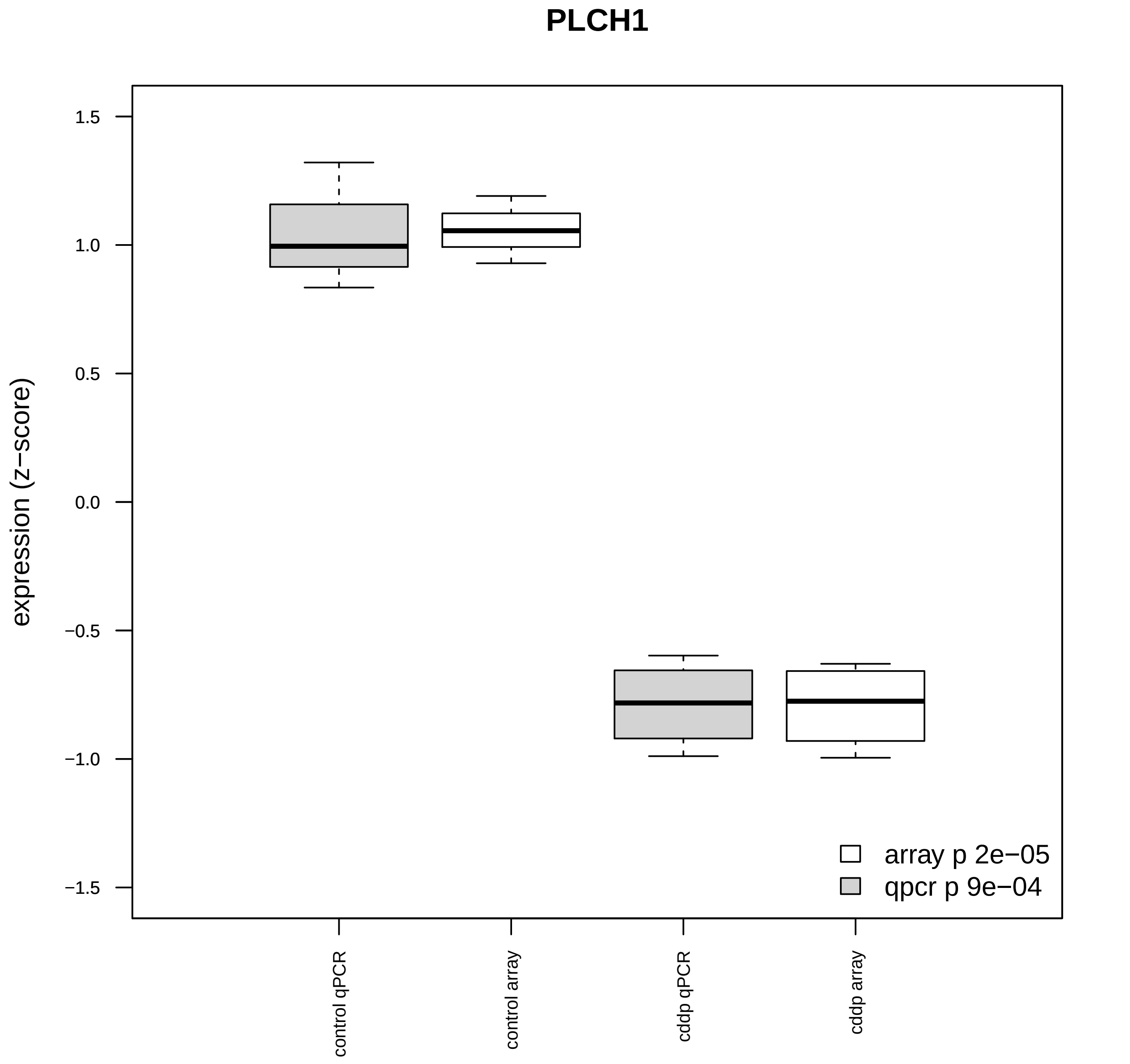
PLCH1
expression (z−score)
1.5
1.0
0.5
0.0
−0.5
−1.0
−1.5
array p 2e−05
qpcr p 9e−04
control qPCR
control array
cddp qPCR
cddp array

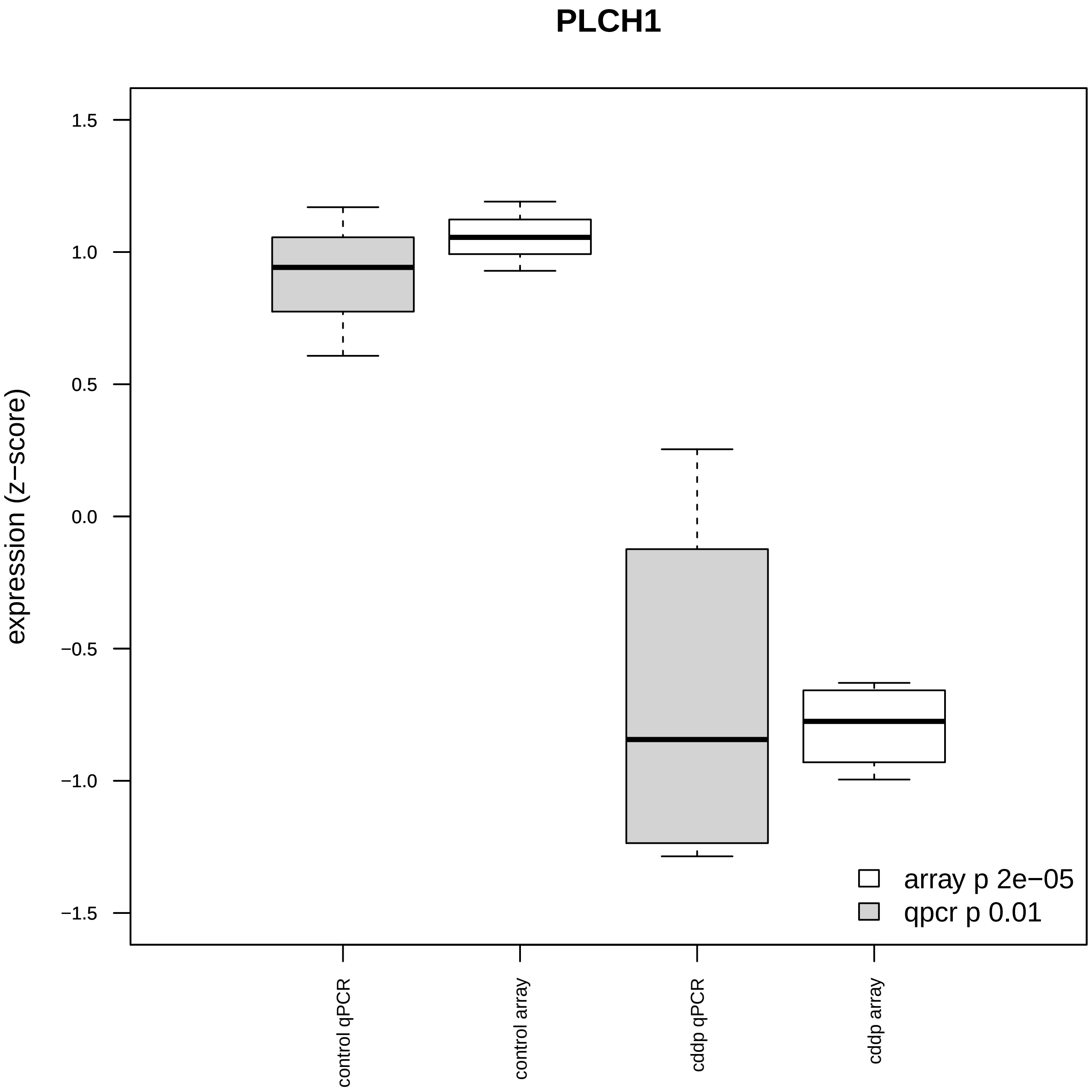
PLCH1
expression (z−score)
1.5
1.0
0.5
0.0
−0.5
−1.0
−1.5
control qPCR
control array
cddp qPCR
cddp array
array p 2e−05
qpcr p 0.01

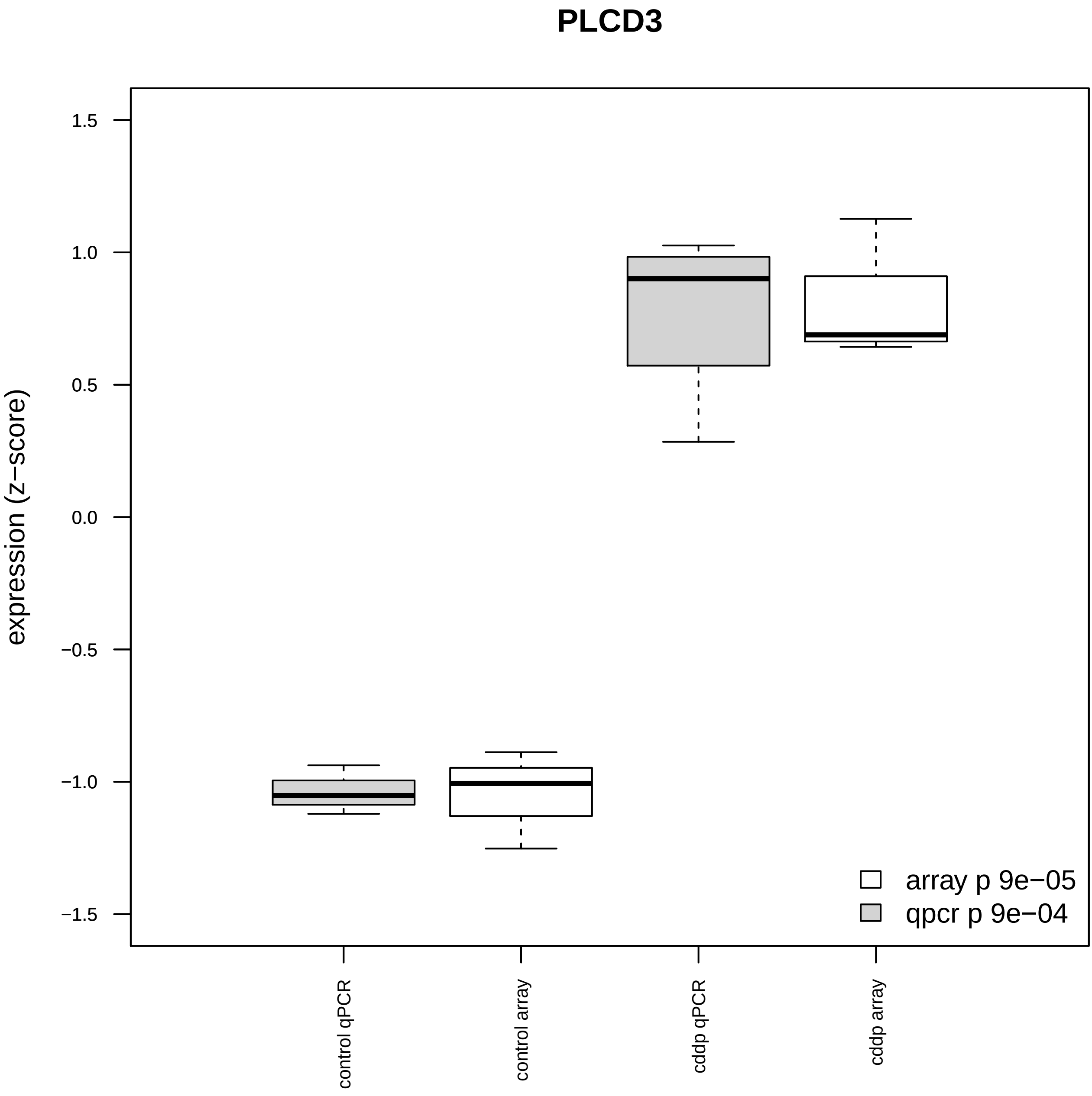
PLCD3
expression (z−score)
1.5
1.0
0.5
0.0
−0.5
−1.0
−1.5
control qPCR
control array
cddp qPCR
cddp array
array p 9e−05
qpcr p 9e−04

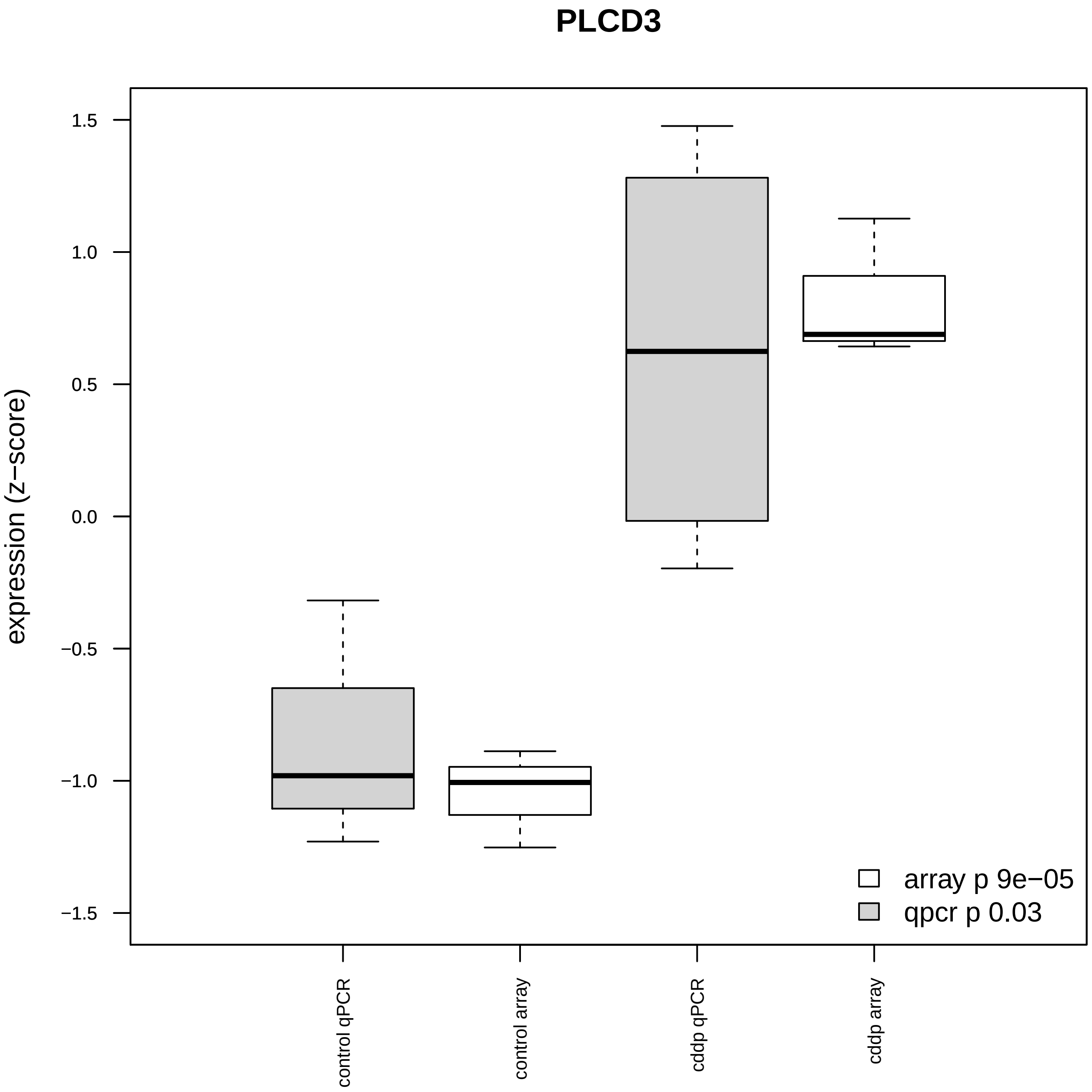
PLCD3
expression (z−score)
1.5
1.0
0.5
0.0
−0.5
−1.0
−1.5
control qPCR
control array
cddp qPCR
cddp array
array p 9e−05
qpcr p 0.03

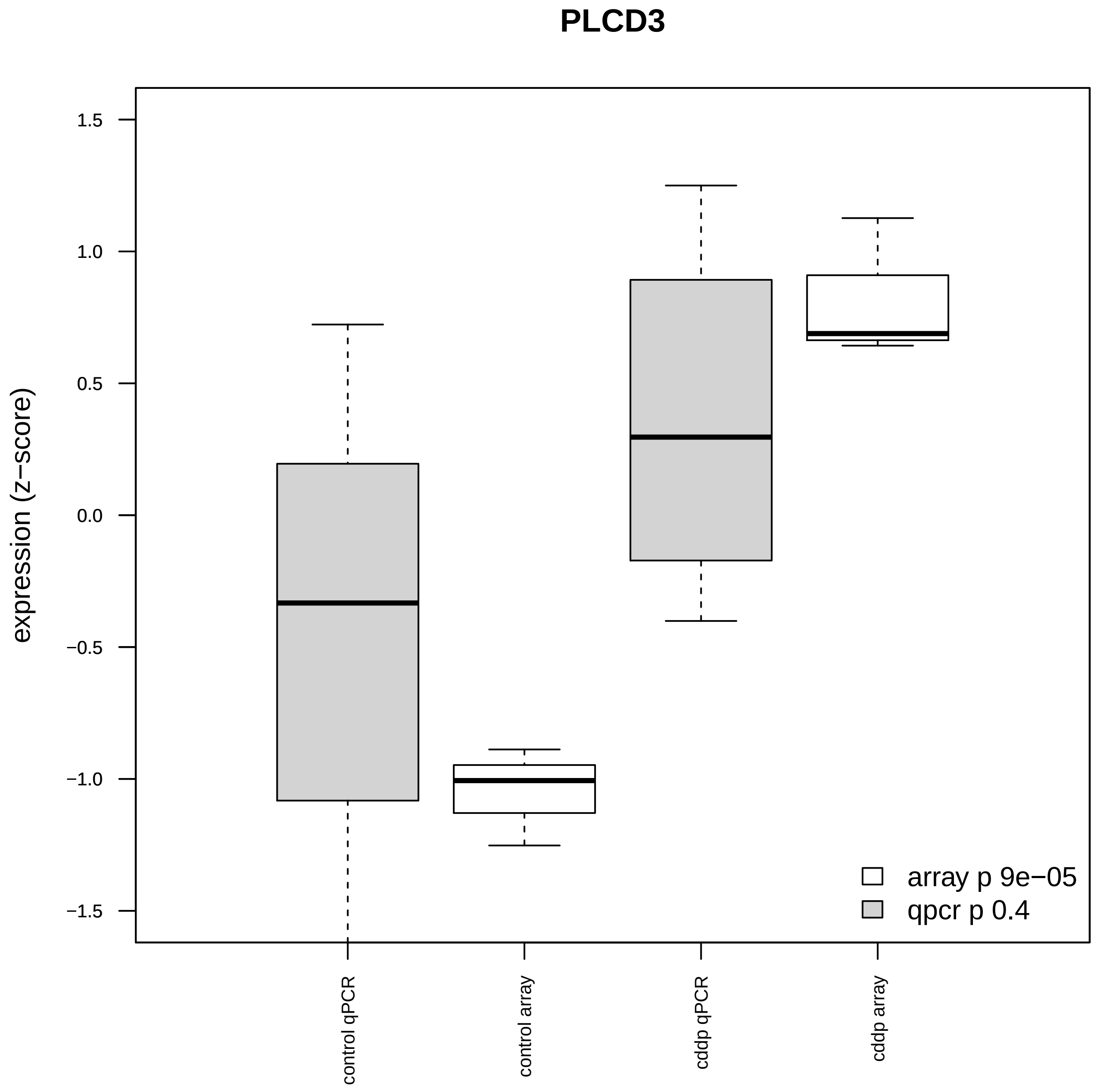

PLCD3
expression (z−score)
1.5
1.0
0.5
0.0
−0.5
−1.0
−1.5
control qPCR
control array
cddp qPCR
cddp array
array p 9e−05
qpcr p 0.4

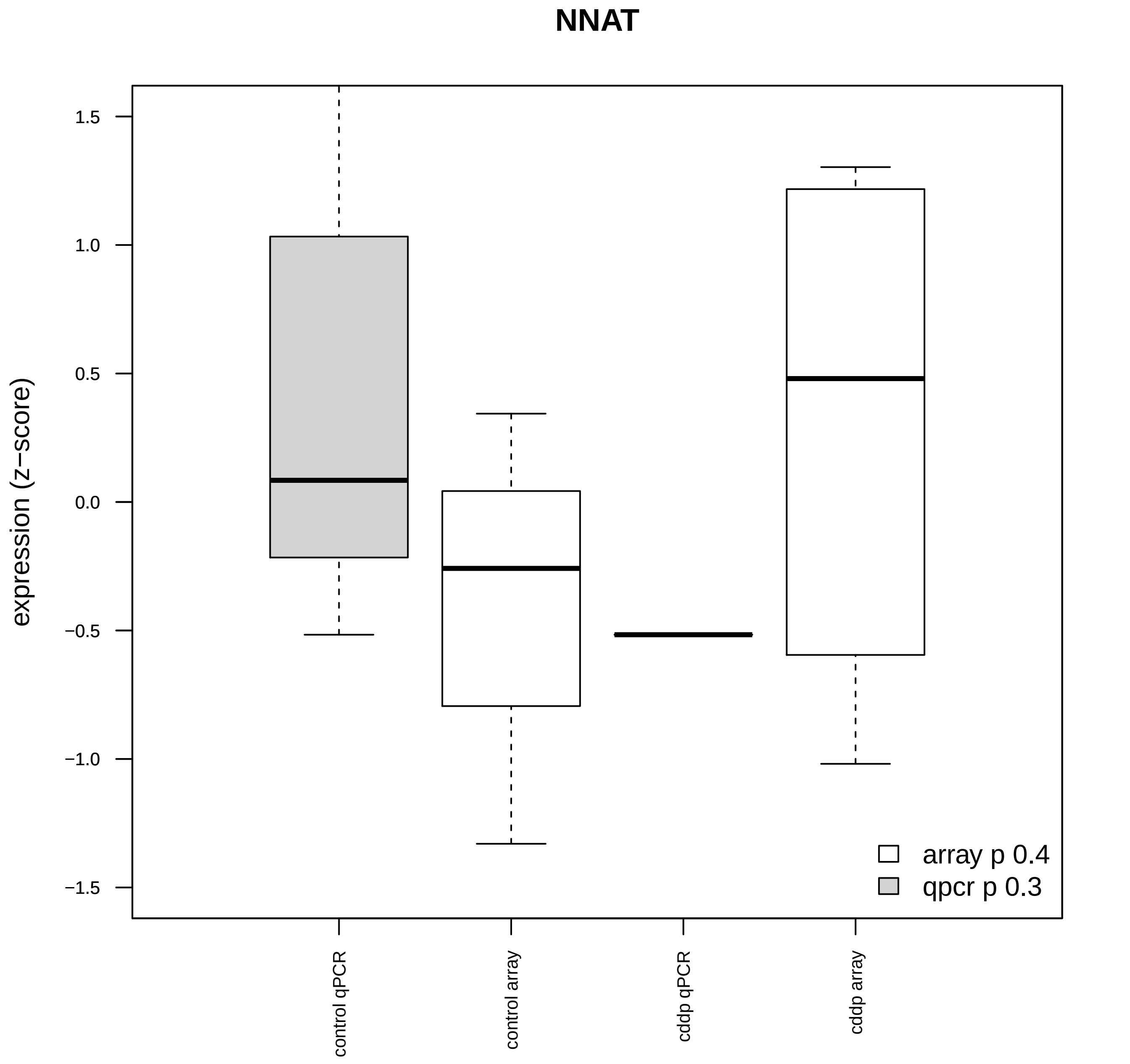
NNAT
expression (z−score)
1.5
1.0
0.5
0.0
−0.5
−1.0
−1.5
array p 0.4
qpcr p 0.3
control qPCR
control array
cddp qPCR
cddp array

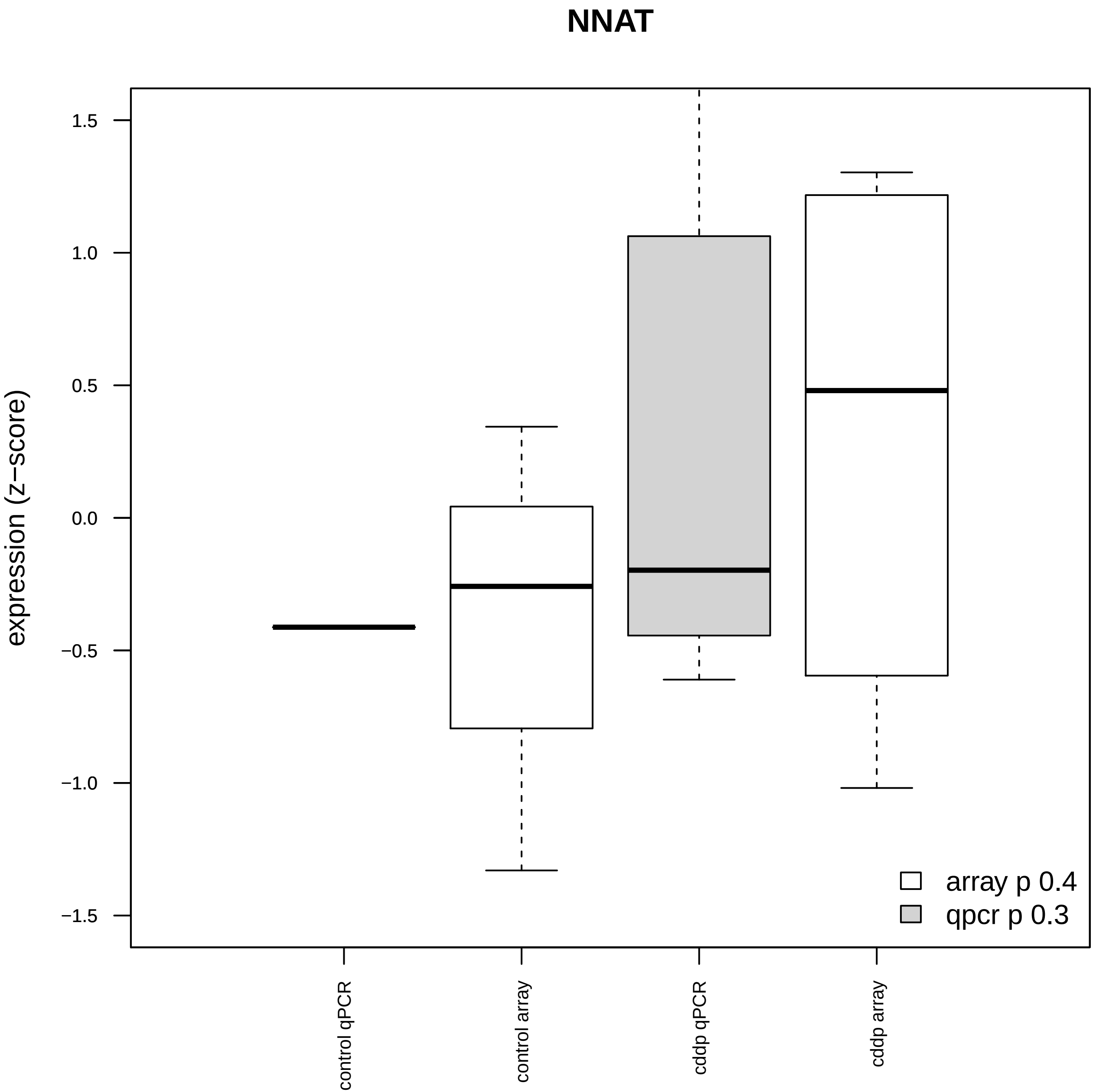
NNAT
expression (z−score)
1.5
1.0
0.5
0.0
−0.5
−1.0
−1.5
control qPCR
control array
cddp qPCR
cddp array
array p 0.4
qpcr p 0.3

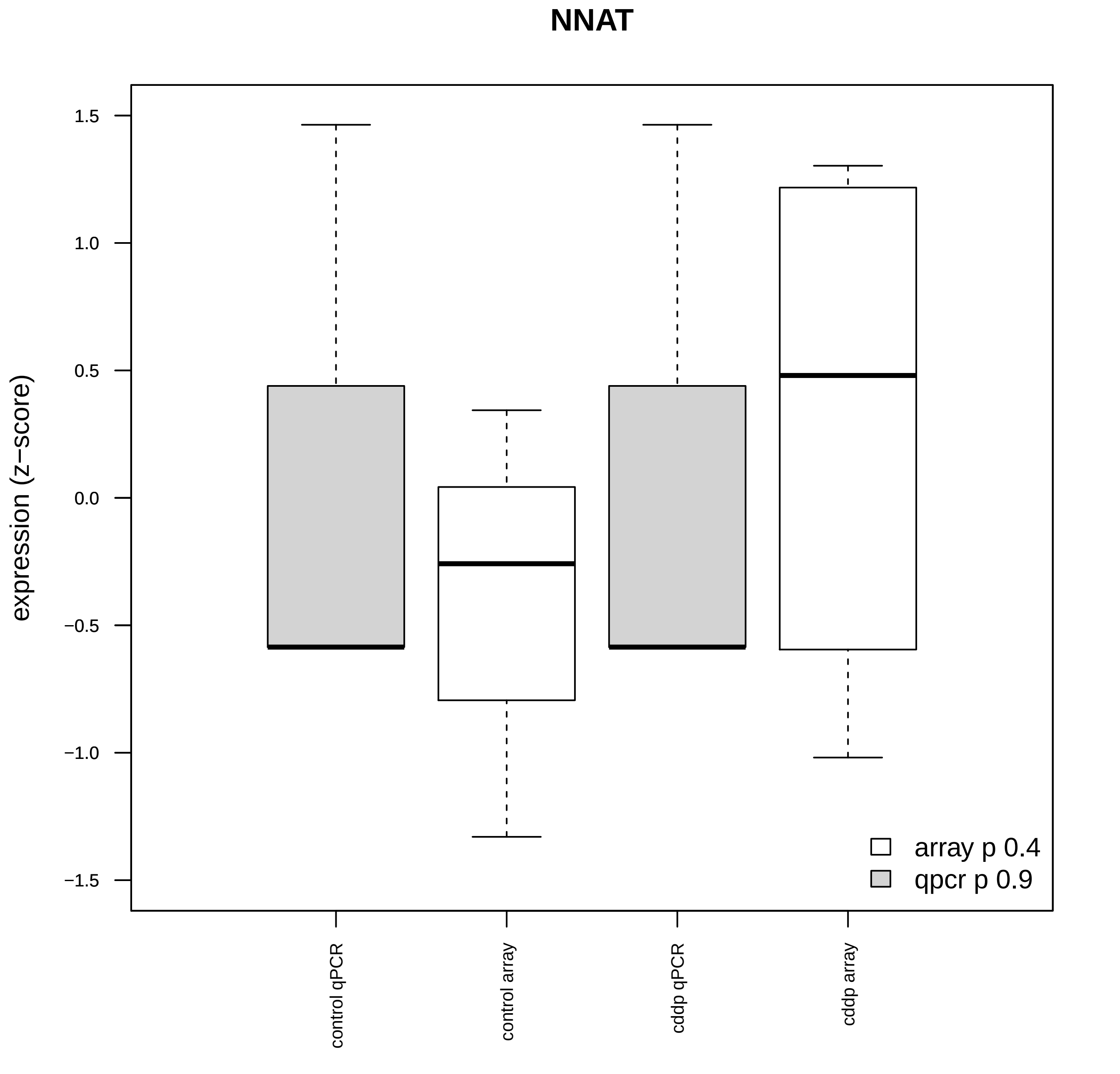
NNAT
expression (z−score)
1.5
1.0
0.5
0.0
−0.5
−1.0
−1.5
array p 0.4
qpcr p 0.9
control qPCR
control array
cddp qPCR
cddp array

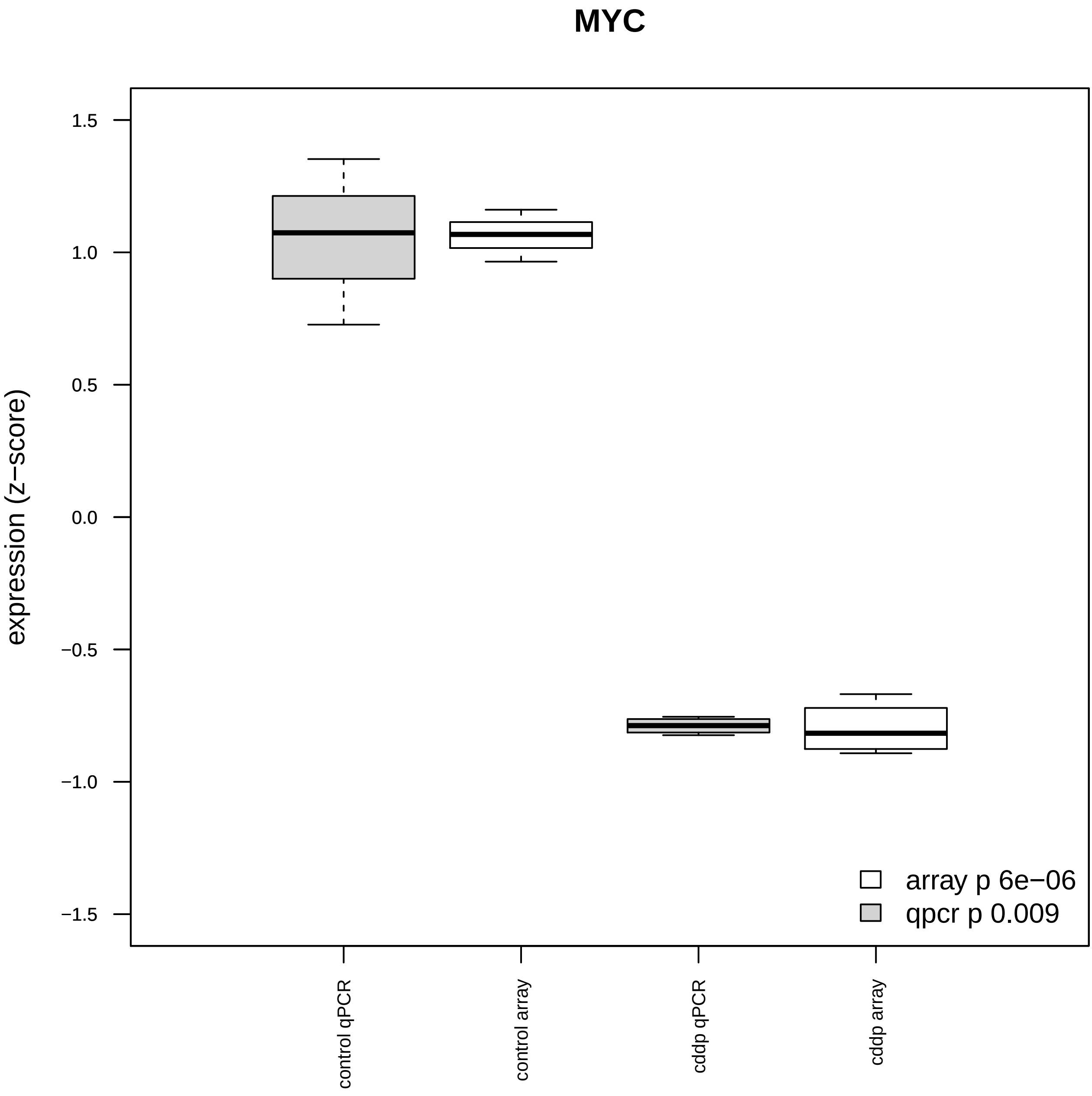
MYC
expression (z−score)
1.5
1.0
0.5
0.0
−0.5
−1.0
−1.5
array p 6e−06
qpcr p 0.009
control qPCR
control array
cddp qPCR
cddp array

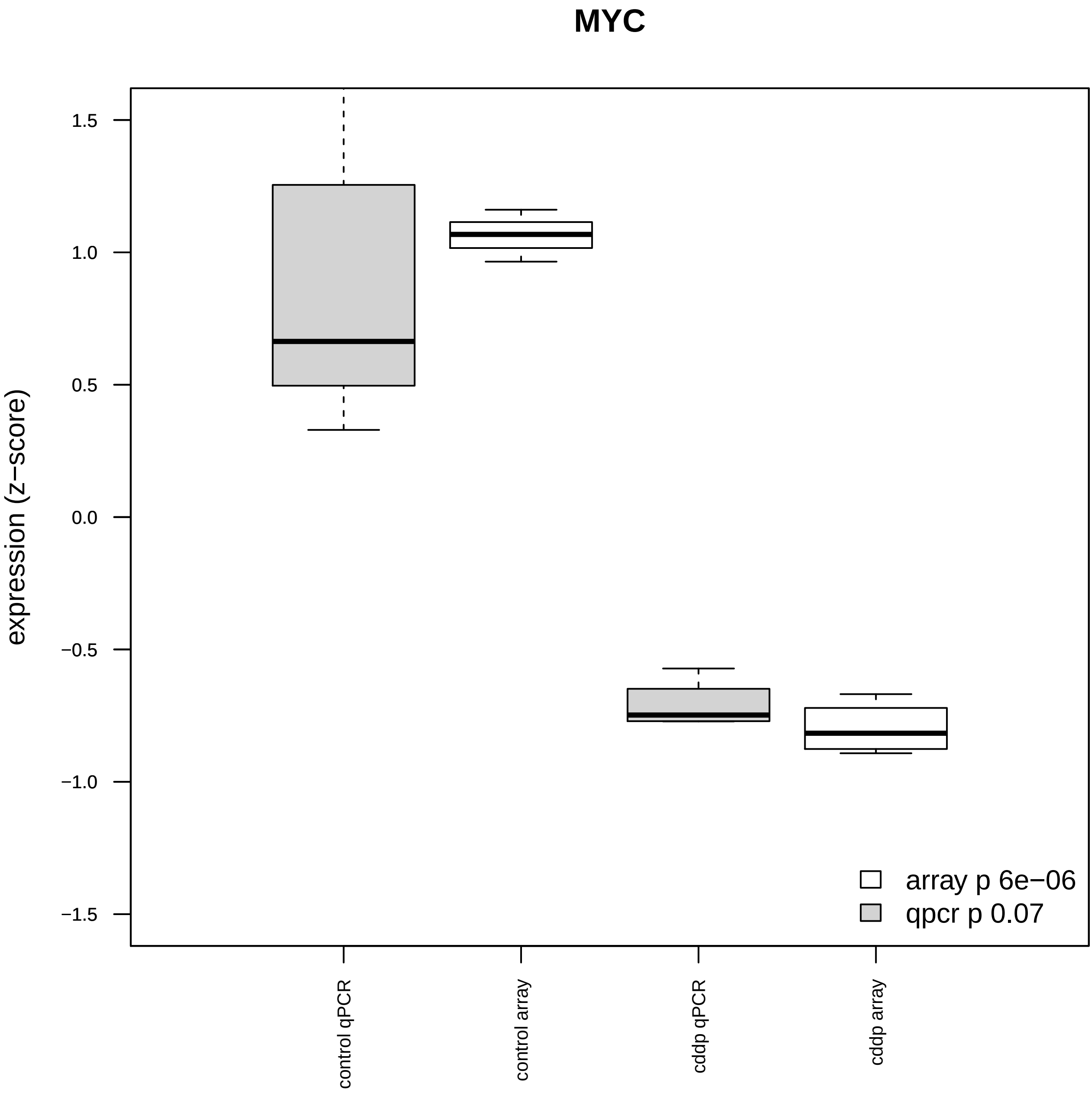
MYC
expression (z−score)
1.5
1.0
0.5
0.0
−0.5
−1.0
−1.5
control qPCR
control array
cddp qPCR
cddp array
array p 6e−06
qpcr p 0.07

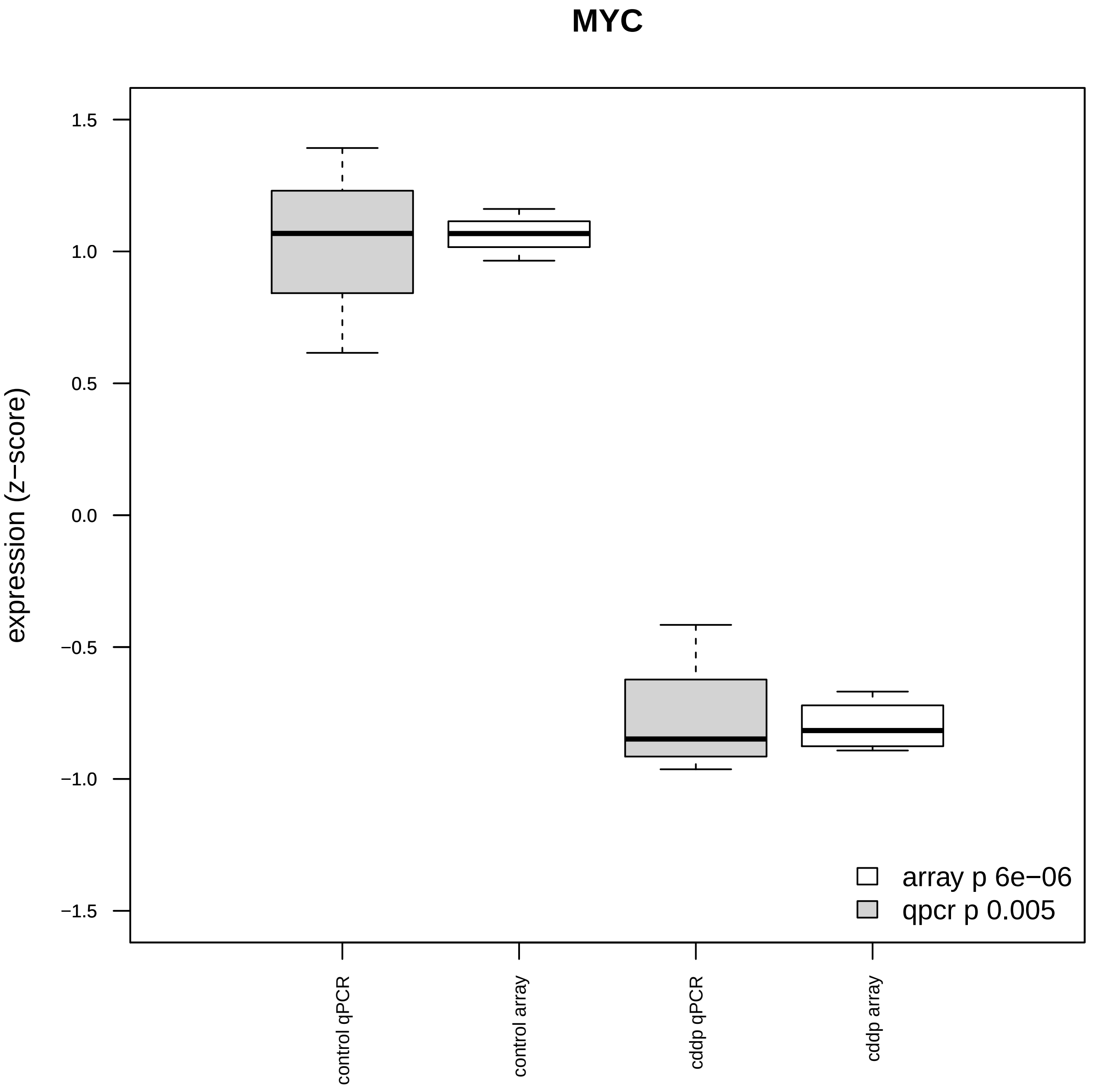

MYC
expression (z−score)
1.5
1.0
0.5
0.0
−0.5
−1.0
−1.5
array p 6e−06
qpcr p 0.005
control qPCR
control array
cddp qPCR
cddp array

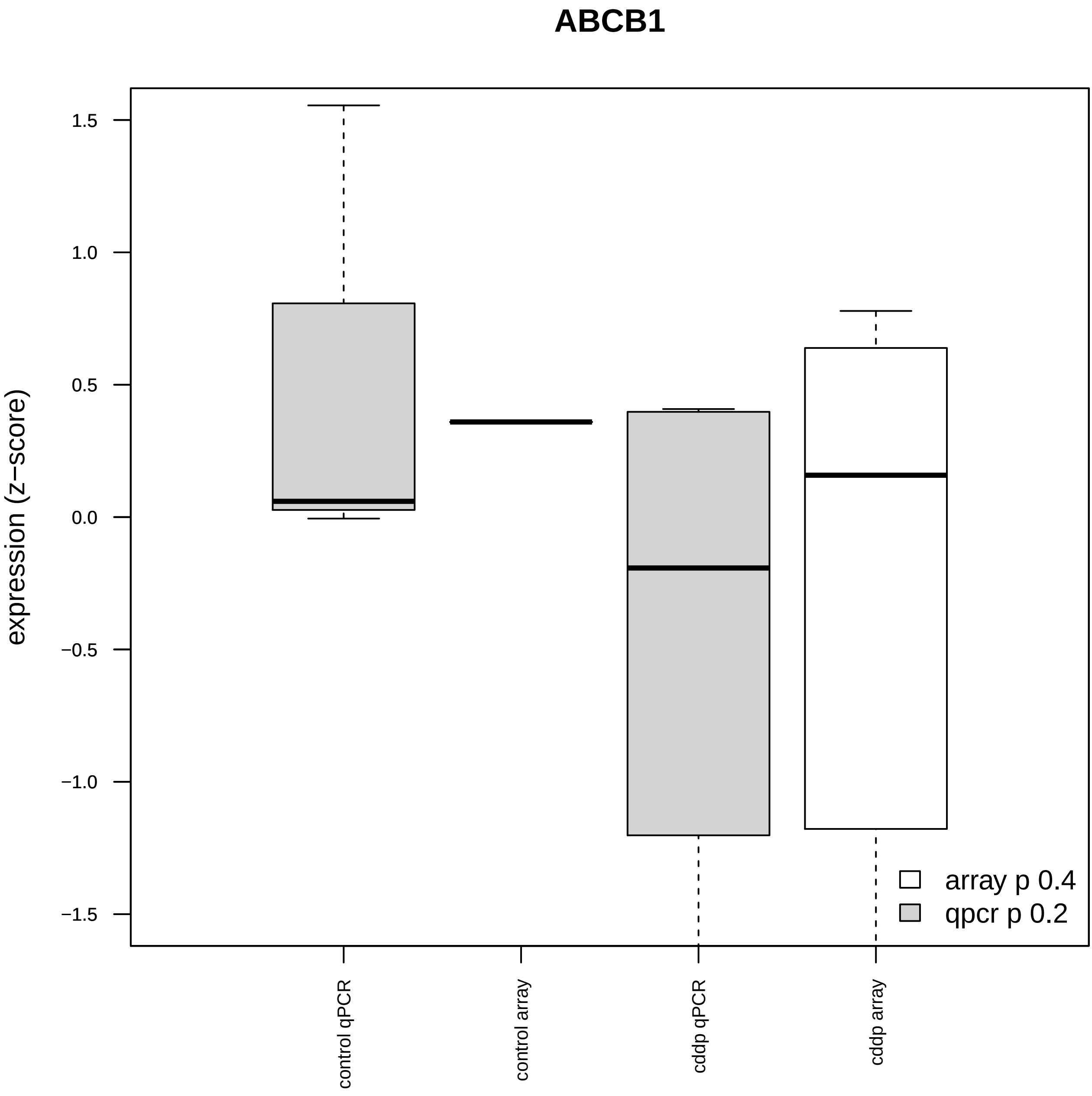
ABCB1
expression (z−score)
1.5
1.0
0.5
0.0
−0.5
−1.0
−1.5
control qPCR
control array
cddp qPCR
cddp array
array p 0.4
qpcr p 0.2

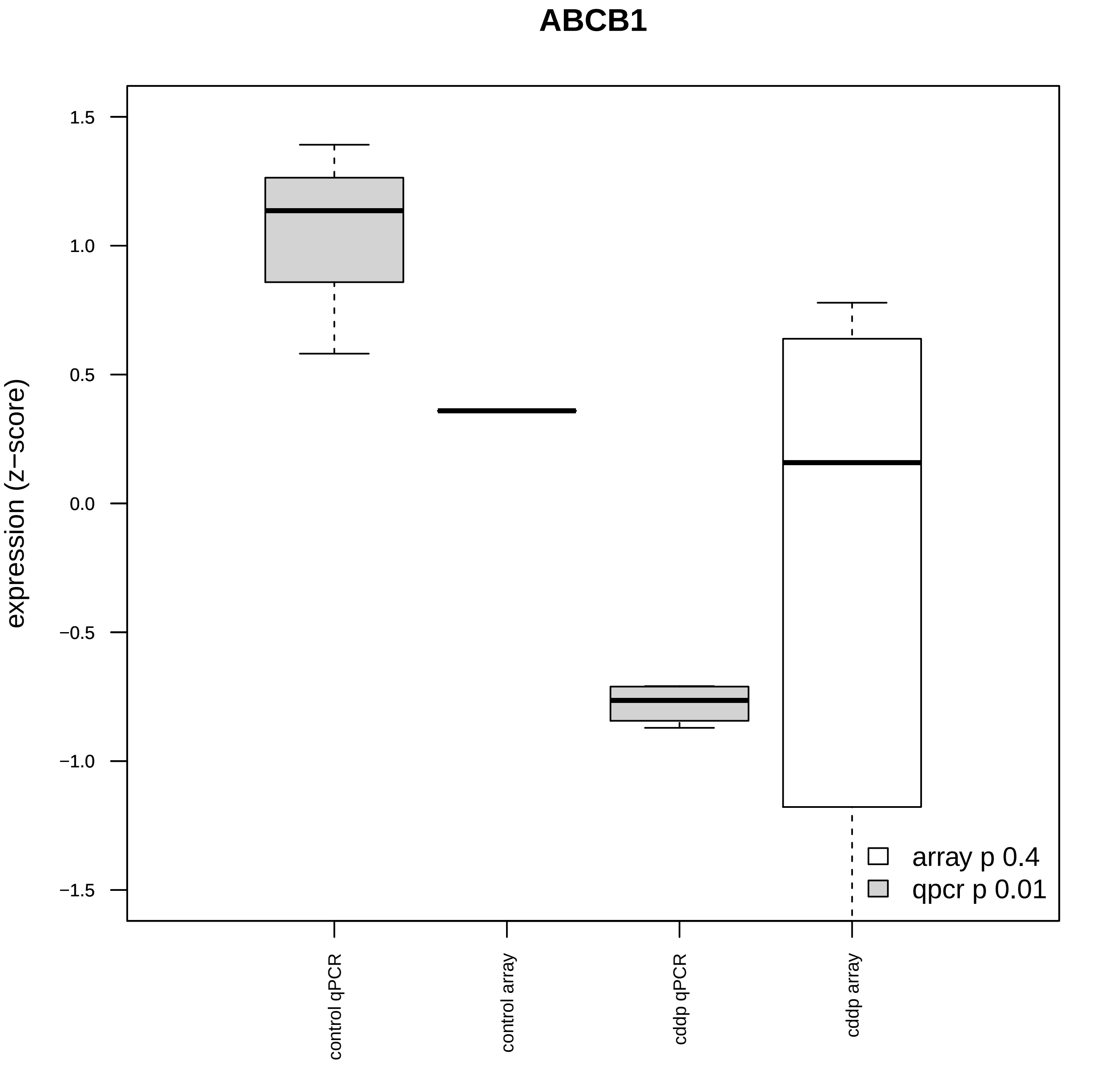
ABCB1
expression (z−score)
1.5
1.0
0.5
0.0
−0.5
−1.0
−1.5
array p 0.4
qpcr p 0.01
control qPCR
control array
cddp qPCR
cddp array

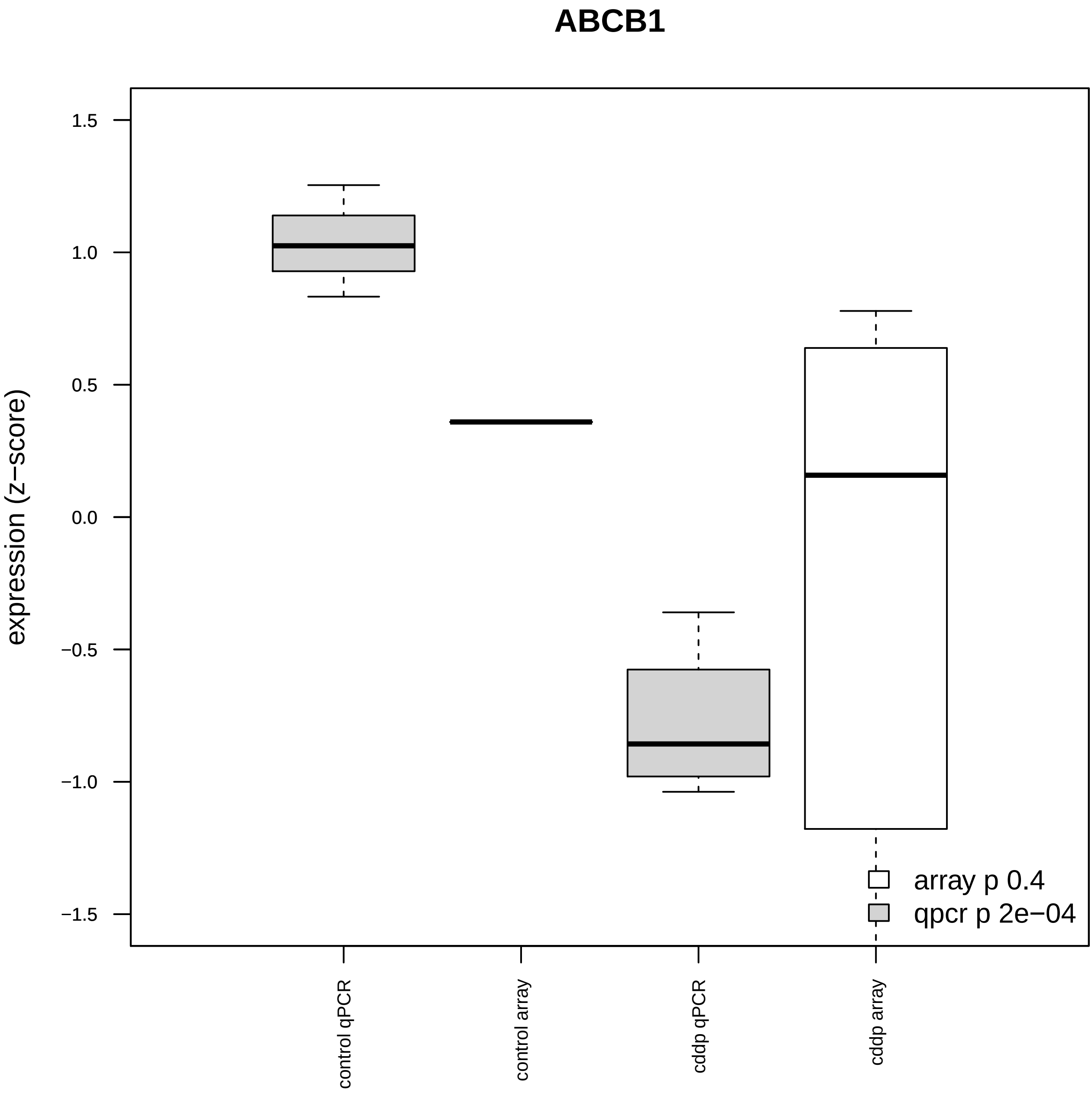

ABCB1
expression (z−score)
1.5
1.0
0.5
0.0
−0.5
−1.0
−1.5
control qPCR
control array
cddp qPCR
cddp array
array p 0.4
qpcr p 2e−04

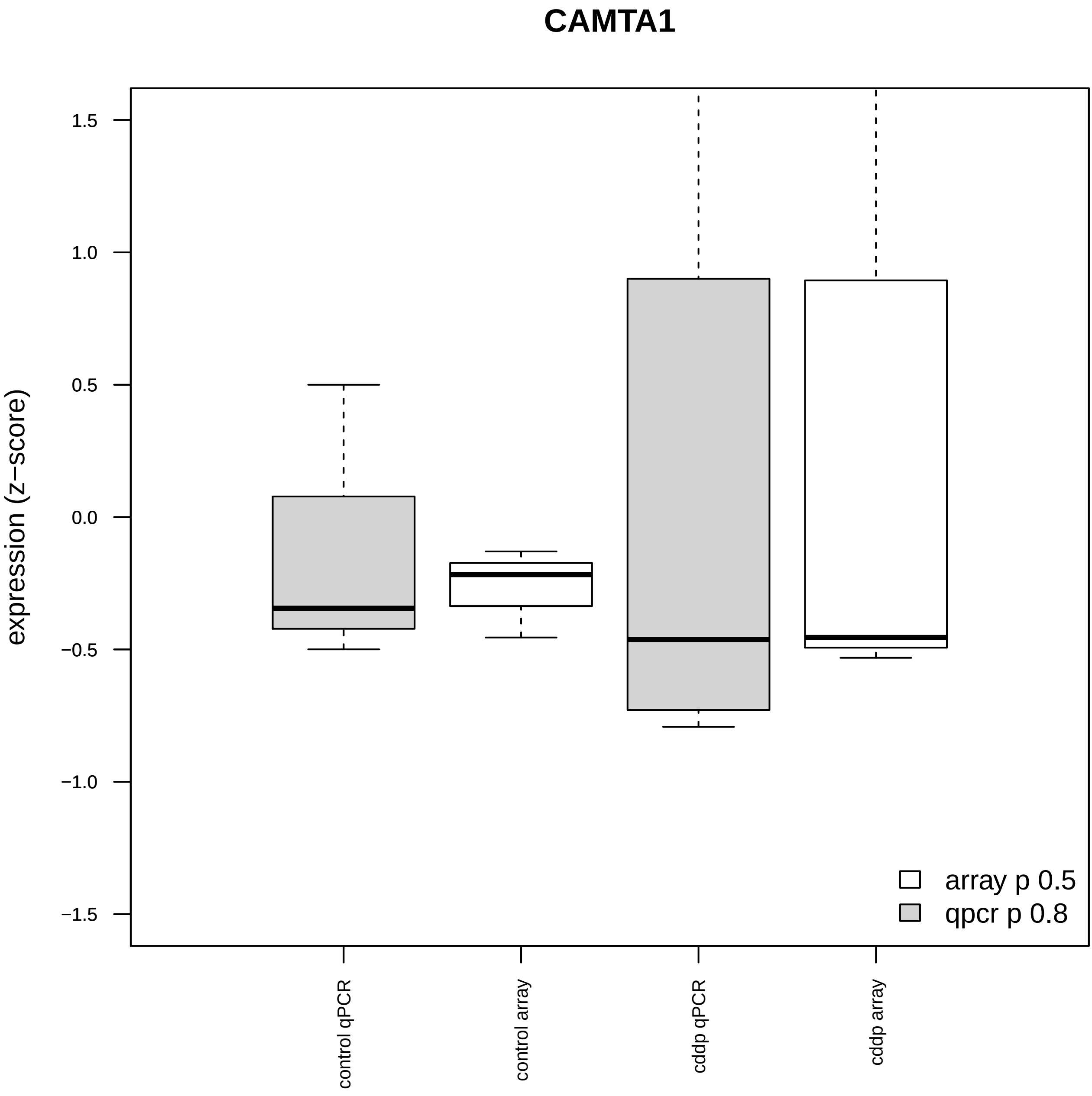

CAMTA1
expression (z−score)
1.5
1.0
0.5
0.0
−0.5
−1.0
−1.5
control qPCR
control array
cddp qPCR
cddp array
array p 0.5
qpcr p 0.8

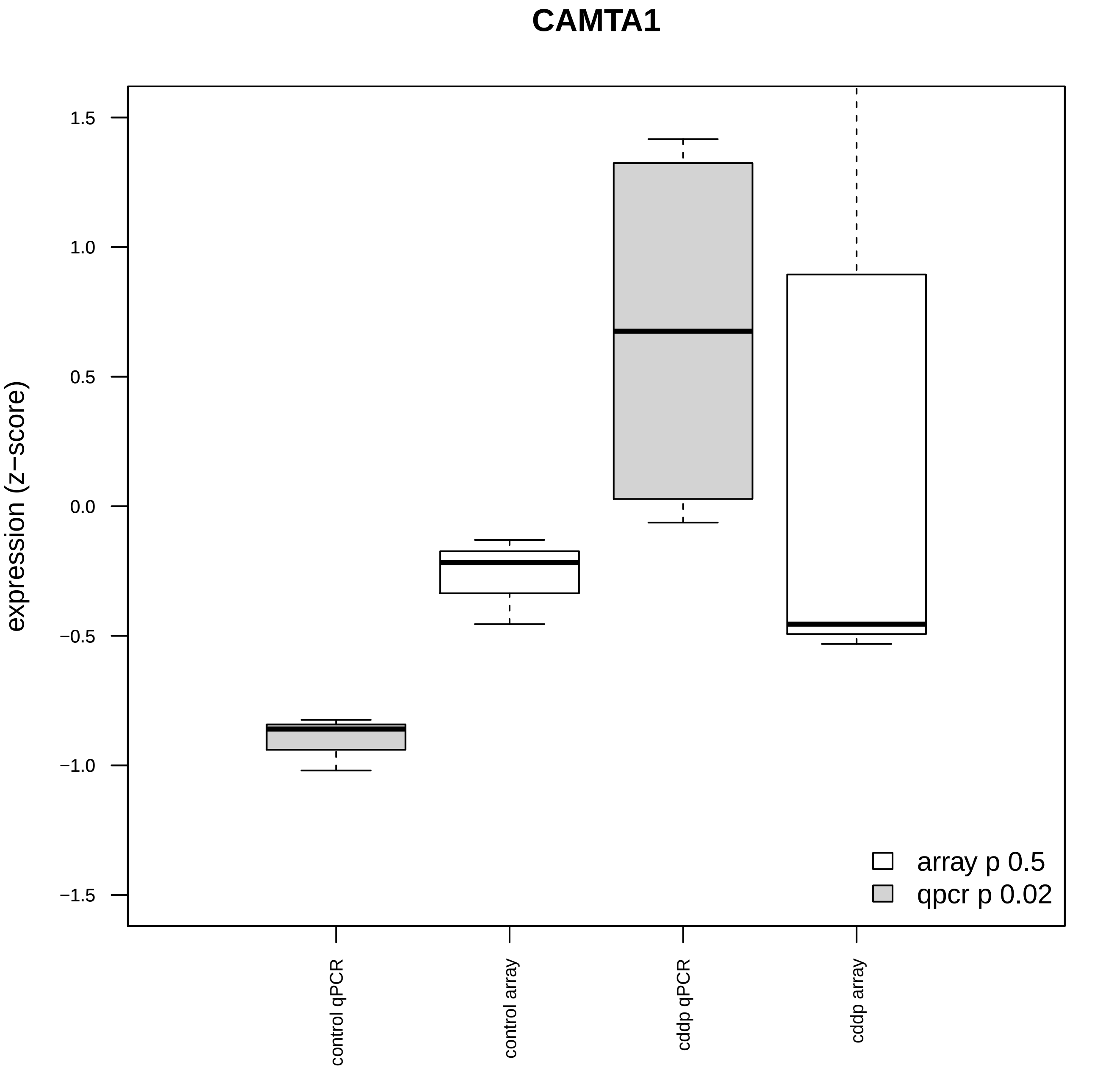

CAMTA1
expression (z−score)
1.5
1.0
0.5
0.0
−0.5
−1.0
−1.5
array p 0.5
qpcr p 0.02
control qPCR
control array
cddp qPCR
cddp array

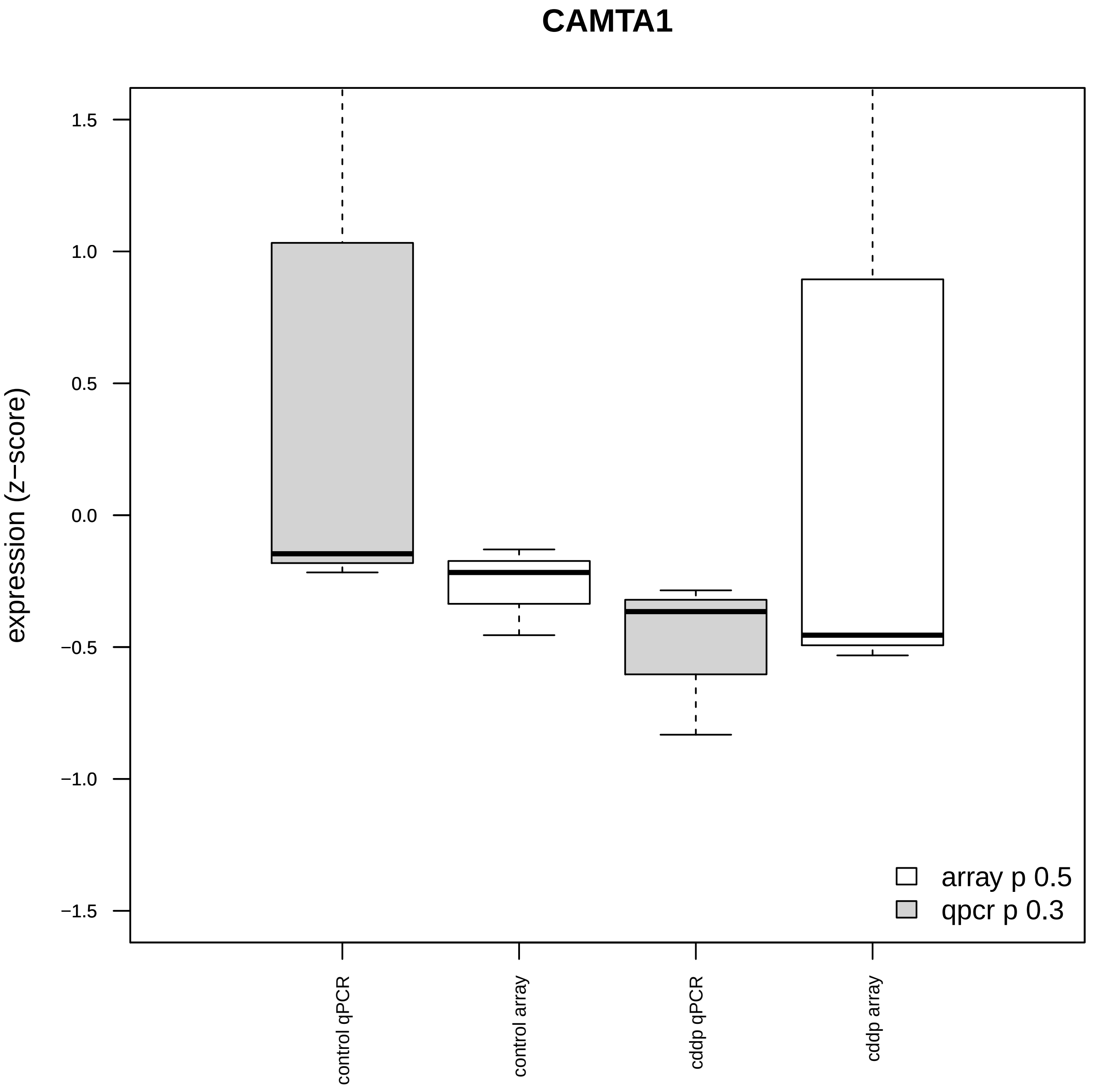
CAMTA1
expression (z−score)
1.5
1.0
0.5
0.0
−0.5
−1.0
−1.5
control qPCR
control array
cddp qPCR
cddp array
array p 0.5
qpcr p 0.3

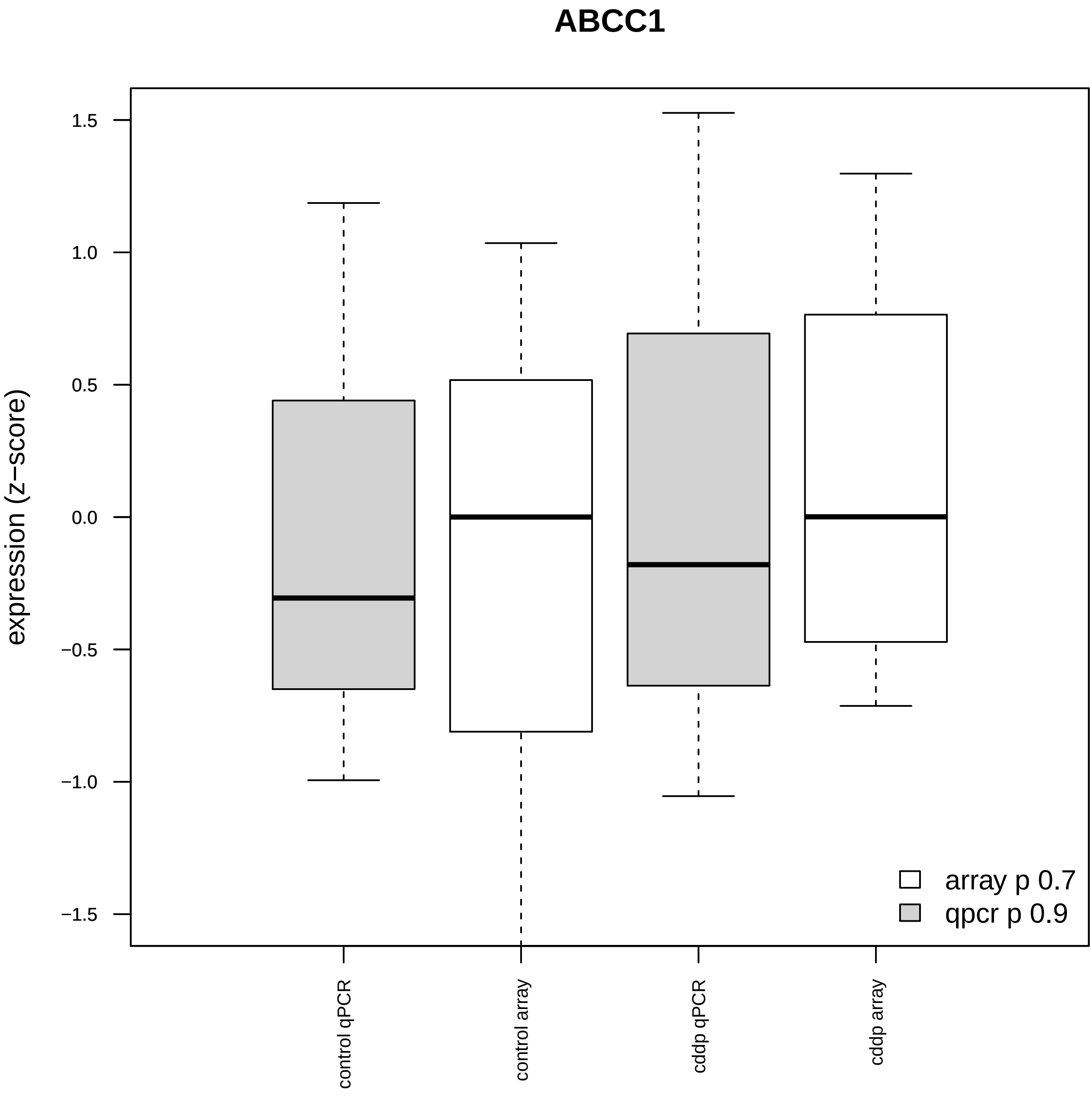
ABCC1
expression (z−score)
1.5
1.0
0.5
0.0
−0.5
−1.0
−1.5
control qPCR
control array
cddp qPCR
cddp array
array p 0.7
qpcr p 0.9

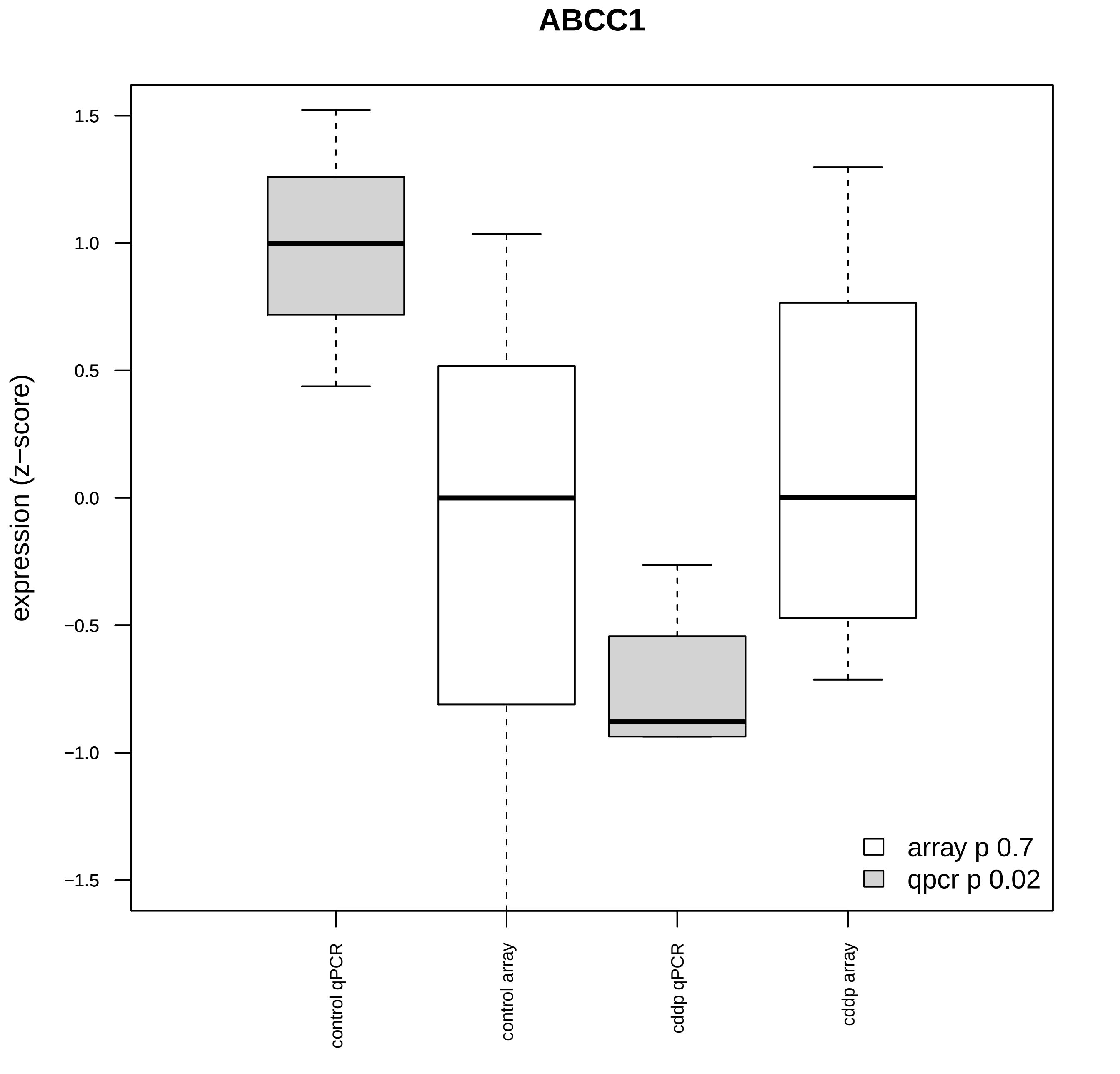
ABCC1
expression (z−score)
1.5
1.0
0.5
0.0
−0.5
−1.0
−1.5
control qPCR
control array
cddp qPCR
cddp array
array p 0.7
qpcr p 0.02

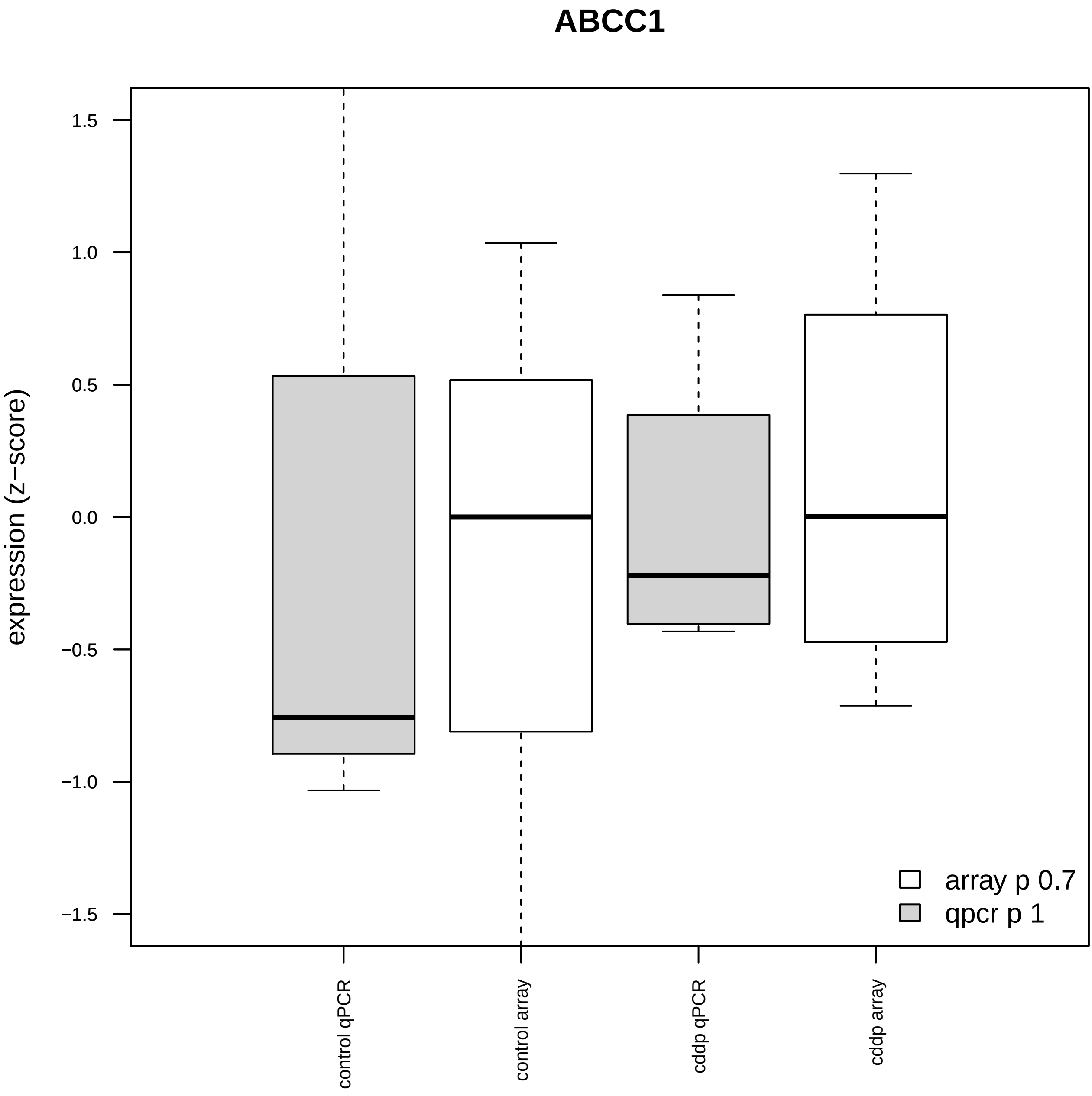
ABCC1
expression (z−score)
1.5
1.0
0.5
0.0
−0.5
−1.0
−1.5
array p 0.7
qpcr p 1
control qPCR
control array
cddp qPCR
cddp array

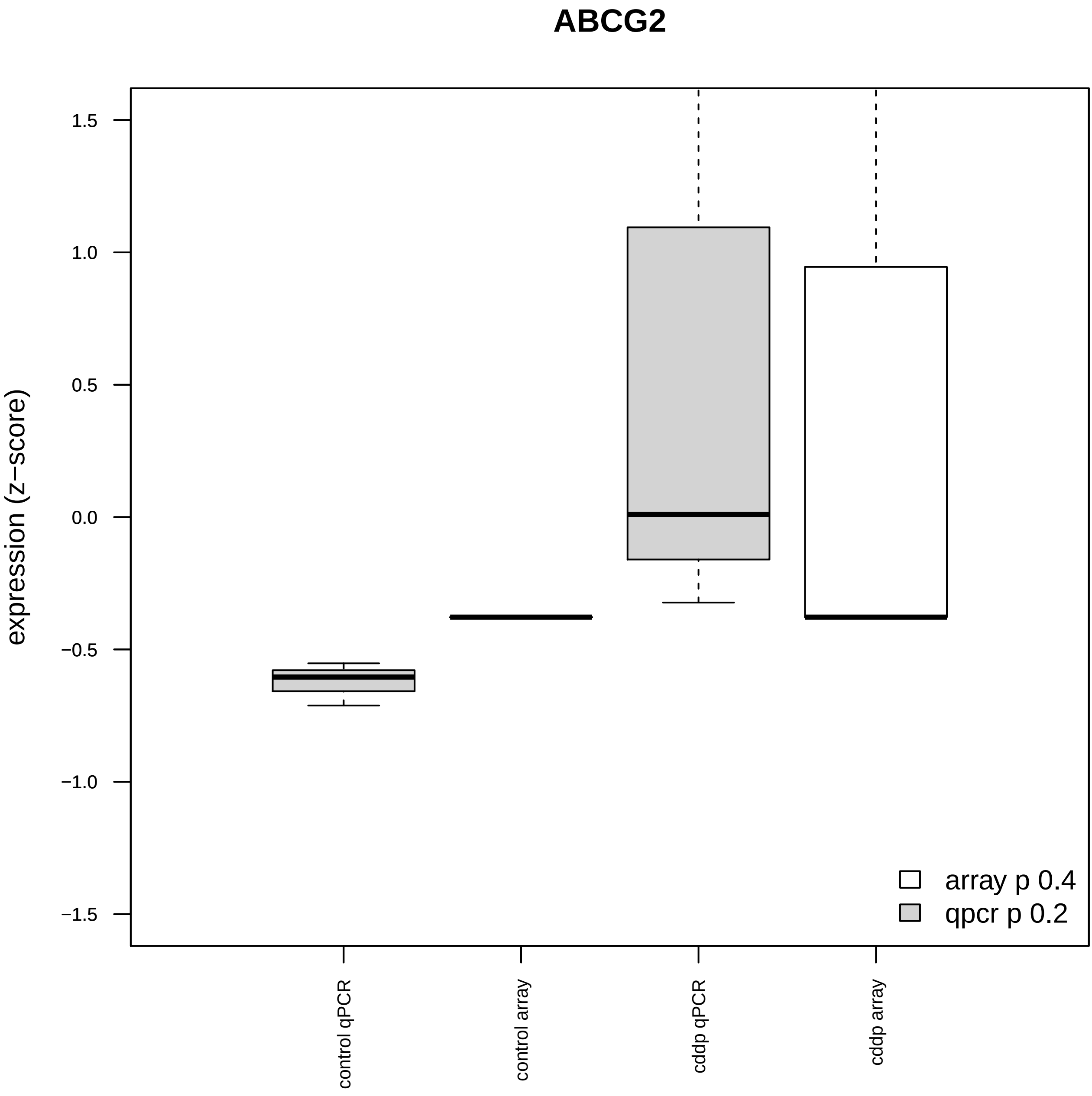
ABCG2
expression (z−score)
1.5
1.0
0.5
0.0
−0.5
−1.0
−1.5
array p 0.4
qpcr p 0.2
control qPCR
control array
cddp qPCR
cddp array

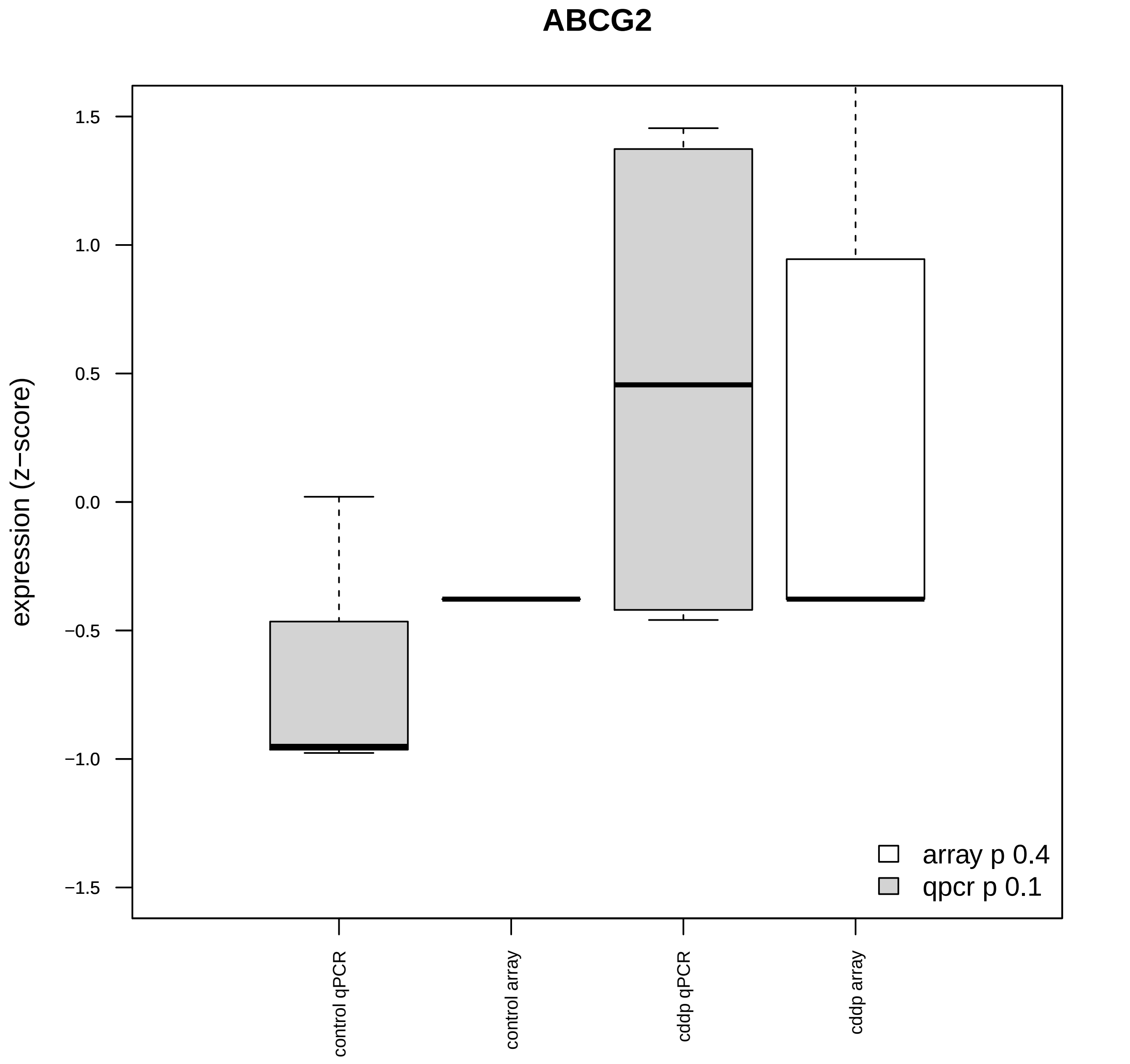

ABCG2
expression (z−score)
1.5
1.0
0.5
0.0
−0.5
−1.0
−1.5
array p 0.4
qpcr p 0.1
control qPCR
control array
cddp qPCR
cddp array

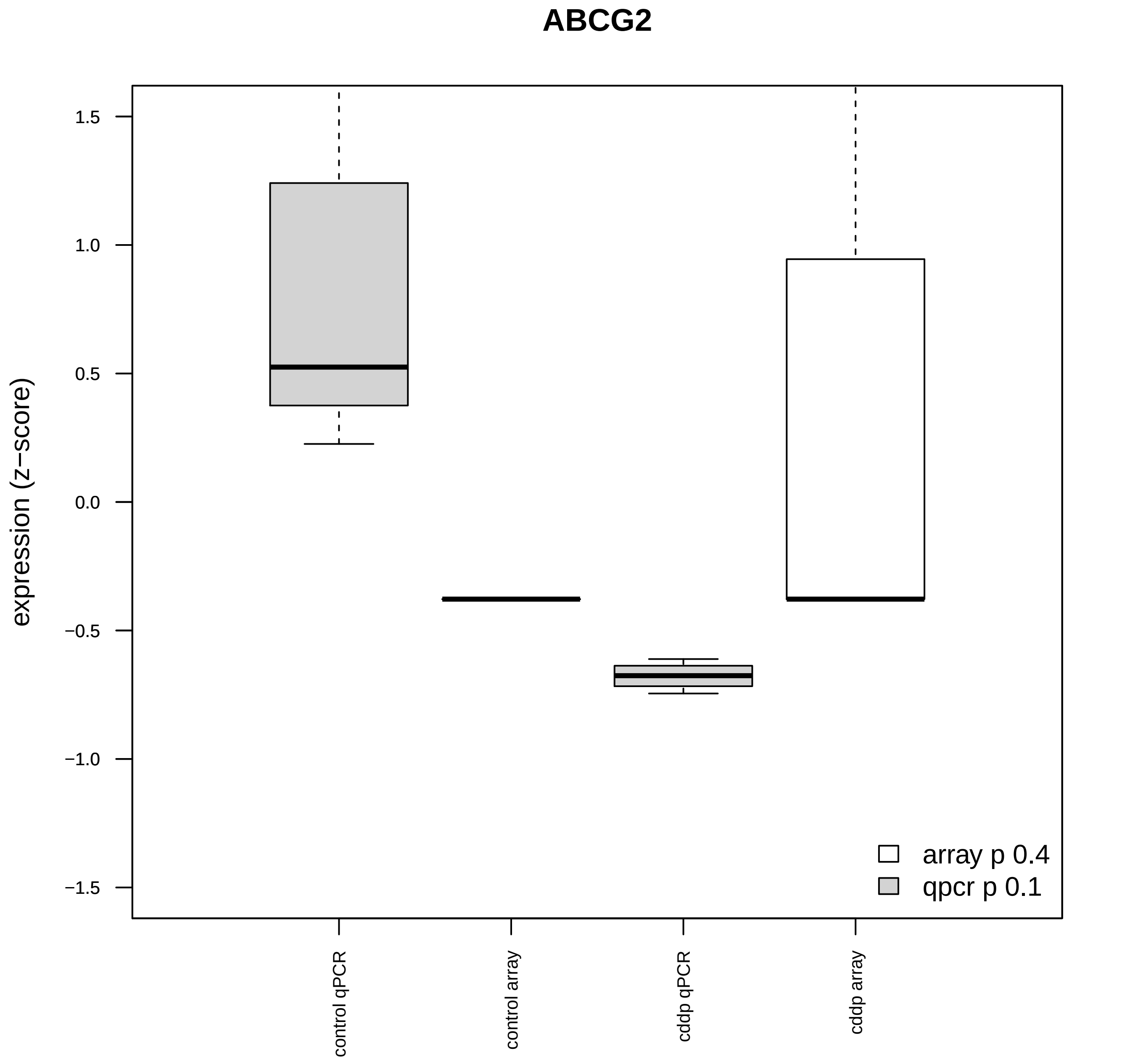
ABCG2
expression (z−score)
1.5
1.0
0.5
0.0
−0.5
−1.0
−1.5
control qPCR
control array
cddp qPCR
cddp array
array p 0.4
qpcr p 0.1

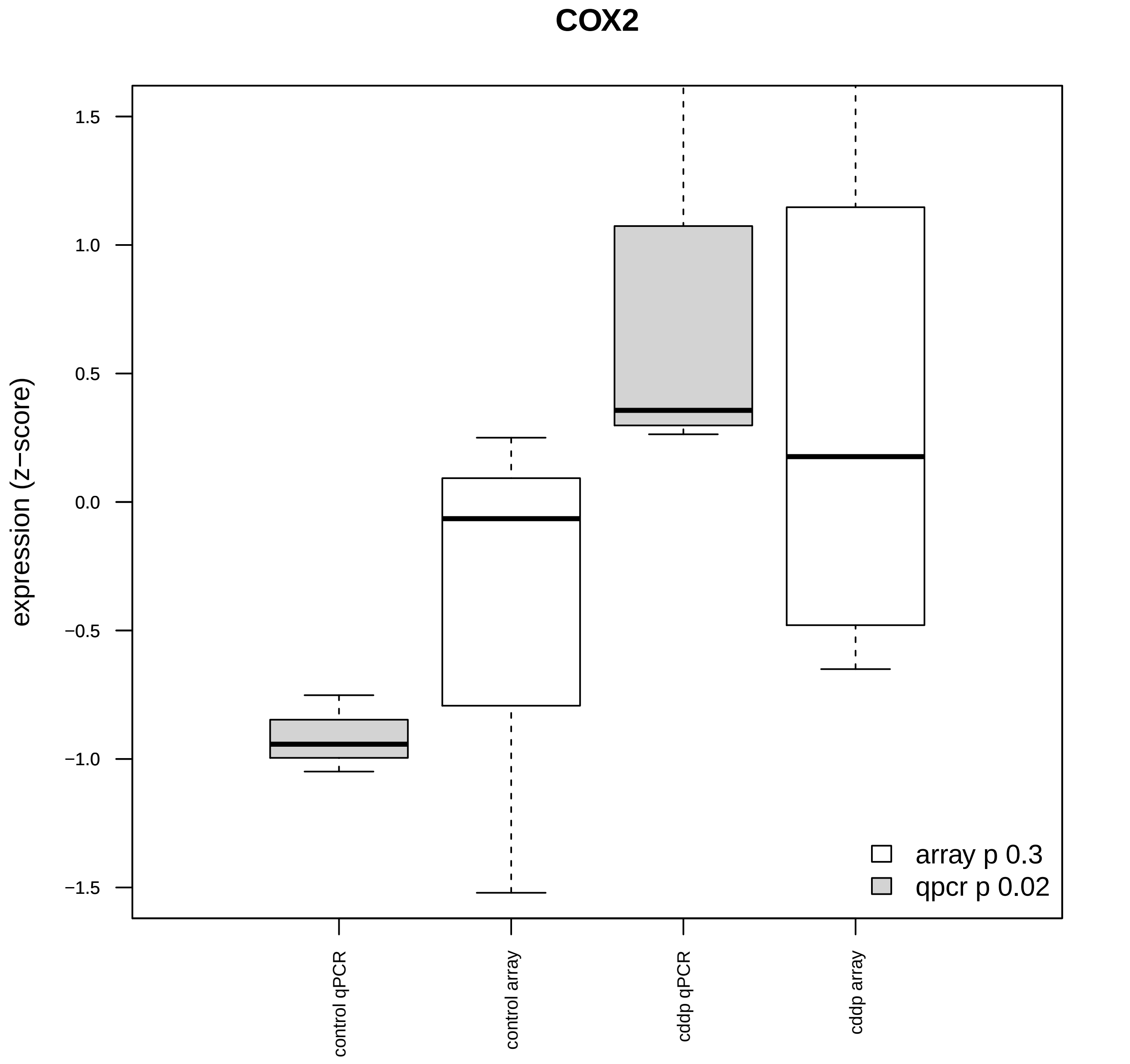
COX2
expression (z−score)
1.5
1.0
0.5
0.0
−0.5
−1.0
−1.5
control qPCR
control array
cddp qPCR
cddp array
array p 0.3
qpcr p 0.02

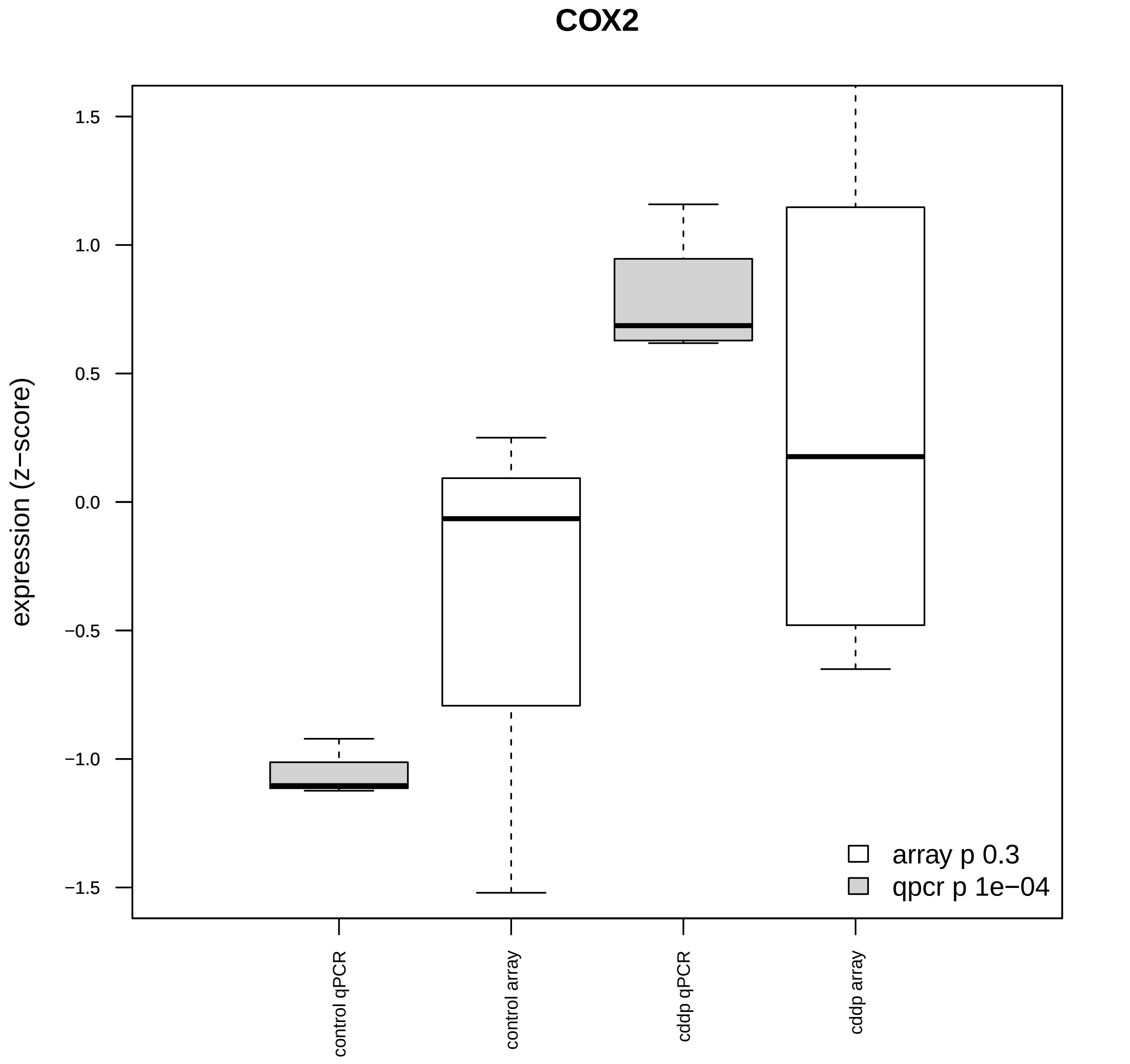

COX2
expression (z−score)
1.5
1.0
0.5
0.0
−0.5
−1.0
−1.5
control qPCR
control array
cddp qPCR
cddp array
array p 0.3
qpcr p 1e−04

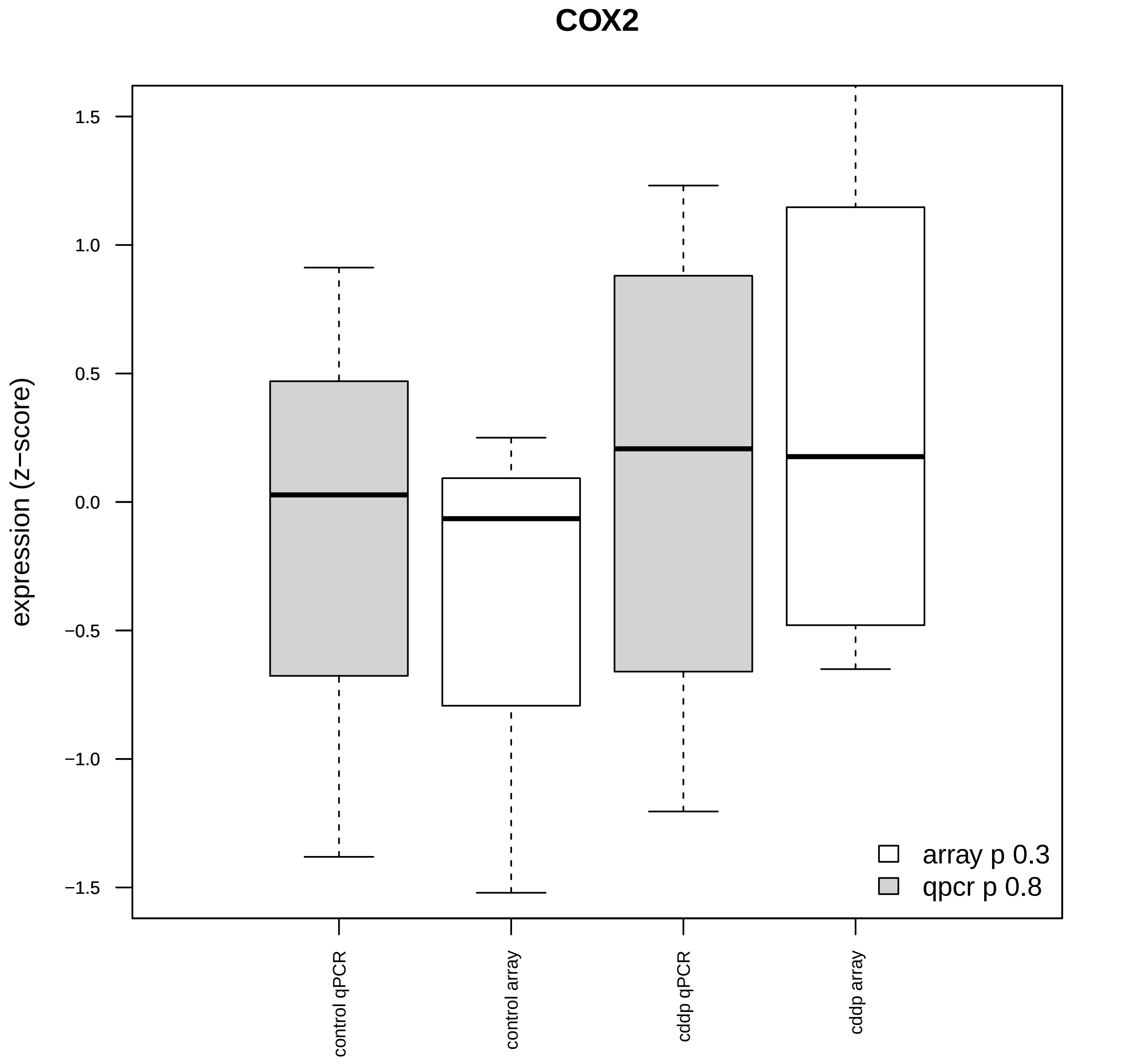

COX2
expression (z−score)
1.5
1.0
0.5
0.0
−0.5
−1.0
−1.5
array p 0.3
qpcr p 0.8
control qPCR
control array
cddp qPCR
cddp array

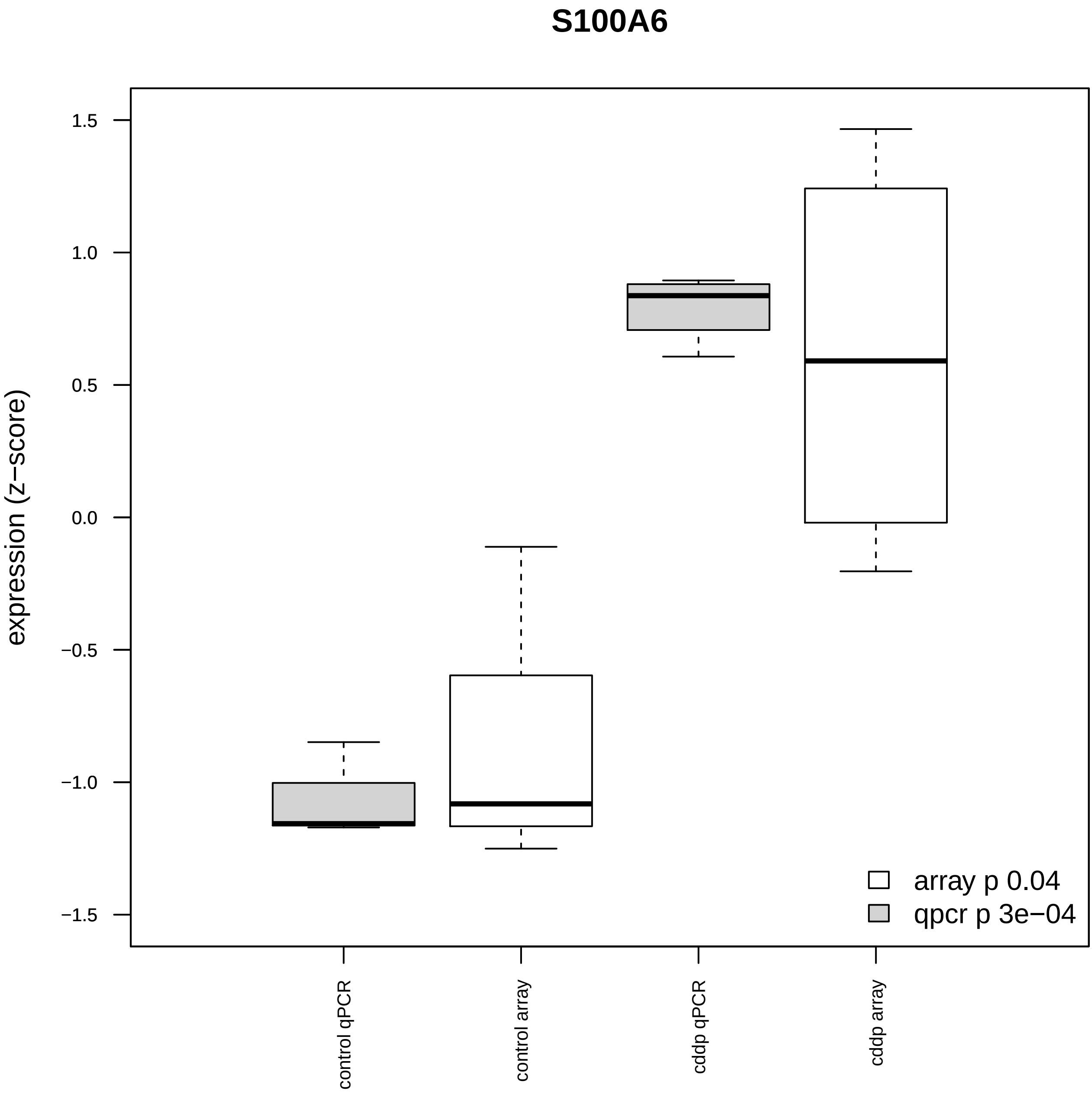

S100A6
expression (z−score)
1.5
1.0
0.5
0.0
−0.5
−1.0
−1.5
array p 0.04
qpcr p 3e−04
control qPCR
control array
cddp qPCR
cddp array

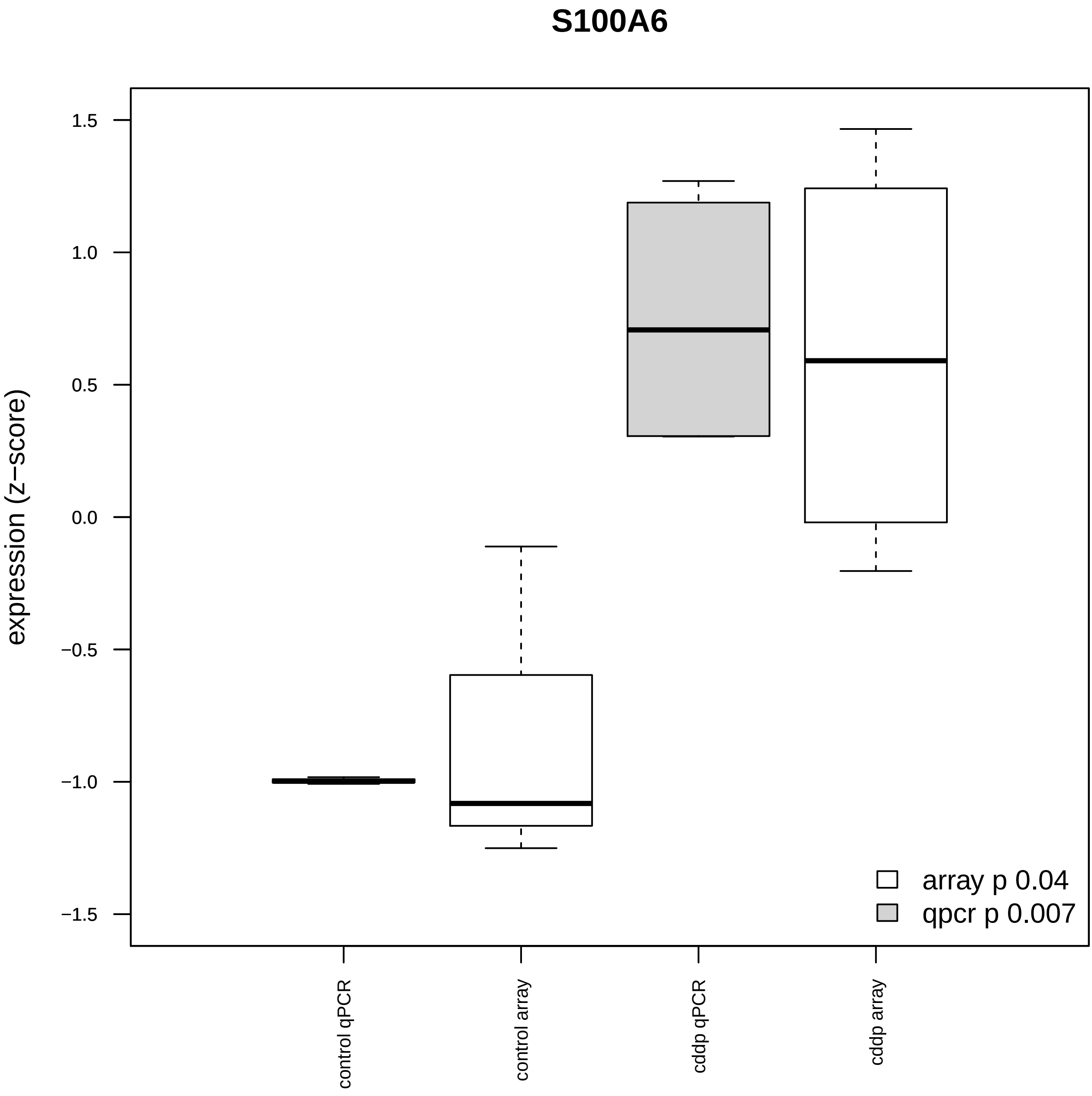
S100A6
expression (z−score)
1.5
1.0
0.5
0.0
−0.5
−1.0
−1.5
control qPCR
control array
cddp qPCR
cddp array
array p 0.04
qpcr p 0.007

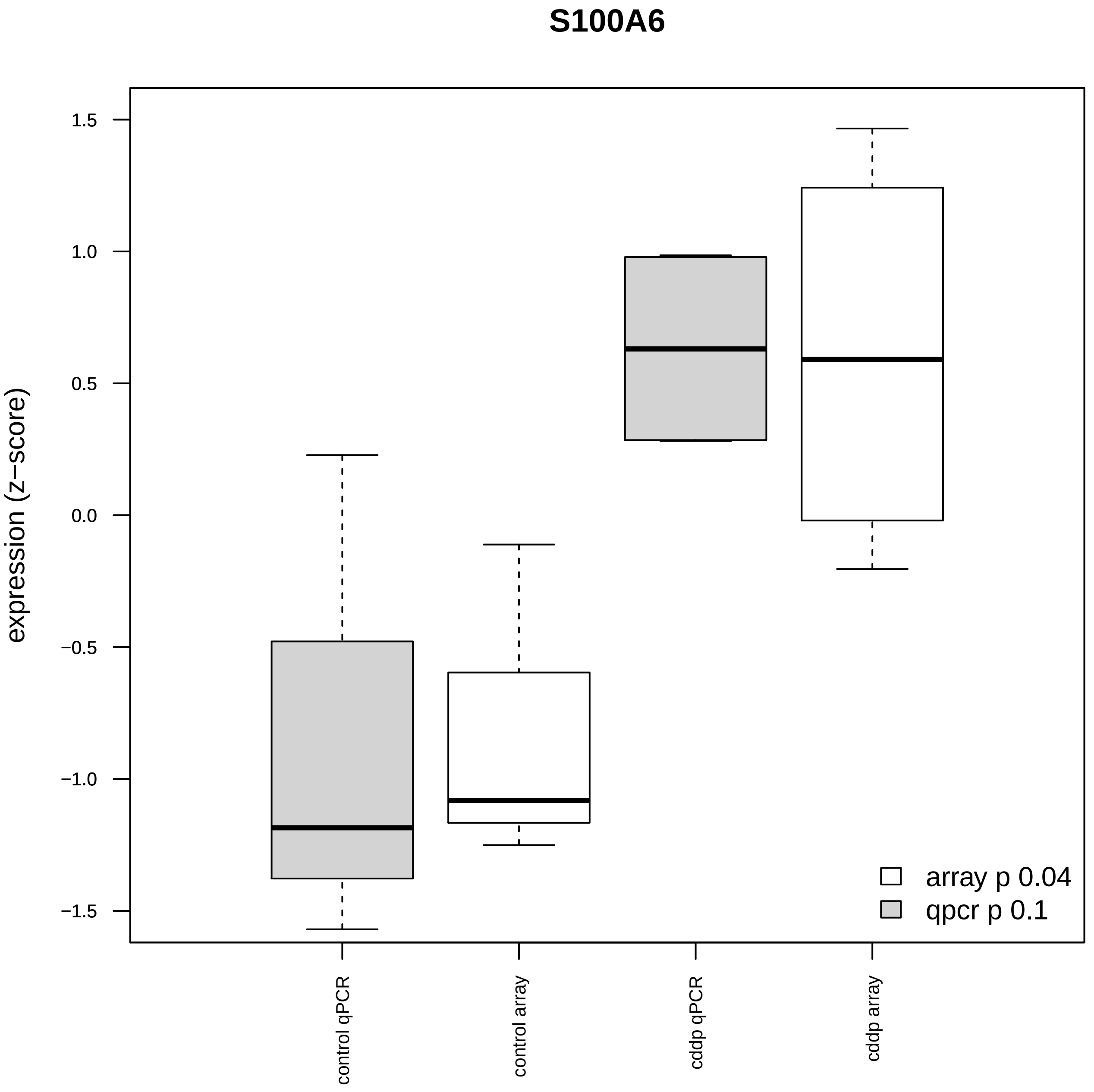
S100A6
expression (z−score)
1.5
1.0
0.5
0.0
−0.5
−1.0
−1.5
control qPCR
control array
cddp qPCR
cddp array
array p 0.04
qpcr p 0.1

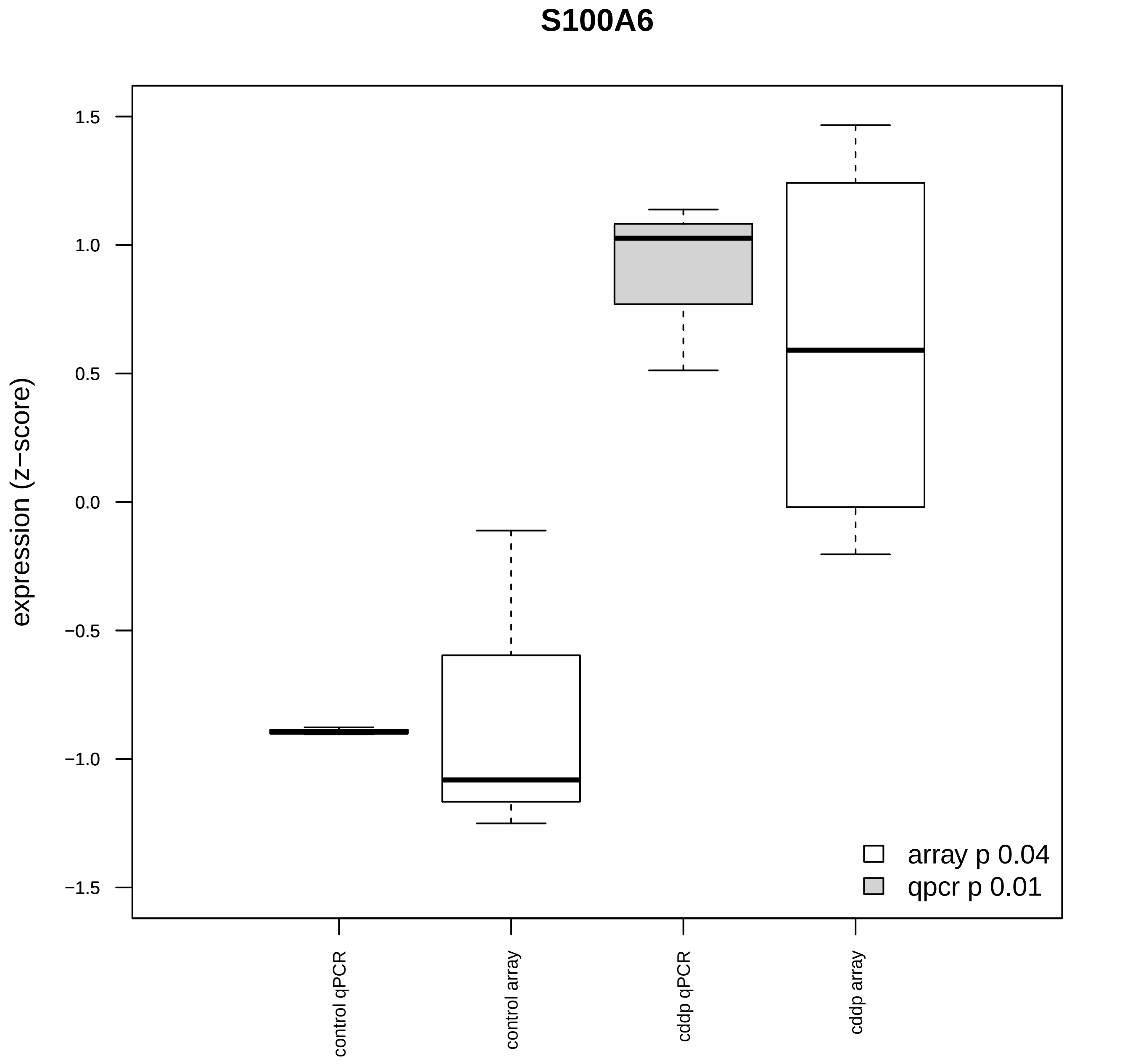
S100A6
expression (z−score)
1.5
1.0
0.5
0.0
−0.5
−1.0
−1.5
array p 0.04
qpcr p 0.01
control qPCR
control array
cddp qPCR
cddp array

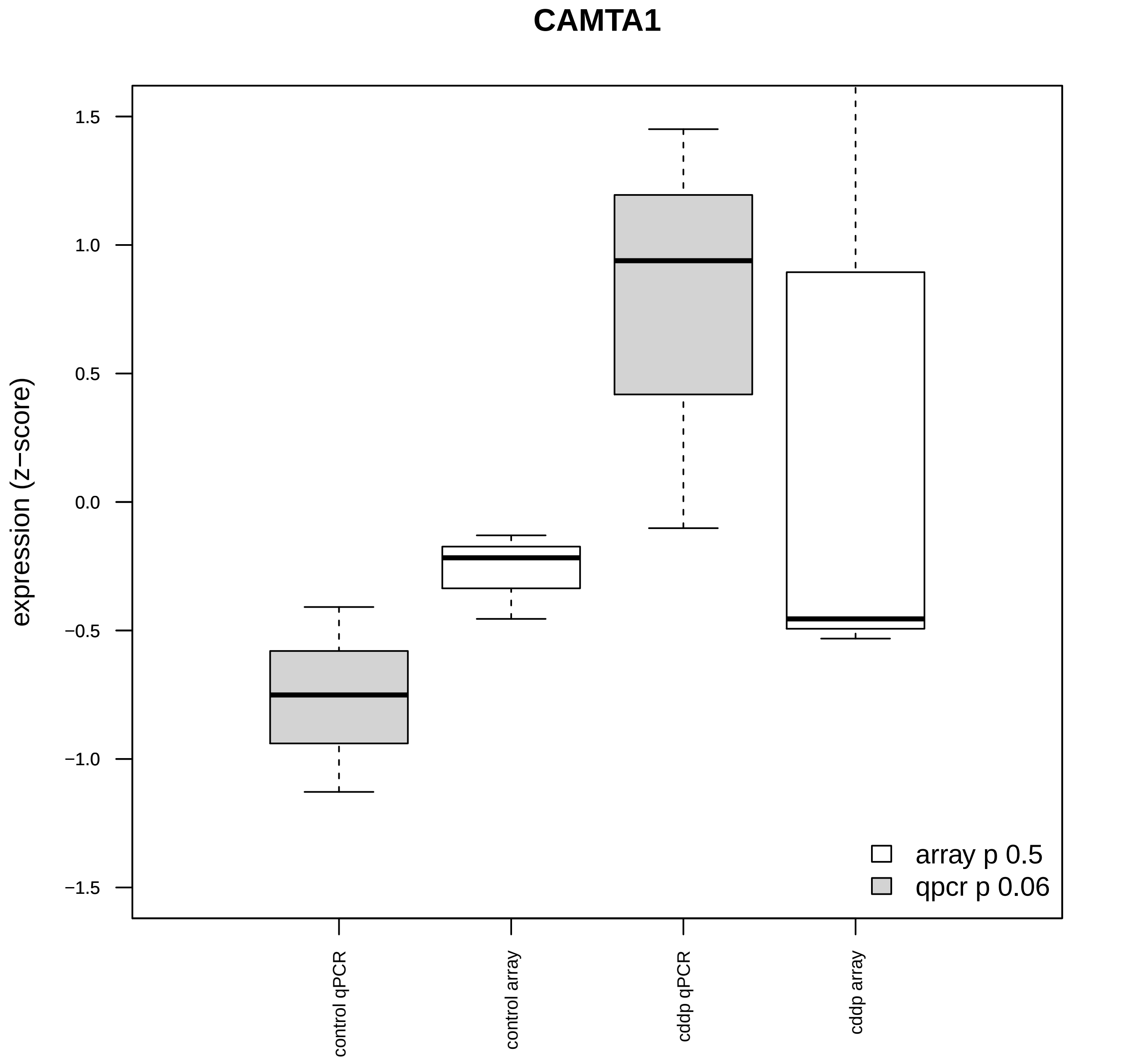

CAMTA1
expression (z−score)
1.5
1.0
0.5
0.0
−0.5
−1.0
−1.5
control qPCR
control array
cddp qPCR
cddp array
array p 0.5
qpcr p 0.06

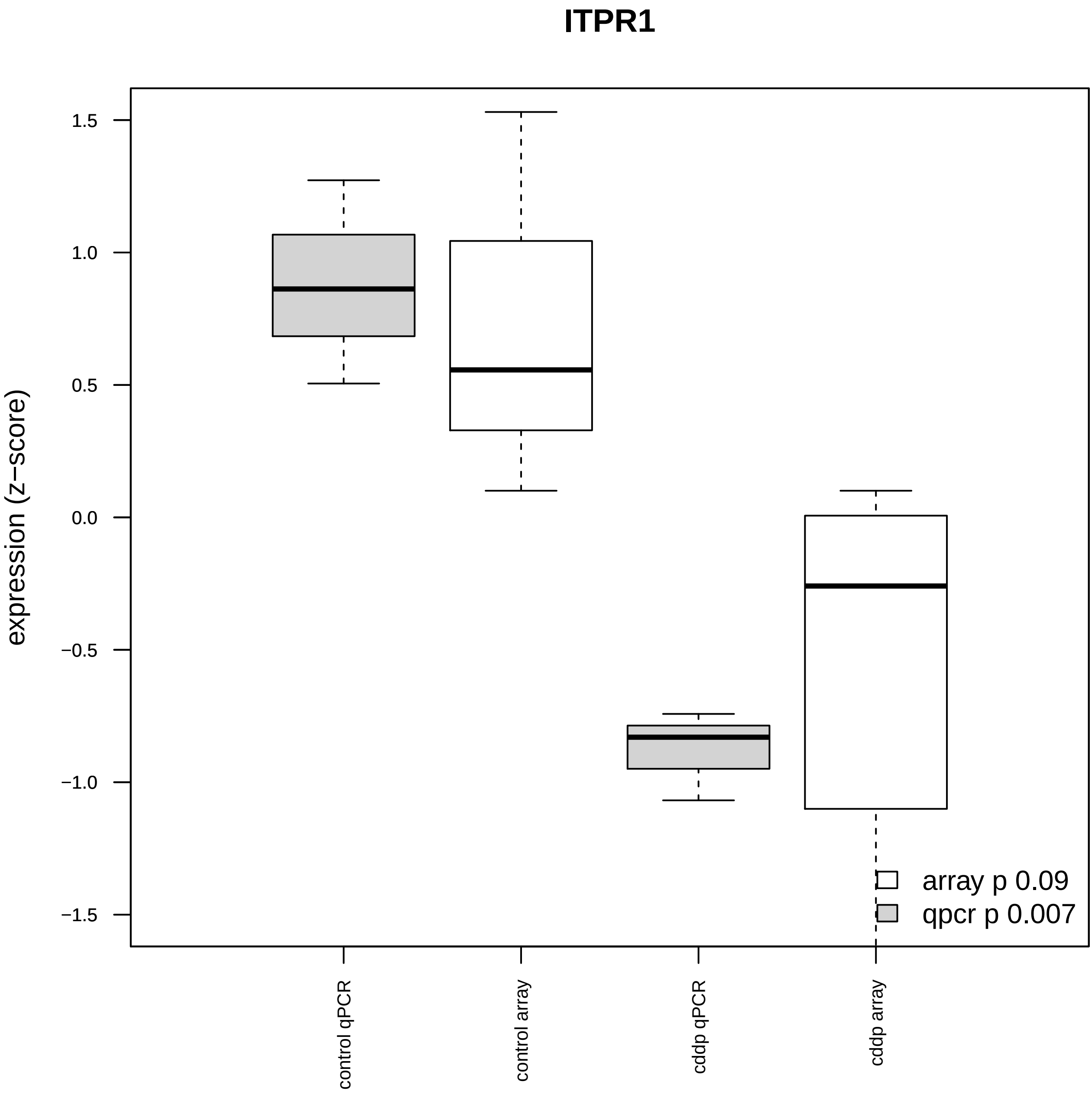
ITPR1
expression (z−score)
1.5
1.0
0.5
0.0
−0.5
−1.0
−1.5
control qPCR
control array
cddp qPCR
cddp array
array p 0.09
qpcr p 0.007

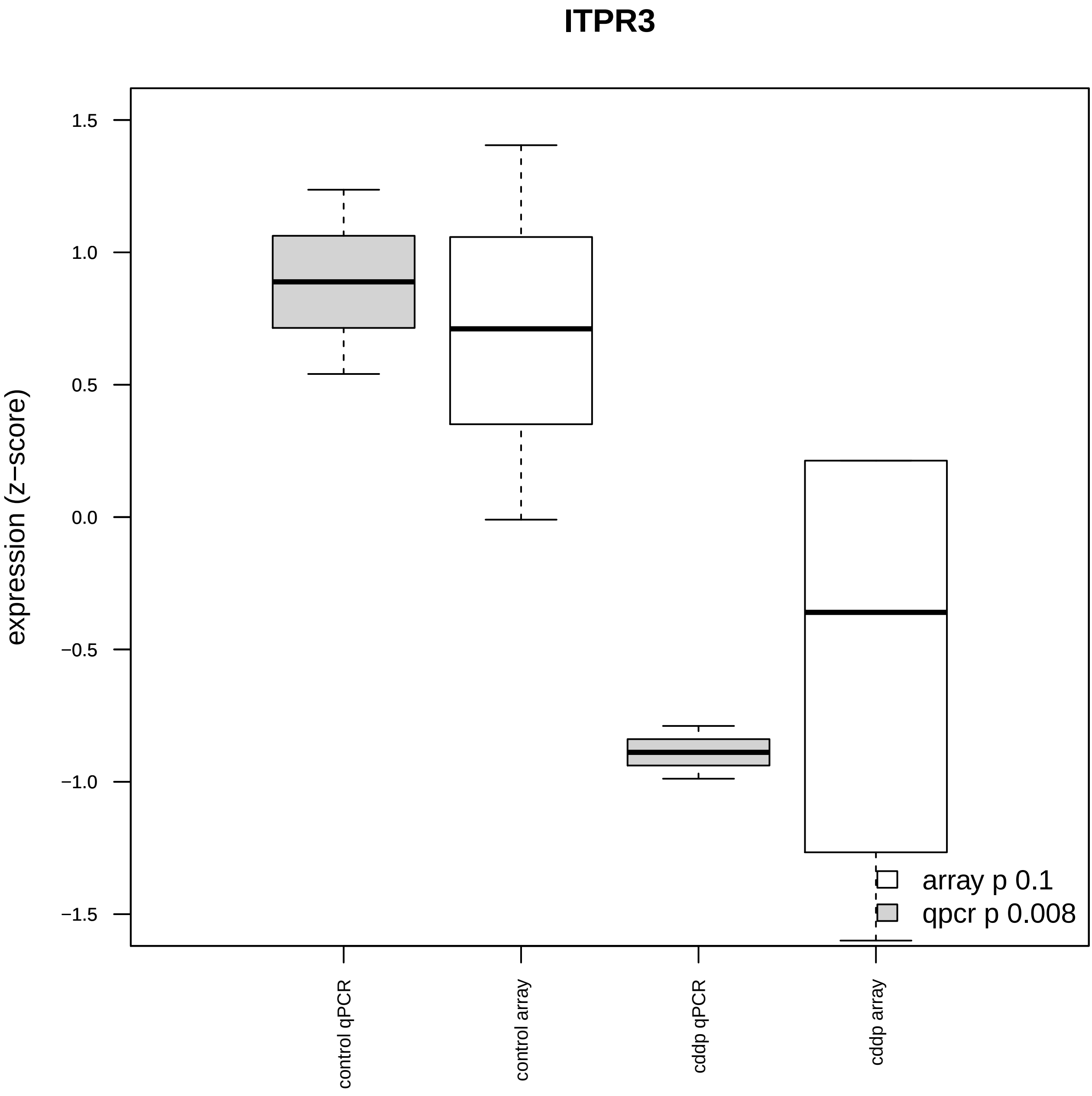
ITPR3
expression (z−score)
1.5
1.0
0.5
0.0
−0.5
−1.0
−1.5
control qPCR
control array
cddp qPCR
cddp array
array p 0.1
qpcr p 0.008

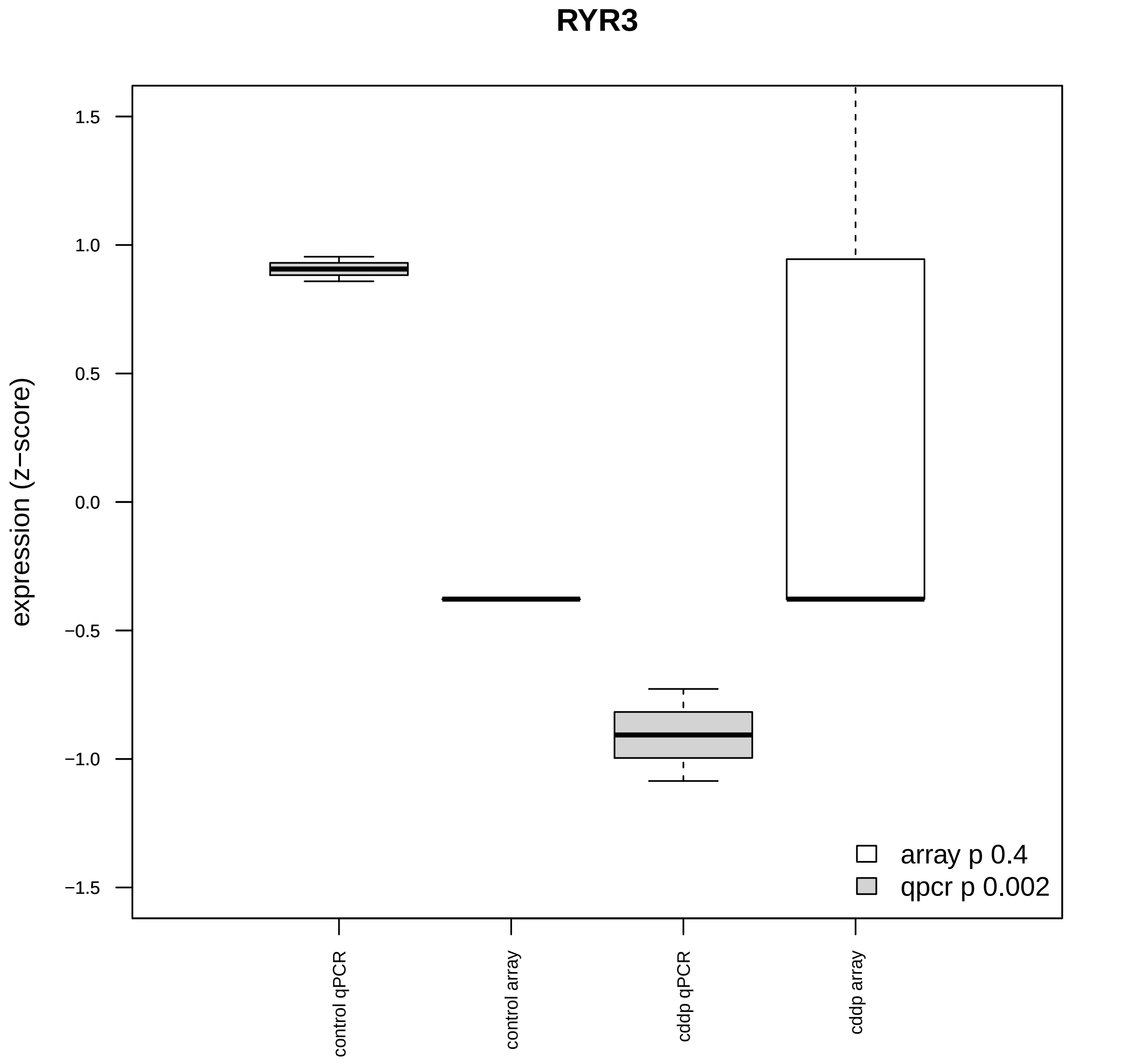
RYR3
expression (z−score)
1.5
1.0
0.5
0.0
−0.5
−1.0
−1.5
control qPCR
control array
cddp qPCR
cddp array
array p 0.4
qpcr p 0.002

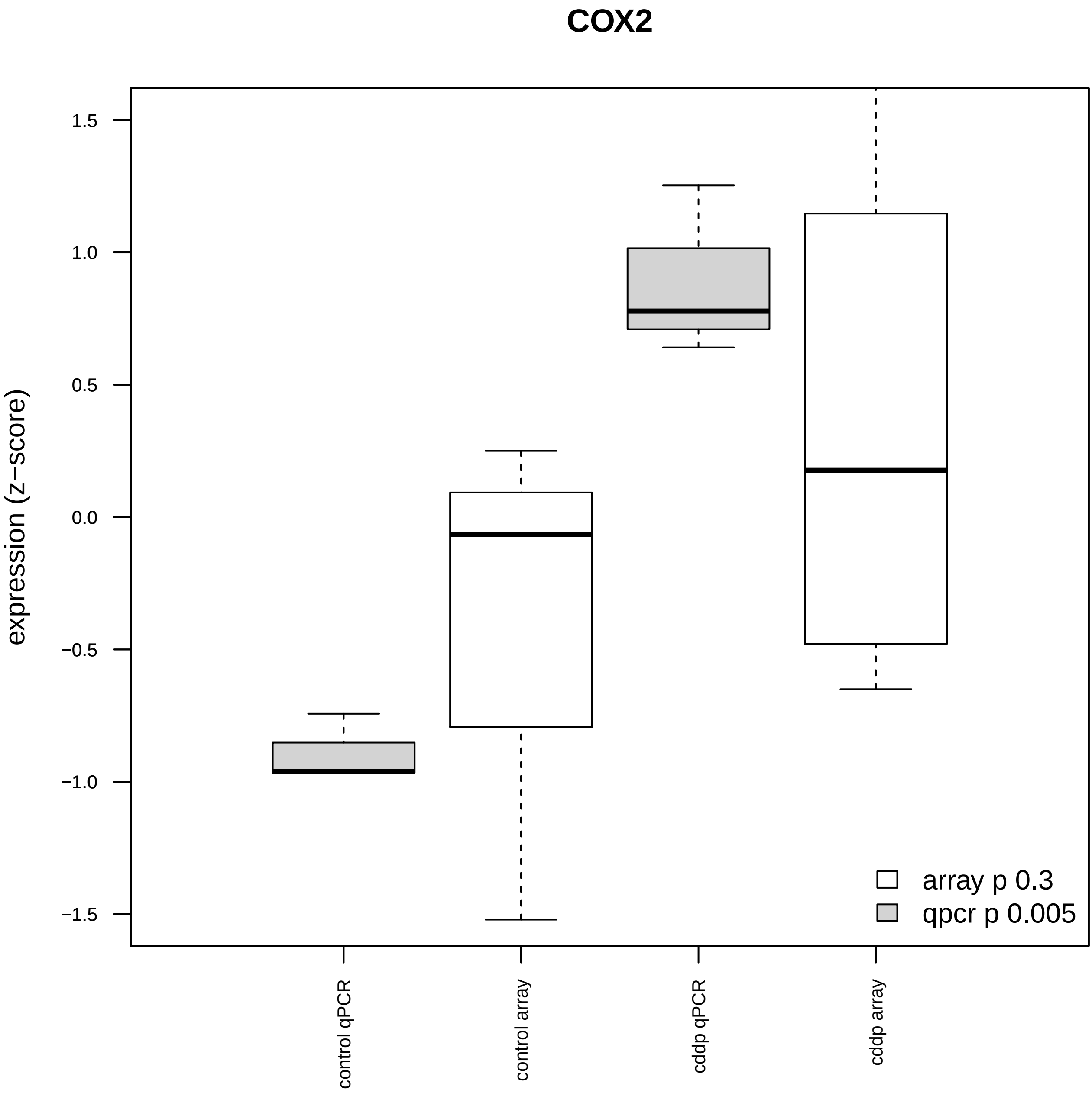
COX2
expression (z−score)
1.5
1.0
0.5
0.0
−0.5
−1.0
−1.5
control qPCR
control array
cddp qPCR
cddp array
array p 0.3
qpcr p 0.005
