# Supplementary material for: Calcium-regulatory proteins as modulators of chemotherapy in human neuroblastoma
Source: Oncotarget. 2017 Feb 11;8(14):22876–93. doi: 10.18632/oncotarget.15283 (PMC5410270; doi:10.18632/oncotarget.15283)
Supplement: Supplementary file 6 [file oncotarget-08-22876-s006.pdf]

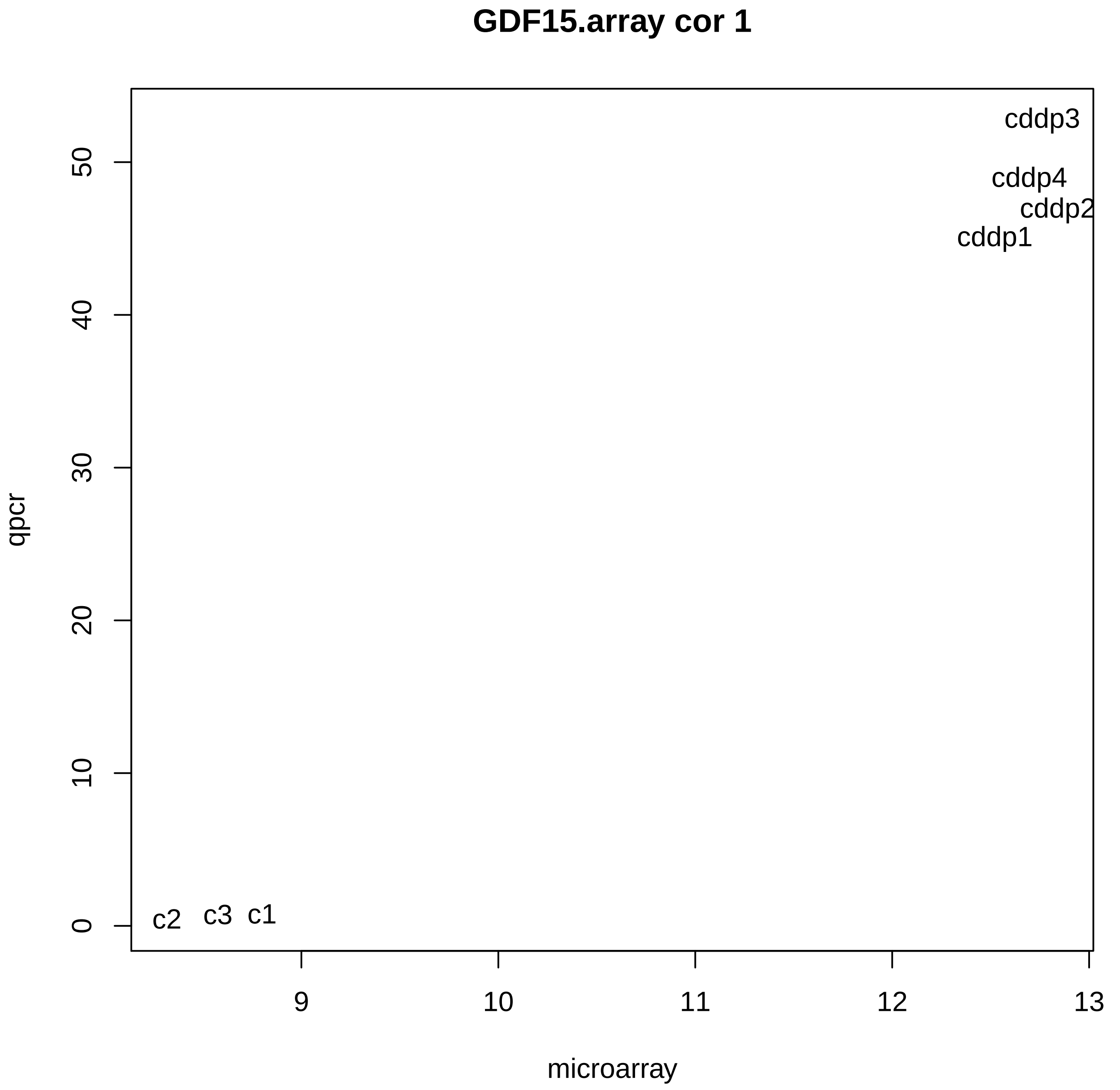
GDF15.array cor 1
cddp3
cddp4
cddp2
cddp1
c2
c3
c1
qpcr
0
10
20
30
40
50
9
10
11
12
13
microarray

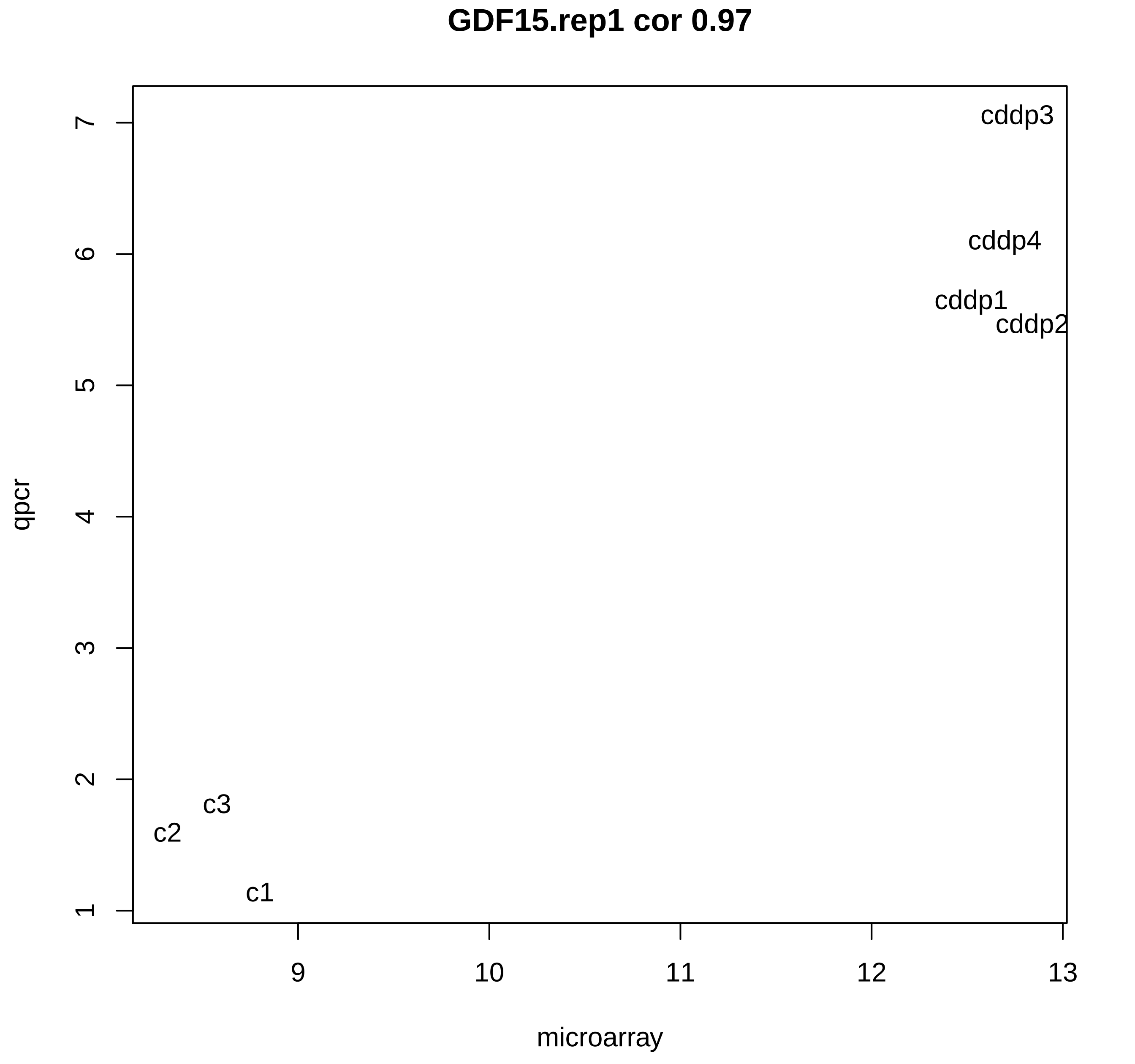
GDF15.rep1 cor 0.97
cddp3
cddp4
cddp1
cddp2
c3
c2
c1
qpcr
microarray
1
2
3
4
5
6
7
9
10
11
12
13

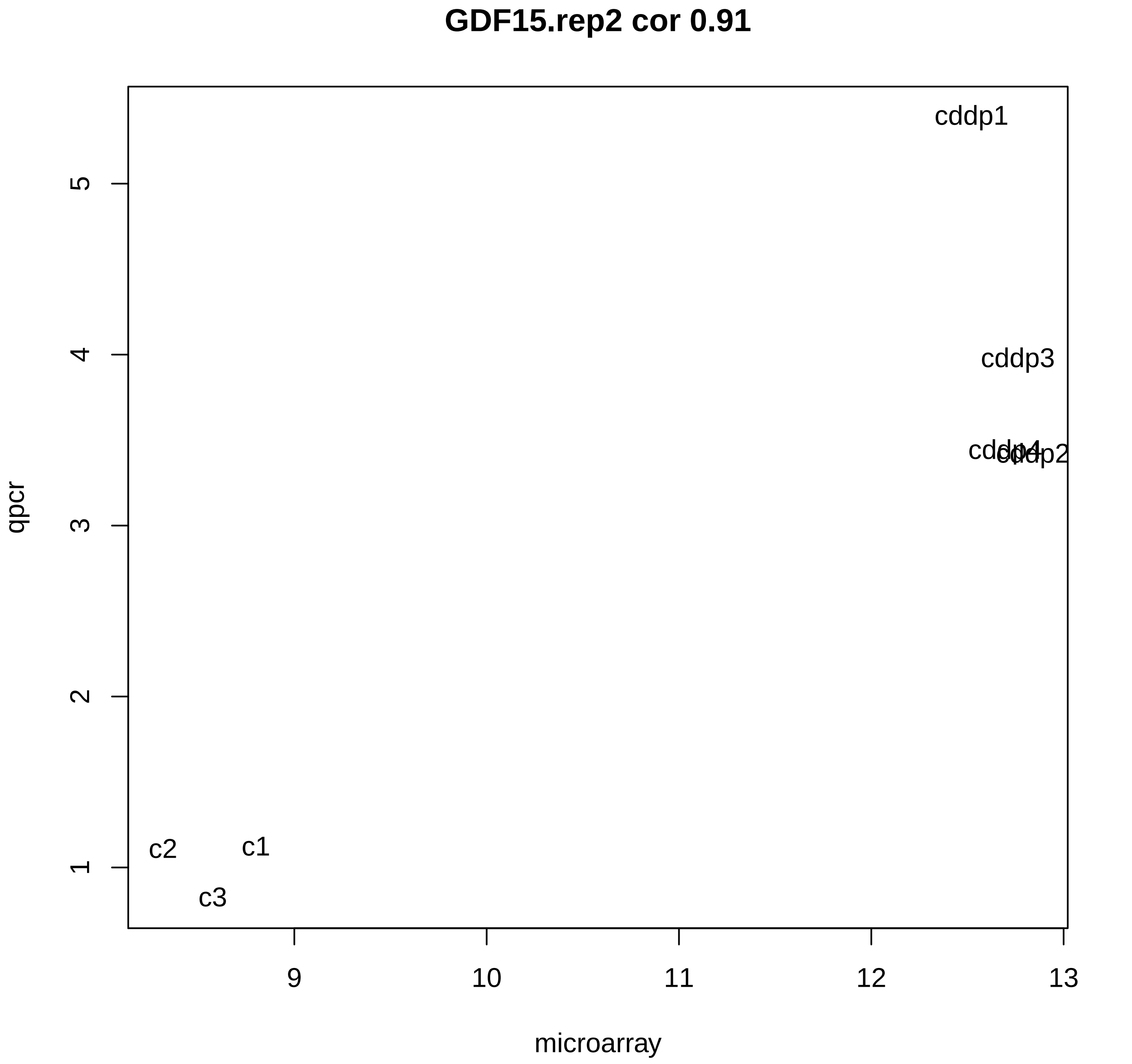

GDF15.rep2 cor 0.91
cddp1
cddp3
cddp4
cddp2
c2
c1
c3
5
4
3
2
1
qpcr
9
10
11
12
13
microarray

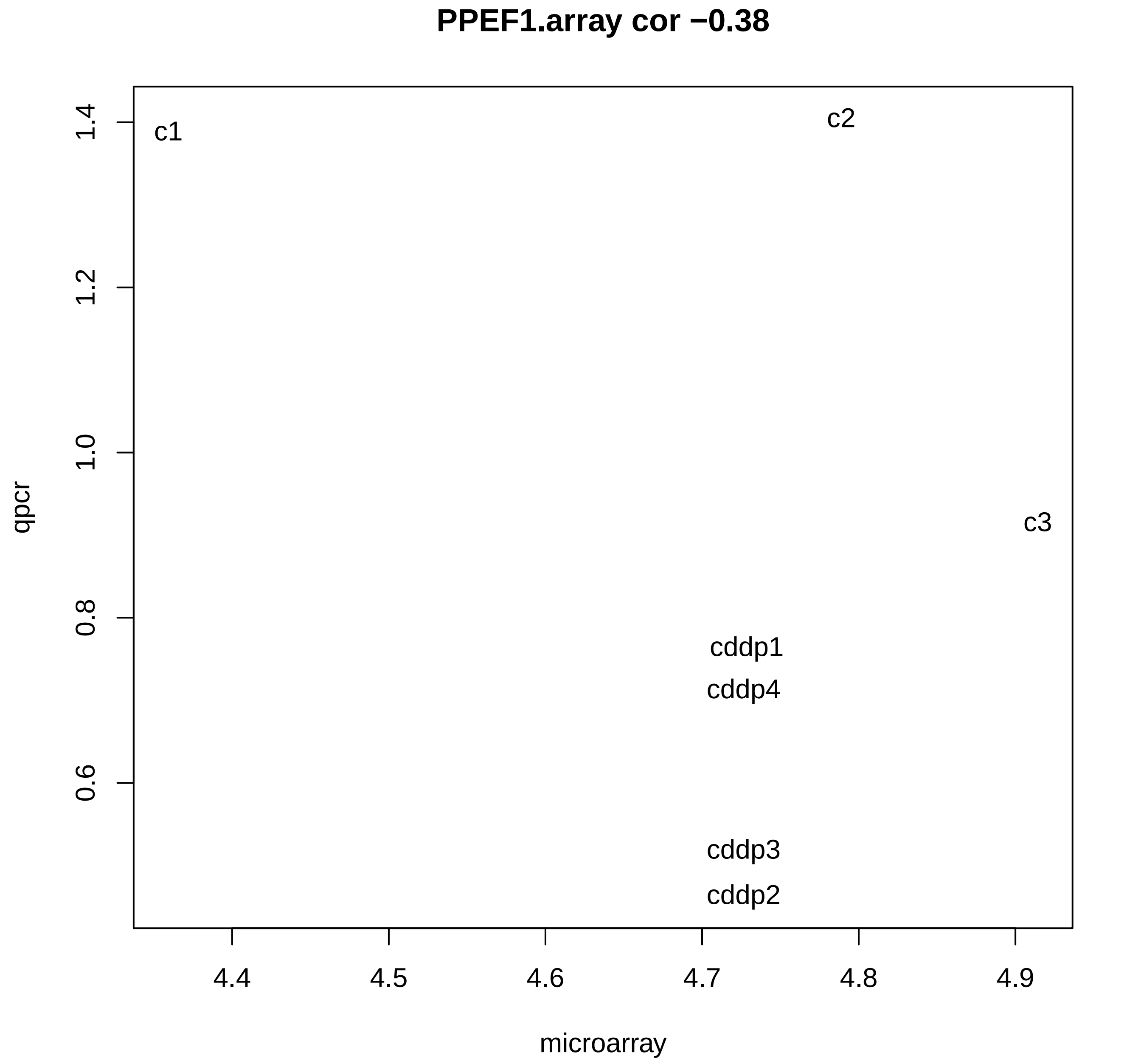
PPEF1.array cor −0.38
qpcr
microarray
1.4
1.2
1.0
0.8
0.6
4.4
4.5
4.6
4.7
4.8
4.9
c1
c2
c3
cddp1
cddp4
cddp3
cddp2

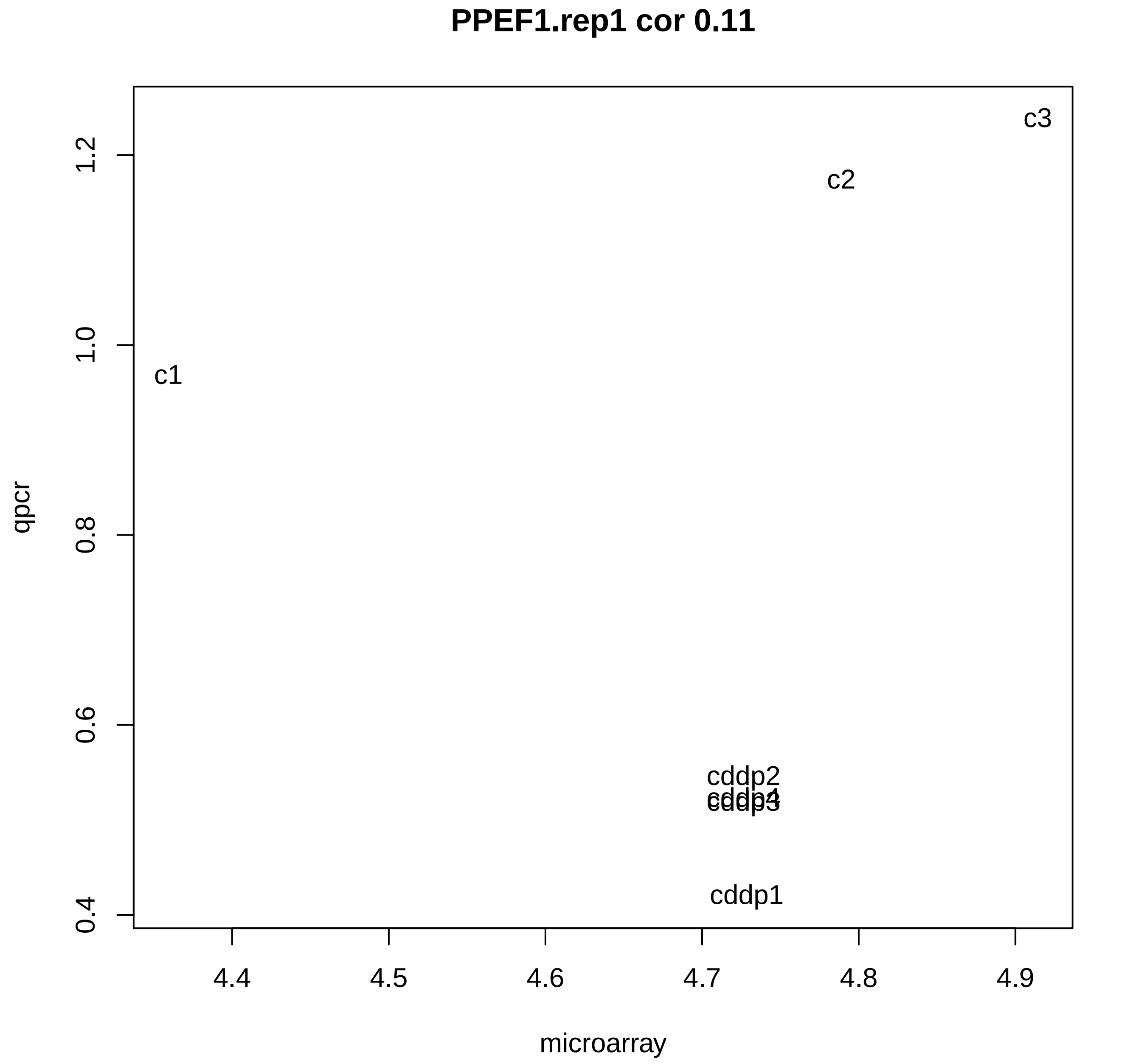
PPEF1.rep1 cor 0.11
qpcr
microarray
1.2
1.0
0.8
0.6
0.4
4.4
4.5
4.6
4.7
4.8
4.9
c1
c2
c3
cddp1
cddp2
cddp3
cddp4

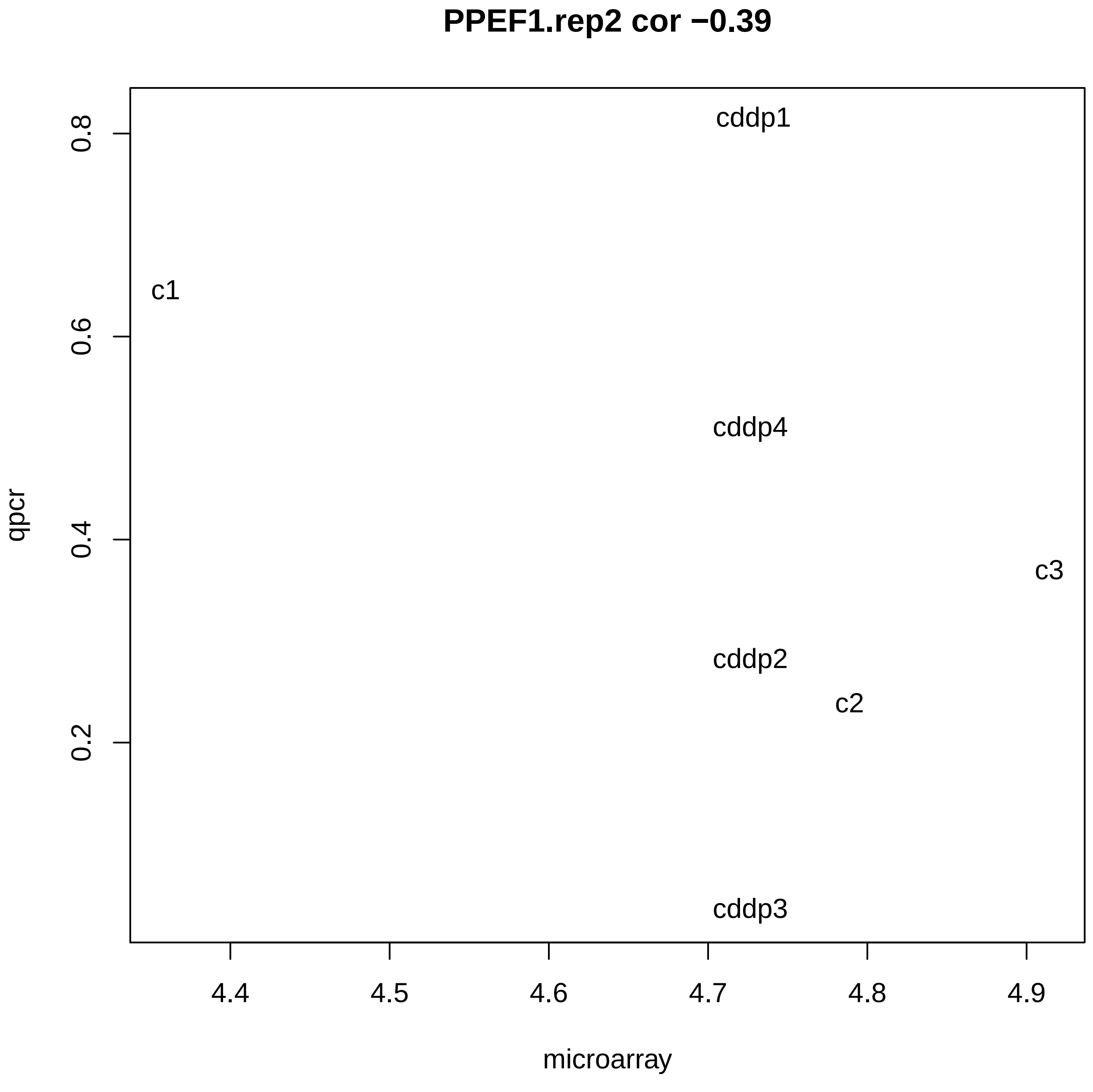
PPEF1.rep2 cor −0.39
cddp1
c1
cddp4
c3
cddp2
c2
cddp3
0.2
0.4
0.6
0.8
qpcr
4.4
4.5
4.6
4.7
4.8
4.9
microarray

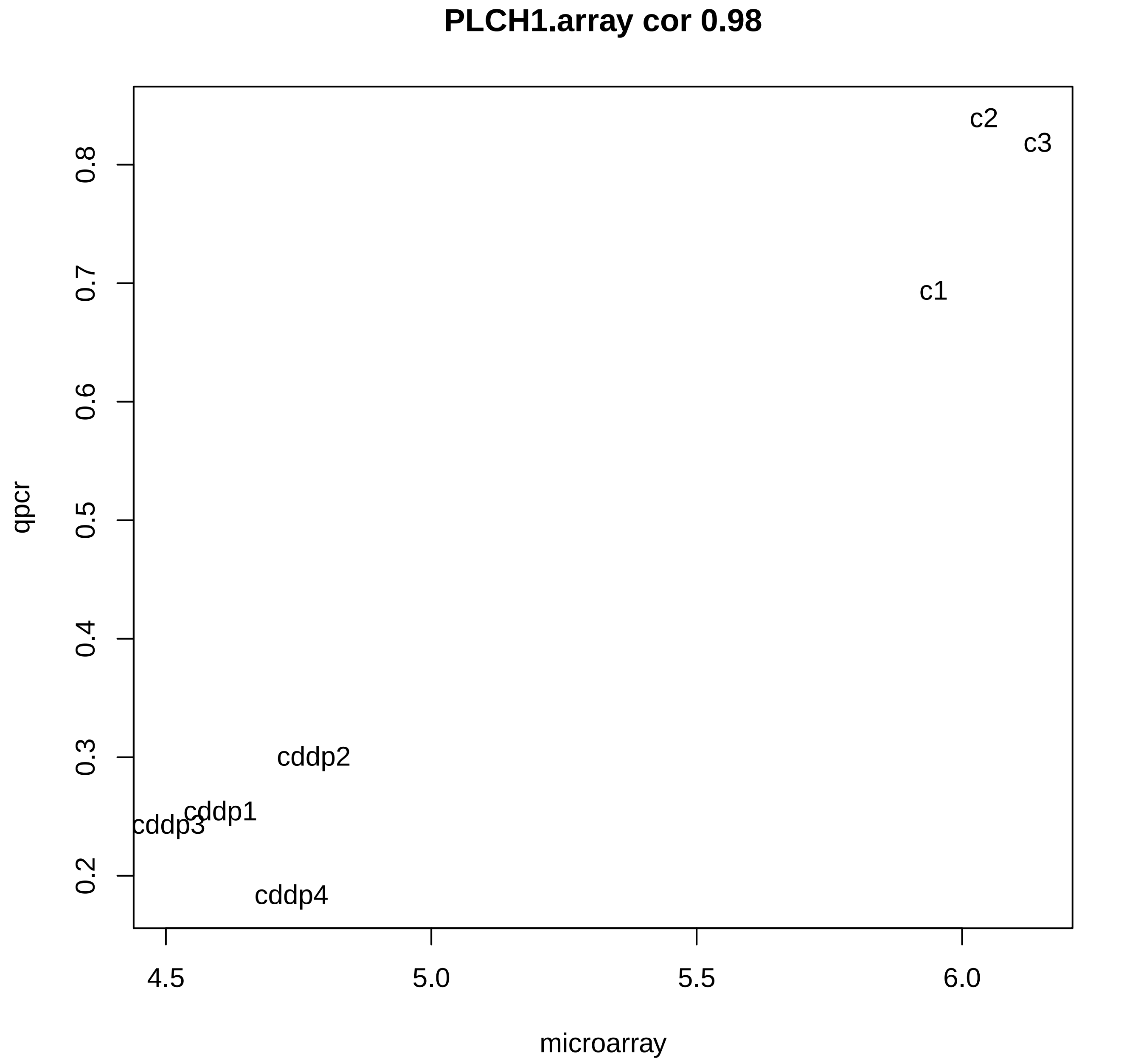
PLCH1.array cor 0.98
qpcr
microarray
0.2
0.3
0.4
0.5
0.6
0.7
0.8
4.5
5.0
5.5
6.0
c2
c3
c1
cddp2
cddp1
cddp3
cddp4

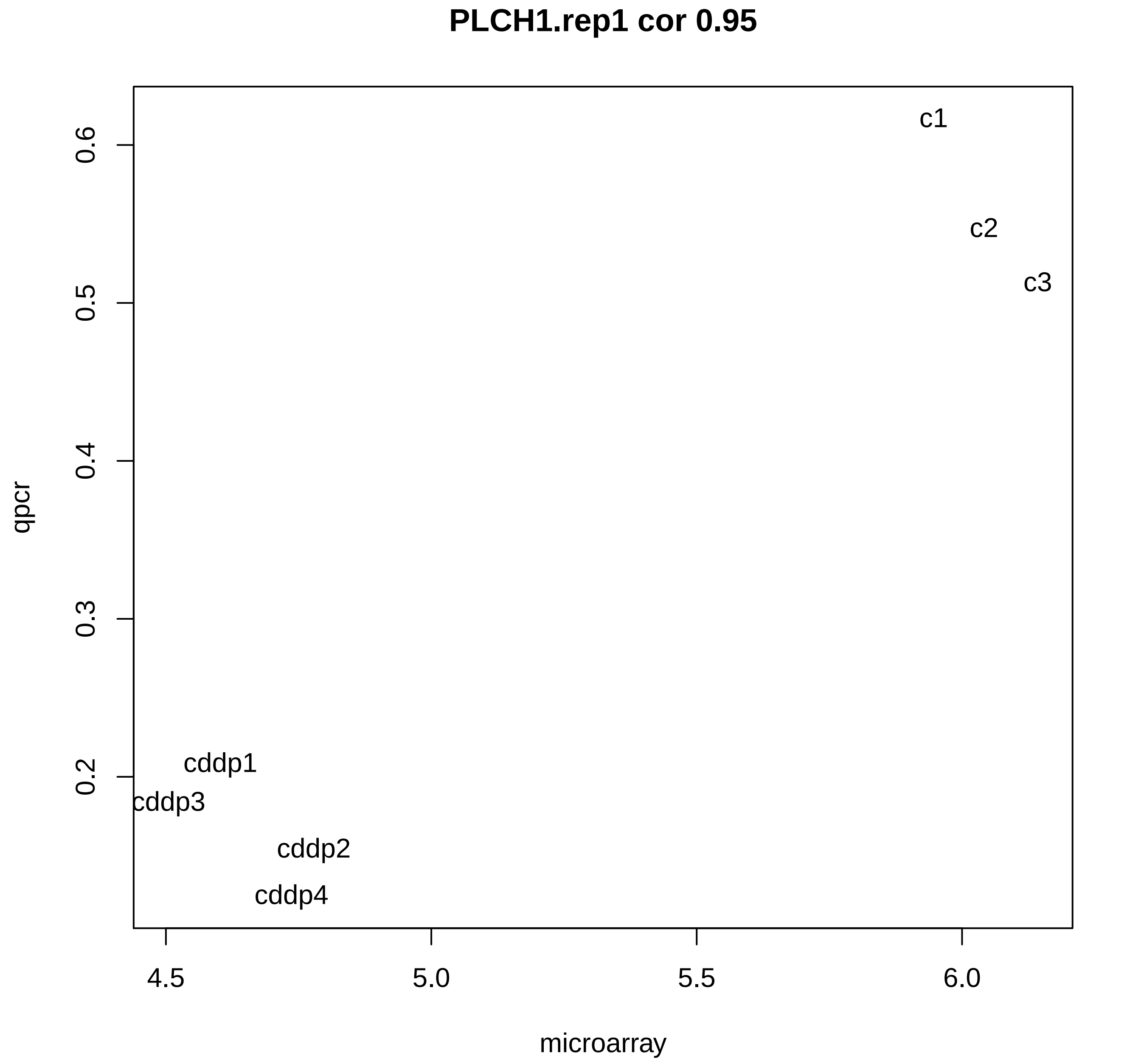
PLCH1.rep1 cor 0.95
qpcr
microarray
0.2
0.3
0.4
0.5
0.6
4.5
5.0
5.5
6.0
c1
c2
c3
cddp1
cddp3
cddp2
cddp4

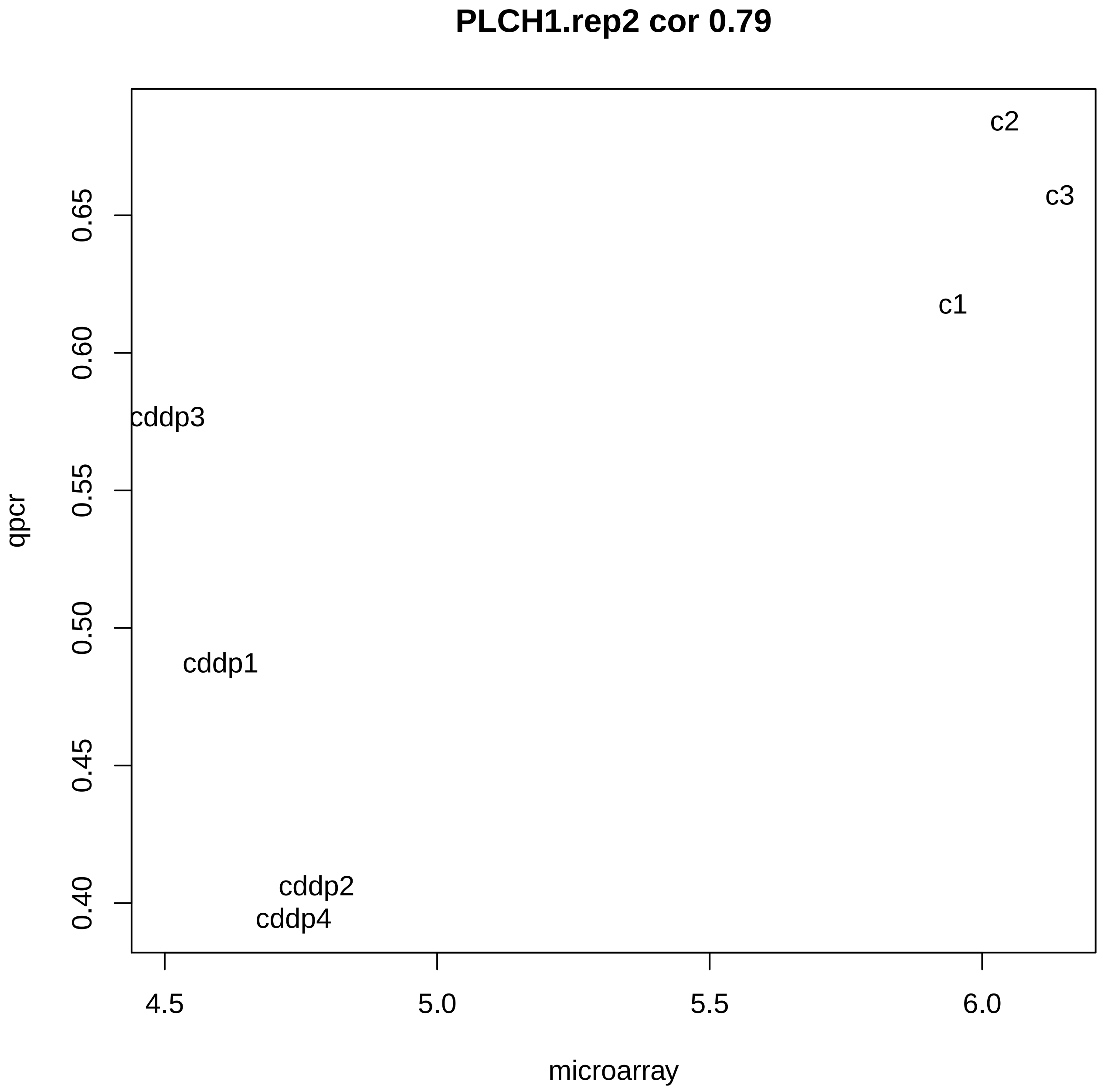
PLCH1.rep2 cor 0.79
qpcr
microarray
0.40
0.45
0.50
0.55
0.60
0.65
4.5
5.0
5.5
6.0
c2
c3
c1
cddp3
cddp1
cddp2
cddp4

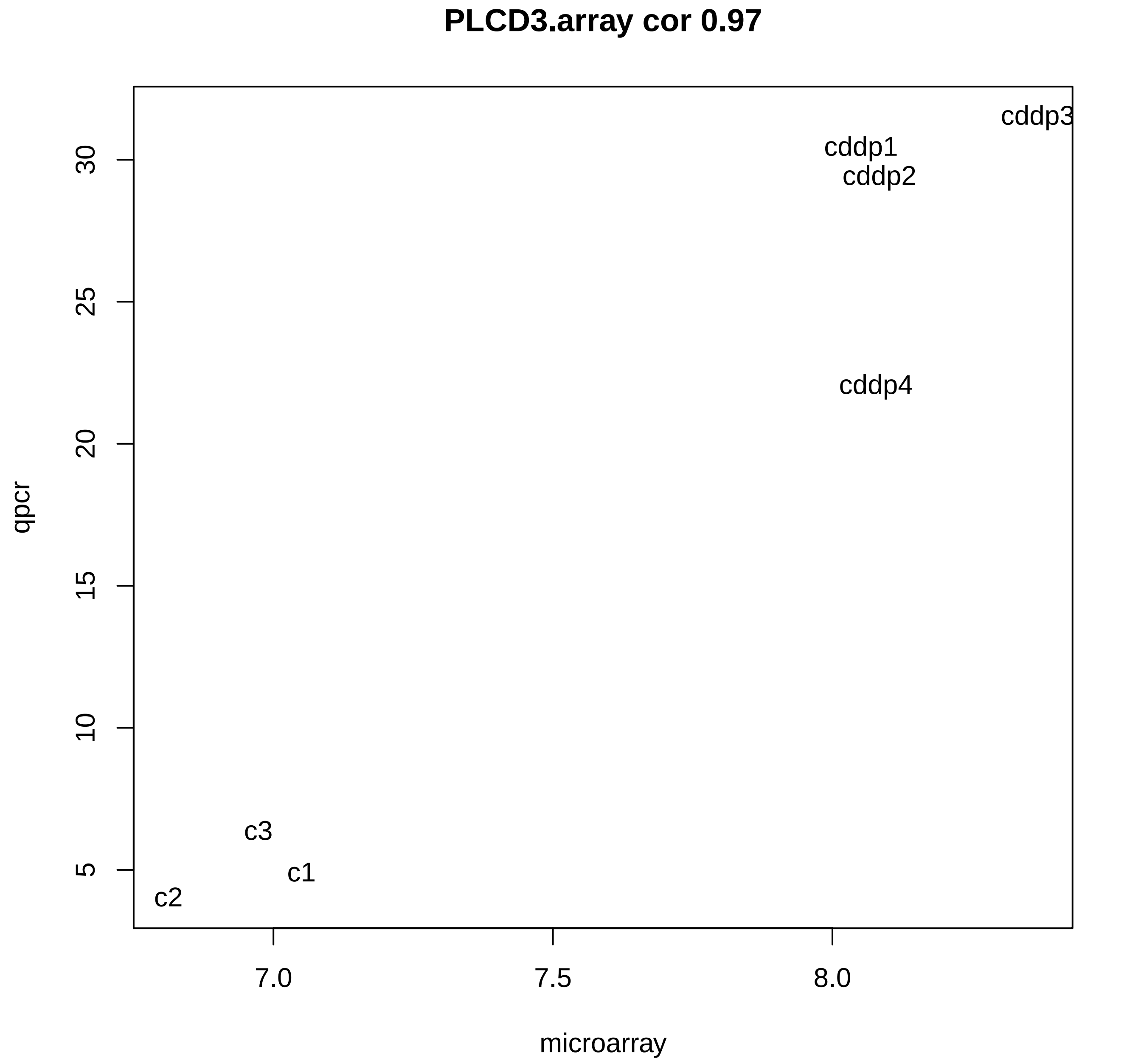
PLCD3.array cor 0.97
cddp3
cddp1
cddp2
cddp4
c3
c1
c2
30
25
20
15
10
5
qpcr
7.0
7.5
8.0
microarray

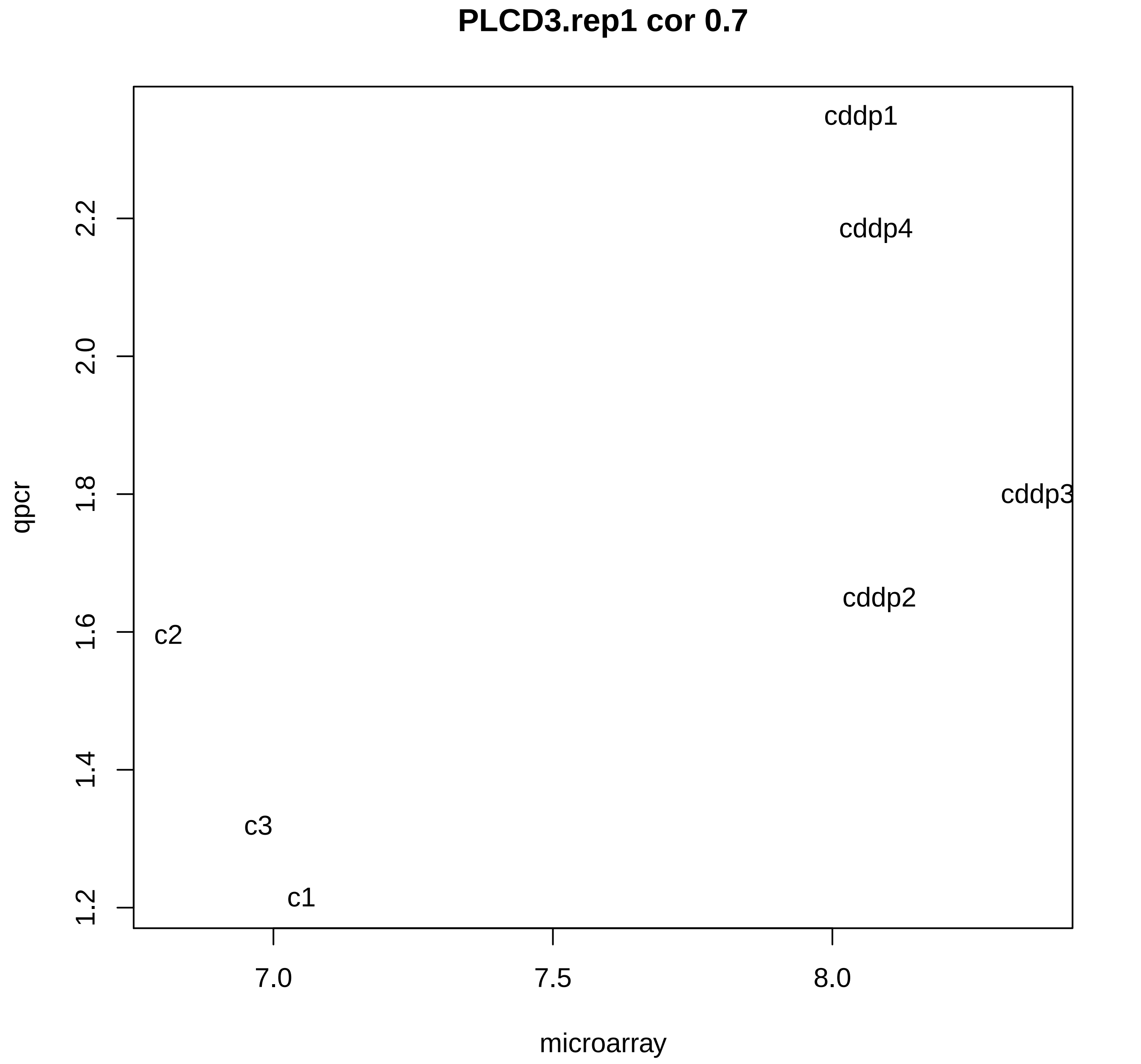
PLCD3.rep1 cor 0.7
cddp1
cddp4
cddp3
cddp2
c2
c3
c1
2.2
2.0
1.8
1.6
1.4
1.2
qpcr
7.0
7.5
8.0
microarray

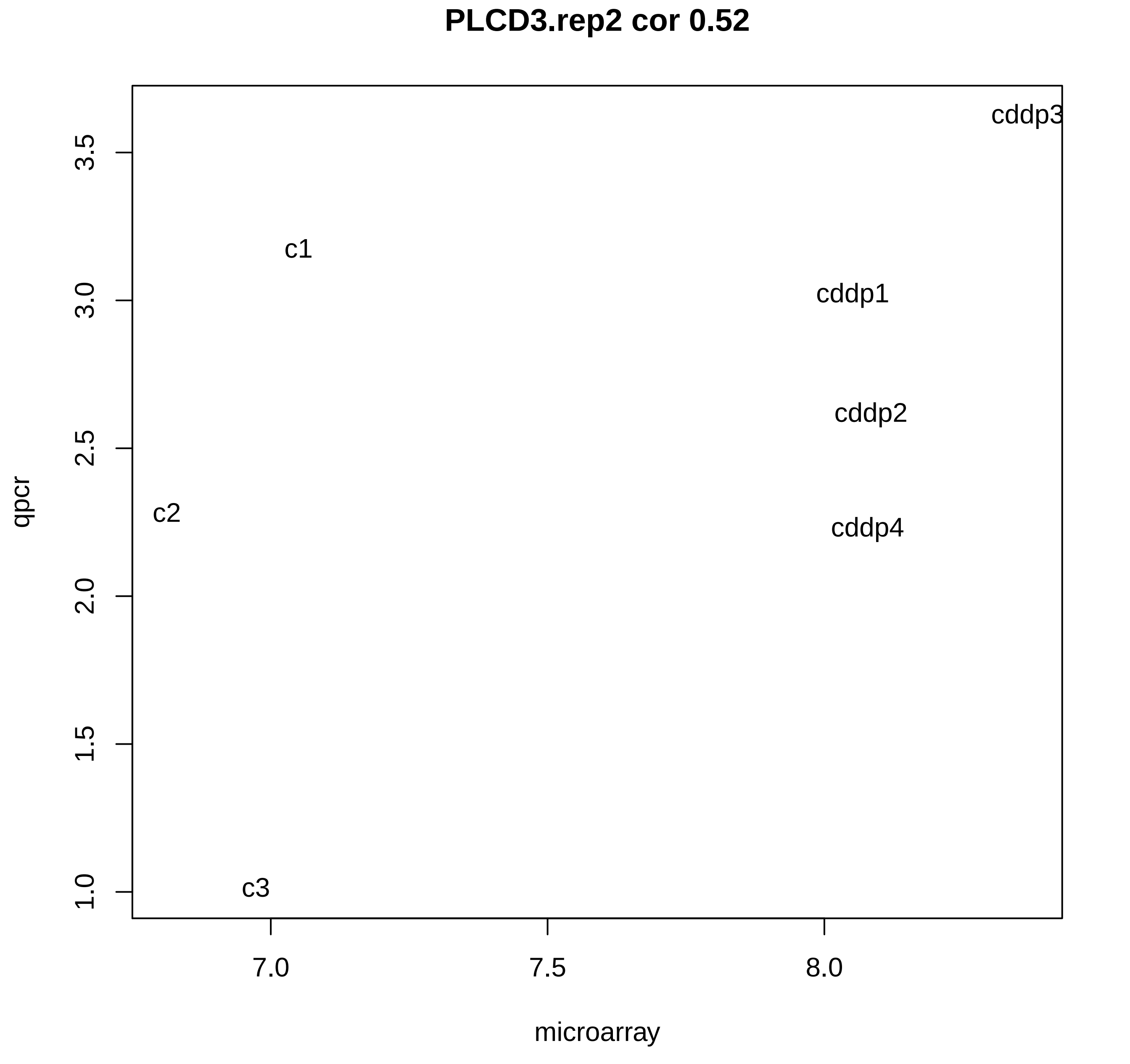
PLCD3.rep2 cor 0.52
cddp3
c1
cddp1
cddp2
c2
cddp4
c3
3.5
3.0
2.5
2.0
1.5
1.0
qpcr
7.0
7.5
8.0
microarray

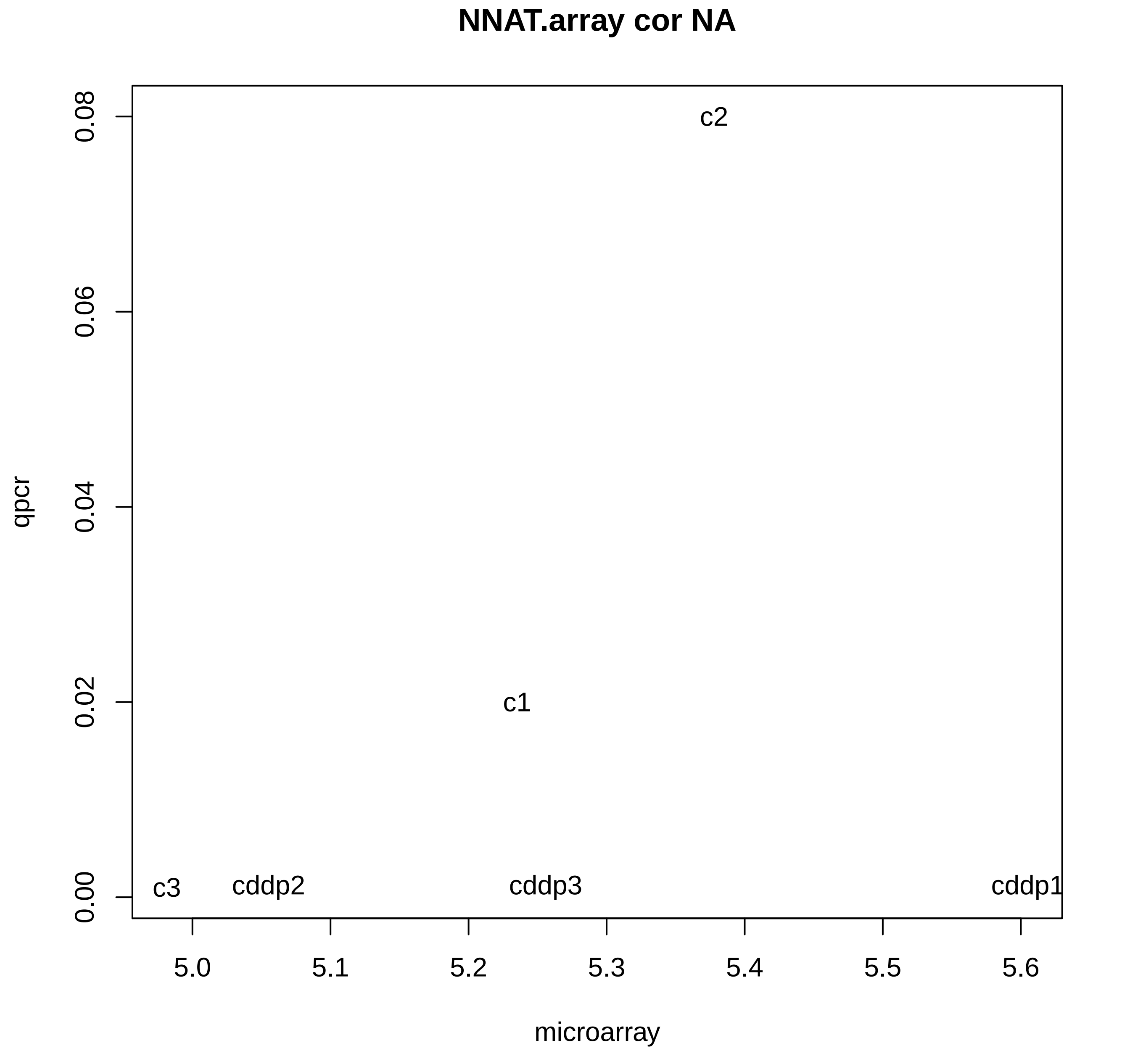

NNAT.array cor NA
qpcr
microarray
0.00
0.02
0.04
0.06
0.08
5.0
5.1
5.2
5.3
5.4
5.5
5.6
c2
c1
c3
cddp2
cddp3
cddp1

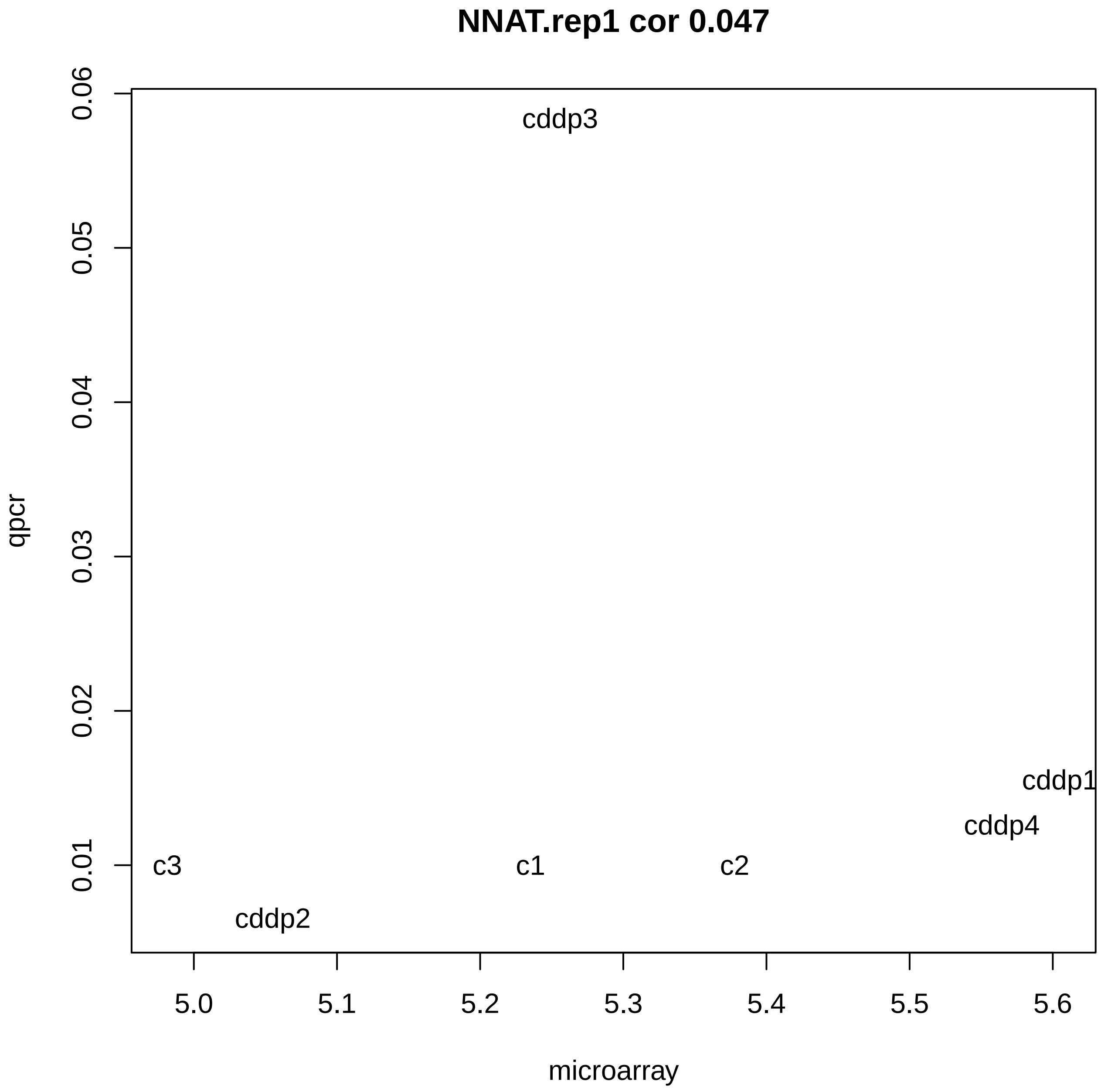
NNAT.rep1 cor 0.047
qpcr
0.06
0.05
0.04
0.03
0.02
0.01
cddp3
cddp1
cddp4
c3
c1
c2
cddp2
5.0
5.1
5.2
5.3
5.4
5.5
5.6
microarray

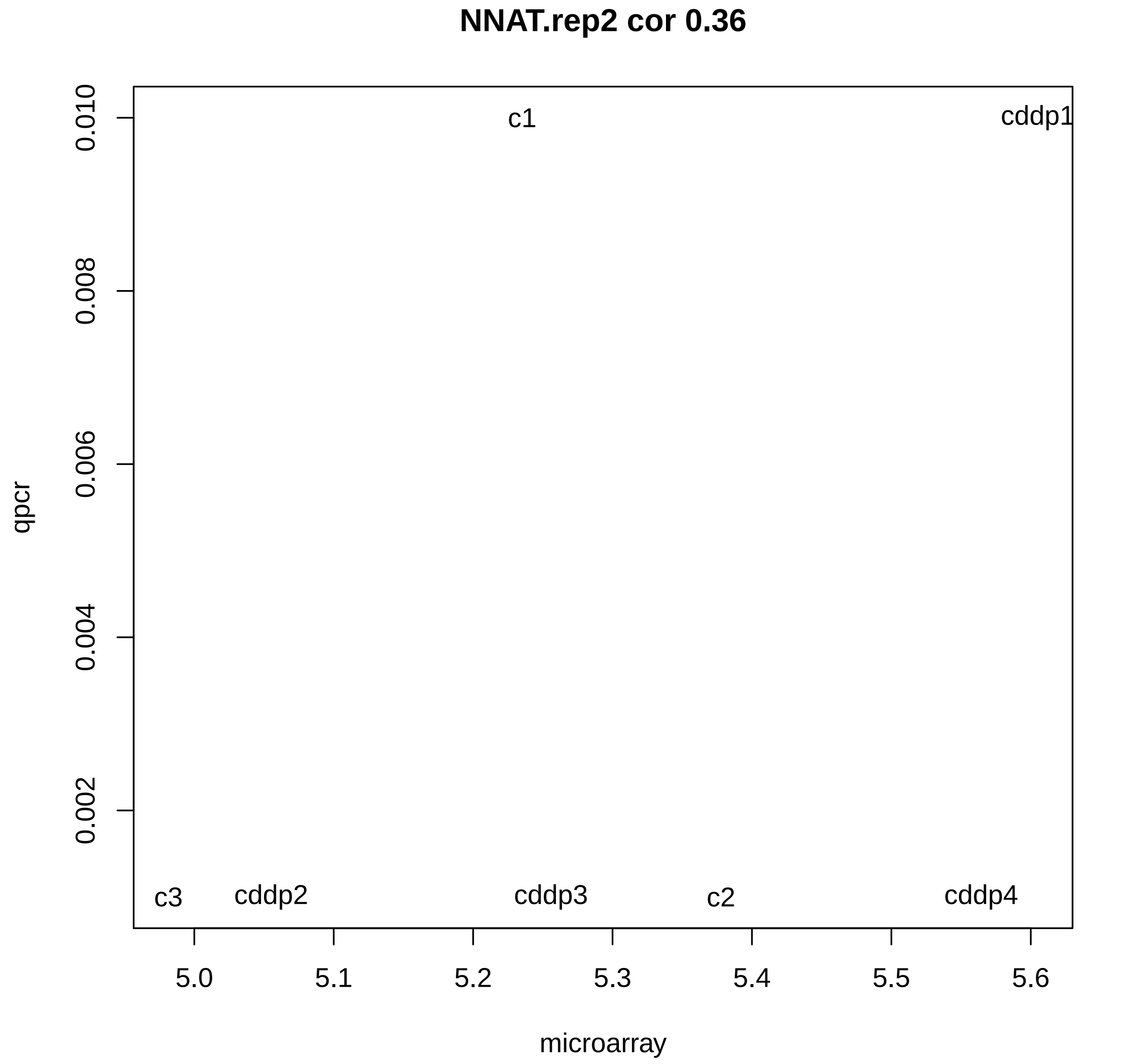

NNAT.rep2 cor 0.36
qpcr
0.010
0.008
0.006
0.004
0.002
c1
cddp1
c3
cddp2
cddp3
c2
cddp4
5.0
5.1
5.2
5.3
5.4
5.5
5.6
microarray

MYC.array cor 0.99

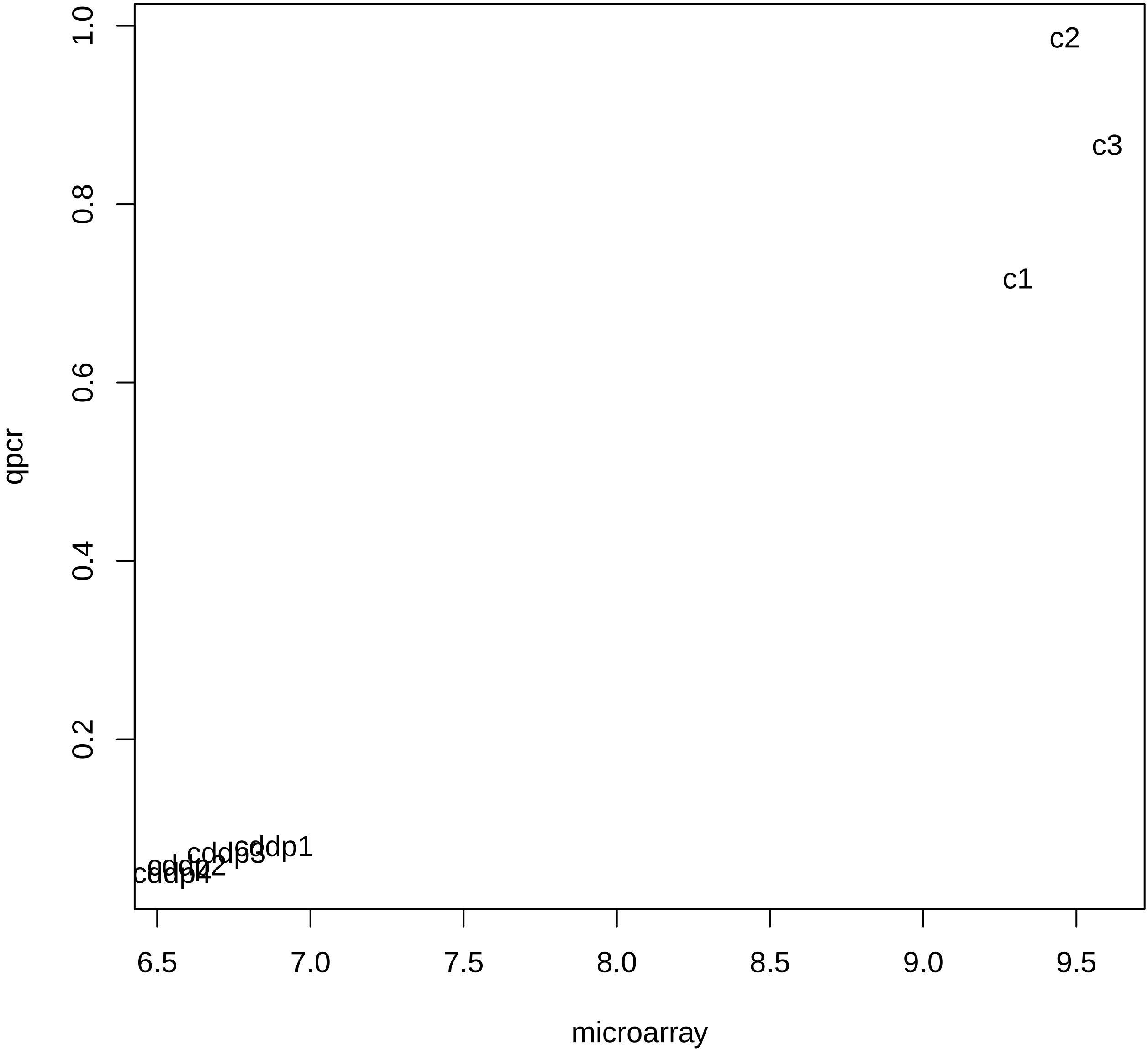

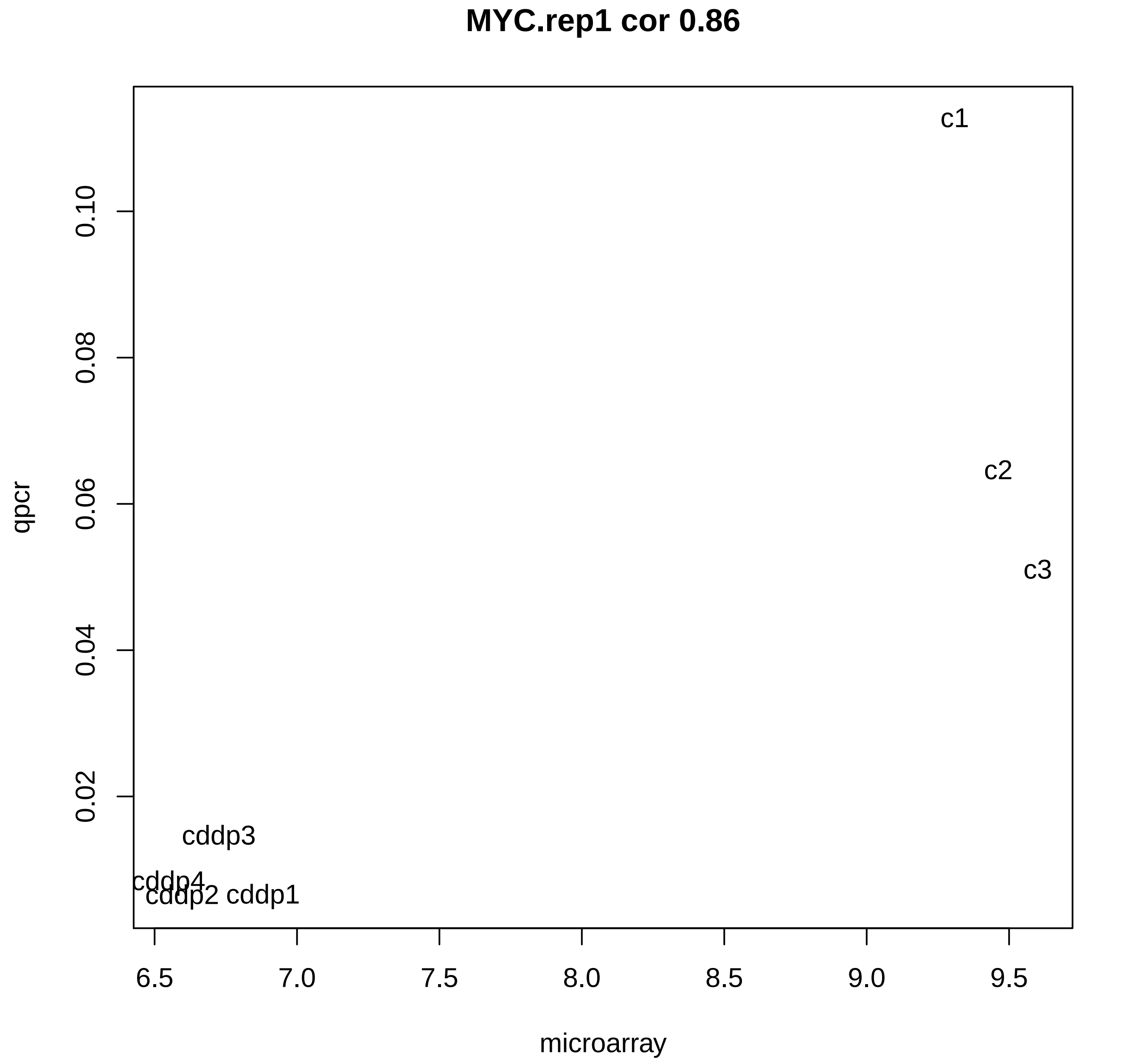

MYC.rep1 cor 0.86
c1
c2
c3
cddp3
cddp4
cddp2
cddp1
0.02
0.04
0.06
0.08
0.10
qpcr
6.5
7.0
7.5
8.0
8.5
9.0
9.5
microarray

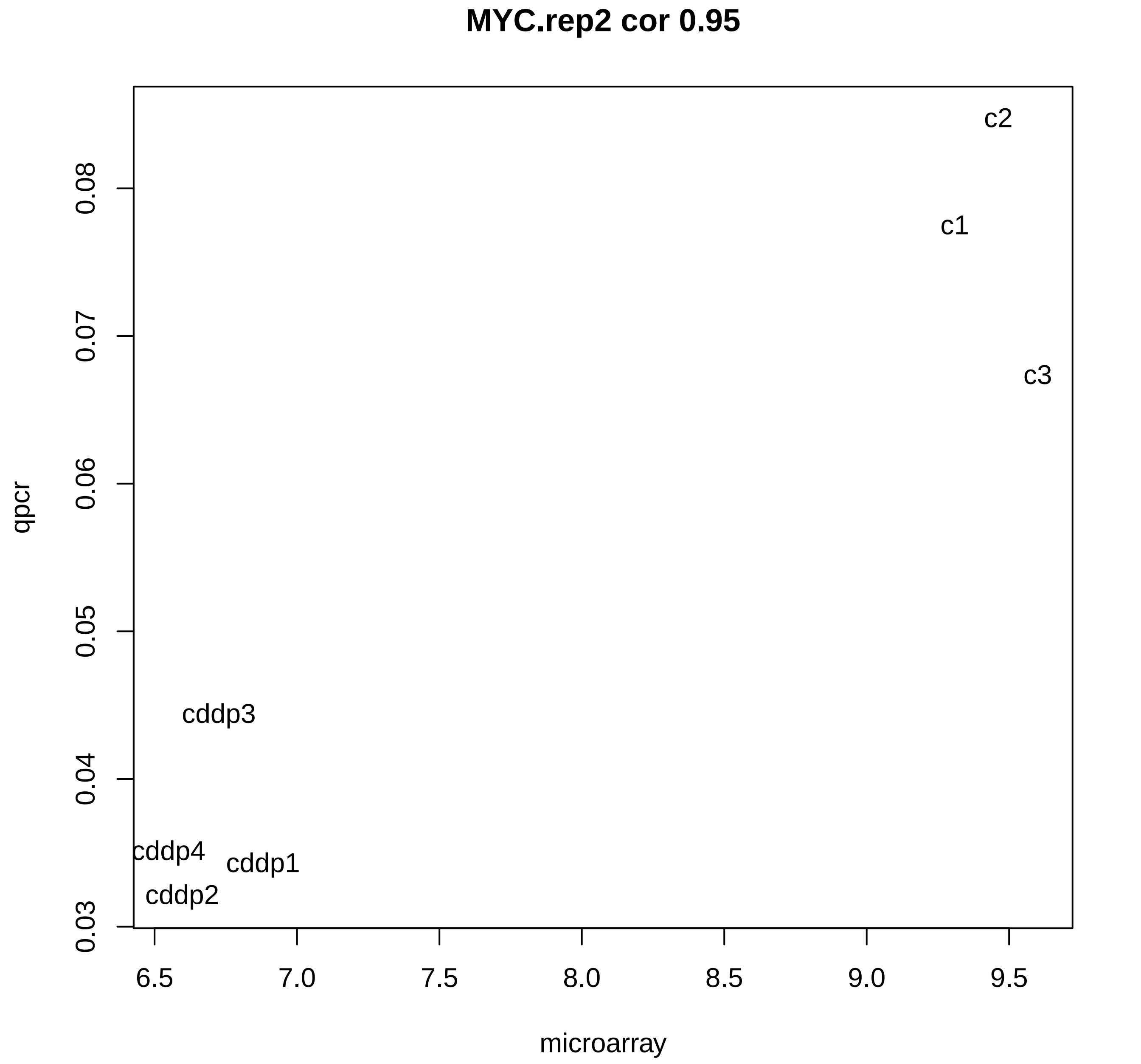
MYC.rep2 cor 0.95
qpcr
microarray
0.03
0.04
0.05
0.06
0.07
0.08
6.5
7.0
7.5
8.0
8.5
9.0
9.5
c2
c1
c3
cddp3
cddp4
cddp1
cddp2

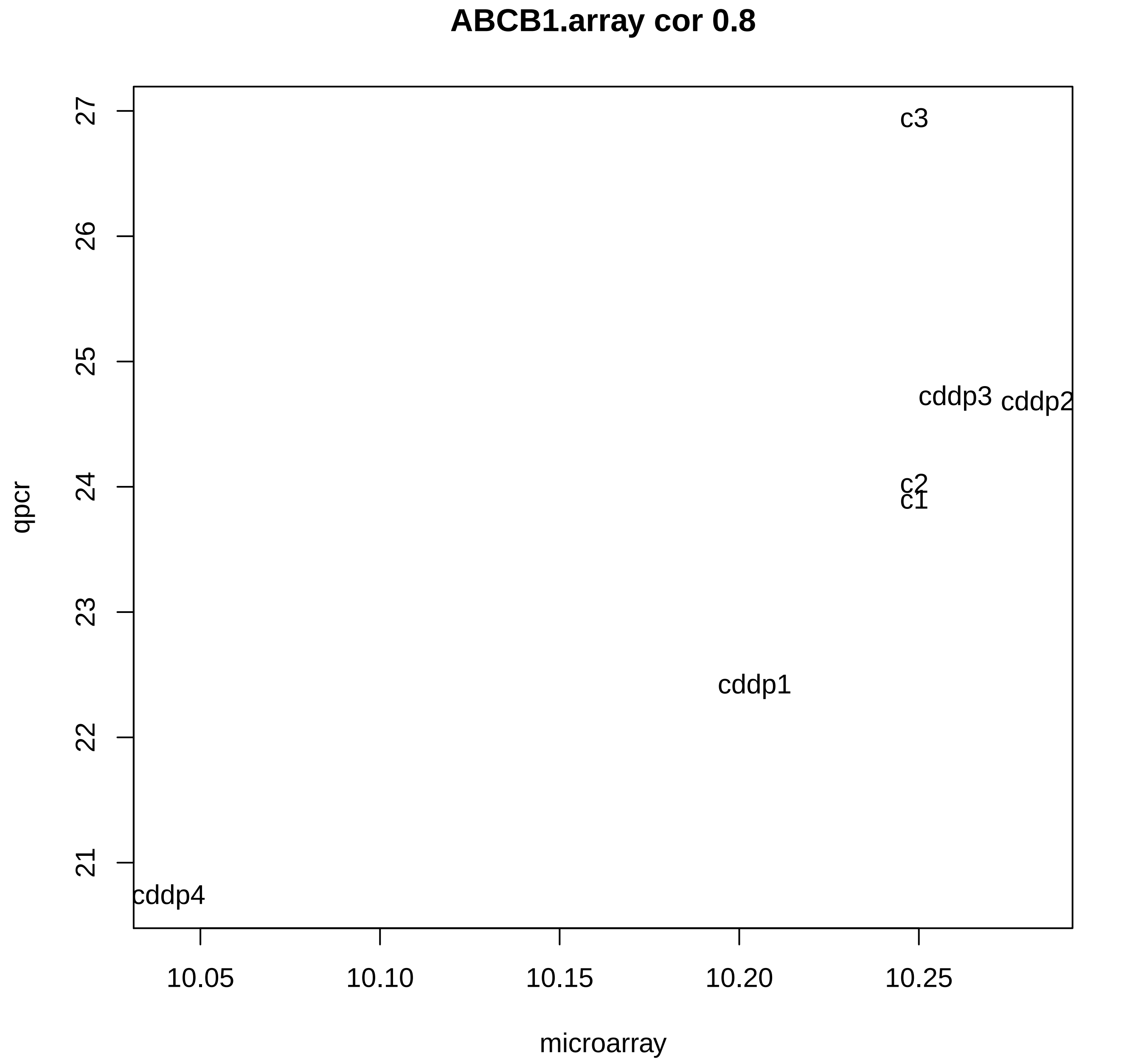
ABCB1.array cor 0.8
qpcr
microarray
27
26
25
24
23
22
21
10.05
10.10
10.15
10.20
10.25
c3
cddp3
cddp2
c2
c1
cddp1
cddp4

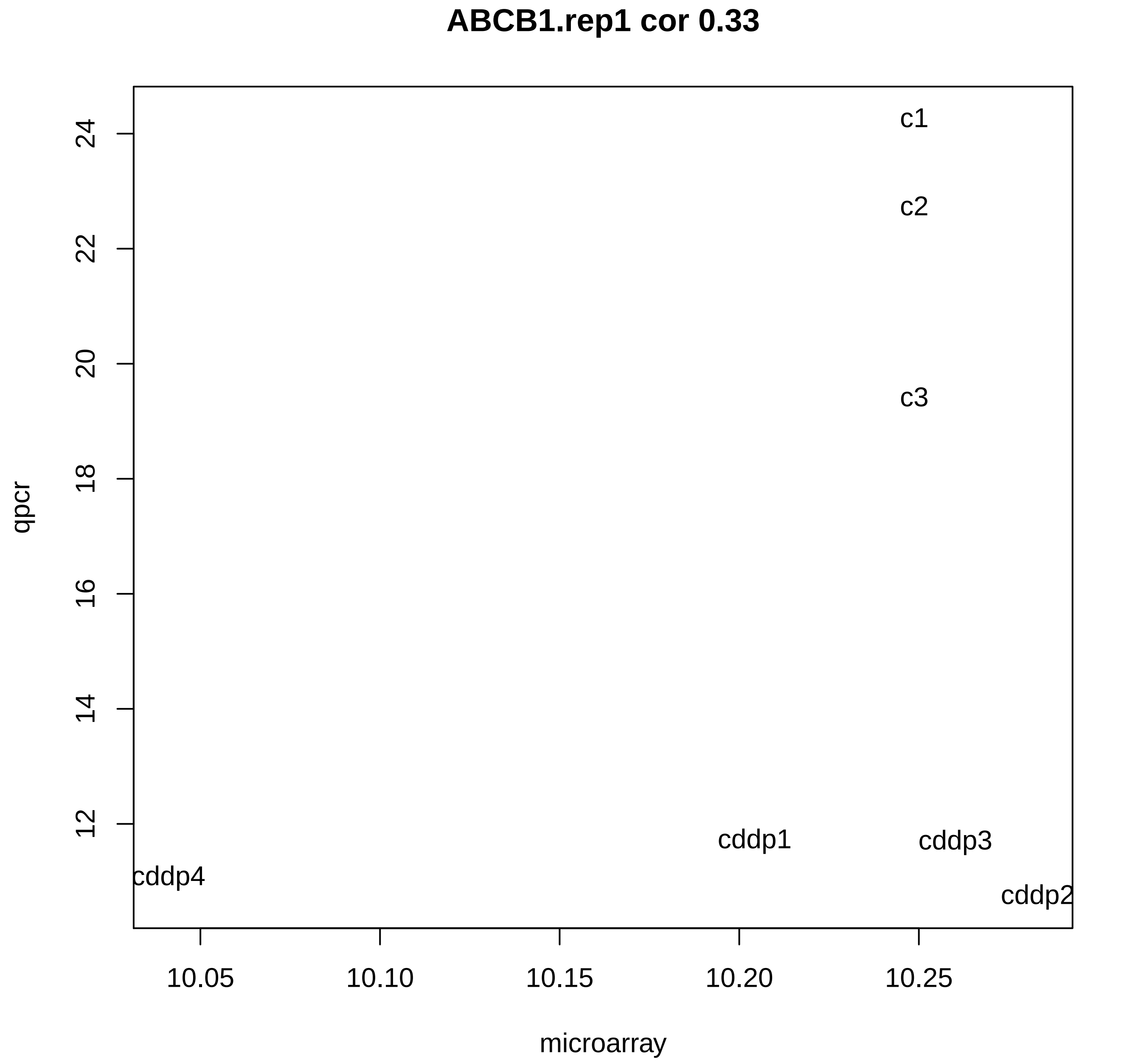
ABCB1.rep1 cor 0.33
qpcr
24
22
20
18
16
14
12
c1
c2
c3
cddp1
cddp3
cddp4
cddp2
10.05
10.10
10.15
10.20
10.25
microarray

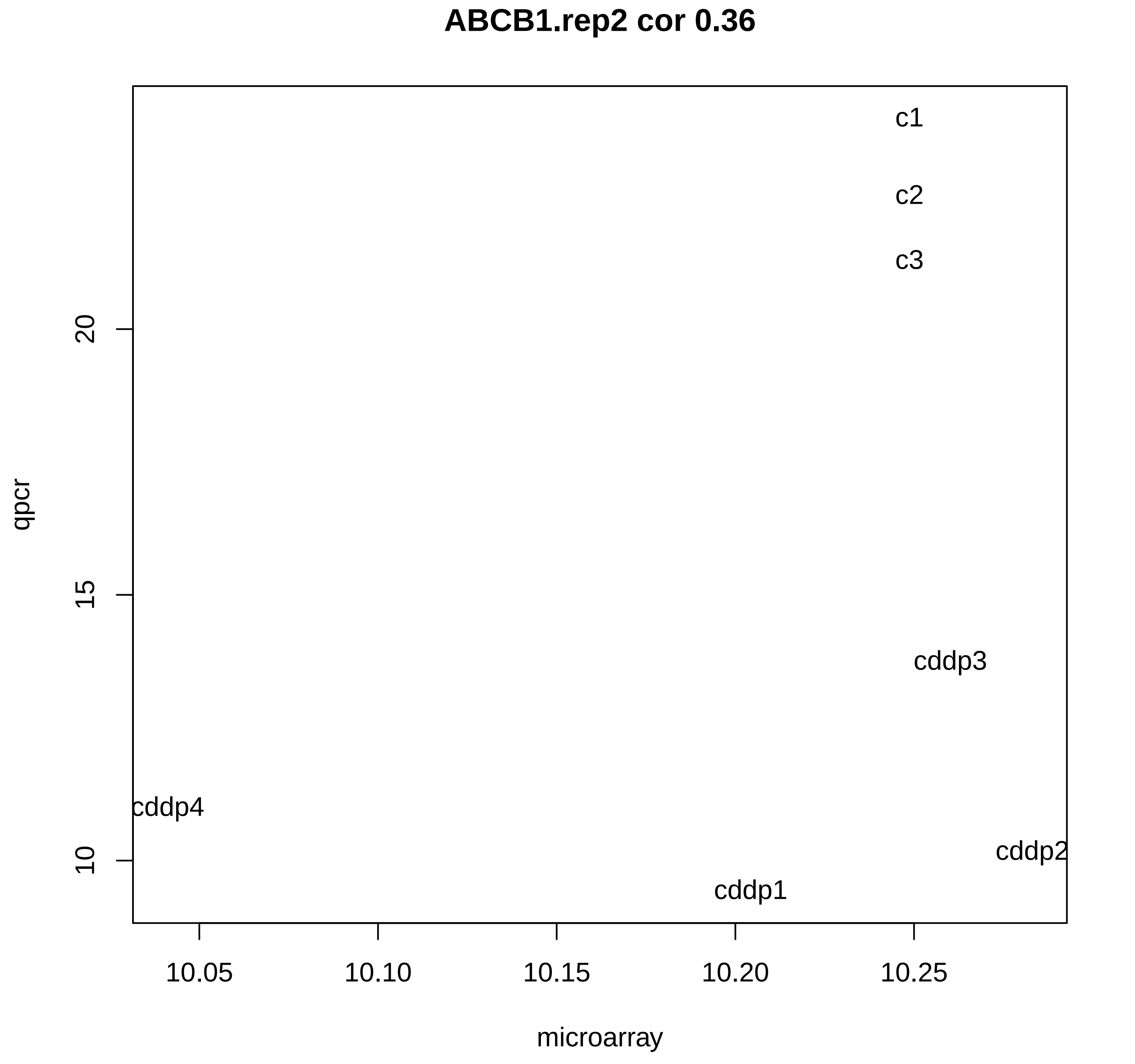

ABCB1.rep2 cor 0.36
c1
c2
c3
cddp3
cddp4
cddp2
cddp1
20
15
10
qpcr
10.05
10.10
10.15
10.20
10.25
microarray

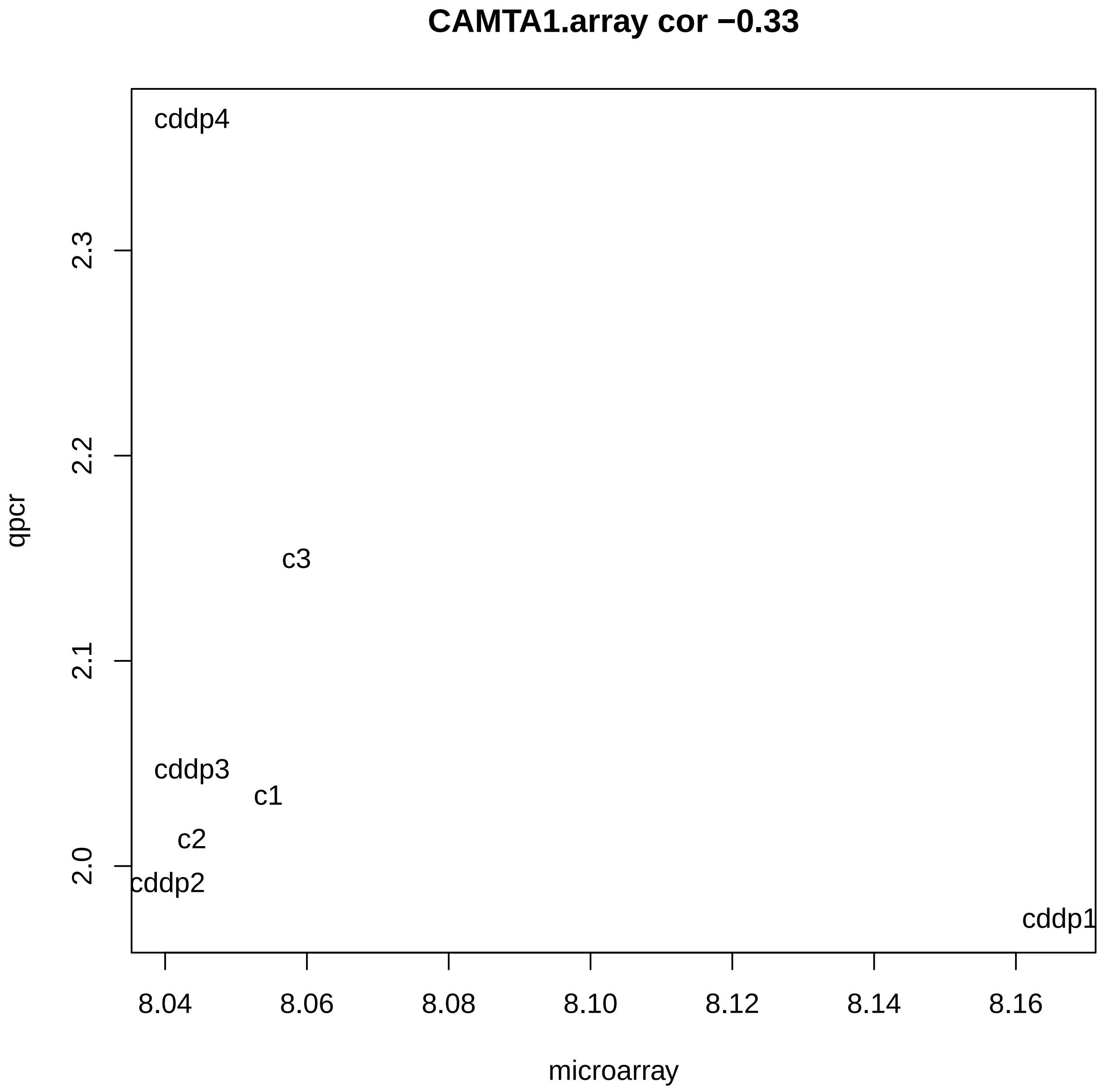
CAMTA1.array cor −0.33
cddp4
c3
cddp3
c1
c2
cddp2
cddp1
2.0
2.1
2.2
2.3
qpcr
8.04
8.06
8.08
8.10
8.12
8.14
8.16
microarray

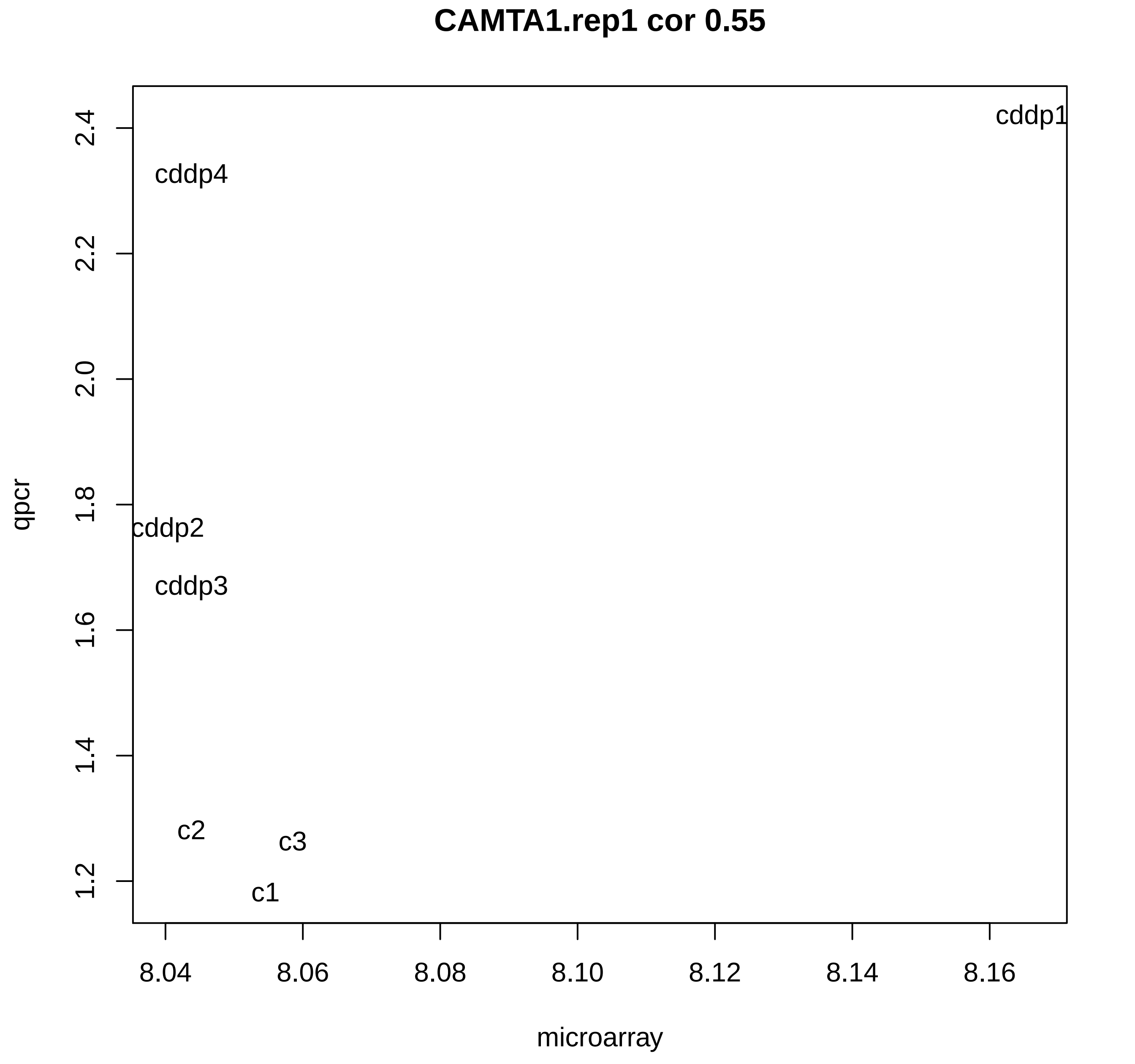
CAMTA1.rep1 cor 0.55
cddp1
cddp4
cddp2
cddp3
c2
c3
c1
2.4
2.2
2.0
1.8
1.6
1.4
1.2
qpcr
8.04
8.06
8.08
8.10
8.12
8.14
8.16
microarray

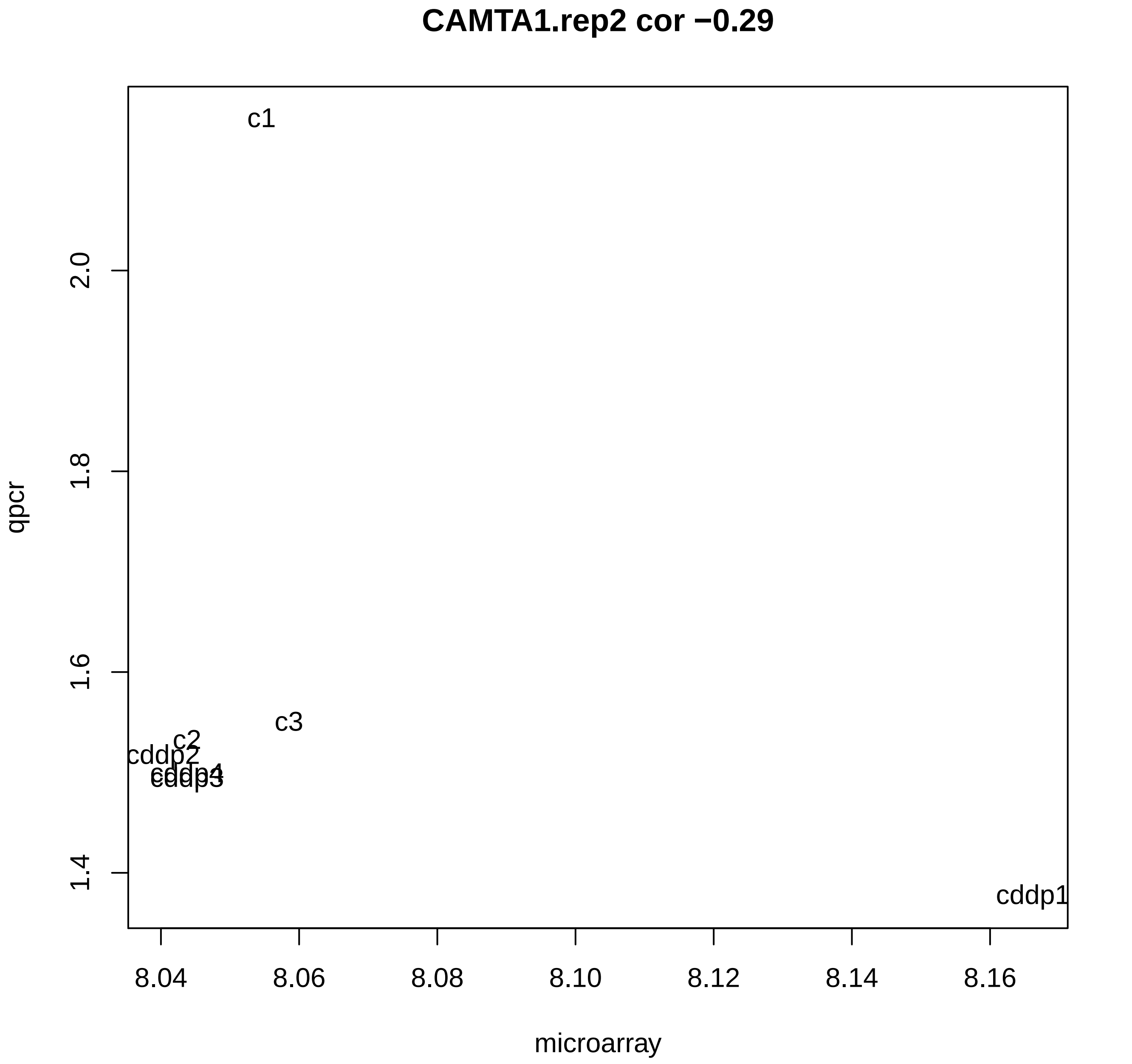
CAMTA1.rep2 cor −0.29
qpcr
microarray
2.0
1.8
1.6
1.4
8.04
8.06
8.08
8.10
8.12
8.14
8.16
c1
c3
c2
cddp2
cddp4
cddp3
cddp1

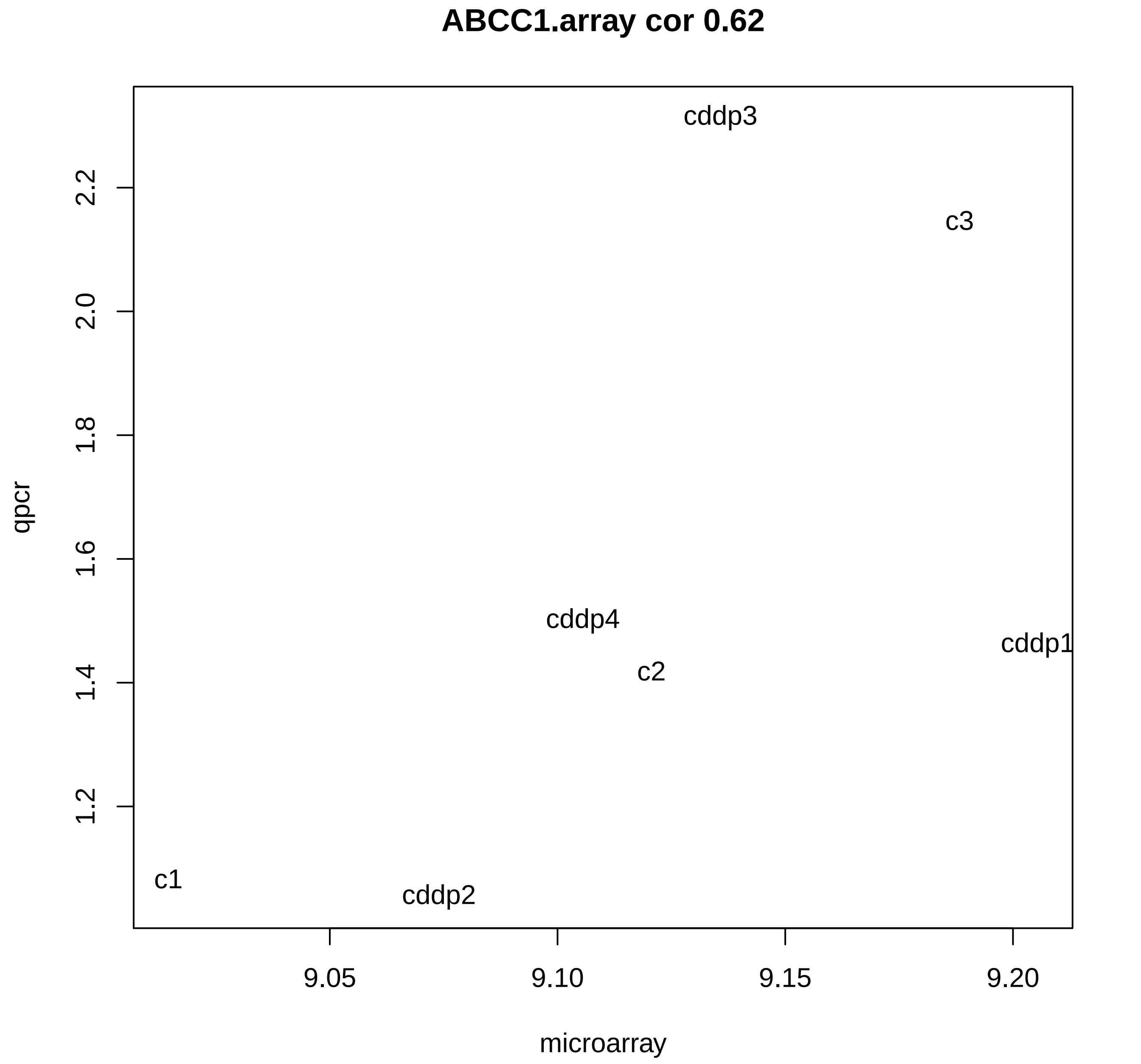
ABCC1.array cor 0.62
cddp3
c3
cddp4
cddp1
c2
c1
cddp2
2.2
2.0
1.8
1.6
1.4
1.2
qpcr
9.05
9.10
9.15
9.20
microarray

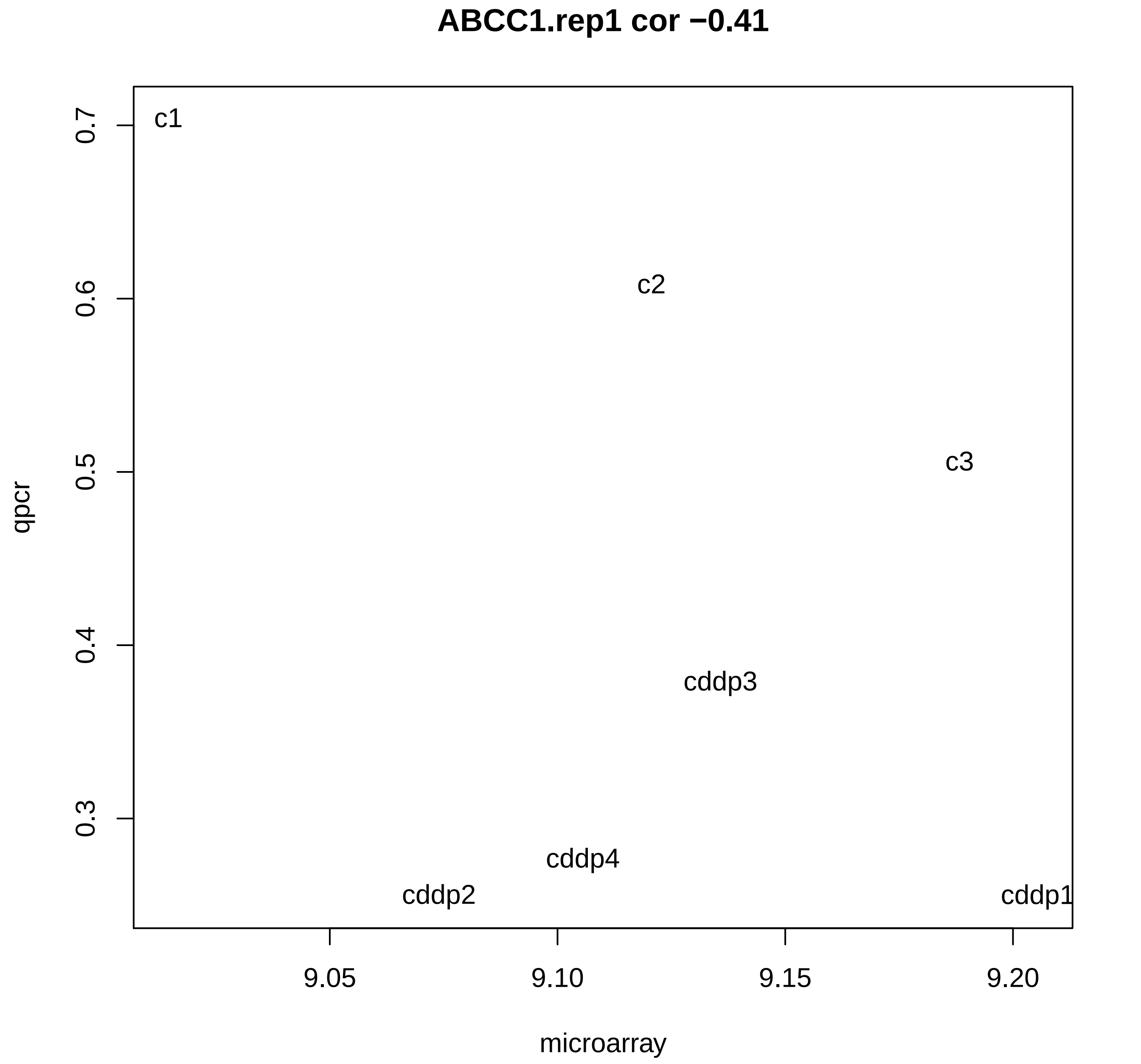

ABCC1.rep1 cor −0.41
qpcr
0.3
0.4
0.5
0.6
0.7
c1
c2
c3
cddp3
cddp4
cddp2
cddp1
9.05
9.10
9.15
9.20
microarray

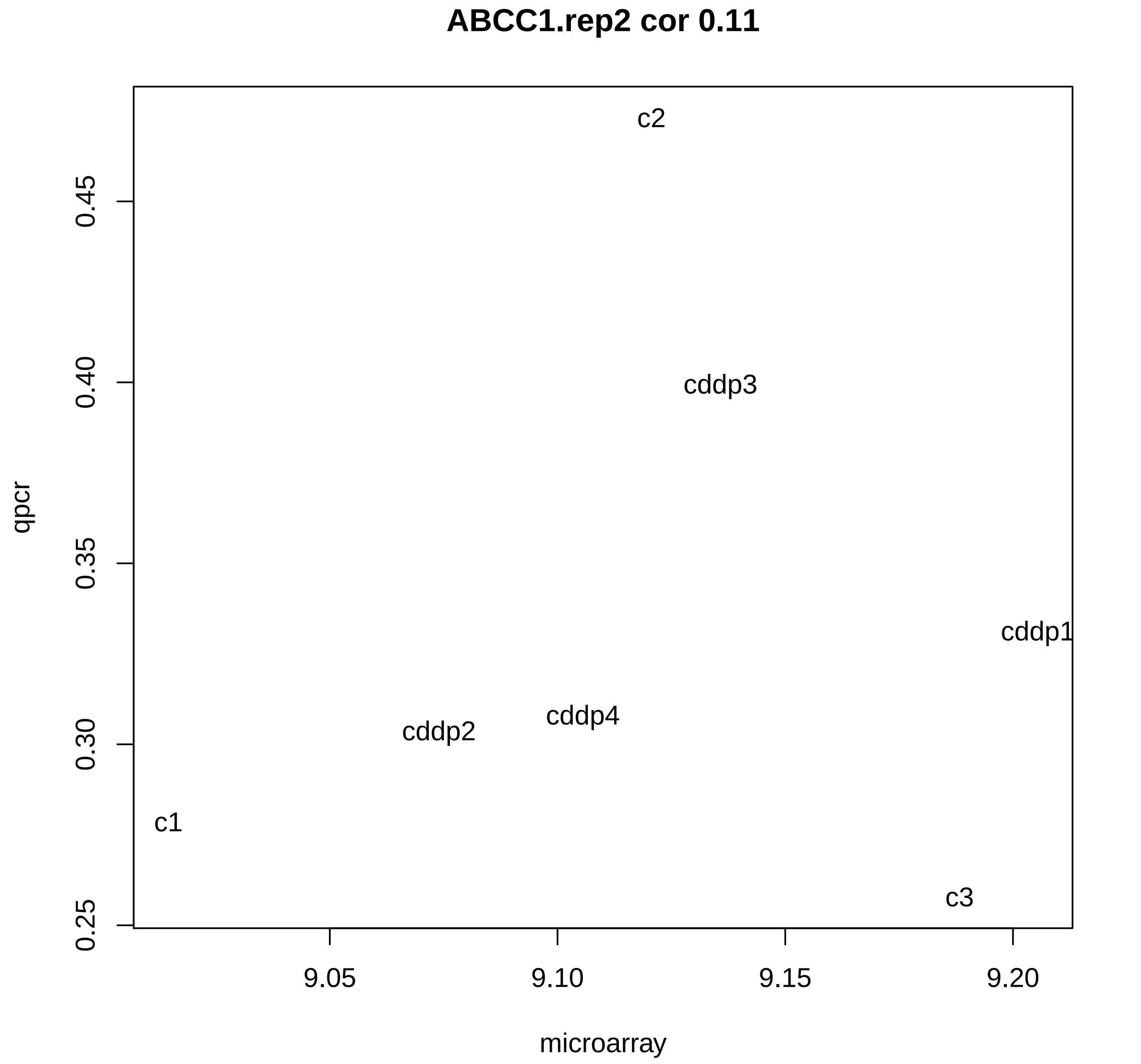
ABCC1.rep2 cor 0.11
c2
cddp3
cddp1
cddp4
cddp2
c1
c3
0.25
0.30
0.35
0.40
0.45
qpcr
9.05
9.10
9.15
9.20
microarray

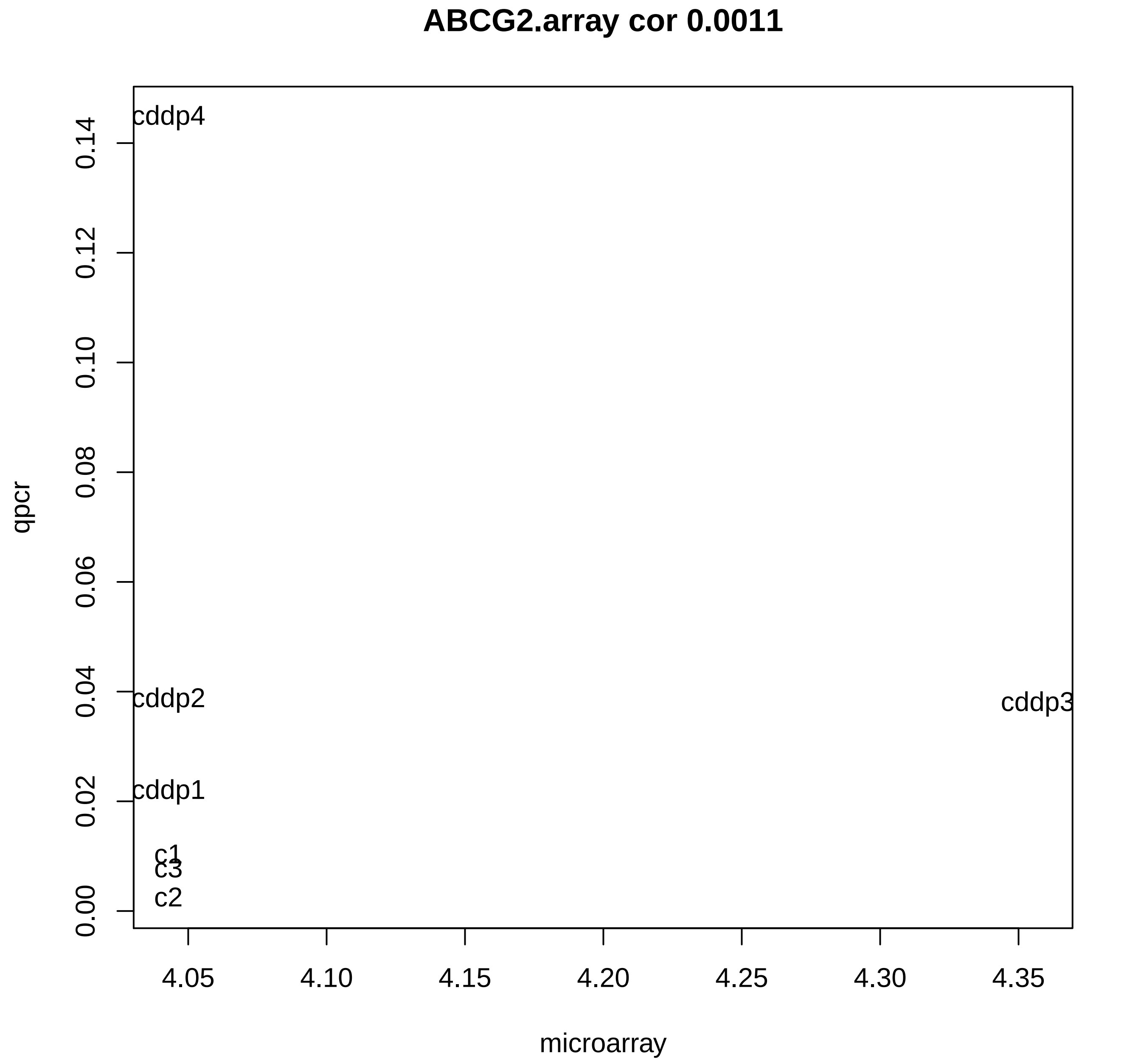
ABCG2.array cor 0.0011
qpcr
microarray
0.00
0.02
0.04
0.06
0.08
0.10
0.12
0.14
4.05
4.10
4.15
4.20
4.25
4.30
4.35
cddp4
cddp2
cddp3
cddp1
c1
c3
c2

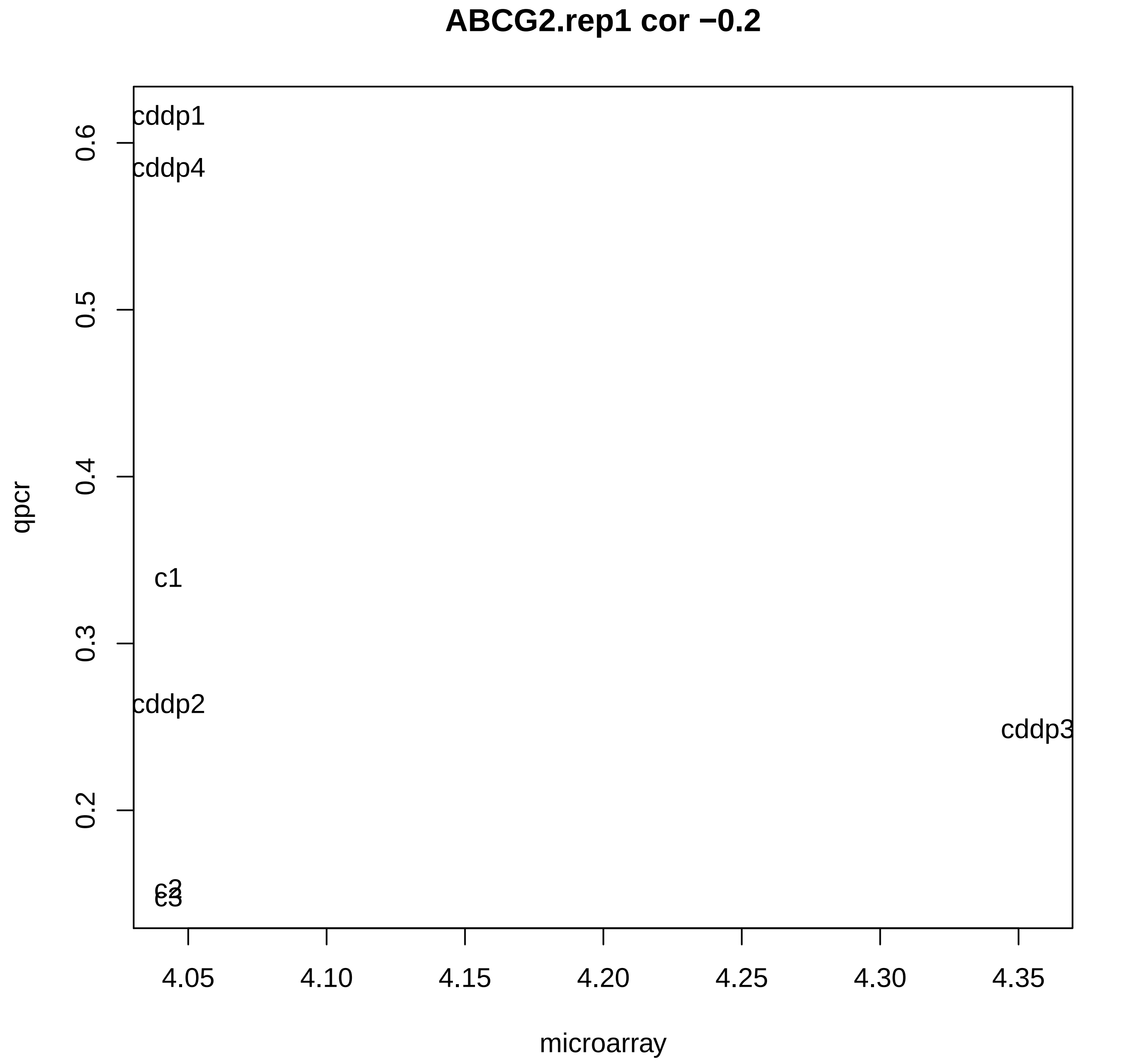
ABCG2.rep1 cor −0.2
qpcr
microarray
0.2
0.3
0.4
0.5
0.6
4.05
4.10
4.15
4.20
4.25
4.30
4.35
cddp1
cddp4
c1
cddp2
cddp3
c2
c3

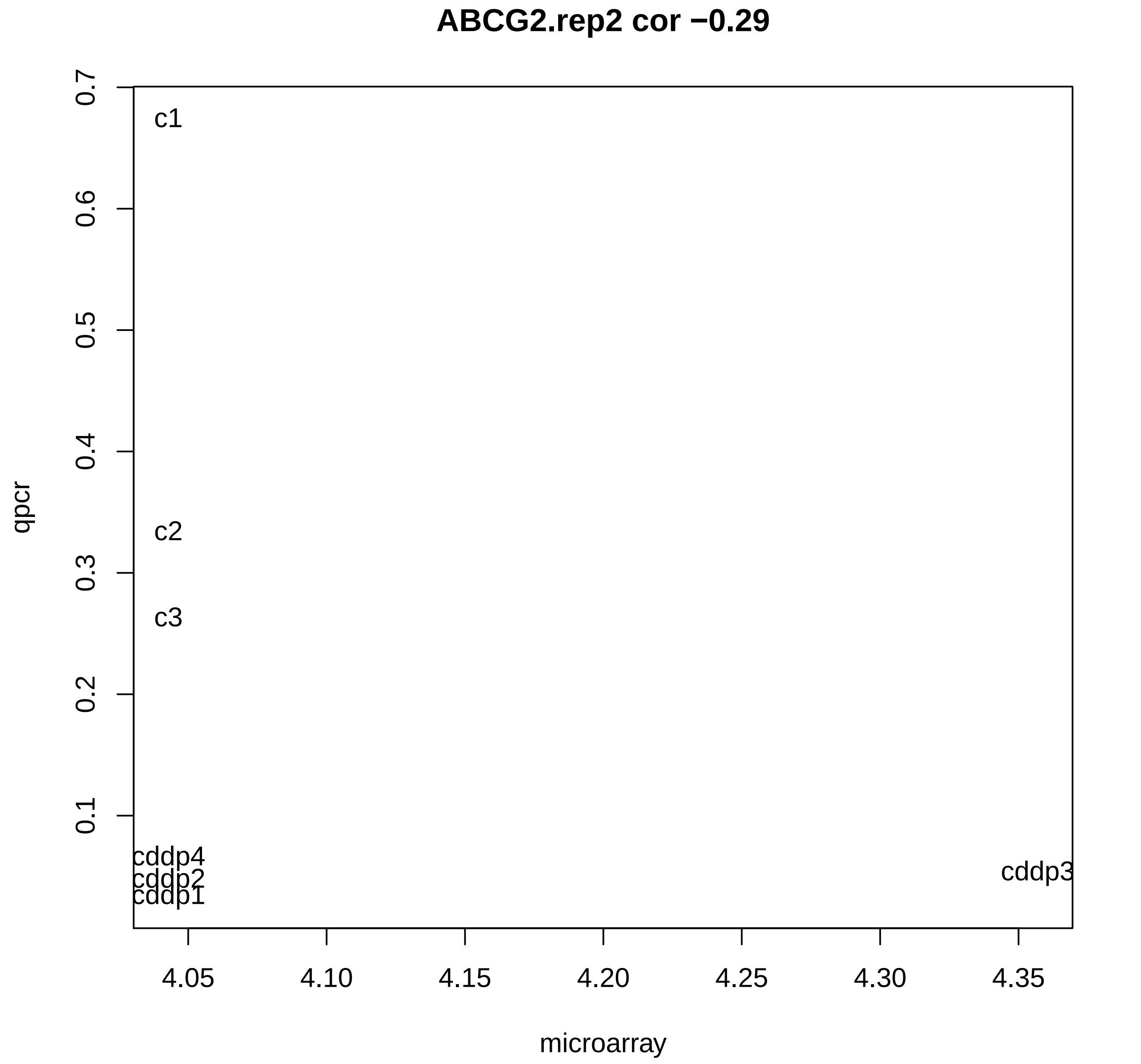
ABCG2.rep2 cor −0.29
qpcr
microarray
0.1
0.2
0.3
0.4
0.5
0.6
0.7
4.05
4.10
4.15
4.20
4.25
4.30
4.35
c1
c2
c3
cddp4
cddp2
cddp1
cddp3

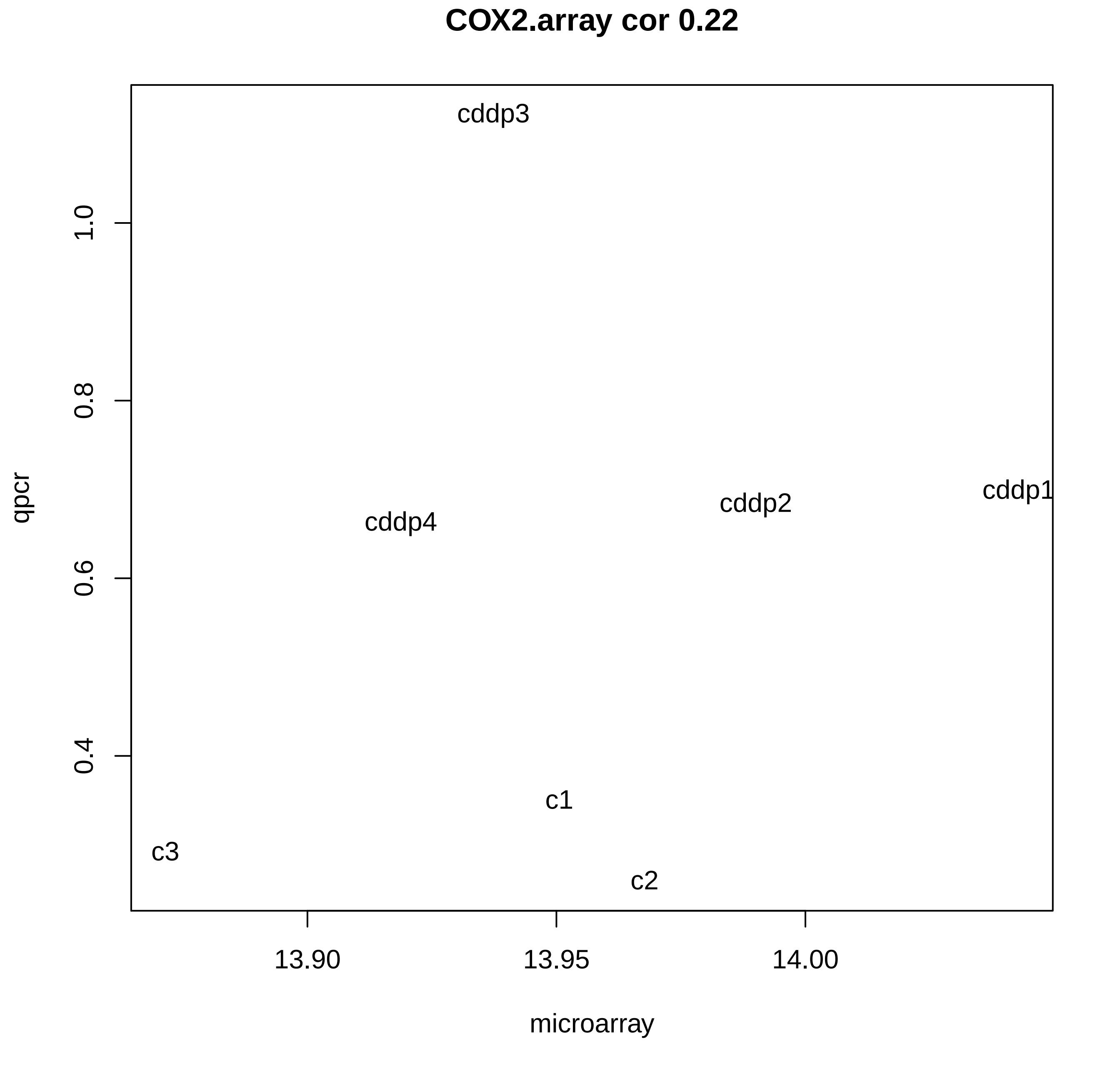
COX2.array cor 0.22
cddp3
cddp4
cddp2
cddp1
c1
c3
c2
1.0
0.8
0.6
0.4
13.90
13.95
14.00
qpcr
microarray

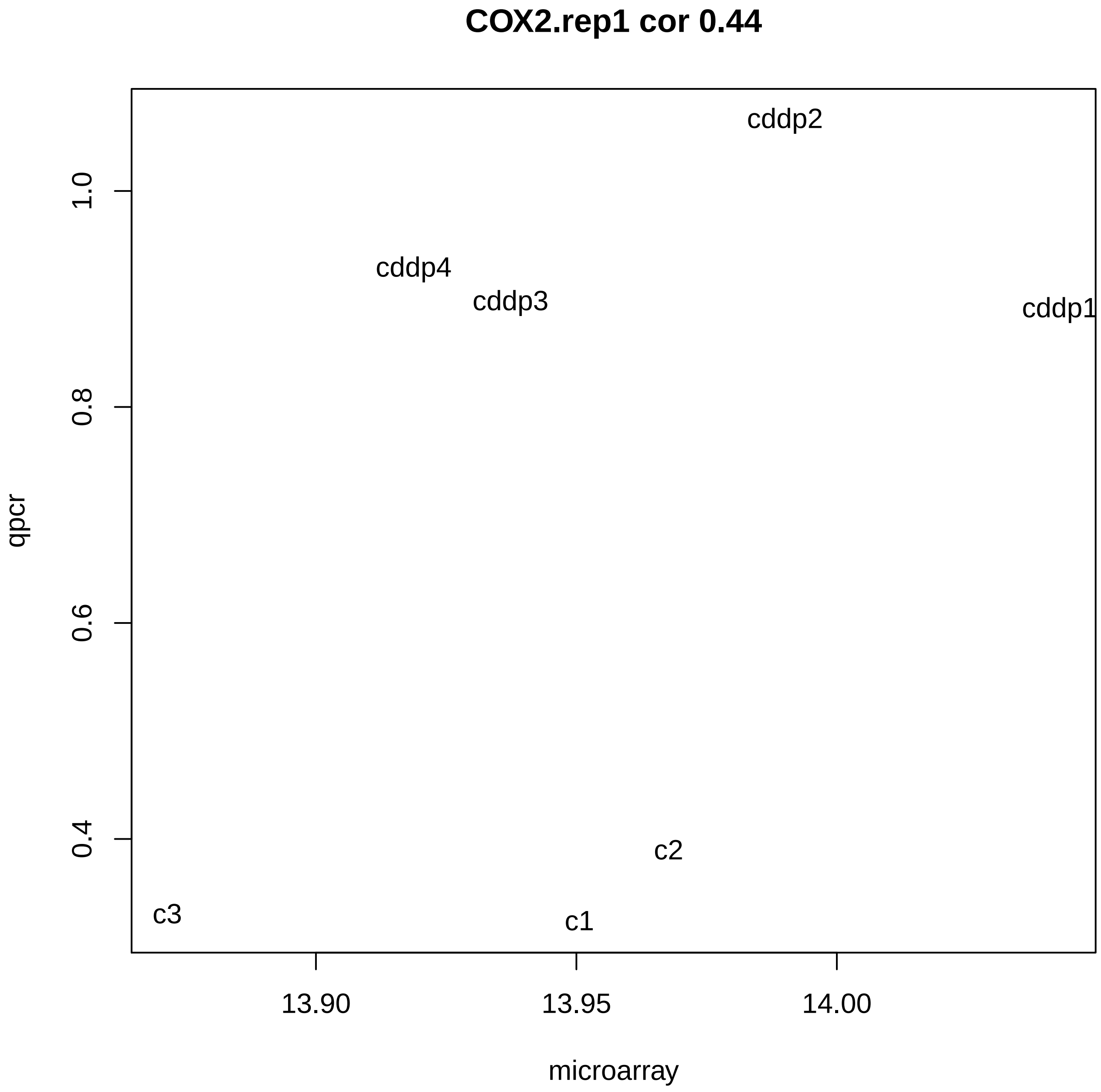

COX2.rep1 cor 0.44
cddp2
cddp4
cddp3
cddp1
c2
c3
c1
1.0
0.8
0.6
0.4
qpcr
13.90
13.95
14.00
microarray

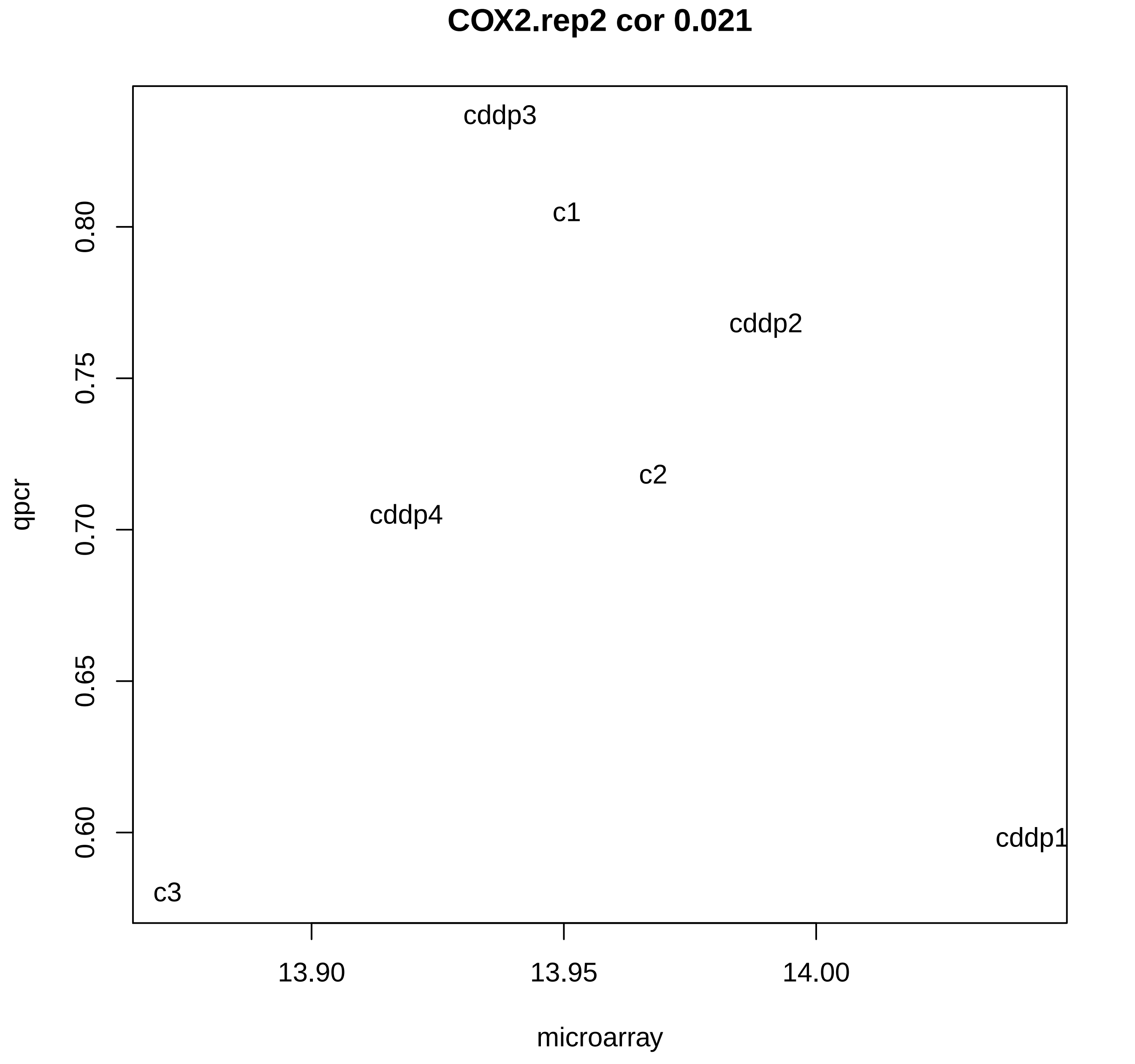
COX2.rep2 cor 0.021
cddp3
c1
cddp2
c2
cddp4
cddp1
c3
0.80
0.75
0.70
0.65
0.60
qpcr
13.90
13.95
14.00
microarray

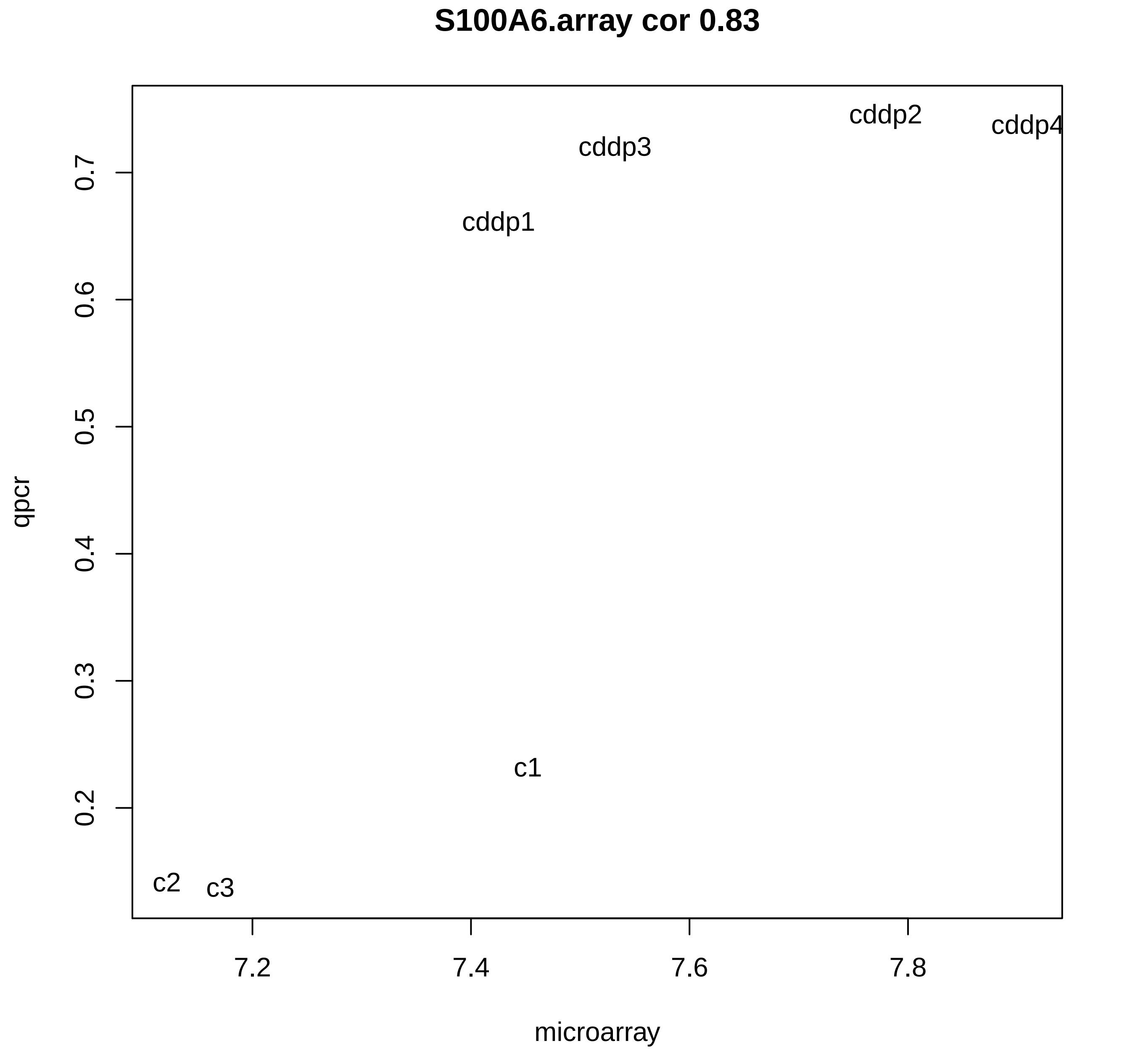

S100A6.array cor 0.83
cddp2
cddp4
cddp3
cddp1
c1
c2
c3
0.2
0.3
0.4
0.5
0.6
0.7
qpcr
7.2
7.4
7.6
7.8
microarray

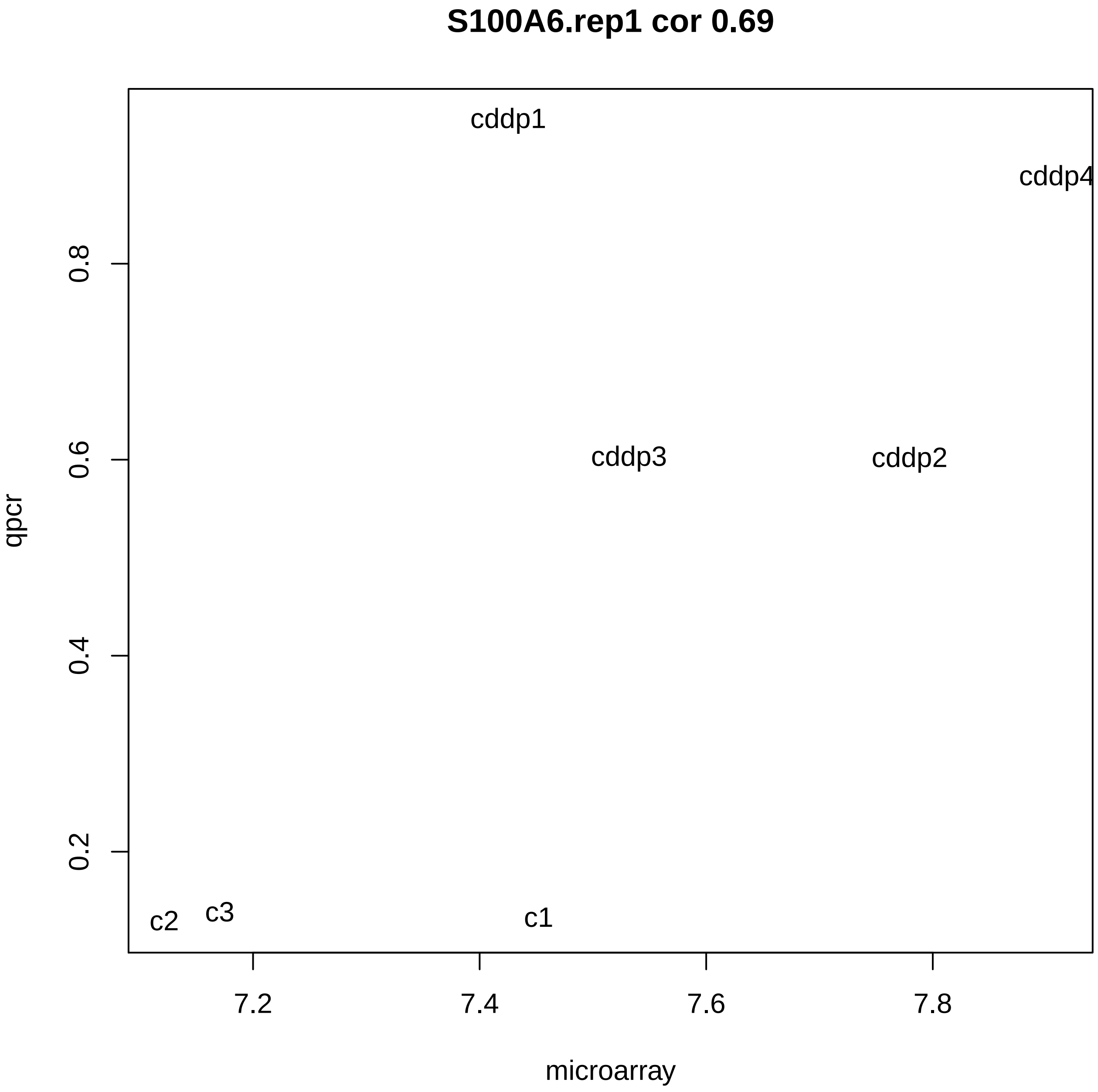
S100A6.rep1 cor 0.69
cddp1
cddp4
cddp3
cddp2
c3
c2
c1
0.2
0.4
0.6
0.8
qpcr
7.2
7.4
7.6
7.8
microarray

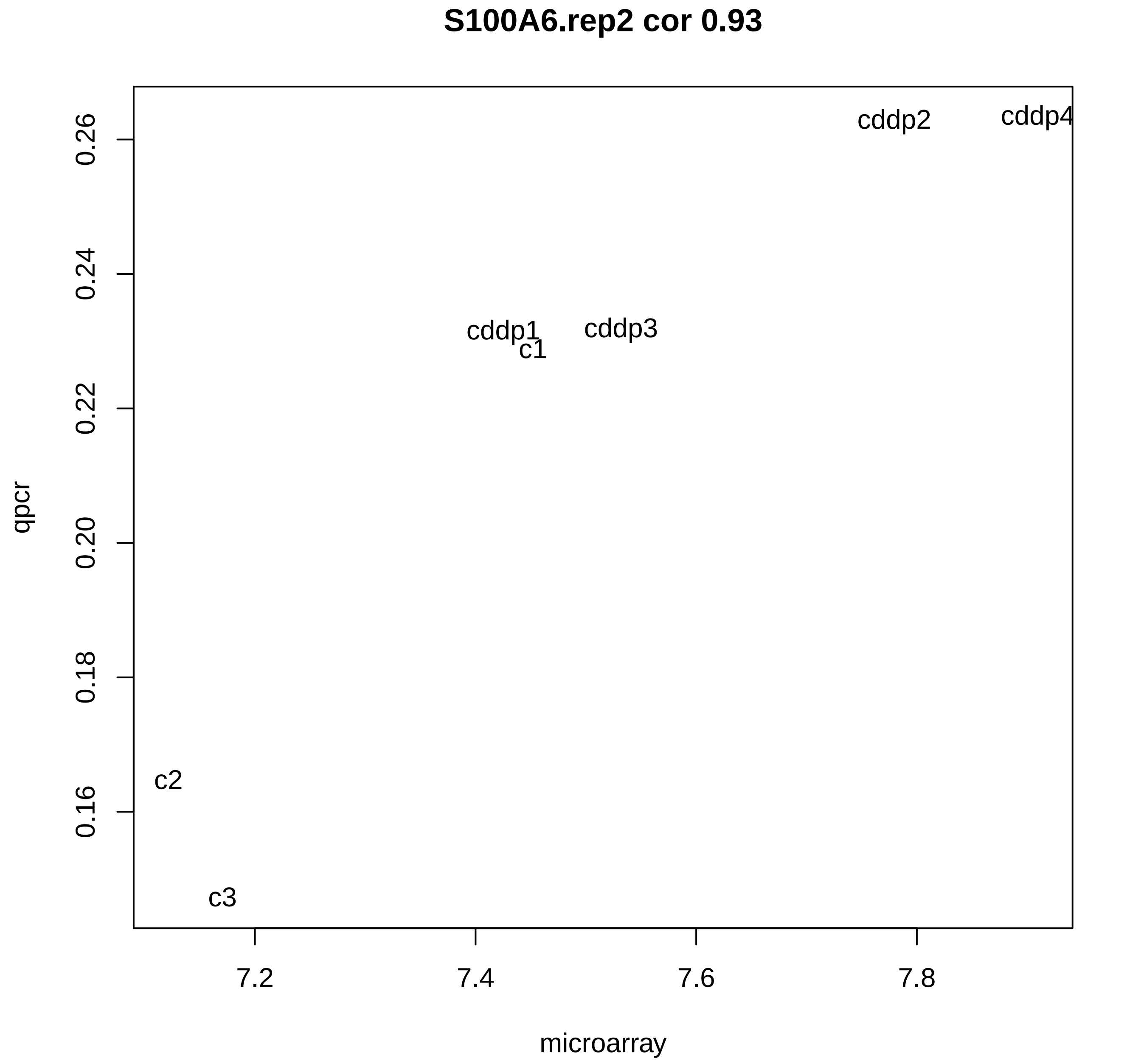
S100A6.rep2 cor 0.93
qpcr
0.16
0.18
0.20
0.22
0.24
0.26
microarray
7.2
7.4
7.6
7.8
cddp2
cddp4
cddp1
c1
cddp3
c2
c3

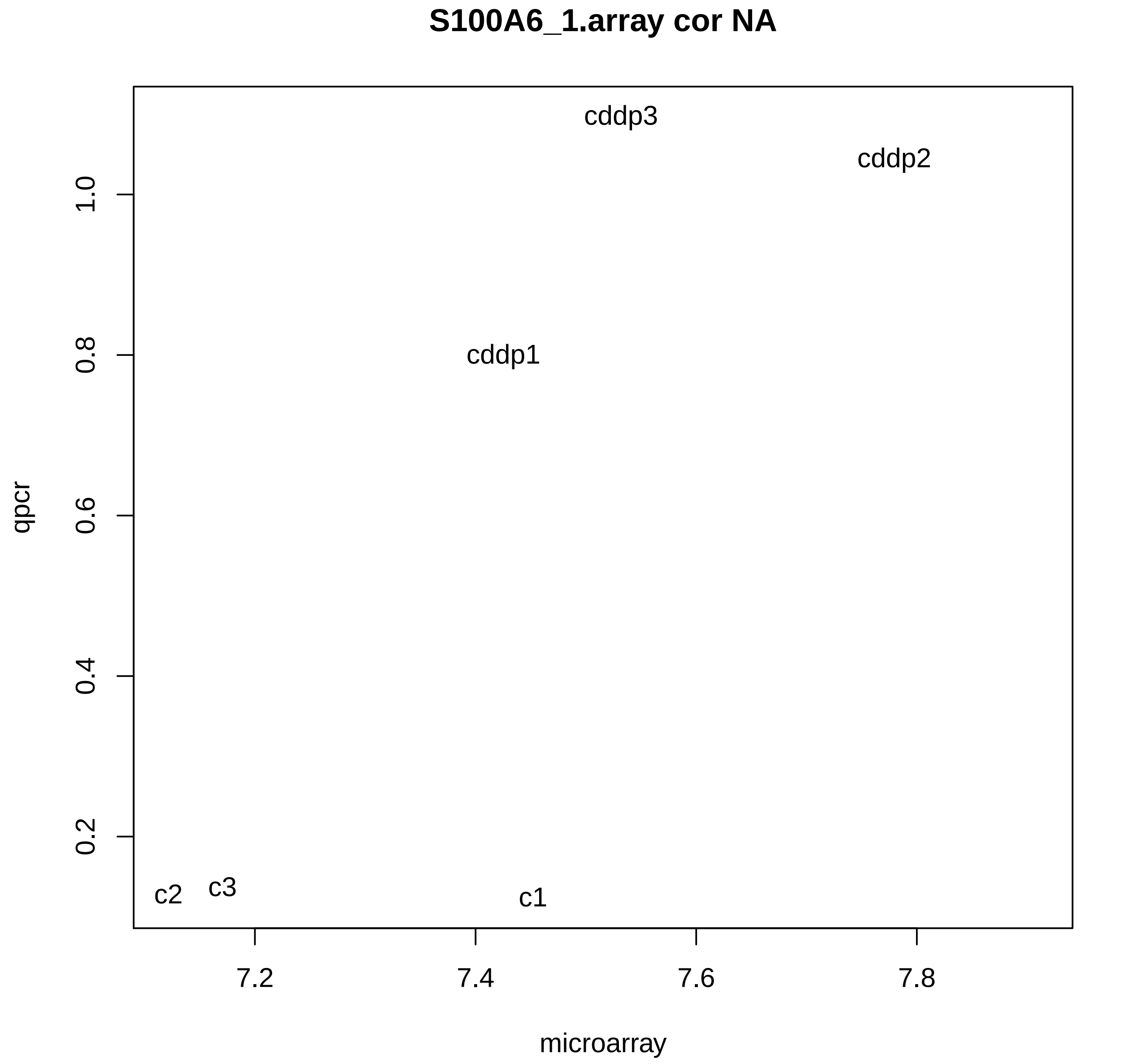
S100A6_1.array cor NA
cddp3
cddp2
cddp1
c2
c3
c1
1.0
0.8
0.6
0.4
0.2
qpcr
7.2
7.4
7.6
7.8
microarray

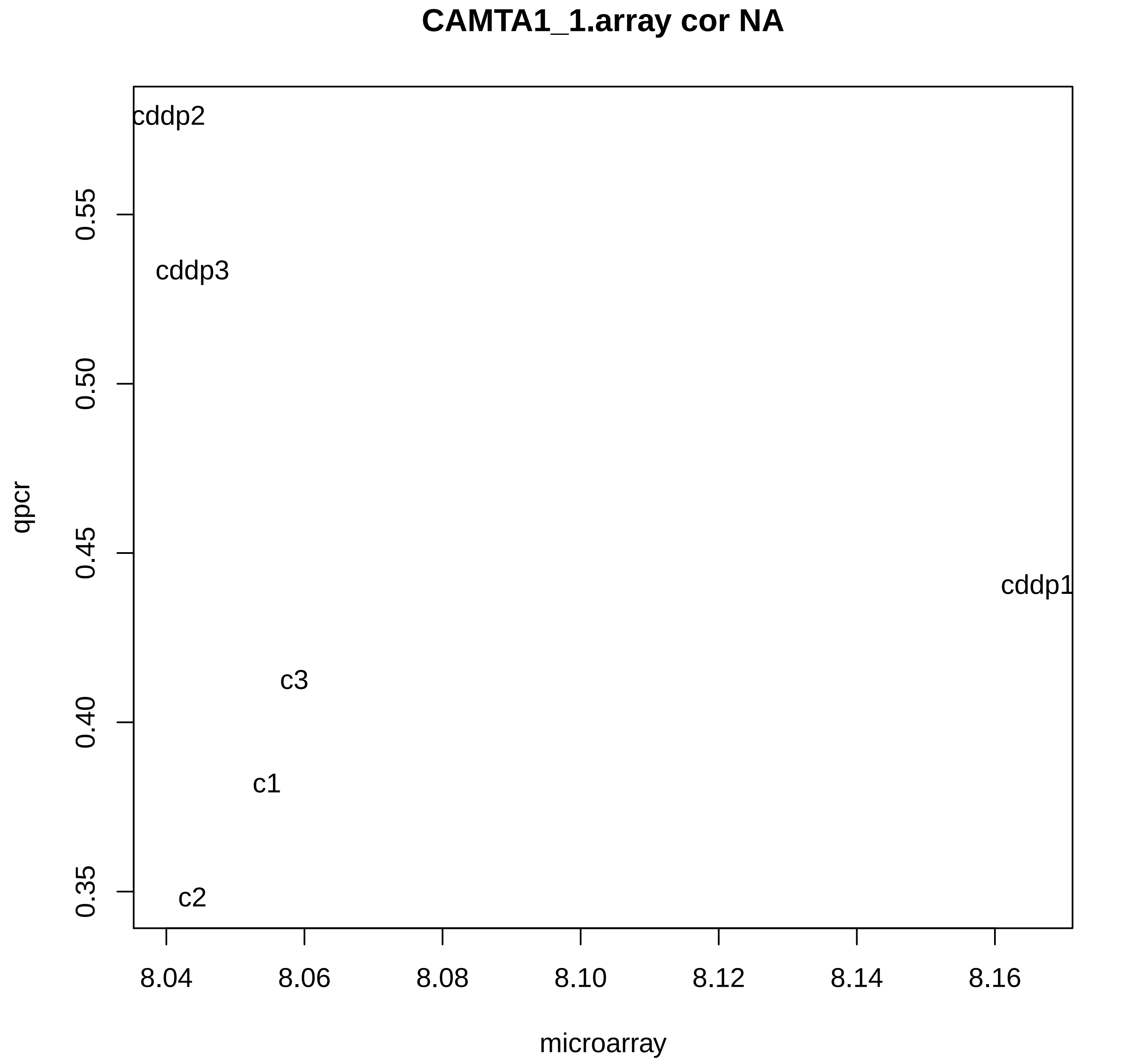
CAMTA1_1.array cor NA
cddp2
cddp3
cddp1
c3
c1
c2
0.55
0.50
0.45
0.40
0.35
qpcr
8.04
8.06
8.08
8.10
8.12
8.14
8.16
microarray

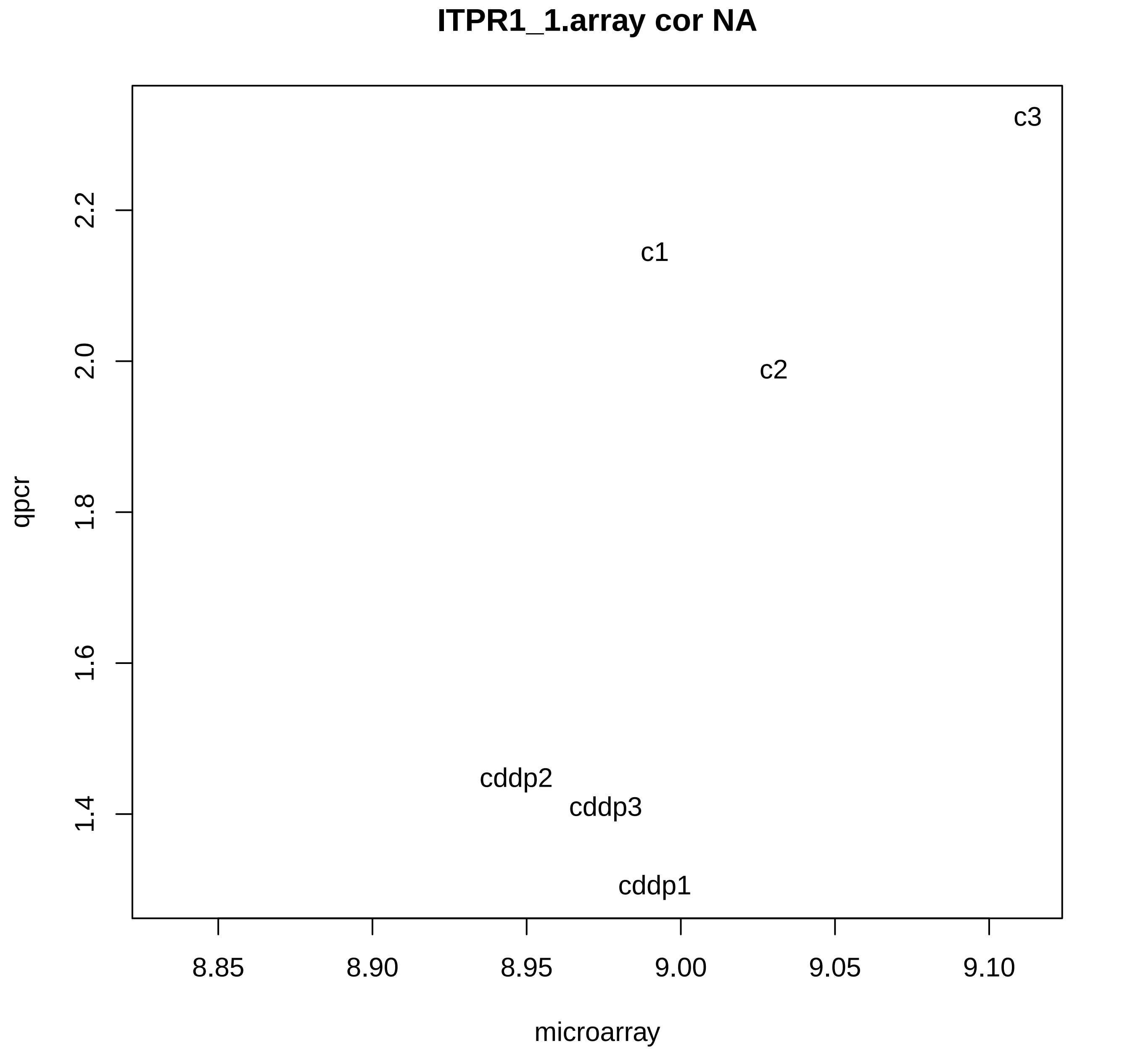
ITPR1_1.array cor NA
c3
c1
c2
cddp2
cddp3
cddp1
2.2
2.0
1.8
1.6
1.4
qpcr
8.85
8.90
8.95
9.00
9.05
9.10
microarray

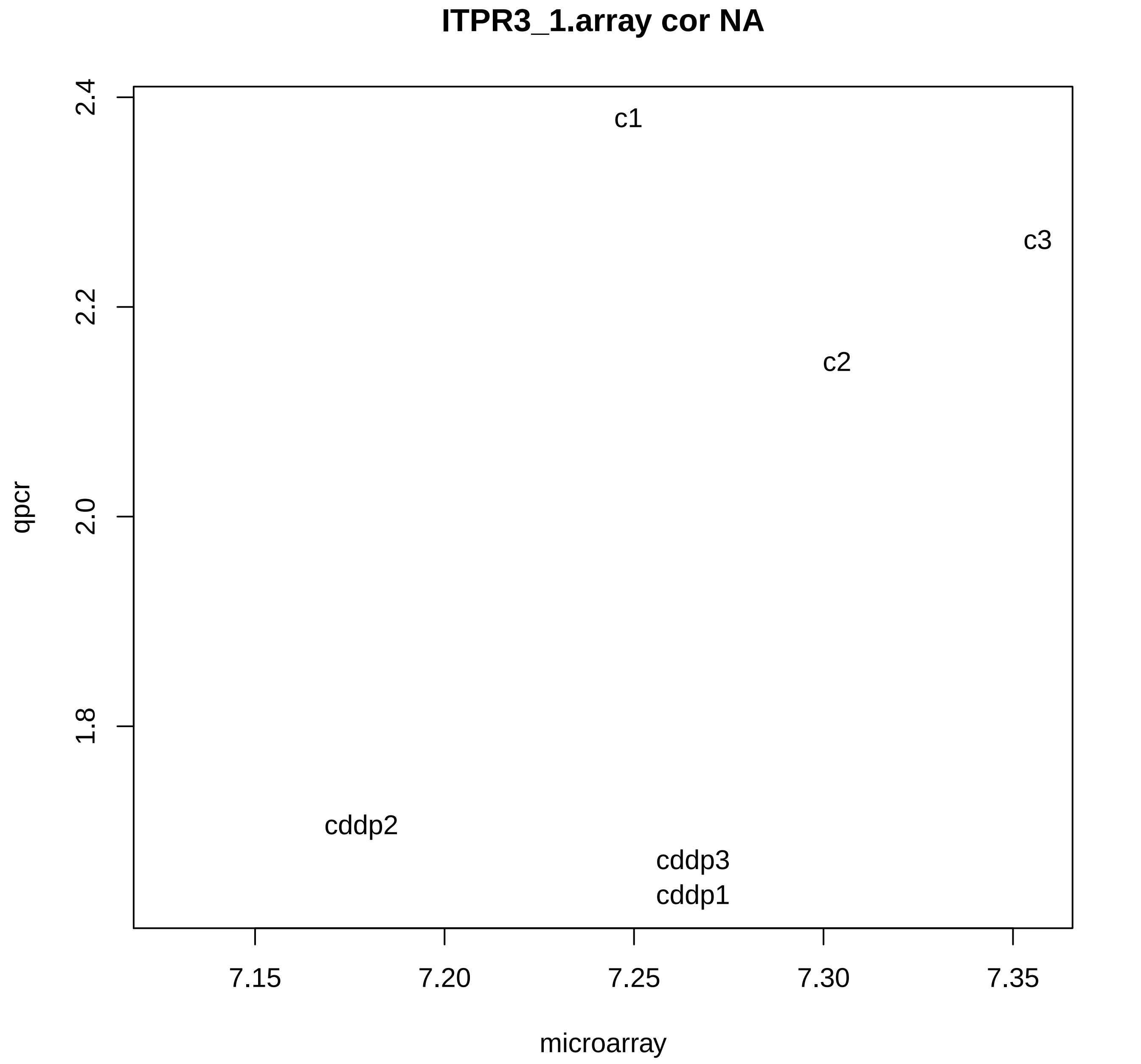
ITPR3_1.array cor NA
qpcr
2.4
2.2
2.0
1.8
c1
c3
c2
cddp2
cddp3
cddp1
7.15
7.20
7.25
7.30
7.35
microarray

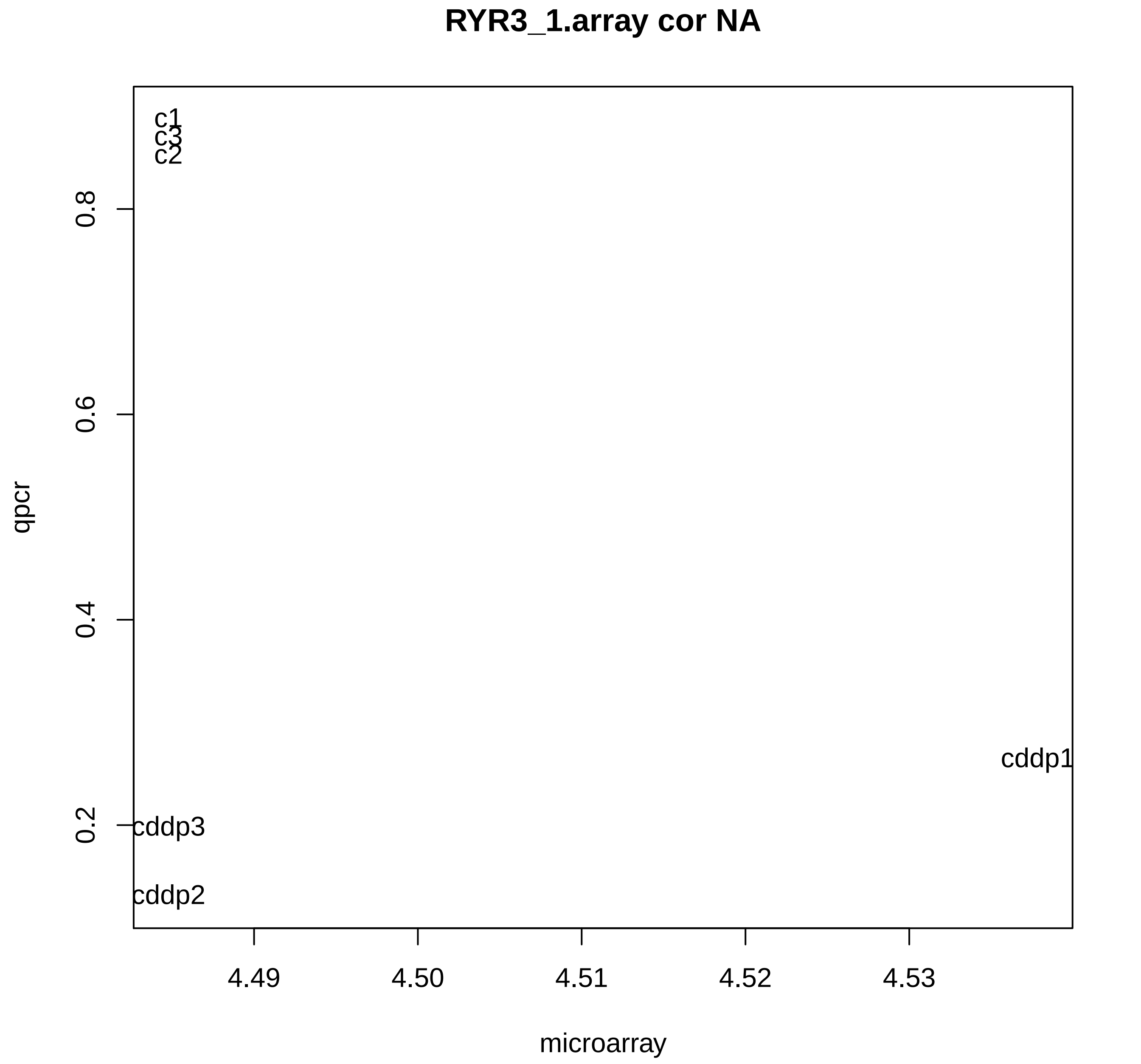

RYR3_1.array cor NA
c1
c3
c2
cddp1
cddp3
cddp2
0.8
0.6
0.4
0.2
qpcr
4.49
4.50
4.51
4.52
4.53
microarray

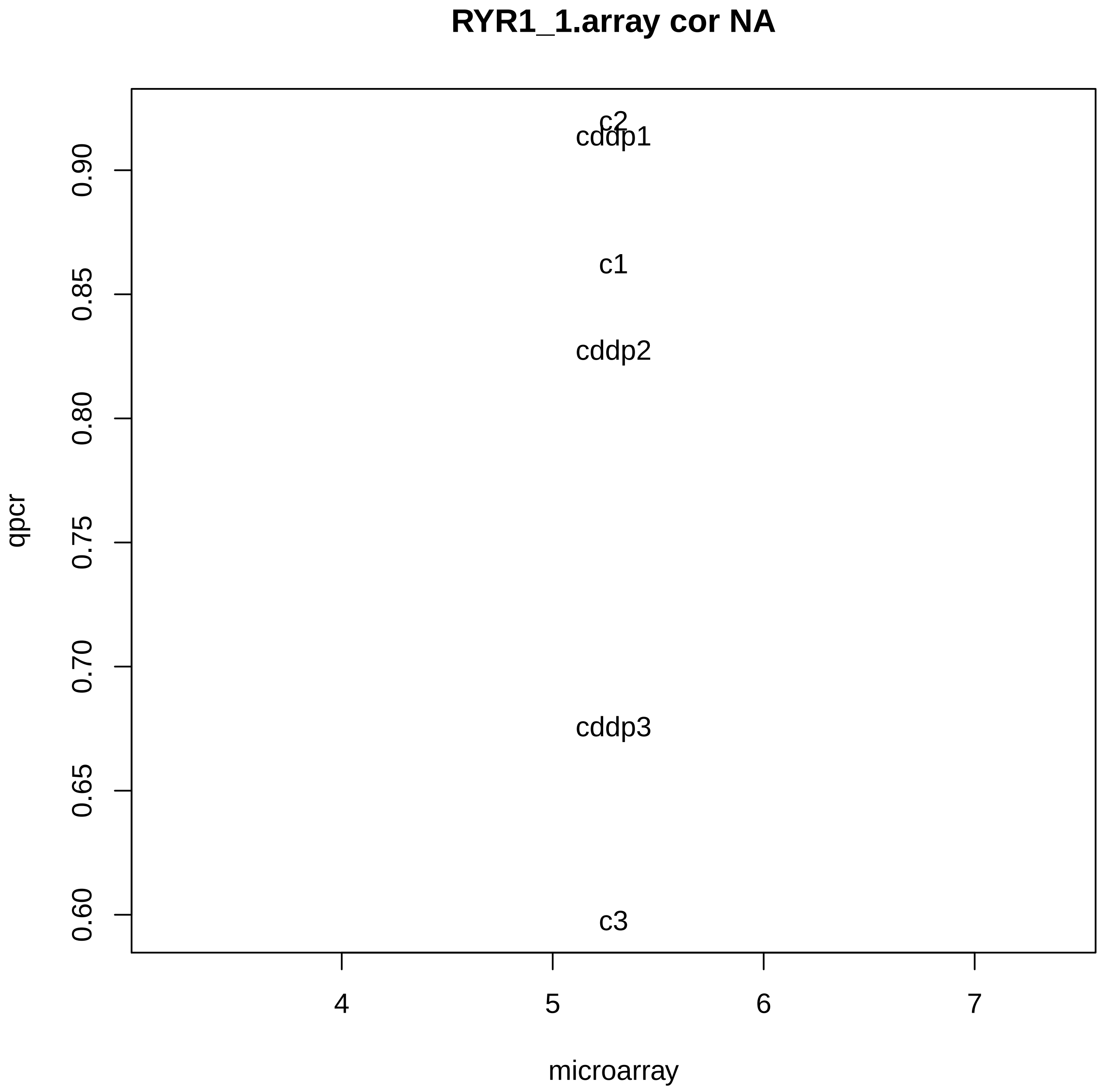

RYR1_1.array cor NA
c2
cddp1
c1
cddp2
cddp3
c3
0.90
0.85
0.80
0.75
0.70
0.65
0.60
qpcr
4
5
6
7
microarray

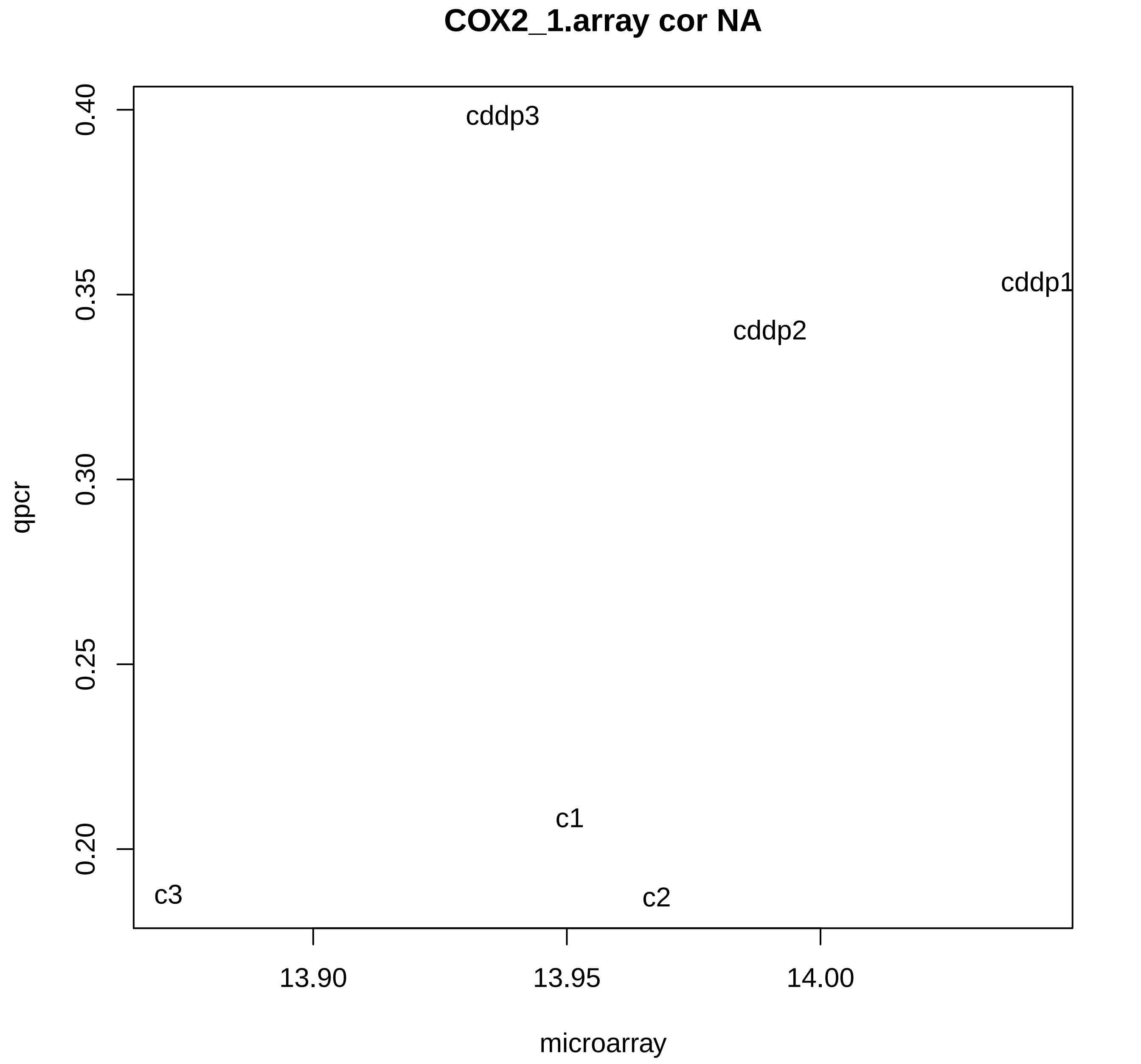
COX2_1.array cor NA
qpcr
0.40
0.35
0.30
0.25
0.20
cddp3
cddp1
cddp2
c1
c3
c2
13.90
13.95
14.00
microarray
